# Identification and comparative expression analysis of odorant binding protein genes in the tobacco cutworm *Spodoptera litura*

**DOI:** 10.1038/srep13800

**Published:** 2015-09-08

**Authors:** Shao-Hua Gu, Jing-Jiang Zhou, Shang Gao, Da-Hai Wang, Xian-Chun Li, Yu-Yuan Guo, Yong-Jun Zhang

**Affiliations:** 1 State Key Laboratory for Biology of Plant Diseases and Insect Pests, Institute of Plant Protection, Chinese Academy of Agricultural Sciences, Beijing, China; 2 Department of Biological Chemistry and Crop Protection, Rothamsted Research, Harpenden, UK; 3 Education Ministry Key Laboratory of Integrated Management of Crop Diseases and Pests, College of Plant Protection, Nanjing Agricultural University, Nanjing, China; 4 Department of Entomology and BIO5 Institute, University of Arizona, Tucson, AZ 85721 USA; 5 Beijing Autolab Biotechnology Company, Beijing, China

## Abstract

Insect odorant binding proteins (OBPs) are thought to involve in insects’ olfaction perception. In the present study, we identified 38 OBP genes from the antennal transcriptomes of *Spodoptera litura.* Tissue expression profiles analysis revealed that 17 of the 38 SlitOBP transcripts were uniquely or primarily expressed in the antennae of both sexes, suggesting their putative role in chemoreception. The RPKM value analysis revealed that seven OBPs (*SlitPBP1-3, SlitGOBP1-2, SlitOBP3* and *SlitOBP5*) are highly abundant in male and female antennae. Most *S. litura* antennal unigenes had high homology with Lepidoptera insects, especially genes of the genus *Spodoptera*. Phylogenetic analysis of the Lepidoptera OBPs demonstrated that the OBP genes from the genus *Spodoptera* (*S. litura*, *Spodoptera littoralis* and *Spodoptera exigua*) had a relatively close evolutionary relationship. Some regular patterns and key conserved motifs of OBPs in genus *Spodoptera* are identified by MEME, and their putative roles in detecting odorants are discussed here. The motif-patterns between Lepidoptera OBPs and CSPs are also compared. The *SlitOBPs* identified here provide a starting point to facilitate functional studies of insect OBPs at the molecular level both *in vivo* and *in vitro*.

Insects use their sensitive and selective olfactory organs, usually their antennae, to smell the outside world. Species-specific pheromones and general plant volatiles can diffuse inside the sensilla via the multipores to penetrate the cuticular surface of antennae^1^. When the odorant molecules enter the sensillum lymph, two types of processes occur. First, perireceptor events^2^ occur as the hydrophobic pheromones and/or the odorants bind to the soluble odorant binding proteins (OBPs), which deliver the pheromones and odorants to the membrane-bound olfactory receptors (ORs). After activating the ORs, the odorant molecules are subsequently quickly degraded by the odorant degrading enzymes (ODEs)^3^. After these processes, receptor events occur by the specific interaction of odorants with ORs, leading to the activation of the chemoelectric transduction machinery in the olfactory receptor neurons (ORNs)^4^. Although many researchers have focus on these two events, the events prior to membrane receptor stimulation are still poorly understood.

Insect OBPs are highly concentrated in antennal sensilla and are proposed to be involved in the first biochemical step of perireceptor events in odorant reception^5^,^6^. Insect OBPs were first identified in the silkmoth *Antheraea polyphemus*, where they are uniquely expressed in male antennae and bind with the sex pheromones, so these OBPs are named as pheromone binding proteins (PBPs)^7^. Based on the similarity of their amino acid sequences, moth OBPs can be grouped into three major classes, pheromone binding proteins (PBPs)^7^, general odorant binding proteins (GOBP1 and GOBP2)^8^, and antennal binding proteins X (ABPX)^9^. There is no structural homology between insect OBPs and vertebrate OBPs^6^,^10^. A typical feature of insect OBPs is the presence of six highly conversed cysteine residues, which are paired in three interlocked disulfide bridges^11^. This sequence motif has been used for genome/transcriptome-wide identification and annotation of OBP genes in a range of insect species^12^,^13^,^14^.

Insect OBPs are presumed to be synthesized by non-neuronal auxiliary cells (trichogen and tormogen cells) of the sensory neurons and secreted into the sensillum lymph with a very high concentration (up to 10 mM)^7^,^15^. Several functions of insect OBPs in odorant and pheromone perception have been proposed, including (i) transporting hydrophobic odorant molecules across the sensillum lymph to the ORs, which contributes to the sensitivity of insect ORs^4^,^16^; (ii) solubilizing hydrophobic odorants^17^; (iii) concentrating odorants in the sensillum lymph^17^; and (iv) removing or deactivating odorants after simulating the receptors^7^,^18^. However, till now, the physiological and behavioral evidences that support those functions are still exclusive. In the fire ant *Solenopsis invicta*, the pheromone binding protein gene *Gp-9* has been shown to regulate the social organization in the colony between the monogyne social form (with a single queen) and the polygyne form (with multiple queens)^19^. The *Drosophila* OBP76a/LUSH mutants have shown a complete loss of sensitivity to their sex pheromone 11-*cis* vaccenyl acetate (VA), indicating that OBP76a/LUSH is absolutely required to activate the pheromone-sensitive sensory neurons in the fruit fly^20^. However, the function of insect OBPs in olfactory reception may be more complicated than previously image, because some odorants, such as 3-hydroxy-2-butanone released by the male cockroach *Nauphoeta cinerea*, and some ethanol released from ripe fruits, are water soluble, even though they still need to be detected by OBPs or PBPs^21^,^22^. It is also noteworthy that synthetizing such extremely high concentrations of OBPs requires the consumption of large amounts of energy, suggesting an important physiological role, both for the survival of the individual and the conservation of the species^6^,^23^.

The *Spodoptera litura* (Fabricius) (Lepidoptera: Noctuidae), commonly known as tobacco budworm, common cutworm or cotton cutworm, is a model of a serious polyphagous pest that can attack more than 389 host plants of 109 plant families^24^, This pest causes yearly widespread losses of a variety of cash crops yields, especially cotton, tobacco, chili, tea, tobacco, cabbage^25^. Normally, chemical insecticides are the main methods to control this pest^26^,^27^; however, it has developed high levels of resistance to many traditional insecticides including organophosphates, pyrethroids, carbamates and some newly introduced insecticides, resulting in failure to effectively control this pest^26^,^27^. The negative impacts of overuse of insecticides on the environment and human health drive us to develop a safe and environmentally friendly intervention strategy against this pest. The olfaction-based approaches, using synthetic sex pheromones and host volatiles to interfere with insects’ ability to find suitable mates and hosts, have been applied successfully in the “push-pull” pest control strategy^28^. The *S. litura* moth is very attracted to particular odorant released by the host plant^29^, and the male *S. litura* moth is very sensitive to sex pheromones released by virgin females^30^. These make sex pheromones and host plant volatiles are effective biological control agents to control this pest through population monitoring and mass trapping in integrated pest management (IPM) programs^31^,^32^. However, the molecular and cellular mechanisms of *S. litura* finding mates and host plants are still unknown.

In present study, we identified and annotated 38 OBP genes from the antennae of the *S. litura* male and female moth using next-generation sequencing (NGS) platform 454 GS FLX and Hiseq2000. Their expression profiles in different tissues were investigated using semi-quantitative RT-PCR and real-time quantitative-PCR, their putative functions in chemoreception are proposed, the motif pattern and the evolutionary relationship of Lepidoptera OBPs are also discussed.

## Results

### Unigene assembly and annotation

**Figure 1:**
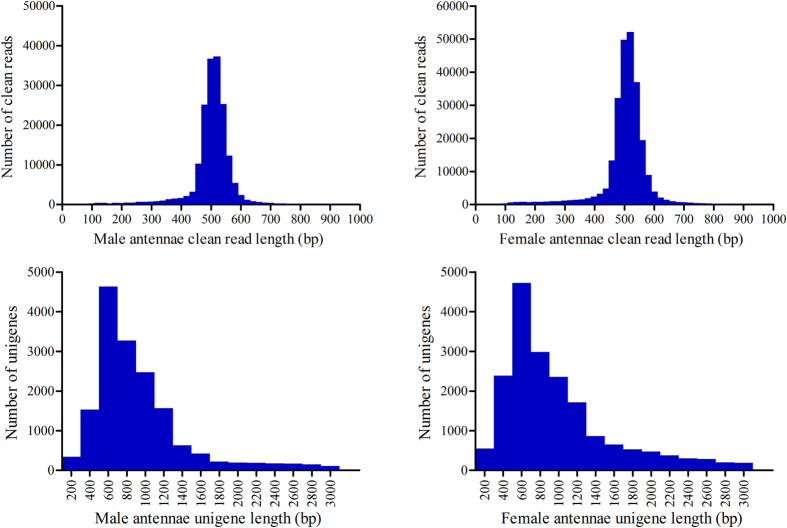
The size distribution of the assembled unigenes from *S. litura* male and female antennal transcriptome.

The antennal cDNA libraries of the male and female *S. litura* moth were sequenced using the 454 GS FLX Titanium platform. After 1/4 sequencing run of each sex, a total of 178345 (mean length 516 bp) and 253266 raw reads (mean length 514 bp) were produced from the male and female antennae samples, respectively. After trimming the adaptor sequences, contaminating sequences and low quality sequences, 177227 (mean length 494 bp) and 251805 clean reads (mean length 495 bp) were remained for the following assembly from male and female antennae, and produced 16478 (mean length 864 bp) and 19000 (mean length 808 bp) unigenes, respectively (Fig. 1). Additionally, we assembled all clean reads from the male and female antennae together and ultimately generated 21223 unigenes. Among these unigenes, 19393 were contigs (91.4%) and 1830 were singletons (8.6%). The assembled unigene lengths ranged from 100 bp to 10423 bp with an average length of 766 bp. An overview of the sequencing and assembly process is presented in Table 1. We also downloaded and assembled a recently released Hiseq2000 transcriptome data of *S. litura* antennae in the Short Read Archive (SRA) database at NCBI (Release data: 05/23/2015) and obtained 75028 unigenes with an average length of 589 bp. The two assemblies are combined and used for the OBP identification of *S. litura*.

### Homology searching of *S. litura* antennal unigenes with other insect species

**Figure 2:**
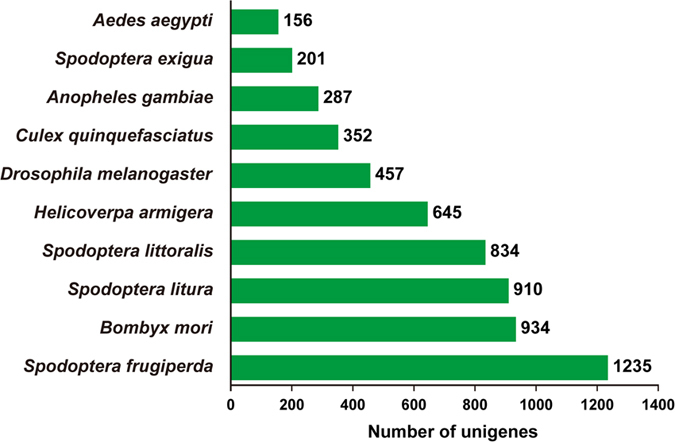
Top 10 best hits of the BLASTn results. All *S. litura* antennal unigenes were used in BLASTn search the GenBank entries. The best hits with an E-value < = 1.0E-5 for each query was grouped according to species.

Homology searching of the 21223 *S. litura* antennal unigenes with other insect species was conducted using the BLASTx and BLASTn programs with the E-value cut-off of 10e-5^33^. The results indicated that 10438 of the 21223 unigenes (49.2%) had BLASTx hits in the non-redundant protein (nr) databases and 8957 unigenes (42.2%) had BLASTn hits in the non-redundant nucleotide sequence (nt) databases. Most annotated *S. litura* antennal unigenes had the highest hits with the Lepidoptera insect genes (5173 of the 8957 nt-hit unigenes); the highest number of hits include 1235 unigenes that were homologous to *Spodoptera frugiperda* genes, 934 unigenes that were homologous to *Bombyx mori* genes, 910 and 834 unigenes that were homologous to *S. litura* and *S. littoralis* genes, respectively. The second highest number of hits were with the Dipteran species genes, with 457 hits of the *D. melanogaster* genes, and 352 and 287 hits that were homologous to genes of the mosquitoes *Culex quinquefasciatus* and *Anopheles gambiae*, respectively (Fig. 2).

### Functional annotation of the *S. litura* antennal unigenes

**Figure 3:**
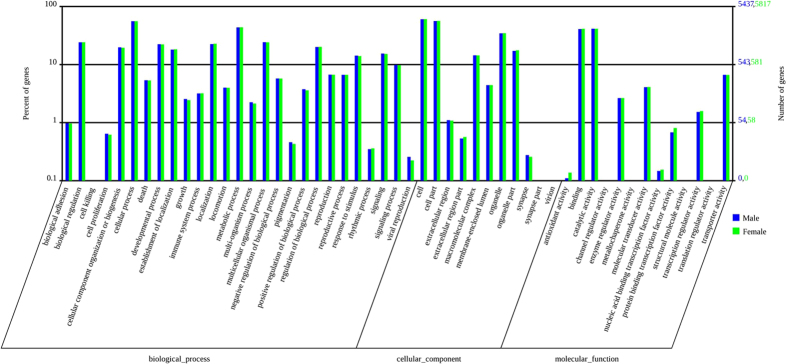
Gene Ontology (GO) classifications of *S. litura* antennal unigenes according to their involvement in biological processes, cellular component and molecular function.

Similar to those genes that were found in the antennal transcriptomes of *Manduca sexta*^34^, *S. littoralis*^35^, *Helicoverpa armigera*^36^ and *Agrotis ipsilon*^14^, only 6266 of the 21223 *S. litura* antennal unigenes (29.3%) could be annotated into different functional groups (biological process, cellular components and molecular functions) according to Gene Ontology (GO) category analysis^37^(Fig. 3). Some transcripts were annotated into more than one GO category. The numbers of each GO category were similar between the male and female antennal transcriptomes (Fig. 3). The cellular process (3035 male antennal unigenes and 3251 female antennal unigenes) and metabolic process (2374 male antennal unigenes and 2548 female antennal unigenes) GO categories were most abundantly represented within the biological process GO ontology. In the cellular components GO ontology, the transcripts were primarily distributed in the cell (3279 male antennal unigenes and 3517 female antennal unigenes) and in cell part (3055 male antennal unigenes and 3280 female antennal unigenes). The GO analysis also showed that the binding (2221 male antennal unigenes and 2400 female antennal unigenes) and catalytic activity (2255 male antennal unigenes and 2404 female antennal unigenes) were most abundant in the molecular function ontology (Fig. 3).

### Identification of *S. litura* odorant binding proteins

**Figure 4:**
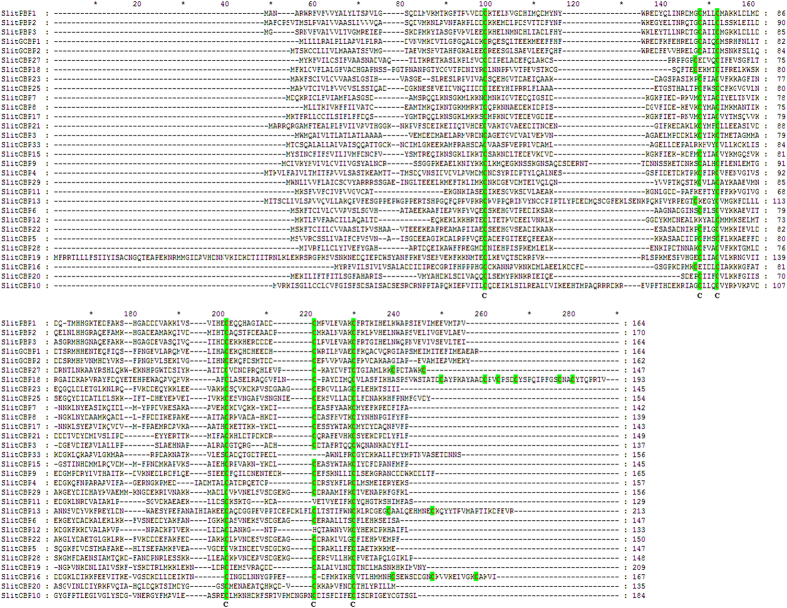
Alignment of the *S. litura* OBPs. Full-length amino acid sequences of *S. litura* OBPs are aligned by ClustalX 2.1. Green boxes show conserved cysteine residues. Accession numbers of the *S. litura* OBPs are listed in Table 2.

We identified 38 putative OBP genes in the *S. litura* 454 and Hiseq2000 antennal transcriptome data. These include three pheromone binding proteins (PBPs) and two general odorant binding proteins (GOBPs) (Table 2). Thirty of the 38 OBP genes (except *SlitOBP1, SlitOBP2, SlitOBP14, SlitOBP24, SlitOBP26, SlitOBP30-32*) have intact ORFs with lengths ranging from 387 bp to 1017 bp, all the full-length OBPs have a signal peptide at their N-terminal, a signature of secretory proteins (Table 2). Based on the number and location of the conserved cysteines, the 30 full-length SlitOBPs can be divided into three families: SlitOBP11, SlitOBP12 and SlitOBP33 belong to the Minus-C OBP family, which have no conserved cysteines C2 and C5 (Fig. 4); SlitOBP13, SlitOBP16, SlitOBP18 and SlitOBP27 belong to the Plus-C OBP family, which have additional 2, 3, 6 and 2 cysteines located downstream of conserved C6 in addition to the six conserved cysteines. Furthermore, the conserved C2 and C3 of the four Plus-C OBPs are separated by 4 amino acid residues rather than usual 3 of the Classic OBP, and the conserved C5 and C6 of three Plus-C OBPs (SlitOBP16, SlitOBP18 and SlitOBP27) are separated by 7 amino acid residues rather than usual 8 as in the Plus-C OBP SlitOBP13 and the non-Plus-C SlitOBPs (Fig. 4); The remaining 23 SlitOBPs belong to the Classic OBP family, which all having typical six conserved cysteines and spacing between them (Fig. 4).

The BLASTx results indicated that the 38 identified SlitOBPs shared relatively high amino acid identities (32%–98%) with Lepidoptera OBPs at NCBI. Thirty-three of the 38 *SlitOBPs* (except three PBPs and two GOBPs) were first identified in *S. litura*. All of the 38 *SlitOBPs* were manually checked by the BLASTx program and then named according to the highest protein similarities of the best BLASTx hit. The nucleotide sequences of the 38 *SlitOBPs* were confirmed by cloning and sequencing and had been deposited in the GenBank under the accession numbers KP331511 to KP331523 and KT192030 to KT192054.

### Motif pattern analysis of *S. litura* OBPs

**Figure 5:**
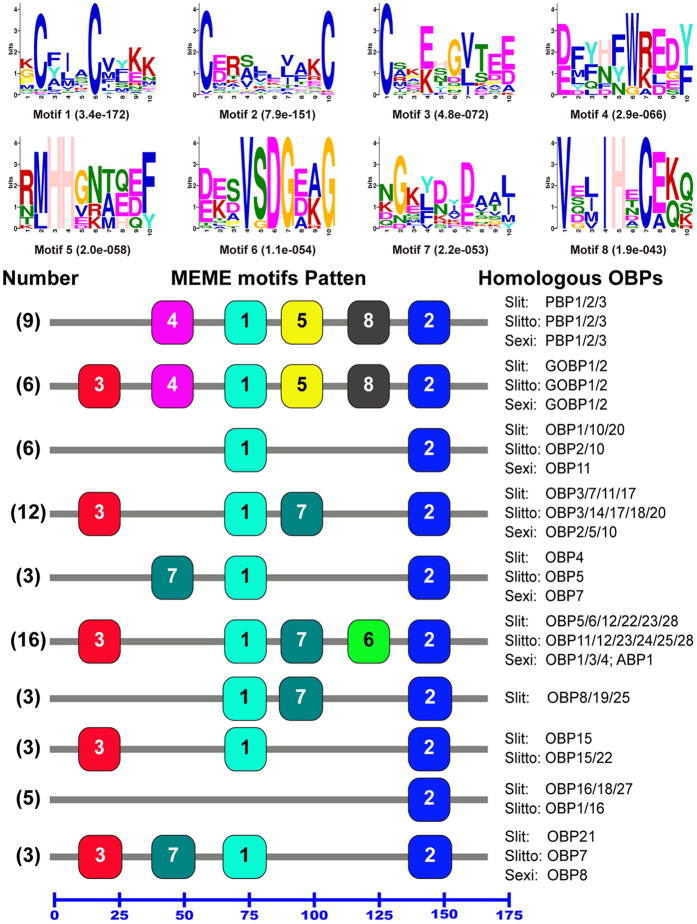
Motif analysis of OBPs in the genus *Spodoptera*. Parameters used for motif discovery were: minimum width = 6, maximum width = 10, maximum number of motif to find = 8. The upper parts listed the eight motifs discovered in the 78 OBPs using MEME^63^ (version 4.9.1) on line server (http://meme.nbcr.net/meme/). The lower parts indicate approximate locations of each motif on the protein sequence. The numbers in the boxes correspond to the numbered motifs in the upper part of the figure, where small number indicates high conservation. The numbers on the bottom showed the approximate locations of each motif on the protein sequence, starting from the N-terminal. This figure just listed the most common 10 motif-patterns which presented in 66 OBPs, with each motif-pattern present in more than two OBPs, the remaining 12 OBPs had 8 different motif-patterns with each of them presented in less than three OBPs. The protein names and sequences of the 78 of OBPs from *S. litura*, *S. littoralis* and *S. exigua* were listed in Supplementary Table 4.

The conserved motifs are important elements of functional domains. In order to compare the motif-pattern of OBP proteins in the genus *Spodoptera*, a total of 78 OBPs with intact ORFs from *S. litura* (31 OBPs), *S. littoralis* (30 OBPs) and *S. exigua* (17 OBPs) were combined into one set of sequences and then submitted to the MEME server to discover the conserved motifs. The results indicated that eight motifs were found and the OBPs in each species had different motif-patterns, but the homologous OBPs among *S. litura*, *S. littoralis* and *S. exigua* had similar motif-patterns (Fig. 5). Eighteen different motif-patterns were presented in the tested 78 OBPs, in Fig. 5 we just listed and analysis the most common 10 motif-patterns which presented in 66 OBPs, with each motif-pattern present in more than two OBPs, the remaining 12 OBPs had 8 different motif-patterns with each of them presented in less than three OBPs. The three PBPs of the three species had the same motif-pattern with the motif order as 4-1-5-8-2, the GOBP1/2 of the three species had one more motif 3 at the N-terminal compared with PBP1/2/3, with the motif order as 3-4-1-5-8-2. Twelve homologous OBPs (SlitOBP3/7/11/17, SlittoOBP3/14/17/18/20 and SexiOBP2/5/10) had four motifs with an order as 3-1-7-2; sixteen homologous OBPs (SlitOBP5/6/12/22/23/28, SlittoOBP11/12/23/24/25/28, SexiABP1 and SexiOBP1/3/4) had five motifs with the order as 3-1-7-6-2 (Fig. 5). We also found some interesting regular patterns as follows in Fig. 5: the motif 2 and motif 1 existed in 61 out of 66 OBPs and located at same position (motif 2 at the C-terminal and motif 1 in the middle) with the exception of SlitOBP16/18/27 and SlittoOBP1/16 that did not have motif 1. The motif 4, 5 and 8 were only found in the PBP1/2/3 and GOBP1/2 in the three species and located in the N-terminal, central part and the C-terminal, respectively. Interestingly GOBPs had motif 3 which is common for OBPs and motif 4-1-5-8-2 which is common for PBPs. The motif 7 was only found in OBPs but in PBP/GOBP complex.

**Figure 6:**
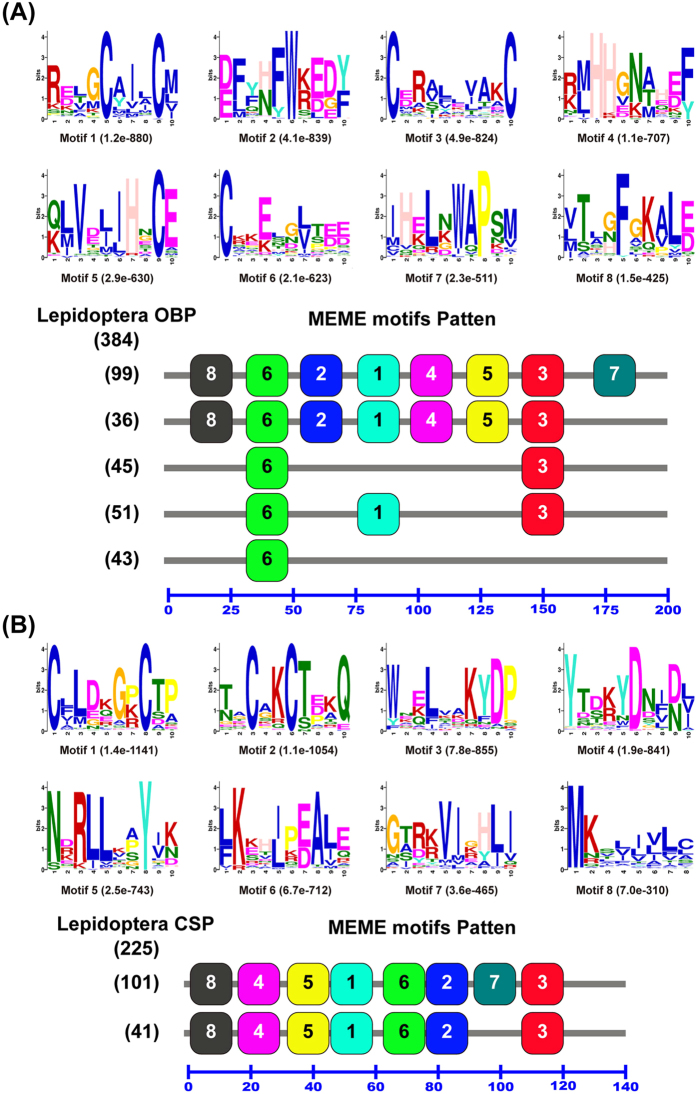
Motif analysis of Lepidoptera OBPs and CSPs. Parameters used for motif discovery were: minimum width = 6, maximum width = 10, maximum number of motif to find = 8. The upper parts in (**A**,**B**) listed the eight motifs discovered in the Lepidoptera OBPs and CSPs, receptively. All the motifs were discovered using MEME^63^ (version 4.9.1) on line server (http://meme.nbcr.net/meme/). The lower parts indicate approximate locations of each motif on the protein sequence. The numbers in the boxes correspond to the numbered motifs in the upper part of the figure, where small number indicates high conservation. The numbers on the bottom showed the approximate locations of each motif on the protein sequence, starting from the N-terminal. The protein names and sequences of the 384 OBPs and 225 CSPs from 36 different Lepidoptera species were listed in Supplementary Table 5.

When compared the motif-patterns of 384 OBPs from 36 Lepidoptera species we found 17 different motif-patterns, and 274 OBPs (71.4%) had the most common five motif-patterns, with 99 OBPs had the same motif-pattern as 8-6-2-1-4-5-3-7, thirty-six OBPs had the same motif-pattern as 8-6-2-1-4-5-3, forty-five OBPs had only two motifs with the order as 6-3, fifty-one OBPs had three motifs with the order as 6-1-3, forty-three OBPs only had one motif motif 6 (Fig. 6A). The remaining 61 OBPs shared the other 12 motif-patterns and none motif was found in 49 OBPs. While the motif-patterns of the 225 Lepidoptera CSPs were more conserved than the OBPs, 142 CSPs (63.1%) had the most common two motif-patterns, with 101 CSPs had motif-pattern as 8-4-5-1-6-2-7-3, and 41 CSPs had motif-pattern as 8-4-5-1-6-2-3. The 41 CSPs lost motif 7 compared with the 101 CSPs (Fig. 6B). The remaining 83 CSPs shared other 25 different motif-pattern. It should be noticed that the motif-patterns discovered by MEME in Figs 5 and 6 are not comparable, because different sets of sequences were used in each analysis.

### Phylogenetic analyses of *S. litura* OBP genes

The 30 full-length SlitOBPs share a relatively low amino acid identities (5%–59%) with each other, the three PBPs SlitPBP1-3 showed overall 46% identities with each other and the SlitGOBP1 showed 50% identity with SlitGOBP2 (Supplementary Table 1). The amino acid identities of homologous OBPs in genus *Spodoptera* (*S. litura*, *S. littoralis* and *S. exigua*) is relatively high, for example, SlitOBP12 showed 100% amino acid identity with SlittoOBP12 (Supplementary Table 2).

**Figure 7:**
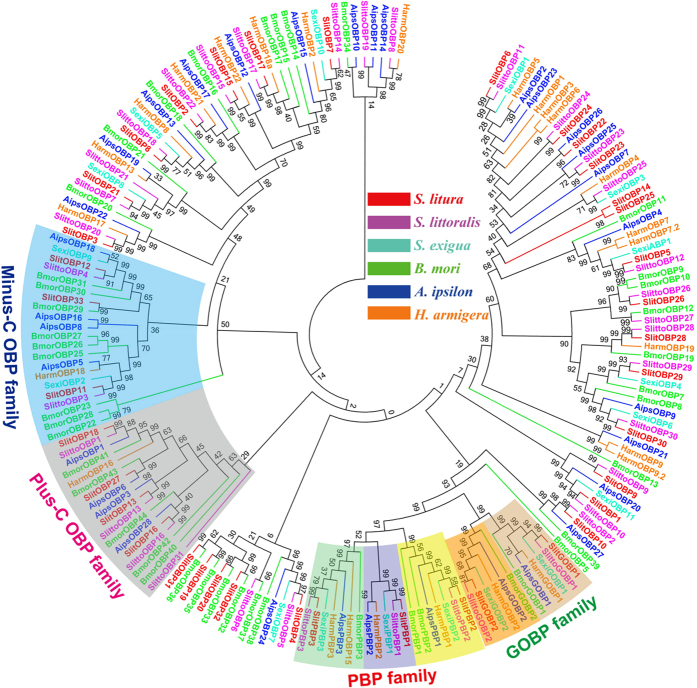
Neighbor-joining tree of candidate OBP proteins from Lepidoptera species. The protein names and sequences of the 193 OBPs that were used in this analysis are listed in Supplementary Table 8.

A neighbor-joining tree of 193 OBP sequences was built from six different Lepidoptera species, including *B. mori*, *A. ipsilon*, *H. armigera* and the three closely related species of genus *Spodoptera* (*S. litura*, *S. littoralis* and *S. exigua*) (Fig. 7). It was shown that Lepidoptera OBPs can be divided into several distinct families, including the GOBP family, the PBP family, the Minus-C OBP family and the Plus-C OBP Family. The identified SlitPBP1-3 are clustered into the PBP family and the SlitGOBP1-2 are clustered into the GOBP family, respectively. In the PBP family, the PBP1, PBP2 and PBP3 from the genus *Spodoptera* (*S. litura*, *S. littoralis* and *S. exigua*) are each located in the same branch with the bootstrap values as high as 100. In the GOBP family, the GOBP1 and GOBP2 from the genus *Spodoptera* are each located in the same branch with the bootstrap values as high as 95. Three OBPs (SlitOBP11, SlitOBP12 and SlitOBP33) are clustered into the insect Minus-C OBP family, four OBPs (SlitOBP13, SlitOBP16, SlitOBP18 and SlitOBP27) are clustered into the insect Plus-C OBP family. The remaining 26 SlitOBPs are located in the same branch with corresponding homologous OBPs from the *S. littoralis* and *S. exigua* (Fig. 7). We found no species specific expansion and few numbers of gene duplication (SlitOBP14 and SlitOBP25).

### Transcripts expression levels of *S. litura* OBPs

**Figure 8:**
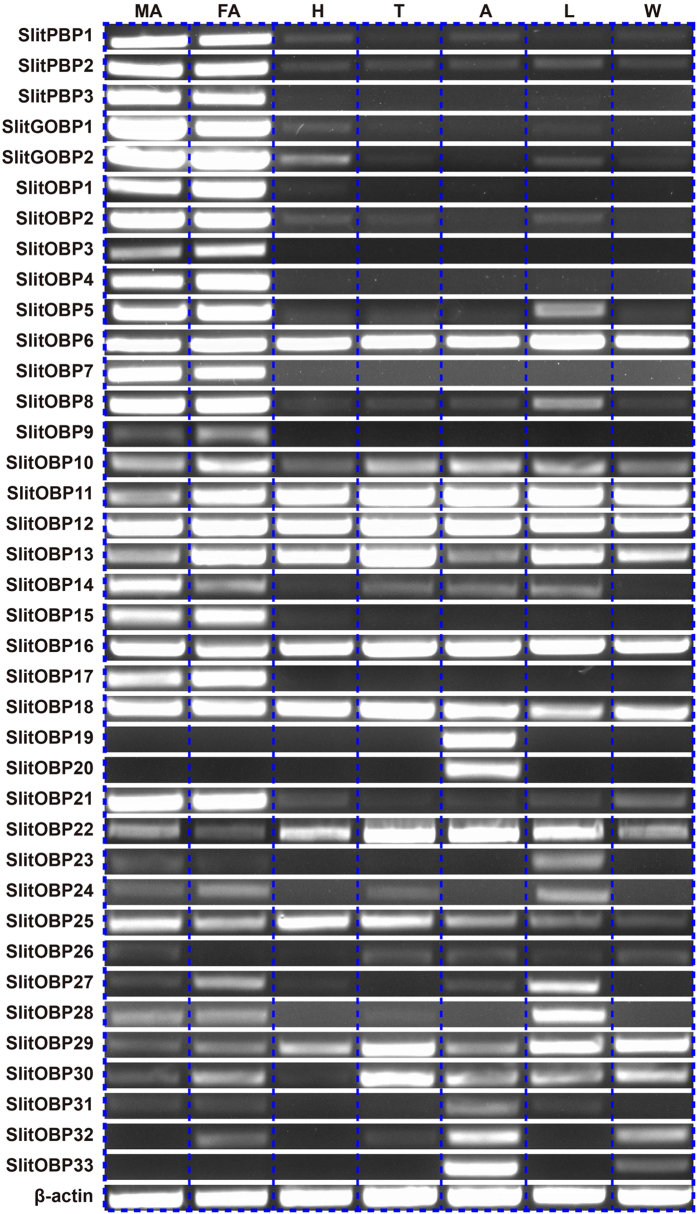
*S. litura* OBP transcript levels in different tissues as evaluated by RT-PCR. MA: male antennae; FA: female antennae; H: heads; T: thoraxes; A: abdomens; L: legs; W: wings. *β-actin* was used as an internal reference gene to test the integrity of each cDNA template; the similar intensity of *β-actin* bands among different tissues indicates the use of equal template concentrations. The RT-PCR for all SlitOBPs are run under the same experimental conditions. The display for each SlitOBPs are cropped figures from the gels. The full-length gels are presented in Supplementary Figure 1.

The expression of the 38 identified SlitOBP genes in different tissue types were examined using RT-PCR (Fig. 8). The results indicated that 17 of the 38 SlitOBP genes (*SlitPBP1-3, SlitGOBP1-2, SlitOBP1-5, SlitOBP7-9, SlitOBP14-15, SlitOBP17 and SlitOBP21*) were uniquely or primarily expressed in the male and female antennae; three OBPs *SlitOBP19*, *SlitOBP20* and *SlitOBP33* were only detected in the abdomen; the remaining 18 OBPs (*SlitOBP6*, *SlitOBP10-13*, *SlitOBP16*, *SlitOBP18*, *SlitOBP22-32*) were expressed not only in the antennae but also in other tissues such as heads, thoraxes, abdomens, legs and wings (Fig. 8). Equal amount cDNA (200 ng) were used in the RT-PCR reactions, the intensity of the PCR bands in antennae of some SlitOBPs was very weak or undetectable, such as *SlitOBP9*, *SlitOBP10*, *SlitOBP19-20*, *SlitOBP22-33*, the reason for this may be the relatively low expression levels of these *SlitOBPs* in the antennae, which consistent with the results of the low abundance of these *SlitOBPs* (RKKM value < 20) (Table 2).

**Figure 9:**
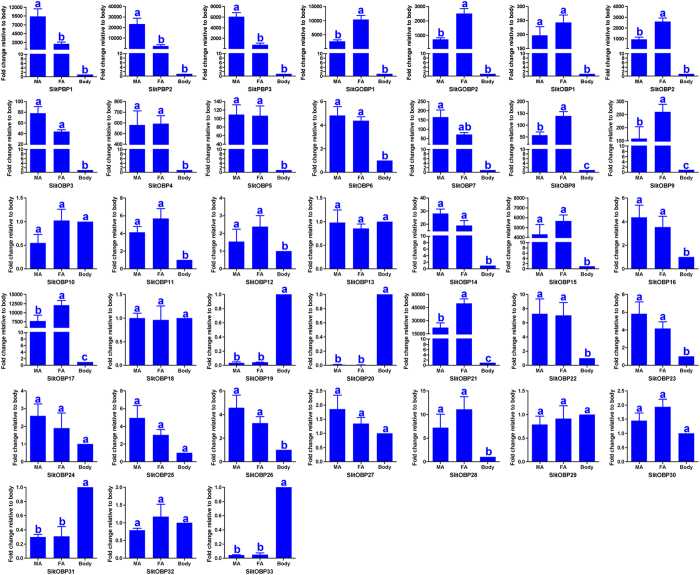
*S. litura* OBP transcript levels in different tissues as measured by RT-qPCR. MA: male antennae; FA: female antennae. The internal controls *β-actin* and *ribosomal protein L31* were used to normalize transcript levels in each sample. This figure was presented using *β-actin* as the reference gene to normalize the target gene expression and to correct sample-to-sample variation; similar results were obtained with *ribosomal protein L31* as the reference gene. The standard error is represented by the error bar, and the different letters (a, b, c) above each bar denote significant differences (p < 0.05).

In order to confirm the RT-PCR results, real-time quantitative PCR (RT-qPCR) analyses were performed to measure quantitatively expression levels of the 38 OBP genes in the male antennae, female antennae and body parts (mixture of heads, thoraxes, abdomens, legs, wings) (Fig. 9). The results confirmed the transcript expression level of 17 of the 38 SlitOBP genes (*SlitPBP1-3, SlitGOBP1-2, SlitOBP1-5, SlitOBP7-9, SlitOBP14-15, SlitOBP17* and *SlitOBP21*) was approximately 30 to 50000 times higher in both the male and female antennae than in the body parts. Furthermore, the three antennae-specific *PBPs* (*SlitPBP1-3*) showed an expression level of 6.8, 7.9 and 6.4 times higher in the male antennae than in the female antennae, respectively (*p* < 0.01), and the expression level of 7 antennae-specific OBPs (*SlitGOBP1-2*, *SlitOBP2*, *SlitOBP8*, *SlitOBP9*, *SlitOBP17*, *SlitOBP21*) was 4.0, 3.4, 2.8, 2.4, 1.6, 1.6 and 2.3 times higher in the female antennae than in the male antennae (*p* < 0.01). Seven OBPs (*SlitOBP1*, *SlitOBP3-7*, *SlitOBP15*) were found to be mainly expressed in the antennae and showed a similar expression level between the sexes (*p* > 0.01). Three OBPs (*SlitOBP19*, *SlitOBP20* and *SlitOBP33*) were detected mainly expressed in the body, with the expression levels 23 to 200 times higher than in the antennae. The remaining 18 OBPs (*SlitOBP6*, *SlitOBP10-13*, *SlitOBP16*, *SlitOBP18*, *SlitOBP22-32*) showed similar expression levels in the male antennae, female antennae and body parts (*p* > 0.01).

## Discussion

Insects mainly rely on various hair-like sensilla located on the antennae to detect the plant volatiles or sex pheromones from the environment^1^. Hundreds of published papers have reported functional studies of insect OBPs in chemoreception since their discovery in 1981. The exact functions of insect OBPs are still unknown, but their most important function is suggested to capture and deliver outside odorants to the ORs^4^,^6^. Insect OBPs, which are specifically or mainly expressed in the antennae, are prosed to play this olfaction function^4^,^6^. In this research, we identified 38 OBP genes from the *S. litura* antennal transcriptome, of which 13 *SlitOBPs* were identified from the 454 sequencing and 25 *SlitOBPs* were identified from Hiseq2000 sequencing. The three *SlitPBPs* and two *SlitGOBPs* have been reported in previous studies^38^,^39^, but the remaining 33 *SlitOBPs* are reported here for the first time. The number of *S. litura* OBPs identified in this study was comparable with the numbers identified from the antennal transcriptome of *A. ipsilon* (33)^14^, *S. littoralis* (36)^40^, and more than those identified in *H. armigera* (26)^36^, *M. sexta* (18)^34^ and in the beet armyworm *S. exigua*^41^ (11). The possible reasons for the small number of OBPs identified in 454 data than in Hiseq2000 data may be the poorer sequencing depth of the 454 sequencing (317 Mb raw data) than that of the Hiseq 2000 sequencing platform (5.6 G raw data). Actually, 19 of the 25 new identified OBPs showed very low abundance in the antennae with the RPKM value less than 100, this well explain why these low-expression OBP transcripts can be sequenced by Hiseq2000 sequencing but 454 sequencing.

Seven OBPs (*SlitPBP1-3, SlitGOBP1-2, SlitOBP3* and *SlitOBP5*) are highly abundant in the male and female antennal transcriptomes and are antennae-specific(RPKM value > 1000) (Table 2). As we know, insect OBPs exist in the sensillum lymph at an extremely high concentration (up to 10 mM), and this concentration requires the consumption of large amounts of energy^7^,^15^,^42^. Therefore, these highly abundant and antennae-specific OBPs must play some important physiological functions especially in the detecting mates and searching for host plants in *S. litura*.

Insect PBPs are thought to play a function in detecting sex pheromones, and insect GOBPs are proposed to detect the general plant volatiles^4^,^6^,^7^. However, some studies showed GOBPs have high binding affinity to sex pheromones^39^. The motif pattern analysis showed GOBPs have five same motifs as PBPs, supporting GOBP binding to sex pheromones (Fig. 5). The only difference between PBPs and GOBPs is the presence of Motif 3 in GOBPs at N-terminal region. Motif 4, 5 and 8 were only present in PBP1/2/3 and GOBP1/2 in the genus *Spodoptera*, this finding suggests these three conserved motifs may be important in sex pheromone binding thus in insect olfaction. On other hand, the motif 3 only existed in GOBP1/2 in the genus *Spodoptera*, and this conserved domain may play an essential role in the function differentiation between the PBP family and GOBP family in the genus *Spodoptera*.

The phylogenetic analysis of 193 OBPs from 6 different Lepidoptera species demonstrated that after a long history evolution, the Lepidoptera OBPs have differentiated into several different groups (Fig. 7), which is consistent with previous report^40^. Some OBP genes from the genus *Spodoptera* (*S. litura*, *S. littoralis* and *S. exigua*) have showed a very high protein identity and are located in the same branch with a very high bootstrap value support. For example, the GOBP1, GOBP2, PBP1, PBP2 and PBP3 sequences from *S. litura*, *S. littoralis* and *S. exigua* showed values as high as 97%, 89%, 95%, 96% and 95% identity, respectively. This finding indicated that these OBP genes from the three closely related insect species might have one same ancestor gene and have differentiated along sex isolation and speciation. *S. litura* and *S. littoralis* both had (Z,E)-9,11-tetradecadienyl acetate (Z9,E11-14:Ac) and (Z,E)-9,12-tetradecadienyl acetate (Z9,E12-14:Ac) as their two main female sex compounds^43^,^44^, and *S. exigua* had (Z,E)-9,12-tetradecadienyl acetate (Z9,E12-14:Ac) and (Z)-9-tetradecenl-ol (Z9-14:OH) as its main sex pheromones^45^. The minor sequence difference among the three PBPs in the three species may cause different sensitivity and specificity of the three PBPs to these highly structure-related sex pheromones of *S. litura*, *S. littoralis* and *S. exigua*, and finally help the male moths correctly discriminate the conspecific females.

The functional study of antennae-specific or antennae-enriched OBPs can help us to better understand the molecular and cellular mechanisms of insect olfaction and design an odorant-based insect control strategy. In this study, the RT-PCR and RT-qPCR results indicated that 17 of the 38 identified *S. litura* OBPs are antennae-specific or enriched, suggesting their putative role in the odorant detection. Indeed, several studies have reported their putative physiological roles in detecting the sex pheromones of *S. litura* and general plant volatiles. SlitPBP1, SlitPBP2 and SlitPBP3 all can bind the female sex pheromones with different binding abilities (PBP1 > PBP2 ≫ PBP3), but they cannot discriminate particular sex pheromone components^38^, all three *SlitPBPs* are male antennae-biased, with the expression levels 6.8, 7.9 and 6.4 times higher in the male antennae than in the female antennae, respectively (*p* < 0.01), Similar results were also found in the PBPs of the diamondback moth, *Plutella xyllotella*^46^ and in the black cutworm moth *A. ipsilon*^42^, the RPKM value analysis showed PBP1 are most abundance among the three SlitPBPs in male antennae (PBP1_RPKM_: PBP2_PRKM_: PBP3_RPKM_ = 2.3:0.94:1), suggesting PBP1 may play a major role in the sex pheromone detection, similar results were also obtained in other two papers^47^,^48^. The two sibling species *S. litura* and *S. littoralis* both use Z9,E11-14:Ac and Z9,E12-14:Ac as their main sex pheromone components but in a very different ratios, 20:1 and 9:1 in the *S. litura* and *S. littoralis,* respectively^43^,^44^. The three homologous PBPs in the sibling species *S. littoralis* were also identified^40^, but the comparative analysis of their expression in male antennae was not reported, but we can suspect that the qualitative differences of the sex pheromone components and the three PBPs expressed in male antennae are important factors that permit correct mate recognition and sexual isolation in the two sibling species. This hypothesis was supported by a recently research, inverted expression ratio of PBP1 and PBP2 was found in two different geographical species of the stem borer *Sesamia nonagrioides* (Lepidoptera:Noctuidae), and this inverted expression ratio of PBP1 and PBP2 are correlated with the inverted ratio of their two sex pheromone components Z11-16:OH and Z11-16:Ald^49^. In addition, the three PBPs in *S. littoralis* were also expressed in the sensilla of the larvae antennae and suspected to be involved in foraging activity^50^. The three PBPs of *S. litura* may be also expressed in the larvae antennae and play a similar function, but this assumption should be confirmed in the future.

The antennae-specific and female antennae-biased expression of two GOBPs of *S. litura* (Figs 8 and 9) is consistent with their binding to the sex pheromones and general plant volatiles with different binding affinities^39^,^51^. There is no functional study on other antennal OBPs (*SlitOBP1-5, SlitOBP7-9, SlitOBP14-15, SlitOBP17* and *SlitOBP21*), but from their high expression levels in the antennae, we can speculate that they also have a putative role in detecting of the female sex pheromones and general plant volatiles. Three body-specific expressed OBPs (*SlitOBP19*, *SlitOBP20* and *SlitOBP33*) were reported for the first time for the genus *Spodoptera*, and their functions remain to be solved as well as 18 OBPs (*SlitOBP6*, *SlitOBP10-13*, *SlitOBP16*, *SlitOBP18*, *SlitOBP22-32*) which have similar expression levels in the antennae and body parts. Our study provides a starting point to facilitate functional studies of these OBP genes at the molecular level both *in vivo* and *in vitro*.

## Methods

### Insect rearing and antennae collection

The larvae of *S. litura* were cultivated in the laboratory on an artificial diet^52^ at 24 °C with 75% relative humidity and a light-dark regime of 16L:8D. Pupae were sexed and males and females were kept separately in glass tubes. Emerged Adults were fed a 20% honey solution. Antennae (approximately 400 of each sex) were excised from 3-day-old male and female individuals and were immediately frozen and stored in liquid nitrogen until the RNA extraction.

### RNA extraction, cDNA library construction and 454 sequencing

Total RNA was extracted from male and female antennae using a TRIzol reagent (Life Technologies, Carlsbad, CA, USA). The concentration of RNA samples was determined with a NanoDrop ND-1000 spectrophotometer (Thermo Scientific, Wilmington, DE, USA), and the RNA integrity value (RIN) was checked by Bioanalyzer 2100 (Agilent Technologies, Palo Alto, CA, USA). Messenger RNAs were further isolated from the total RNA using a PolyATtract mRNA Isolation System III (Promega, Madison, WI, USA). The mRNAs were then sheared into approximately 800 nucleotides via a RNA Fragmentation Solution (Autolab, Beijing, China) at 70 °C for 30 sec, and then were cleaned and condensed using a RNeasy MinElute Cleanup Kit (Qiagen, Valencia, CA, USA). The first-strand cDNA were synthesized using N6 random primers and MMLV reverse transcriptase (TaKaRa, Dalian, China). Then, the second strand cDNA were synthesized using secondary strand cDNA synthesis enzyme mixtures (Autolab, Beijing, China). The cDNA with desired length were purified using a QIAquick PCR Purification Kit (Qiagen, Valencia, CA, USA) and eluted with 10 μl elution buffer. After blunting and appending with a poly-A tail at the 3′ end according to Roche’s Rapid Library Preparing protocols (Roche, USA), the purified cDNA were linked to GS-FLX Sequencing Adaptors (Roche, USA). Pyrosequencing of the cDNA library was performed using a 454 GS-FLX sequencer (Roche, Indianapolis, IN, USA) according to the manufacturer’s instructions.

### Bioinformatics analysis

Base calling of the raw 454 reads in the SFF files was performed using the python script sff_extract.py developed by COMAV (http://bioinf.comav.upv.es). All of the raw reads were then processed to remove low quality and adaptor sequences using the programs TagDust^53^, LUCY^54^ and SeqClean^55^ with default parameters. The resulting sequences were then screened against the NCBI UniVec database (http://www.ncbi.nlm.nih.gov/VecScreen/UniVec.html) to remove possible vector sequence contamination. The clean reads shorter than 60 bases were discarded based on the assumption that these reads might represent sequencing artifacts^56^.

Two steps were performed to assemble the clean reads. First, the sequence assembler MIRA3^57^ was used with the assembly settings of a minimum sequence overlap of 30 bp and a minimum percentage overlap identity of 80%. Then, CAP3 was used with the assembly parameters of an overlap length cutoff >30 and an overlap percent identity cutoff >90%^58^. The resulting contigs and singletons that were more than 100 bases were retained as unigenes and annotated as described below.

### Homology searches and functional classification

Following the assembly, homology searches of all unigenes were performed using the BLASTx and BLASTn programs against the GenBank non-redundant protein (nr) and nucleotide sequence (nt) databases at the NCBI. Matches with an E-value less than 1.0E-5 were considered to be significant^33^. Gene names were assigned to each unigene based on the best BLASTx hit with the highest score value.

Gene ontology terms were assigned by the tool Blast2GO^59^ through the BLASTx program with an E-value less than 1.0E-5. Then, the WEGO^60^ software was used to assign each GO ID to the related ontology entries. The longest open reading frame (ORF) of each unigene was determined using an ORF finder tool (http://www.ncbi.nlm.nih.gov/gorf/gorf.html).

### Additional *S. litura* transcriptome data and sequence assembly

We have also downloaded the recently released Hiseq2000 transcriptome data of *S. litura* antennae in the Short Read Archive (SRA) database at NCBI(Release date: 05/23/2015; Accession number : SRR1770355, SRR2032109 and SRR2032110). The adapter sequences and low quality reads were discarded and the clean reads were assembled into unigenes using Trinity^61^.

### Identification of putative *S. litura* OBP genes

The tBLASTn program was performed, with available sequences of OBP proteins from Lepidoptera species as a “query” to identify candidate unigenes that encode putative OBPs in the *S. litura*. All candidate OBPs were manually checked by the BLASTx program at the NCBI.

### Verification of the OBP sequences by cloning and sequencing

Gene-specific primers were designed and used to clone the ORF or partial sequences of each OBP gene (Supplementary Table 3). Template cDNA was synthesized using the GoScript Reverse Transcription System (Promega, Madison, USA). PCR reactions were carried out with 200 ng antennal cDNA with 0.5 units of *Ex Taq* DNA Polymerase (TaKaRa, Dalian, China). The following cycling conditions for the reactions were used: initial denaturation at 95 °C for 3 min; followed by 35 cycles of 94 °C for 45 sec, 56 °C for 1 min, 72 °C for 1 min, and a final extension at 72 °C for 10 min. The PCR products were gel-purified and subcloned into the PCR4-TOPO vector (Invitrogen, Carlsbad, CA, USA), and the insert was sequenced using an ABI3730XL automated sequencer (Applied Biosystems) with standard M13 primers.

### Transcript abundance of *S. litura* OBPs in the antennal transcriptome

To compare the differential expression of OBP genes in the *S. litura* male and female antennal transcriptomes, the read number for each OBP gene between the male and female antennae was converted to RPKM (Reads Per Kilobase per Million mapped reads)^62^, using the following formula: RPKM (A) = (1,000,000 × C × 1,000)/(N × L), where RPKM (A) is the expression of the OBP gene A, C is the number of reads that are uniquely aligned to OBP gene A, N is the total number of reads that are uniquely aligned to all unigenes, and L is the number of bases in the OBP gene A. The RPKM method eliminates the influence of gene length and sequencing depth on the calculation of gene expression. Thus, the calculated gene expression can be used to directly compare gene expression between samples.

### Motif analysis

A total of 78 of OBPs from *S. litura*, *S. littoralis* and *S. exigua* (Supplementary Table 4) were used for motif discovery and pattern analysis in the genus *Spodoptera*. A total of 384 OBPs and 225 CSPs from 36 different Lepidoptera species (Supplementary Table 5) were used for comparing the motif-pattern between Lepidoptera OBPs and CSPs. All the OBP and CSP sequences used in this study have intact ORFs and the translated proteins have similar length with insect OBPs and CSPs. The MEME^63^ (version 4.9.1) on the line server (http://meme.nbcr.net/meme/), which has been widely used for the discovery of DNA and protein motifs, was used to discover and analysis the motifs in this analysis. The parameters used for motif discovery were as follows: minimum width = 6, maximum width = 10, and the maximum number of motifs to find = 8.

### Sequence and phylogenetic analysis

The putative N-terminal signal peptides and the most likely cleavage site were predicted using the SignalP V4.0 program^64^ (http://www.cbs.dtu.dk/services/SignalP/). Sequence alignments were performed using the program ClustalX 2.1^65^ with default gap penalty parameters of gap opening 10 and extension 0.2, and were edited using the GeneDoc 2.7.0 software. The percent identity matrix of each pair OBPs is calculated using Vector NTI 11.5. A neighbor-joining tree^66^ was constructed using the program MEGA 6.0^67^ with a p-distance model and a pairwise deletion of gaps. The bootstrap support of tree branches was assessed by re-sampling amino acid positions 1000 times.

### Tissue expression analysis of *S. litura* OBPs

Before transcription, total RNA was treated with RQ1 RNase-Free DNase (Promega, Madison, USA) to remove residual genomic DNA. cDNA from male antennae, female antennae, heads, thoraxes, abdomens, legs, wings and the body parts (mixture of heads, thoraxes, abdomens, legs, wings) were synthesized using a GoScript Reverse Transcription System (Promega, Madison, USA). An equal amount of cDNA (200 ng) was used as the RT-PCR and RT-qPCR templates. Specific primer pairs for the RT-PCR were designed with the program Primer3web (version 4.0.0) (http://bioinfo.ut.ee/primer3/) (Supplementary Table 6). The *β-actin* (GenBank Acc. KP331524) of *S. litura* was used as the control gene to test the integrity of the cDNA. The PCR was performed under following conditions: 95 °C for 2 min, followed by 35 cycles of 95 °C for 30 sec, 56 °C for 30 sec, 72 °C for 1 min, and a final extension for 10 min at 72 °C. PCR products were analyzed on 1.2% agarose gel and visualized after staining with ethidium bromide. In additional, the PCR products were selected and verified by DNA sequencing. To reach reproducibility, each sample was examined at least six times with two biological samples.

RT-qPCR analysis was conducted using an ABI 7500 Real-Time PCR System (Applied Biosystems, Carlsbad, CA). The primers used for the RT-qPCR were designed using the program Beacon Designer 7.90 (PREMIER Biosoft International) (Supplementary Table 7). Two reference genes, *β-actin* (GenBank Acc. KP331524) and *ribosomal protein L31* (GenBank Acc. KP331525) were used for normalizing the target gene expression and for correcting for sample-to-sample variation. Each RT-qPCR reaction was conducted in a 25 μl reaction mixture containing 12.5 μl of SuperReal PreMix Plus (TianGen, Beijing, China), 0.75 μl of each primer (10 μM), 0.5 μl of Rox Reference Dye, 1 μl of sample cDNA, and 9.5 μl of sterilized H_2_O. The RT-qPCR cycling parameters were as follows: 95 °C for 15 min, followed by 40 cycles of 95 °C for 10 sec and 60 °C for 32 sec. Then, the PCR products were heated to 95 °C for 15 sec, cooled to 60 °C for 1 min, heated to 95 °C for 30 sec and cooled to 60 °C for 15 sec to measure the dissociation curves. Negative controls without template were included in each experiment. To check reproducibility, each RT-qPCR reaction for each sample was performed in three technical replicates and two biological replicates. The comparative 2^−ΔΔCT^ method^68^ was used to calculate the relative quantification between tissues. The comparative analyses of each target gene among various tissues were determined using a one-way nested analysis of variance (ANOVA), followed by Tukey’s honest significance difference (HSD) test using the SPSS Statistics 18.0 software (SPSS Inc., Chicago, IL, USA). When applicable, the values were presented as the mean ± SE.

## Additional Information

**How to cite this article**: Gu, S.-H. *et al.* Identification and comparative expression analysis of odorant binding protein genes in the tobacco cutworm *Spodoptera litura*. *Sci. Rep.* **5**, 13800; doi: 10.1038/srep13800 (2015).

## Supplementary Material

Supplementary Information

## Figures and Tables

**Table 1:** An overview of the sequencing and assembly process.

	Male	Female	Total
Raw reads	178345	253266	431611
Raw read mean length	516 bp	514 bp	515 bp
Clean reads	177227	251805	429032
Clean read mean length	494 bp	495 bp	494.5 bp
Singletons	735	1095	1830
Contigs	15743	17905	19393
Unigenes	16478	19000	21223
Unigene mean length	864 bp	808 bp	766 bp

**Table 2:** List of OBP genes in *S. litura* antennal transcriptome.

Gene name	ORF(bp)	Signal peptide (AA)	Accession number	RPKM value		BLASTx annotation	Score	*E*-value	% Identify
				Male	Female				
SlitPBP1	492	1-23	KP331511	4810	1368	gb|AAY21255.1| pheromone binding protein 1 [Spodoptera litura]	341	2e-117	100%
SlitPBP2	510	1-27	KP331512	1978	1211	gb|AAZ22339.1| pheromone binding protein 2 [Spodoptera litura]	314	8e-107	100%
SlitPBP3	492	1-22	KP331513	2110	1194	gb|ACY78414.1| pheromone binding protein 3 [Spodoptera litura]	300	1e-101	96%
SlitGOBP1	492	1-19	KP331514	1368	1975	gb|ABM54823.1|general odorant-binding protein GOBP1 [Spodoptera litura]	301	8e-102	100%
SlitGOBP2	486	1-21	KP331515	1031	1808	gb|ABM54824.1|general odorant-binding protein GOBP2 [Spodoptera litura]	323	1e-110	96%
SlitOBP1	5′ missing	ND	KP331516	38	48	gb|AGH70107.1| odorant binding protein 11 [Spodoptera exigua]	300	3e-101	95%
SlitOBP2	3′ missing	1-23	KP331517	5	8	gb|AGP03456.1| odorant binding protein 10 [Spodoptera exigua]	214	6e-69	89%
SlitOBP3	411	1-19	KP331518	1080	1225	emb|CAA05508.1|antennal binding protein X [Heliothis virescens]	208	5e-66	92%
SlitOBP4	471	1-20	KP331519	93	129	gb|ADY17882.1|odorant binding protein [Spodoptera exigua]	313	1e-106	96%
SlitOBP5	441	1-21	KP331520	4016	2297	gb|ADY17881.1| antennal binding protein [Spodoptera exigua]	270	3e-90	90%
SlitOBP6	441	1-21	KP331521	119	132	gb|AAR28762.1|odorant-binding protein [Spodoptera frugiperda]	243	3e-79	93%
SlitOBP7	426	1-21	KP331522	104	48	gb|AGH70103.1| odorant binding protein 7 [Spodoptera exigua]	283	2e-95	97%
SlitOBP8	417	1-18	KP331523	40	67	gb|AEB54589.1| odorant binding protein 8 [Helicoverpa armigera]	248	7e-82	84%
SlitOBP9	1017	1-20	KT192030	5	4	ref|XP_011552170.1| odorant-binding protein 71-like [Plutella xylostella]	211	6e-62	62%
SlitOBP10	552	1-20	KT192031	3	5	gb|AII00978.1| odorant binding protein [Dendrolimus houi]	334	7e-114	92%
SlitOBP11	387	1-17	KT192032	185	234	gb|ADY17884.1|odorant binding protein [Spodoptera exigua]	191	2e-59	81%
SlitOBP12	399	1-16	KT192033	347	405	gb|AGH70105.1|odorant binding protein 9 [Spodoptera exigua]	260	2e-86	95%
SlitOBP13	639	1-18	KT192034	77	68	gb|AGC92793.1|odorant-binding protein 19 [Helicoverpa assulta]	191	5e-57	54%
SlitOBP14	3′ missing	1-21	KT192035	34	45	gb|AGP03454.1| odorant-binding protein 8 [Spodoptera exigua]	48.5	6e-05	51%
SlitOBP15	435	1-24	KT192036	289	262	gb|AGP03458.1| odorant-binding protein 12 [Spodoptera exigua]	275	4e-92	92%
SlitOBP16	501	1-16	KT192037	117	215	gb|AII00985.1|odorant binding protein [Dendrolimus houi]	57.4	2e-07	32%
SlitOBP17	429	1-22	KT192038	83	111	gb|AFG72998.1|odorant-binding protein 1 [Cnaphalocrocis medinalis]	232	3e-75	83%
SlitOBP18	579	1-17	KT192039	66	78	gb|AGR39564.1|odorant binding protein 1, partial [Agrotis ipsilon]	231	3e-73	58%
SlitOBP19	627	1-19	KT192040	8	11	gb|EHJ64212.1|odorant-binding protein 2 [Danaus plexippus]	246	3e-78	61%
SlitOBP20	405	1-18	KT192041	3	2	ref|XP_012061112.1|general odorant-binding protein 69a-like [Atta cephalotes]	38.9	0.36	30%
SlitOBP21	447	1-26	KT192042	376	412	gb|AGH70104.1|odorant binding protein 8 [Spodoptera exigua]	280	1e-93	98%
SlitOBP22	450	1-22	KT192043	2	2	gb|AEX07271.1|odorant-binding protein [Helicoverpa assulta]	188	8e-58	59%
SlitOBP23	435	1-24	KT192044	3	2	gb|AGP03459.1| odorant binding protein 13 [Spodoptera exigua]	248	3e-81	84%
SlitOBP24	—	ND	KT192045	1	1	gb|AEX07271.1|odorant-binding protein [Helicoverpa assulta]	114	7e-30	73%
SlitOBP25	462	1-21	KT192046	3	4	gb|AGP03454.1| odorant binding protein 8 [Spodoptera exigua]	176	9e-53	60%
SlitOBP26	—	ND	KT192047	1	1	gb|AGP03457.1| odorant binding protein 11 [Spodoptera exigua]	169	4e-51	95%
SlitOBP27	441	1-17	KT192048	4	3	gb|AGR39569.1|odorant binding protein 6, partial [Agrotis ipsilon]	199	2e-62	76%
SlitOBP28	444	1-19	KT192049	1	1	gb|AGP03460.1| odorant binding protein 14 [Spodoptera exigua]	254	1e-83	93%
SlitOBP29	468	1-17	KT192050	1	1	gb|ADY17886.1|odorant binding protein [Spodoptera exigua]	281	2e-94	90%
SlitOBP30	—	ND	KT192051	1	2	gb|AFM77984.1|odorant binding protein 6 [Spodoptera exigua]	99.8	1e-24	73%
SlitOBP31	—	ND	KT192052	2	1	gb|EHJ67147.1|odorant-binding protein 2 [Danaus plexippus]	159	3e-46	74%
SlitOBP32	—	ND	KT192053	1	1	gb|AEB54584.1| odorant binding protein 4 [Helicoverpa armigera]	39.3	0.11	32%
SlitOBP33	468	1-18	KT192054	7	8	gb|AGK24578.1|odorant-binding protein 2 [Chilo suppressalis]	81.3	4e-16	32%

“—” represent that gene is partial and has not intact ORF. ND means not detected.
