# Supplementary material for: Identification and comparative expression analysis of odorant binding protein genes in the tobacco cutworm Spodoptera litura
Source: Sci Rep. 2015 Sep 8;5:13800. doi: 10.1038/srep13800 (PMC4561897; doi:10.1038/srep13800)
Supplement: Supplementary Information [file srep13800-s1.pdf]

Supplementary Information for

Identification and comparative expression analysis of odorant binding protein genes in the tobacco cutworm *Spodoptera litura*

Shao-Hua Gu[1], Jing-Jiang Zhou[2], Shang Gao[1,3], Da-Hai Wang[5], Xian-Chun Li[1,4], Yu-Yuan Guo[1], Yong-Jun Zhang[1,]*

[1]State Key Laboratory for Biology of Plant Diseases and Insect Pests, Institute of Plant Protection, Chinese Academy of Agricultural Sciences, Beijing, China.

[2]Department of Biological Chemistry and Crop Protection, Rothamsted Research, Harpenden, UK.

[3]Education Ministry Key Laboratory of Integrated Management of Crop Diseases and Pests, College of Plant Protection, Nanjing Agricultural University, Nanjing, China.

[4]Department of Entomology and BIO5 Institute, University of Arizona, Tucson, AZ 85721 USA

[5]Beijing Autolab Biotechnology Company, Beijing, China.

*Corresponding author

Yong-Jun Zhang, PhD. State Key Laboratory for Biology of Plant Diseases and Insect Pests, Institute of Plant Protection, Chinese Academy of Agricultural Sciences, Beijing, 100193, China.

Email: yjzhang@ippcaas.cn; Tel.:+86 10 62815929; Fax: +86 10 62894786.

Supplementary Figures

Supplementary Figure 1. The full-length gels for each SlitOBPs presented in Figure 8.

Supplementary Tables

Supplementary Table 1.

A percent identity matrix of the 30 full-length SlitOBPs. The calculations are based on the alignment of amino acid sequence by Vector NTI 10.2. The percentage of identity of each pair is shown.

Supplementary Table 2.

A percent identity matrix of the 78 OBPs in *S. litura*, *S. littoralis* and *S. exigua*. The calculations are based on the alignment of amino acid sequence by Vector NTI 10.2. The percentage of identity of each pair is shown.

Supplementary Table 3.

Gene specific primers used for molecular cloning of *S. litura* OBP genes.

Supplementary Table 4.

The protein names and sequences of the 78 of OBPs from *S. litura*, *S. littoralis* and *S. exigua* used in Figure 5.

Supplementary Table 5.

The protein names and sequences of the 384 OBPs and 225 CSPs used in Figure 6.

Supplementary Table 6.

Primers used in RT-PCR for determination expression level of *S. litura* OBP genes.

Supplementary Table 7.

Primers used in real-time PCR for determination expression level of *S. litura* OBP genes.

Supplementary Table 8.

The protein names and sequences of the 193 Lepidoptera OBPs used in Figure 7.

Supplementary Figure 1.

SlitPBP1

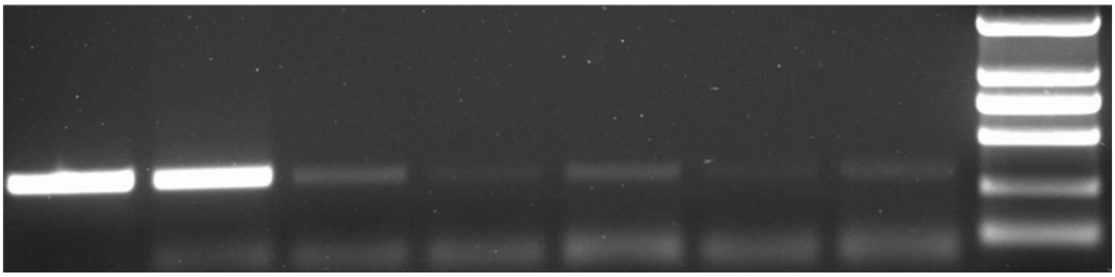

SlitPBP2

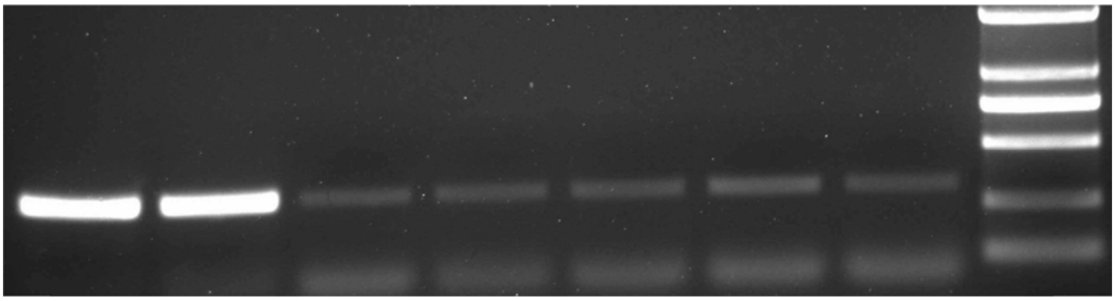

SlitPBP3

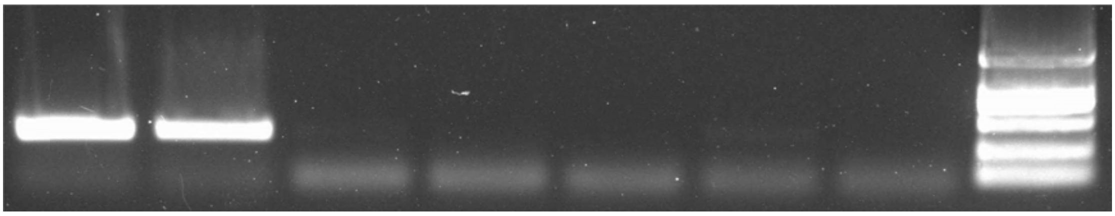

SlitGOBP1

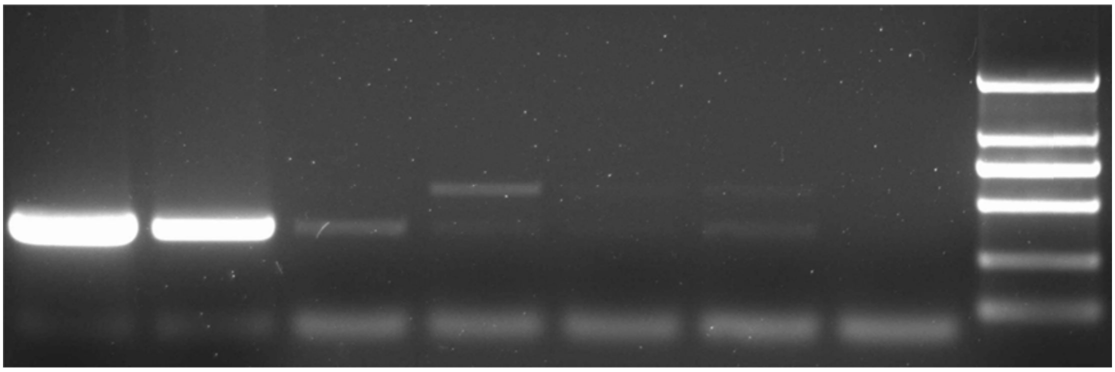

SlitGOBP2

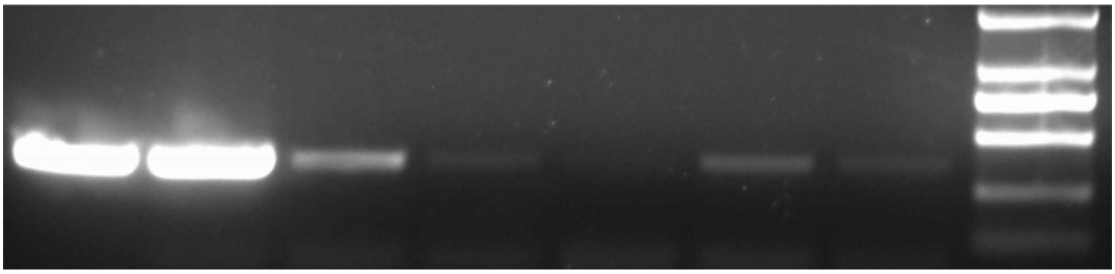

SlitOBP1

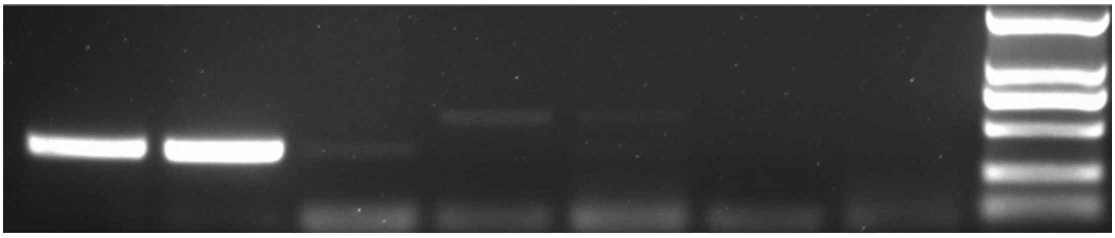

SlitOBP2

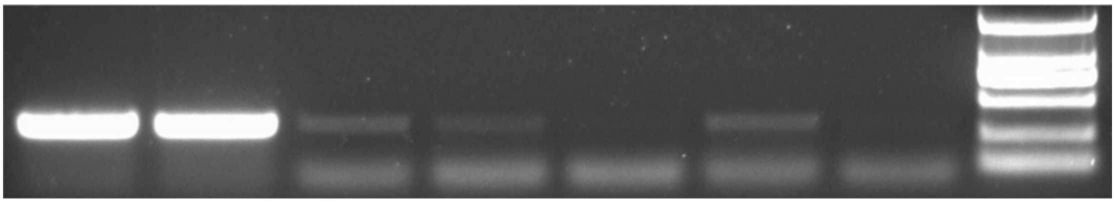

SlitOBP3

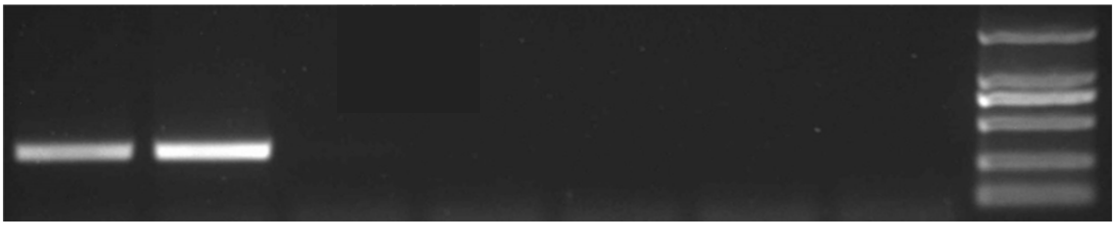

SlitOBP4

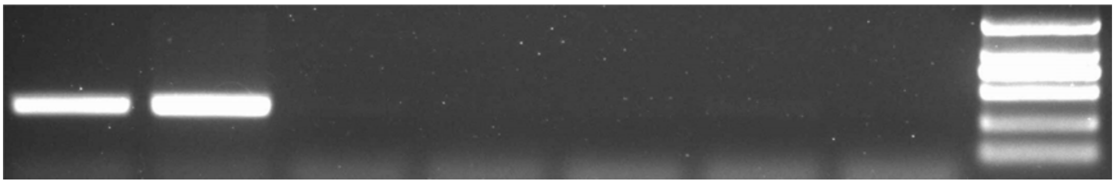

SlitOBP5

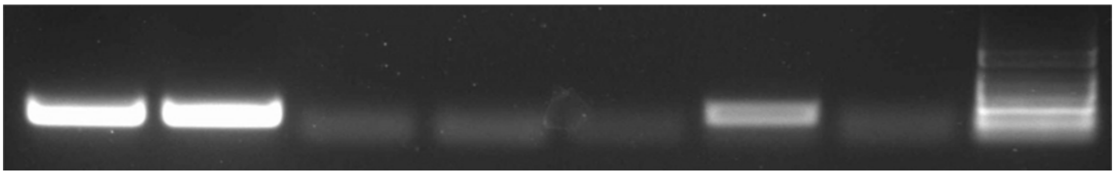

SlitOBP6

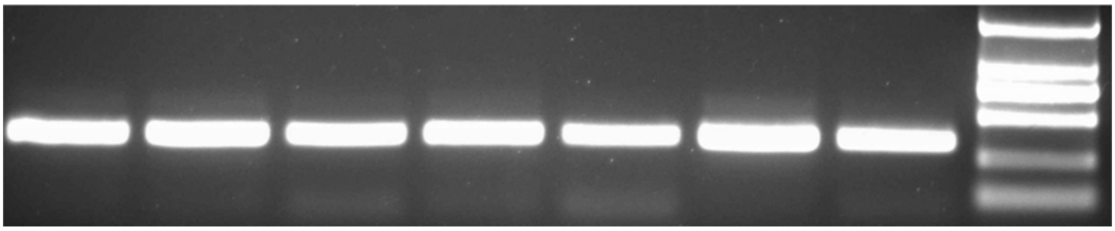

SlitOBP7

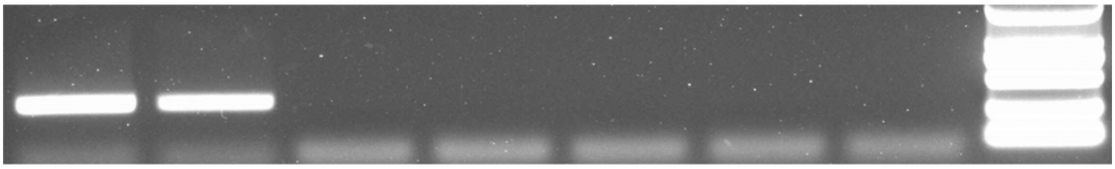

SlitOBP8

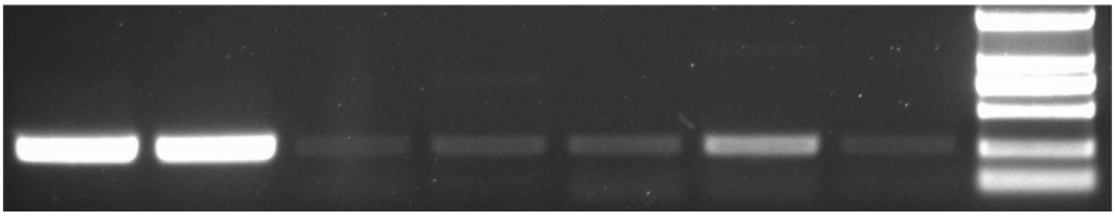

SlitOBP9

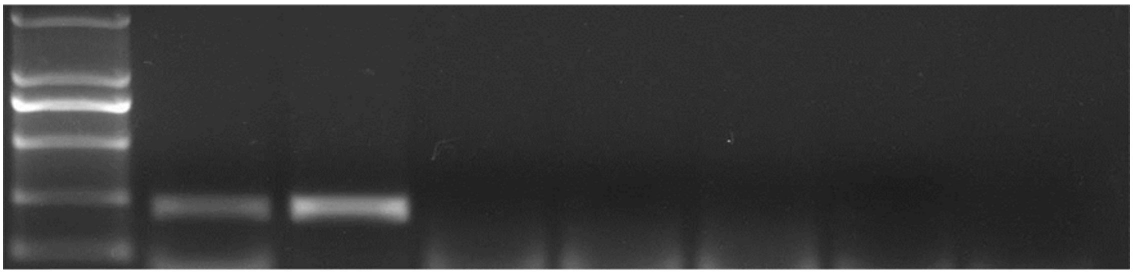

SlitOBP10

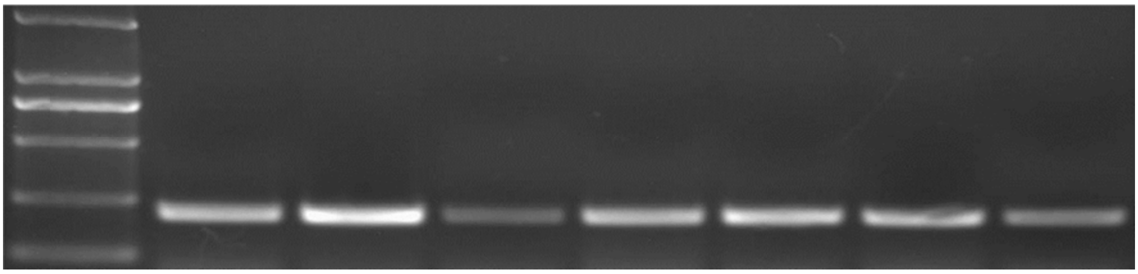

SlitOBP11

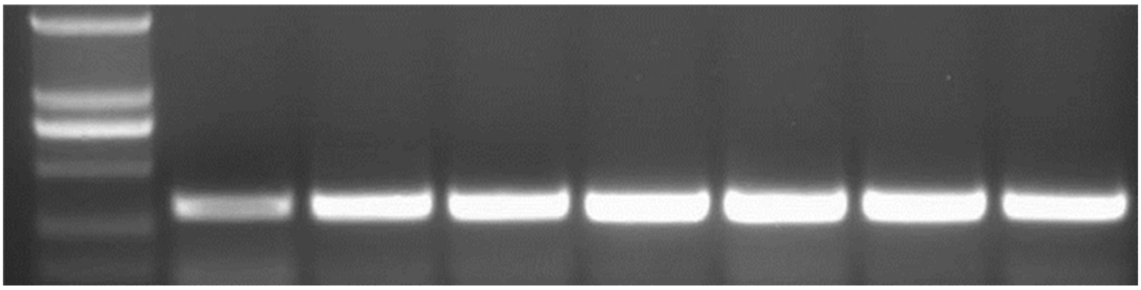

SlitOBP12

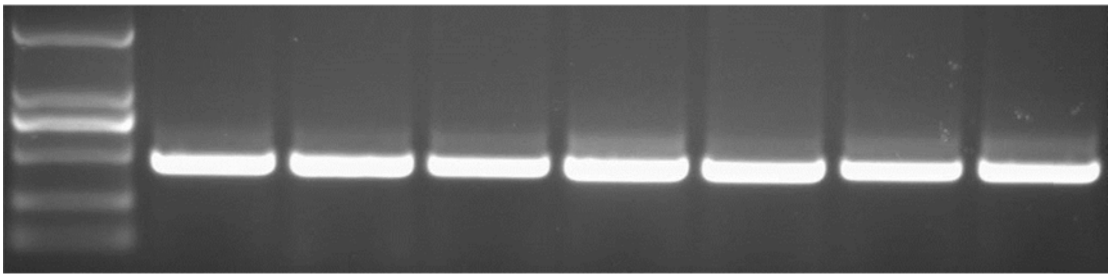

SlitOBP13

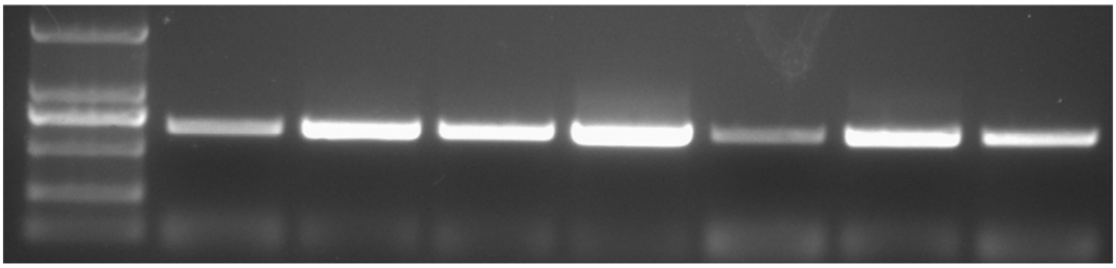

SlitOBP14

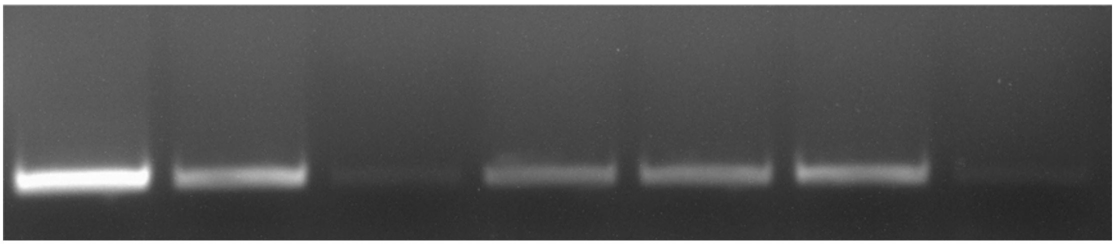

SlitOBP15

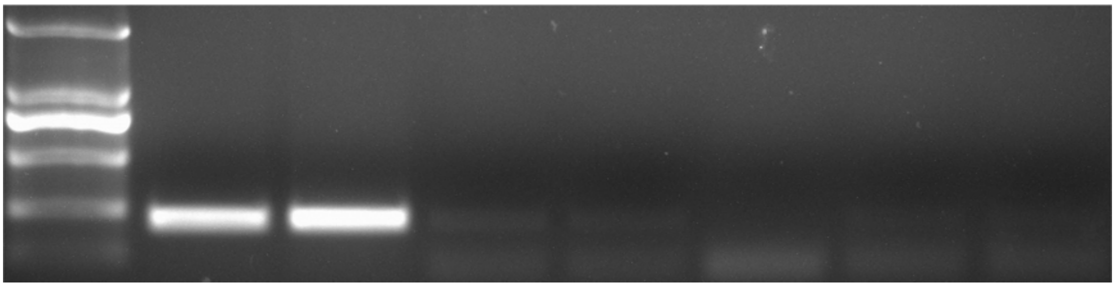

SlitOBP16

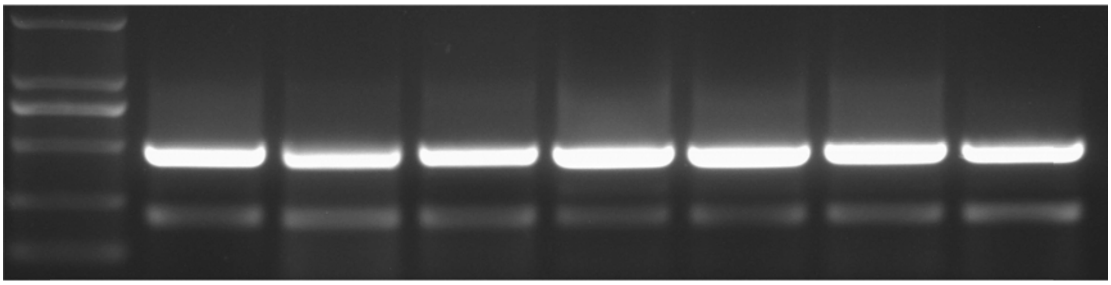

SlitOBP17

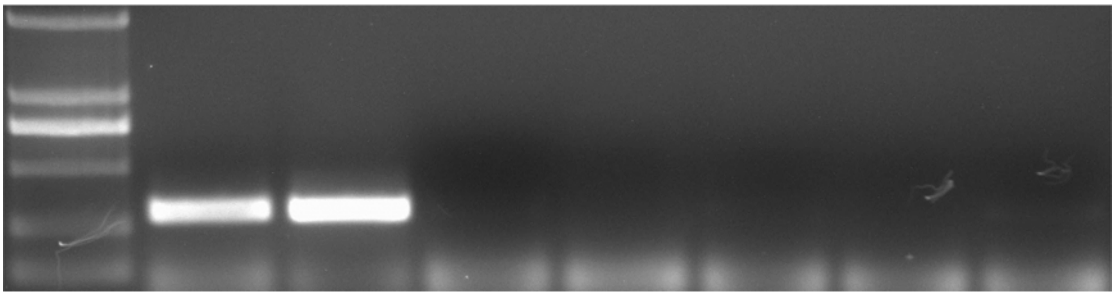

SlitOBP18

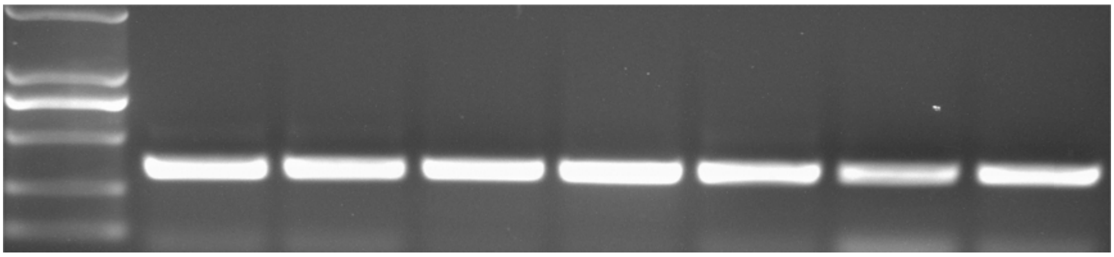

SlitOBP19

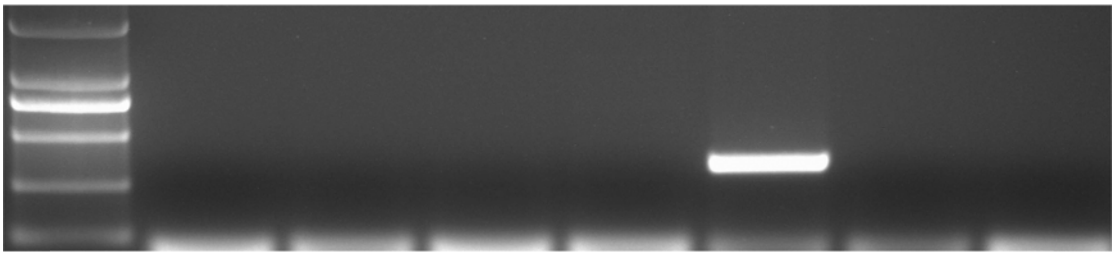

SlitOBP20

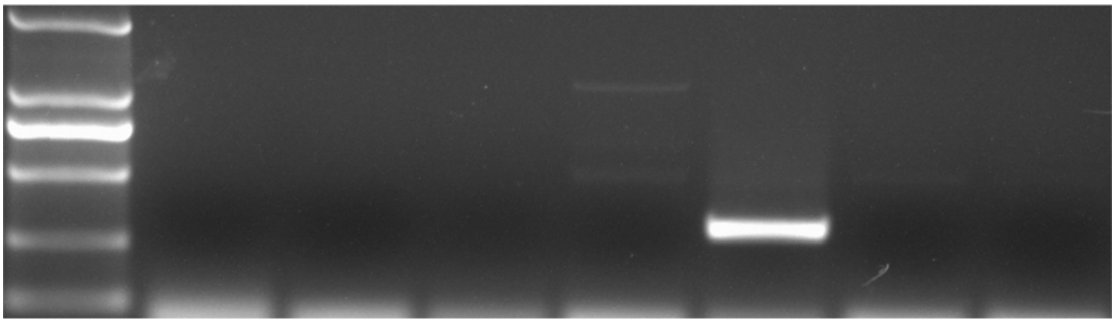

SlitOBP21

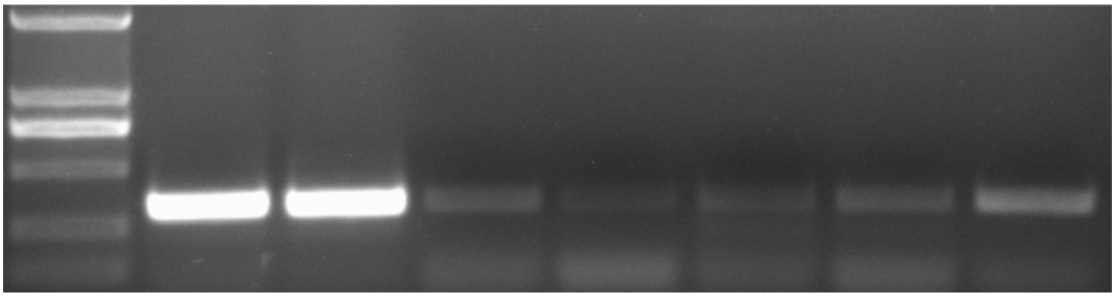

SlitOBP22

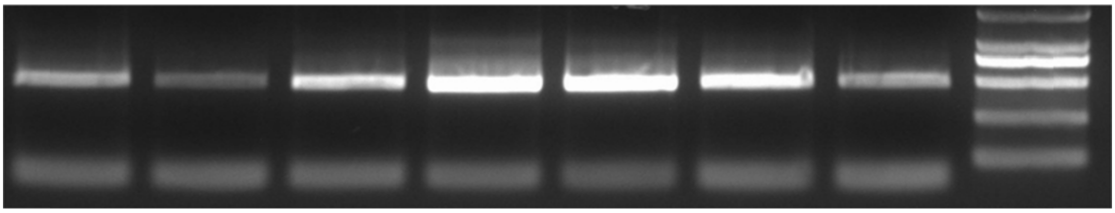

SlitOBP23

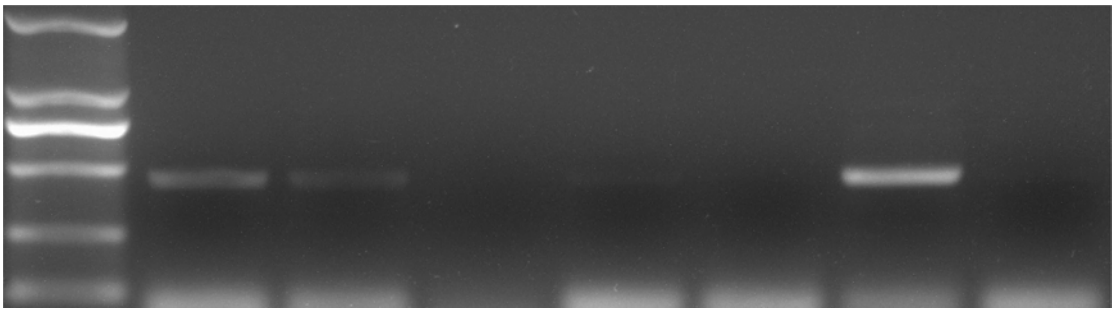

SlitOBP24

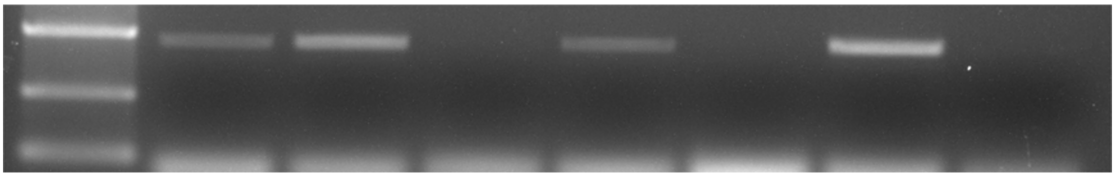

SlitOBP25

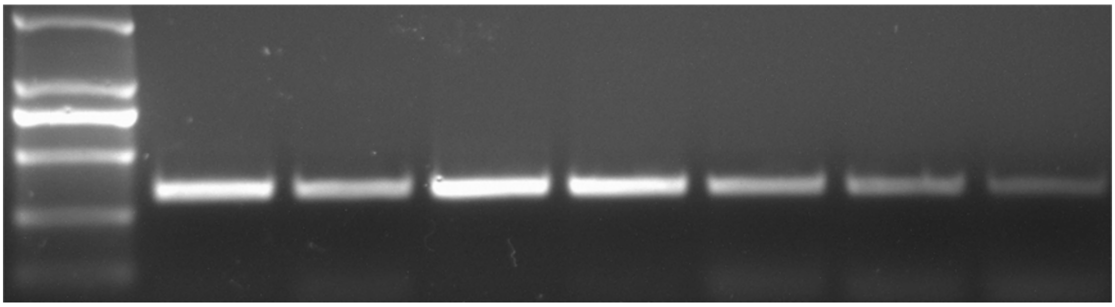

SlitOBP26

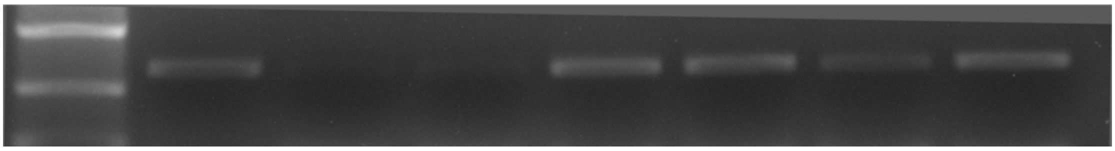

SlitOBP27

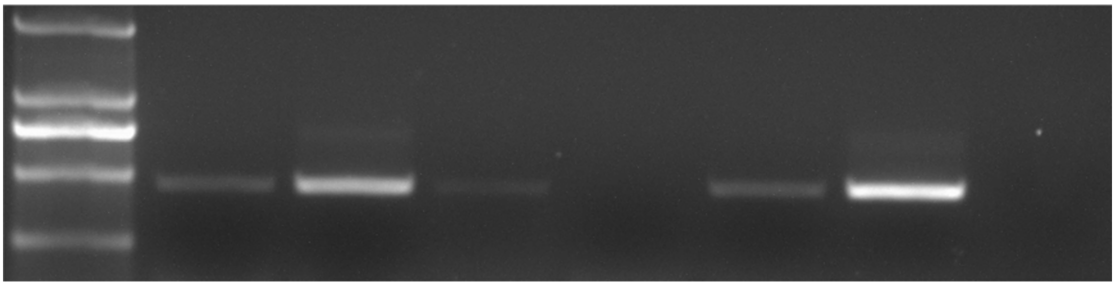

SlitOBP28

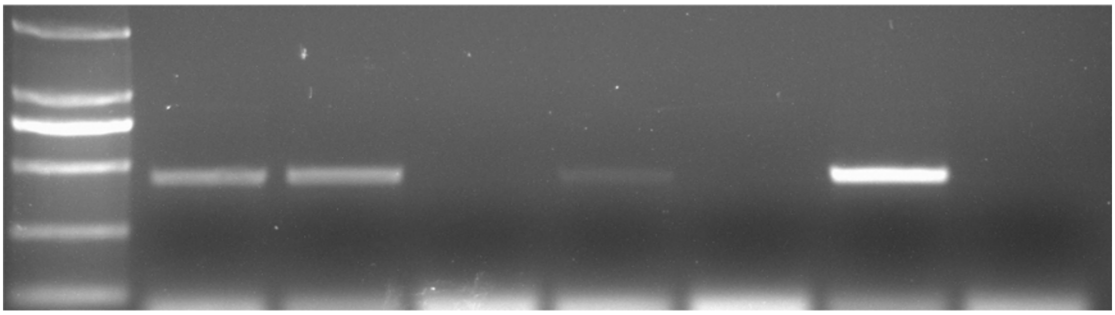

SlitOBP29

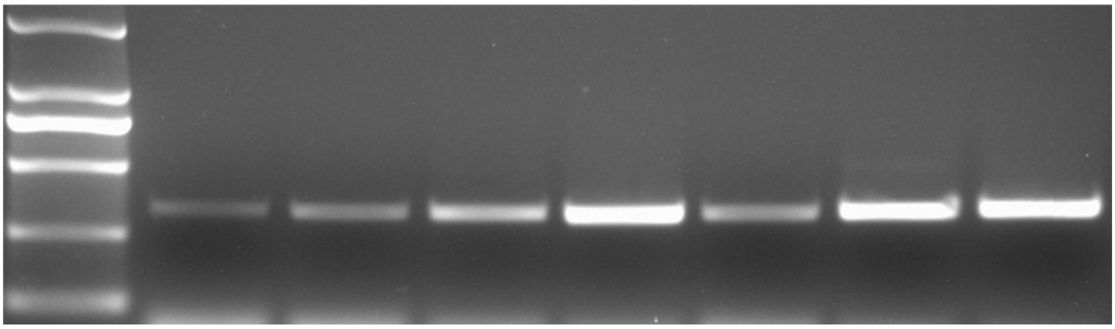

SlitOBP30

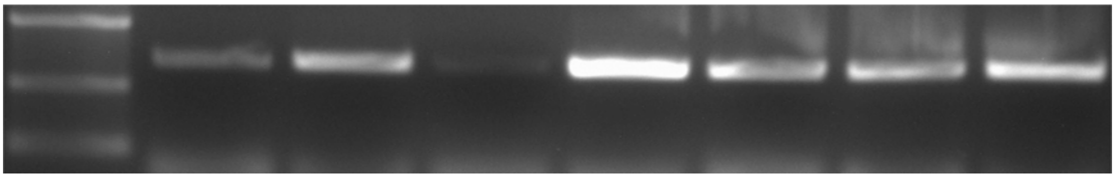

SlitOBP31

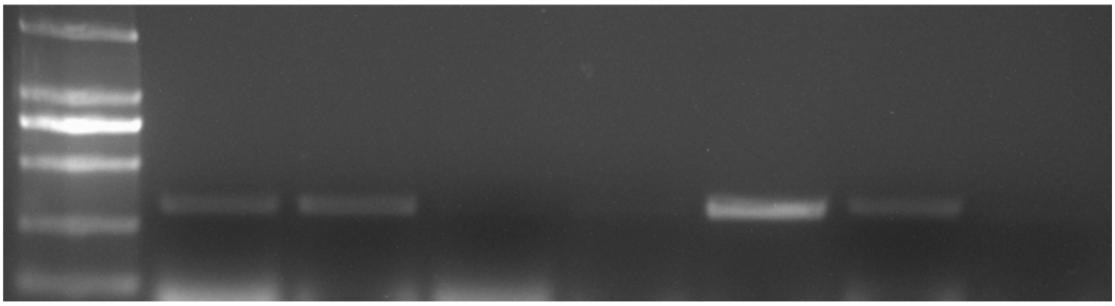

SlitOBP32

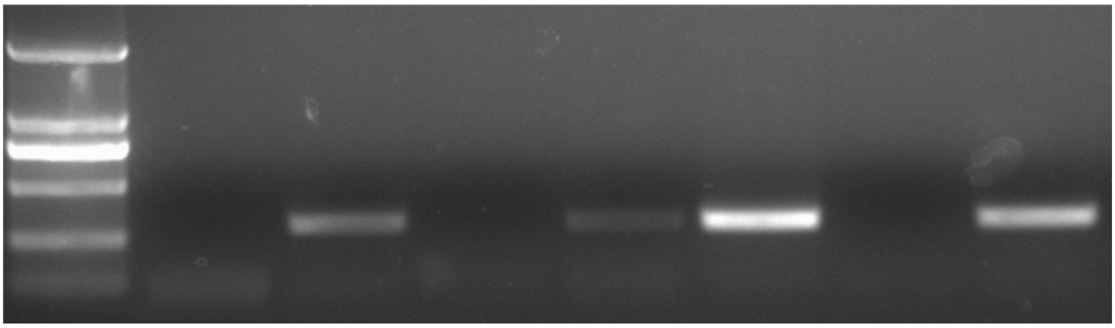

SlitOBP33

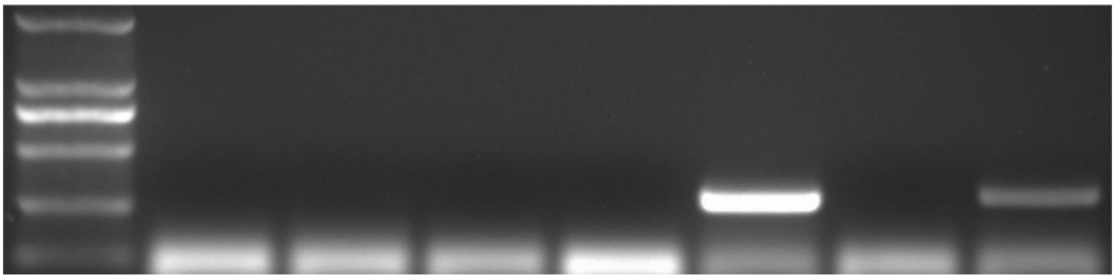

β-actin

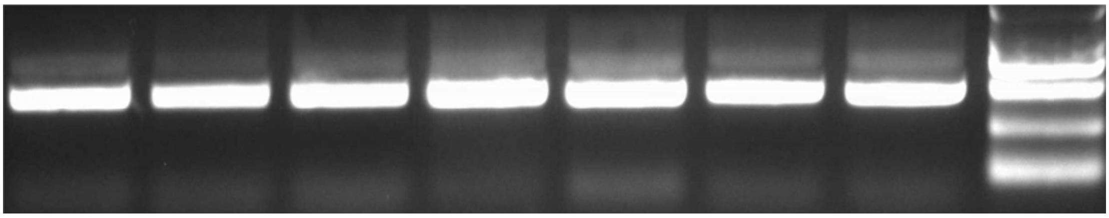

Supplementary Table 1.

	SlitGOBP1	SlitGOBP2	SlitPBP1	SlitPBP3	SlitPBP2	SlitOBP10	SlitOBP22	SlitOBP6	SlitOBP23	SlitOBP25	SlitOBP5	SlitOBP28	SlitOBP29	SlitOBP11	SlitOBP12	SlitOBP33	SlitOBP15	SlitOBP17	SlitOBP7	SlitOBP8	SlitOBP21	SlitOBP3	SlitOBP13	SlitOBP18	SlitOBP27	SlitOBP16	SlitOBP9	SlitOBP19	SlitOBP20	SlitOBP4
SlitGOBP1		50	30	30	30	12	12	11	14	10	12	18	11	8	15	11	13	14	15	18	15	18	9	9	10	10	11	12	13	11
SlitGOBP2			32	30	29	12	19	15	18	19	16	18	10	13	14	11	12	13	13	16	15	14	12	8	8	11	11	13	12	12
SlitPBP1				48	43	14	17	16	21	15	18	16	11	14	16	14	13	10	11	11	15	15	12	8	5	10	11	14	13	10
SlitPBP3					46	14	18	16	16	16	17	17	12	19	14	12	13	11	10	12	14	11	13	6	8	11	12	15	16	12
SlitPBP2						10	18	18	18	15	14	16	13	16	17	16	13	15	13	12	15	11	10	6	10	11	15	13	17	14
SlitOBP10							20	16	16	15	17	12	14	15	11	12	13	15	12	14	12	11	12	13	8	8	14	12	10	13
SlitOBP22								59	51	32	35	28	25	19	17	14	12	10	13	15	14	17	11	7	9	15	16	14	15	14
SlitOBP6									47	33	35	26	26	23	18	14	13	12	16	14	14	15	14	11	10	18	21	16	15	14
SlitOBP23										34	31	26	21	24	13	18	15	15	16	13	12	18	10	9	10	15	18	18	16	13
SlitOBP25											28	23	19	20	12	14	12	11	12	10	11	10	12	12	10	14	13	14	11	17
SlitOBP5												28	23	18	19	14	11	11	10	14	15	13	11	11	12	12	19	15	14	14
SlitOBP28													31	20	20	15	15	16	21	17	17	13	12	6	16	12	18	15	14	16
SlitOBP29														17	19	12	15	14	18	15	17	12	8	8	13	13	15	12	13	13
SlitOBP11															32	21	20	15	16	13	16	20	11	14	12	20	14	16	16	16
SlitOBP12																28	16	17	13	15	23	22	12	14	15	14	13	14	13	11
SlitOBP33																	12	13	14	17	20	15	10	13	17	13	11	16	14	14
SlitOBP15																		53	42	38	20	12	9	6	11	14	17	14	16	12
SlitOBP17																			54	36	18	11	10	10	12	12	18	14	12	9
SlitOBP7																				42	24	17	9	9	14	7	16	11	12	12
SlitOBP8																					22	17	12	5	13	11	13	14	15	11
SlitOBP21																						24	10	8	15	15	15	12	12	14
SlitOBP3																							10	9	11	11	9	12	16	20
SlitOBP13																								17	15	14	8	7	12	9
SlitOBP18																									16	12	9	7	8	12
SlitOBP27																										18	10	10	16	10
SlitOBP16																											20	11	10	10
SlitOBP9																												13	9	12
SlitOBP19																													21	14
SlitOBP20																														15
SlitOBP4																														

Supplementary Table 2.

	SexiABP1	SlitOBP12	SlittoOBP12	SlitOBP5	SexiOBP1	SlitOBP6	SlittoOBP11	SlitOBP22	SlittoOBP24	SlitOBP23	SlittoOBP23	SexiOBP3	SlittoOBP25	SlitOBP25	SexiOBP4	SlitOBP29	SexiOBP6	SlitOBP28	SlittoOBP28	SlittoOBP26	SexiGOBP1	SlitGOBP1	SlittoGOBP1	SexiGOBP2	SlitGOBP2	SlittoGOBP2
SexiABP1		91	91	90	34	36	37	37	31	30	30	27	29	27	23	22	24	25	25	22	16	14	14	16	15	15
SlitOBP12			100	97	32	35	36	35	32	31	30	26	28	26	23	22	24	27	28	23	16	14	15	15	15	14
SlittoOBP12				97	32	35	36	35	32	31	30	26	28	26	23	22	24	27	28	23	16	14	15	15	15	14
SlitOBP5					32	35	36	35	31	31	30	26	27	27	23	23	25	29	29	22	15	14	14	14	15	14
SexiOBP1						89	88	56	53	48	44	40	42	31	27	25	24	27	26	28	13	13	12	19	20	19
SlitOBP6							98	58	56	48	44	40	41	32	27	25	23	28	27	29	13	12	11	16	19	17
SlittoOBP11								58	58	48	45	39	41	32	27	26	24	27	26	29	12	11	11	16	18	17
SlitOBP22									39	51	49	39	36	31	26	25	27	29	29	32	11	13	12	17	19	17
SlittoOBP24										38	37	39	37	28	23	23	20	28	27	22	10	9	9	18	17	17
SlitOBP23											92	36	34	35	22	21	29	28	27	26	14	14	14	16	18	16
SlittoOBP23												34	34	33	23	22	26	26	26	25	14	15	14	14	18	15
SexiOBP3													70	29	23	22	20	26	25	23	14	14	14	21	22	22
SlittoOBP25														31	20	19	18	24	24	25	14	12	12	20	19	19
SlitOBP25															22	20	24	24	24	22	12	10	10	19	21	20
SexiOBP4																96	34	33	33	29	11	11	10	13	12	12
SlitOBP29																	33	32	31	27	11	11	10	13	12	12
SexiOBP6																		31	30	24	11	14	15	11	12	11
SlitOBP28																			97	32	14	17	16	20	20	19
SlittoOBP28																				32	14	16	15	20	20	19
SlittoOBP26																					12	11	11	16	16	15
SexiGOBP1																						91	93	52	49	51
SlitGOBP1																							96	53	50	50
SlittoGOBP1																								52	50	51
SexiGOBP2																									96	96
SlitGOBP2																										98

	SexiPBP1	SlitPBP1	SlittoPBP1	SexiPBP3	SlitPBP3	SlittoPBP3	SexiPBP2	SlitPBP2	SlittoPBP2	SexiOBP10	SlitOBP7	SlittoOBP14	SlitOBP15	SlittoOBP15	SlitOBP17	SlittoOBP17	SlittoOBP22	SexiOBP5	SlitOBP8	SlittoOBP18	SexiOBP8	SlitOBP21	SlittoOBP7	SexiOBP2	SlitOBP11	SlittoOBP3	SexiOBP9
SexiABP1	18	20	20	18	18	18	16	17	16	14	14	14	11	12	13	13	16	16	16	16	14	15	15	21	18	17	12
SlitOBP12	17	19	20	17	17	18	15	15	15	14	14	13	12	13	14	14	15	16	17	16	15	16	16	22	18	17	13
SlittoOBP12	17	19	20	17	17	18	15	15	15	14	14	13	12	13	14	14	15	16	17	16	15	16	16	22	18	17	13
SlitOBP5	16	18	19	18	18	18	15	16	16	14	14	14	12	13	15	14	15	16	17	16	15	16	16	22	18	17	12
SexiOBP1	14	14	15	15	15	15	17	17	17	19	20	19	13	14	14	14	14	16	15	16	19	19	19	16	17	17	12
SlitOBP6	15	15	15	17	17	17	16	17	16	19	20	19	14	15	13	13	16	16	16	16	19	19	19	15	16	16	14
SlittoOBP11	14	14	15	17	17	17	16	17	16	19	20	19	14	16	13	13	16	16	16	16	19	19	19	15	16	16	13
SlitOBP22	13	15	15	19	18	18	16	17	16	17	18	16	12	13	11	11	13	16	14	14	18	18	18	16	14	14	12
SlittoOBP24	14	15	15	16	15	15	11	12	12	14	14	14	16	18	13	12	13	15	14	15	14	14	14	16	16	16	11
SlitOBP23	18	18	18	16	16	15	15	15	16	23	24	24	14	16	17	17	14	19	18	19	15	15	15	19	20	20	12
SlittoOBP23	16	17	17	14	16	16	16	16	16	21	22	22	15	17	16	16	12	21	18	19	15	15	15	20	19	19	10
SexiOBP3	14	15	15	20	18	18	15	16	16	17	17	17	14	16	14	14	11	14	12	12	14	14	14	17	17	17	16
SlittoOBP25	16	16	16	19	17	18	15	15	15	17	18	17	17	19	12	12	10	13	11	11	14	14	14	12	15	15	12
SlitOBP25	14	14	14	14	16	15	16	15	16	17	17	17	14	15	13	13	15	13	14	14	12	12	12	13	17	17	8
SexiOBP4	16	16	16	18	19	18	14	14	14	16	16	16	17	18	15	15	17	21	20	20	16	16	16	13	14	14	18
SlitOBP29	15	15	15	17	18	18	13	13	13	16	16	16	16	17	15	15	18	21	19	20	15	15	16	12	13	13	17
SexiOBP6	16	15	15	14	12	12	16	17	16	19	19	19	14	16	14	14	17	20	20	21	17	17	17	13	11	10	17
SlitOBP28	13	14	14	20	19	19	14	14	14	21	21	21	18	19	19	18	20	21	18	18	18	18	18	11	12	12	13
SlittoOBP28	12	15	15	19	19	19	16	15	15	21	21	21	18	18	19	18	19	20	17	18	17	17	17	12	12	12	13
SlittoOBP26	16	15	16	18	17	19	15	16	15	13	14	13	13	12	15	15	12	13	14	15	17	17	17	9	12	12	12
SexiGOBP1	30	29	30	32	31	31	30	29	29	12	13	12	14	14	13	13	14	16	16	16	15	15	15	9	8	8	11
SlitGOBP1	31	30	31	31	30	30	30	30	30	13	14	13	12	12	14	14	15	16	16	16	17	17	17	8	7	7	11
SlittoGOBP1	30	30	30	32	31	31	29	29	29	13	14	13	12	13	14	14	17	16	16	16	17	17	17	8	7	7	11
SexiGOBP2	32	31	31	35	33	33	30	30	30	12	13	13	14	14	11	11	16	13	14	14	17	17	17	14	16	16	11
SlitGOBP2	32	32	32	34	30	31	29	29	29	13	14	14	13	13	11	11	17	12	12	12	18	18	18	13	15	15	11
SlittoGOBP2	33	33	33	34	31	31	29	29	29	13	14	14	13	13	10	10	15	12	12	12	17	17	17	13	16	16	10
SexiPBP1		86	86	48	49	50	41	41	42	11	11	10	15	14	11	11	14	15	14	12	17	17	17	12	13	13	17
SlitPBP1			98	48	48	49	43	43	44	9	9	10	14	13	11	10	14	15	14	14	18	18	19	13	13	13	15
SlittoPBP1				48	48	49	44	43	44	9	9	10	14	13	11	10	14	15	14	14	18	18	19	13	13	13	15
SexiPBP3					87	86	44	43	44	11	11	11	13	14	11	11	12	18	16	17	17	17	17	12	14	14	13
SlitPBP3						98	46	46	46	11	11	11	13	15	9	9	12	18	17	18	15	15	15	11	12	12	11
SlittoPBP3							46	46	46	11	12	12	13	14	10	10	12	18	18	18	15	15	15	11	12	12	12
SexiPBP2								94	97	13	13	14	11	12	13	13	13	14	15	14	16	16	16	10	12	12	13
SlitPBP2									98	13	13	14	11	12	13	13	14	14	15	14	17	17	18	10	12	12	14
SlittoPBP2										14	14	14	11	12	14	13	14	14	15	15	17	17	17	10	12	12	13
SexiOBP10											97	96	43	47	52	53	47	46	42	41	21	21	21	12	14	14	15
SlitOBP7												98	43	47	51	52	46	47	42	42	22	22	22	12	14	14	14
SlittoOBP14													42	46	52	53	46	47	42	42	20	20	20	12	14	14	14
SlitOBP15														98	53	54	36	40	39	37	19	19	19	14	18	18	14
SlittoOBP15															58	58	38	41	42	42	19	19	19	15	19	19	13
SlitOBP17																98	44	40	37	37	19	19	19	12	15	14	16
SlittoOBP17																	44	40	37	37	19	19	19	13	16	15	16
SlittoOBP22																		39	35	35	26	26	26	14	12	12	15
SexiOBP5																			92	92	25	25	25	13	12	12	13
SlitOBP8																				98	24	24	24	13	12	11	14
SlittoOBP18																					22	22	23	14	13	12	15
SexiOBP8																						98	98	14	12	12	20
SlitOBP21																							100	14	12	12	20
SlittoOBP7																								14	12	12	20
SexiOBP2																									84	84	27
SlitOBP11																										99	29
SlittoOBP3																											28

	SlittoOBP4	SlitOBP3	SlittoOBP20	SexiOBP11	SlitOBP1	SlittoOBP10	SlitOBP9	SlittoOBP9	SlitOBP10	SlittoOBP2	SlittoOBP19	SlittoOBP8	SexiOBP7	SlitOBP4	SlittoOBP5	SlitOBP33	SlitOBP13	SlittoOBP13	SlitOBP18	SlittoOBP1	SlitOBP16	SlittoOBP16	SlitOBP27	SlitOBP19	SlitOBP20
SexiABP1	12	15	16	10	10	10	12	11	12	12	10	10	13	14	14	11	10	10	7	7	12	13	10	15	15
SlitOBP12	13	15	16	10	10	10	11	11	13	13	10	10	12	13	13	11	10	11	7	7	11	13	10	15	14
SlittoOBP12	13	15	16	10	10	10	11	11	13	13	10	10	12	13	13	11	10	11	7	7	11	13	10	15	14
SlitOBP5	12	15	16	11	11	11	11	11	12	12	10	10	12	13	13	12	10	10	8	8	12	13	11	15	13
SexiOBP1	12	15	15	10	9	10	10	11	11	11	13	12	14	14	14	14	11	9	10	10	11	12	11	15	13
SlitOBP6	14	14	14	9	8	9	11	12	12	12	12	13	13	13	13	14	12	10	11	11	12	12	11	15	13
SlittoOBP11	13	14	14	9	8	9	11	12	12	12	12	14	13	13	13	14	12	10	10	10	11	12	9	16	13
SlitOBP22	12	15	15	11	10	11	10	10	16	16	12	12	11	11	11	13	12	10	10	11	12	16	10	13	14
SlittoOBP24	11	14	15	8	8	8	10	9	8	8	10	10	13	13	13	10	11	10	7	7	10	10	9	18	14
SlitOBP23	12	17	17	13	12	12	10	10	12	12	11	14	13	13	13	16	9	8	10	10	10	13	9	16	15
SlittoOBP23	10	17	17	12	11	11	8	8	12	12	10	16	12	12	12	14	10	9	10	10	9	11	9	16	15
SexiOBP3	16	12	11	6	7	8	10	9	8	8	12	12	14	15	16	10	10	12	8	8	8	9	18	14	16
SlittoOBP25	12	10	10	8	8	10	11	11	10	10	11	10	15	15	15	9	8	9	7	7	8	10	9	14	15
SlitOBP25	8	9	10	10	10	10	7	6	9	9	10	10	16	16	16	14	10	10	9	9	9	8	13	11	13
SexiOBP4	18	14	16	6	6	7	9	7	9	9	8	10	12	12	12	8	10	10	7	7	8	8	12	14	12
SlitOBP29	17	14	15	8	8	9	10	9	9	9	7	9	12	12	12	8	9	9	7	8	7	8	13	15	11
SexiOBP6	17	15	14	10	10	10	9	8	14	14	12	10	12	12	12	15	7	7	8	8	12	12	13	17	14
SlitOBP28	14	10	10	10	10	10	14	12	8	8	16	9	16	15	15	13	11	10	12	12	12	11	14	20	14
SlittoOBP28	14	10	10	9	9	9	13	12	8	8	14	9	16	15	15	12	11	10	11	11	12	11	13	19	14
SlittoOBP26	11	14	13	10	10	10	9	8	9	9	8	6	15	14	14	12	9	9	9	9	8	9	12	15	11
SexiGOBP1	11	13	12	6	6	6	8	8	9	9	5	5	6	6	6	9	5	6	7	7	7	6	12	13	14
SlitGOBP1	11	13	13	8	8	8	8	8	9	9	5	5	6	6	6	9	7	6	8	8	8	7	13	12	13
SlittoGOBP1	11	14	13	8	8	8	8	8	9	9	6	6	6	6	6	10	7	8	8	8	8	7	13	12	14
SexiGOBP2	11	12	12	7	6	6	8	8	8	8	6	6	7	7	7	10	5	5	5	5	9	9	12	14	13
SlitGOBP2	11	14	13	8	8	8	6	6	8	8	9	7	8	8	8	11	7	7	8	8	10	9	13	13	12
SlittoGOBP2	10	12	13	6	6	6	7	6	8	8	7	6	7	7	7	9	6	6	5	5	9	9	12	13	12
SexiPBP1	17	16	15	11	11	11	8	8	4	4	11	9	6	8	8	11	6	6	6	6	9	6	11	12	14
SlitPBP1	16	12	12	11	11	11	10	11	6	6	11	11	5	6	6	10	6	7	6	6	13	9	11	12	14
SlittoPBP1	16	12	12	11	11	11	11	11	6	6	11	11	5	6	6	10	6	7	6	6	12	9	11	12	14
SexiPBP3	13	10	11	11	11	11	8	8	8	8	7	11	8	8	8	11	9	8	11	11	10	11	14	12	16
SlitPBP3	13	11	11	12	12	12	8	8	8	8	7	10	8	8	8	10	7	6	10	10	10	11	13	12	16
SlittoPBP3	14	12	11	12	12	12	9	9	8	8	7	12	10	10	10	10	7	6	11	11	10	11	13	13	14
SexiPBP2	14	12	12	9	9	9	10	10	6	6	10	9	10	10	10	10	6	6	8	8	10	12	15	10	16
SlitPBP2	14	12	13	8	8	8	10	10	6	6	9	9	10	11	11	11	6	6	9	9	10	12	16	11	16
SlittoPBP2	14	12	12	8	8	8	10	10	6	6	9	9	9	10	10	11	6	5	8	8	10	12	15	11	16
SexiOBP10	14	18	18	6	6	6	8	8	12	12	12	12	8	8	8	10	7	6	8	9	11	11	12	15	15
SlitOBP7	14	17	17	6	6	6	8	8	12	12	12	11	8	8	8	10	6	6	8	9	11	10	12	15	15
SlittoOBP14	14	18	18	6	6	6	8	8	12	12	12	13	8	9	9	10	7	6	8	8	11	11	12	15	15
SlitOBP15	14	12	12	10	9	9	10	10	11	11	8	10	9	9	8	8	4	3	7	7	11	10	9	16	16
SlittoOBP15	13	13	13	9	8	8	9	10	11	11	8	11	9	9	9	8	4	4	7	7	9	10	9	16	18
SlitOBP17	15	13	13	10	11	11	9	10	11	11	8	12	9	10	10	13	7	8	6	6	12	10	8	19	13
SlittoOBP17	15	13	13	10	11	11	10	10	11	11	8	12	9	10	10	13	8	8	6	6	11	10	8	20	12
SlittoOBP22	15	13	12	12	12	12	12	13	9	9	6	15	9	10	10	12	6	6	9	10	10	10	10	12	12
SexiOBP5	14	18	18	6	7	7	8	8	5	5	7	8	9	10	10	13	8	8	6	6	9	10	12	15	17
SlitOBP8	14	16	15	6	7	7	8	8	5	5	9	9	8	9	9	14	7	6	5	5	12	10	13	14	16
SlittoOBP18	16	16	15	5	6	6	7	8	7	7	8	10	9	10	10	14	6	5	5	5	12	10	12	15	15
SexiOBP8	20	21	21	9	10	10	7	8	7	7	8	10	11	11	11	11	5	6	10	10	16	12	13	16	12
SlitOBP21	20	21	21	9	10	10	8	9	7	7	8	9	11	11	11	11	5	6	10	10	16	12	12	15	12
SlittoOBP7	20	21	21	9	9	9	8	8	8	8	8	9	12	11	11	11	5	6	10	10	16	12	12	15	12
SexiOBP2	30	24	24	8	8	8	6	6	8	8	9	11	12	12	12	13	8	8	8	8	13	15	9	13	15
SlitOBP11	32	19	19	6	7	7	5	5	11	11	9	12	12	12	12	12	9	9	8	8	13	15	12	12	12
SlittoOBP3	31	19	19	6	7	7	5	5	12	12	9	12	12	12	12	12	9	9	8	8	13	15	12	12	12
SexiOBP9	94	24	23	6	5	5	6	6	8	8	8	13	5	5	5	14	10	8	10	10	11	11	13	7	11
SlittoOBP4		26	24	5	5	5	4	5	8	8	8	13	5	5	5	16	9	8	9	9	11	11	13	7	10
SlitOBP3			97	7	7	7	6	6	3	3	8	10	12	12	12	13	7	5	10	10	10	9	10	11	12
SlittoOBP20				7	7	7	6	6	4	4	8	9	12	12	12	12	7	6	10	10	10	9	8	10	12
SexiOBP11					95	94	22	22	15	15	6	5	10	10	10	8	6	6	8	8	5	6	11	9	10
SlitOBP1						98	22	22	13	13	5	5	10	9	9	8	6	6	8	8	5	6	11	9	9
SlittoOBP10							22	22	13	13	6	5	10	9	9	8	6	5	7	8	5	6	10	9	9
SlitOBP9								96	9	9	6	7	10	10	10	8	6	6	6	6	7	8	8	9	5
SlittoOBP9									11	11	6	7	10	10	10	8	7	6	6	6	6	7	8	9	5
SlitOBP10										100	7	8	10	10	10	10	6	6	5	6	8	8	6	7	8
SlittoOBP2											7	8	10	10	10	10	6	6	5	6	8	8	6	7	8
SlittoOBP19												9	12	12	12	11	5	4	5	5	6	4	7	8	8
SlittoOBP8													3	3	3	6	7	6	4	5	9	8	7	4	6
SexiOBP7														96	96	17	7	7	10	10	7	8	11	14	13
SlitOBP4															99	17	7	8	11	11	7	8	12	14	14
SlittoOBP5																17	7	8	11	11	7	8	12	14	14
SlitOBP33																	5	3	9	9	10	10	11	11	10
SlitOBP13																		87	23	22	13	14	9	10	8
SlittoOBP13																			21	21	13	14	8	10	8
SlitOBP18																				98	17	19	10	8	10
SlittoOBP1																					17	19	10	8	10
SlitOBP16																						98	10	5	7
SlittoOBP16																							10	3	8
SlitOBP27																								12	15
SlitOBP19																									23
SlitOBP20																									

Supplementary Table 3.

Gene	Forward primers (5'-3')	Reverse primers (5'-3')
PBP1	ATGGCGAACGCAAGATGG	TTACACGGCGGTCATGACCT
PBP2	ATGGCGTTCTGTCCATCGG	TTACACTTCAGCAAGCACCTCTC
PBP3	ATGGGATCGCGTAACGTCTTT	CTATATTTCCGTGAGGACCTCACTC
GOBP1	ATGTTGCTGTTGTTGCGCG	TCAGCGCGCCTCAGCCT
GOBP2	ATGACGTCGAAGTGTTGTTTGCT	TCAGTACTTCTCCATAACAGCTTCAAT
OBP1	ATGAAAGAAGGGAACCGATACAG	CTATCCTGTCTGTTCCGTAGAAGATT
OBP2	ATGACTCGAGCACAAGTTAAAAAGA	CTTTTCATACAAACATTTCGATGC
OBP3	ATGTGGATGCAGGCGCTAGT	CTATATGAGGAAGTAGTCCGCCTTG
OBP4	ATGACTAAAGTACTGTTTGCCATCG	TTAAGATTTCTCATATCTTTCAATTTCC
OBP5	ATGTCCGTTGTGCGGTGTTC	TCACTCCATCTTCTTCTTAGTCTCAG
OBP6	ATGTCCAAGTTCACTTGTTTGGTTT	TTAAGCGGAGATCTCGCTCTTGT
OBP7	ATGGATCAAAAAAGAATATGTTTATTTG	TTATGCGAAAATAAAATCTTCTGGC
OBP8	ATGCTTCTGACAAAAATTGTGAAGTT	TCAAGGAAAGTAAAATATACCAGGATT
OBP9	ATGTGCCTCGTCAAGTACCATG	CTAGAACGTCAGGTCATCCTTCC
OBP10	ATGGTTCGTAAAATCAGTGGACTC	TTAGAGGCCGGAGGTTCCAC
OBP11	ATGAAATCGTTTGTTGTATTCTGTATC	TTACGAAGCGAACATAATGTGTGAT
OBP12	ATGAAGACTCTGTTTGTTTTCGCT	TTACAAAAAAATCGCATGCTTG
OBP13	ATGATAACATCATGTTTATTAGTACTAAGTGC	TTAACGAACTTCAAAATCTTTTATTGTT
OBP14	ATGGTTAAAGTTACTTACATAGCCTTGT	GGGGCAAGTAAACTCGCATC
OBP15	ATGTATTCCATTAACTGTTTTATTTTTTC	GGGAACATGAAATTAGCTGGGT
OBP16	ATGTACCGCTTTGTGATCCTCA	TTATATGACTTTCGCACATTTTCC
OBP17	ATGAAAACCTTTCGACTACTCTGTTG	TCAAGGAAATACAAAATTTTGCG
OBP18	ATGTTTAAGCTCTGTGTGTTCCTTG	TTATACTGTACGTGGTTGTGTGTAGC
OBP19	ATGTTTCGCCGAACGCTG	TTAATAGTTAACCATTATTTTGTGTTTATTA
OBP20	ATGGAAAAAATCCTAATTTTTACTTTTATAA	TTACATTAGTAGAATTCTGTATAAATGTGTG
OBP21	ATGGCGCGGAGGCAGC	TCAGAATAAGAAGTATAAGTCTGGATCCT
OBP22	ATGTCGAAGTTTACCTGCATTATCC	TTAGAACGGCATCTCAACTTTGTG
OBP23	ATGGCCAAGTTCTCTTGTCTTGTT	TTAGATGATGATACTAGTCTTATGCTCCAA
OBP24	TGTGTTTATAAAAAGACAGAATTTTTG	TTTATGATCCAAAAAGCATTGGGTT
OBP25	ATGGCTAAAGTTACGTGCATAGTCC	TTAATAATCAACACCAAACATATTTGG
OBP26	TGCTTCTTGGCCTGCATGAT	TTAGATGTCGAACCCAAACTTAGAAG
OBP27	ATGTATAAATTTGTTATTTTGTGCTCTA	CTAGCACTTCCACGCTGTGTC
OBP28	ATGATCGTTCGGTTTTTATTATGTTT	TTATGGTAATTTTATTCCCAGCTGAG
OBP29	ATGTGGAATTTATTGGTTGTGTTTT	TTATAACTTGAATCCAAACTTGGGT
OBP30	GCTTTATCGATGGACGATCTAAAG	CCCTGGTCGTTCATCATTCC
OBP31	CGAAACGAACACCGCGAG	AGCTCTATTAGCTTCTAATAAATGTTC
OBP32	CGAATTGATAGGAATGATATACCTTGTA	CTATTTCCATTCATCGGGATCTCT
OBP33	ATGACTTGCTCCCAGGCGC	TTAGGAGTTGTTGTCAGTCTCGCT

Supplementary Table 4.

>SlitPBP1
MANARWRFVFVVYALYLTSAVLGSQDLMVKMTKGFTRVVDDCKTELNVGDHIMQDMYNYWREDYQLINRD
MGCMLLCMAKKLDLMDDQTMHHGKTEDFAKSHGADDDVAKKLVSVIHECEQQHAGIADDCMRVLEVAKCF
RTKIHELKWAPSIEVIMEEVMTAV
>SlitPBP2
MAFCPSVTMSLRVALVVAASLLVVVQASQDVMKNLAVNFAKPLDDCKKEMDLPDSVTTDFYNFWKEGYELTN
RQTGCAILCLSSKLEILDQELNLHHGRAQEFAMKHGADEAMAKQIVDMIHTCAQSTPDEAADPCMKALNVAK
CFKLKVHELNWAPSVELIVGEVLAEV
>SlitPBP3
MGSRNVFVALVVLTVGMREIEPSKDPMKYIASGFVKVLEECKHELNMNDHLIADLFHYWKLEYTLLNRDTGCAI
ICMGKKLDLLDASGRMHHGNAQEFAKKHGAGDEVASQIVQIIHDCEKKHERDDDECLRVLEVAKCFRTGIHEL
NWQPNVEVIVSEVLTEI
>SlitGOBP1
MLLLLRALPLLAAVLPLRADVNVMKDVTLGFGQALDKCRQESQLTEEKMEEFFHFWREDFKFEHRELGCAIQC
MSRHFNLLTDTSRMHHENTEQFIQSFPNGEVLARQMVELIHACEKQHDHEEDHCWRILHVAECFKQACVQR
GIAPSMEIMITEFIMEAEAR
>SlitGOBP2
MTSKCCLLLVLMAAATSSVMGTAEVMSHVTAHFGKALEECREESGLSAEVLEEFQHFWREDFEVVHRELGCAII
CMSNKFSLLQDDSRMHHVNMHDYVKSFPNGHVLSEKLVGLIHNCEKQFDSMTDDCERVVKVAACFKVDAKA
AGIAPEVAMIEAVMEKY
>SlitOBP1
MKEGNRYSHERRITNDSGDQLMVINATDDDYSGYGSGNMGEKLLTSVPRPATPSNNINKNNTSRTKRNEPLLN
RPDSDQCLSQCVFANLQVVDSRGIPREAELWNKVQSSVTSQQSRSALHDQIQACFQELQSEAEDNGCSYSNKL
ERCLMLRFSDRKVDGKGNAKKSSTEQTG
>SlitOBP3
MWMQALVLTLATLATLAAAAVEMDEDMAELARMVRDNCAGETGVDVALVEKVNAGAELMPDDKLKCYIKC
TMETAGMMADGEVDIEAVLALLPPSLAEHNAPALRACGTQRGADHCDTAFRTQQCWQNANKADYFLI
>SlitOBP4
MTKVLFAIVLTMITFAVVLSASTKEAMTTTMSDQVNSIDVDVLAVMDMCNDSYRIDPTYLQALNESGSFIDETD
KTPKCFIRCVFENVGIVSEDGKQFNPARAAVIFAGERNGKPMEDIADMTALCATDRQETCPCDRSYKFLRCLMS
MEIERYEKS
>SlitOBP5
MSVVRCSSLLVAIFCFVSVNAISGDEEAGIKDALRPFVQECADEFGITEEQFEEAKKKASAADIDPCFMSCFLKKA
EFFDSQGKFDVDSTMAFAKEHLTSEPAMKFVEAVGDECVKINDEDVSDGDKGCDRAKLLFECIAETKKKME
>SlitOBP6
MSKFTCLVLCVVAVSLSGVHATAEEKAAFIEAVKPYVQECSKEHGVTPEDIKSAKAAGNADGINSCFLSCVYKKA
EVITEKGEYDADKALEKLKKFVSNEDDYAKFANIGKKCASVNEKSVSDGEAGCERAALLTSCFLEHKSEISA
>SlitOBP7
MDQKRICLFVIAMFLASGSDAMSRQQLKNSGKMLKKNCMNKIGVTEDQIGSIDKGKFIEDRKVMCYIACIYELT
NVIKNNKLNYEASIKQIDLMYPPDVKESAKAAVEKCKDVQKKYKDICEASFYAAKCMYEFKPEDFIFA
>SlitOBP8
MLLTKIVKFFILVATCEAMTMKQIKNTGKMMRKTCQPKNNAEDEKIDPISDGVFIDEKEVKCYMACIMKMANT

IKNGKLNYDAAMKQADLLFPDDIKEPAKEAITACRKVADAHKDICDASFHVTKCIYNHNPGIFYFP
>SlitOBP9
MCLVKYHVLVLCVILVGSYALNCRSSGGPKEAELKNIYKKCLKMQEGKNSSKGNSAQDWKEPRVQIQRNDWDR
GRVGSKENKNSRDDSRSGSKDKKGDSGMRDNRNDMMSRRDDMMSRGDERNDNRKHRTDDRMGNDND
RSGNRGRGNKNNRNDMNGGRDDRFGRDDYFNGREDFPQSDEYGGDMGQYNNNYYSTTQSSRRYKRERRP
SNSGQRSQYNPNNHKISGYEDNFRSDERNTTDNNSSKETDNKSCALHCFLENLEMTGEDGMPDRYLVTHAIT
KDVKNEDLRDFLQESIEECFQILDNENTEDKCEFSKNLLICLSEKGRANCDDWKDDLTF
>SlitOBP10
MVRKISGLLCCLCVFGISFSDSAISADSESRCRNPPTAPQKIERVITLCQDEIKLSILREALDVIKEEHTMPAQRRRD
KREVPFTHDEKRIAGCLLQCVYRKVKAVDGYGFPTLEGLVGLYSDGVNERGYFMAVLEASRECLMKNHDKFSRT
VPMDNGRNCDISFDIFECISDRIGEYCGTSGL
>SlitOBP11
MKSFVVFCIVFVVGVCATEKGNKIASECIKESGVKSDVLAEAKKGNLGDDPAFKEFTYCFFKKVGIVGEDGKLNR
DVAIAKLPSGVDKAEAEKLLDSCKSKTGKDAVETVYEIFKCYQHGTKSHIMFAS
>SlitOBP12
MSVVRCSSFLVALFCFVSVNAMSGDEEAGIKEALRPFVQECADEFGITEEQFEEAKKKASAADIDPCFMSCFLKK
AEFFDSQGKFDVDSTMAFAKEHLTSEPAMKFVEAVGDECVKINDEDVSDGDKGCDRAKLLFECIAETKKKME
>SlitOBP13
MITSCLLVLSAVVQVLLAKQPVFESGPPEPWGPPERTSHPGQFQPRVPKRCWVPPQRINVYNCCPIPTLYPDED
MQSCGFEKLSENKPQKPVYRPEGTCKEGYCVMGKFDLLLANNSVDYVKFREYLDNWAESYPEFANAIHIAKEE
CAQDGGPEVPPICEPDKLFLCLTSTIFWNCKLRDGEGCAALQEHMNECKQYYTRVMAPTIKDFEVR
>SlitOBP15
MYSINCFIFSVILIVMFDNCFVYSMTREQIKNSGKLIKKTCSAKNDLTEDEVKDVDKGKFIEKKDFMCYIACVYKM
GQSVKGSTINHDMMLRQVDMMFPNDMKAPVKSAIEHCRPVAKNYKDLCEASYWTAKCIYDFDPANFMFP
>SlitOBP16
MYRFVILSIVLVSALADDIDIRECGRIFHPPPHGCCKANNAVKNKDMLAEELKDCFDGSGPKDPMKCEIDLCIAK
KKGFATDDGKLDIKKFEEVITKEVGSDKDLLDEIKTNCINGDLNNYGPPEFCDFMKIKHCVTLHMMNHCSEWS
DDGNCKVVKELVGKCAKVI
>SlitOBP17
MKTFRLLCCILSIFLFFDQSYGMTRQQLKNSGKLMKKSCMPKNDVTEDEVGDIEKGKFIETRNVMCYIACVYTM
SQVVKNNKLSYEAVIKQVDVMFPAEMRDAVKAAATHCKETTKKYKDLCESSYWTAKCMYDYDAQNFVFP
>SlitOBP18
MFKLCVFLALGFVACHGAPNSSPGTPNANPGTYCGVTPDNIYRCLNNPRVVTPEVSTKCGSQFTECEKMTCIFR
ELKWSKRGAIDKAKVRAYFDQYETEHPEWAQAVQHVKAFCLASELRAQGVFLNCPAYDIMQCVLASFIKHASP
SVWSTATDCAYPKAYAADCPVCPSDCYSPQIPFGSCNACYTQPRTV
>SlitOBP19
MFRRTLLLFSIIYISACNGQTEAPEKNRMMGIDAVHDNNVKIDKDTIITRNLKLEKRSRGPKSVSNKNEDQIEPD
WSYANFPKEVSEHVEKFKKNMTECLKEVQTSDKRPVKRLSPKMESPVHGECLIACVLKRNGVIINGKVNKDNLI
ALVSKFYSKDTRLMKKLEKNLDRCIEMSVRAQDDCALALVLNDCTNDLMASNKHKIMVNY
>SlitOBP20
MEKILIFTFITLSGFAHARISVMYAHDKLSDLVAQQCLSEMYPKNKRIEIQESDEPCIIFCVLKKFGIISASGVINLDIY
RKRVQIAHQLDQKTSIMDYGGSCMENAEATQHKQDVCKKAKVFNDCTHLYRILLM
>SlitOBP21
MARRQRGAMFTEALPLFVILVAVTHGGKNKPVFSDEIKEIIQTVHDECVAKTGVAEEDITNCENGIFKEDAKLKC

YMFCLLEEASLVDDDDTVDYDMLVSLIPDEYYERTTKMIFACKHLDTPDKDRCQRAFEVHKCSYEKDPDLYFLF
>SlitOBP22
MSKFTCIILCVVAASLTKVSHAAVTEEEKEAFREAMAPIIAECSEEHGVSEADIKAAKESASADNIKPCFLGCVMK
KIEVLDAKGLYDAETGLGKLRKFVKDDDEFAKFEDIAKKCLKVNDESVSDGEAGCDRAKLVLGCFIEHKVEMPF
>SlitOBP23
MAKFSCLVLCVVAASLGSIHVASGESLRESLRPVIVACSQEHGVTDAEIQAAKDAGSPASIKPCFIACVFKKAGFIN
EQGQLDLETGLKNLRQFVKDDEQYKKLEEVAKKCSQVKDKAVSDGAAGCERGVLLAGCFLEHKTSIII
>SlitOBP25
MAKVTCIVLFVVGVSLSSIQADDGKNESEVEIDVNQIIDDCIEEYHIPRRLFLAAAETGSTHALTPCFWSCCFKGV
GVLNSEGQYDIDATLDLSKKIFTDHEYEKVEIIVKKCESVNGAPVSNGNIECEKSVLLADCLFDNAKKHFPNMFG
VDY
>SlitOBP27
MYKFVILCSIFVAASNADVAQTLTKRETKASLKPLSVCCDIPELADEFQLAKCSPRPPGPCEDVQCIFEVSGFLTDR
NTLNKAAYRSHLQKWEKNHPGWTDSIYKAITDCVDNDPRQHLEVPCKAYDVFTCTGIAMLKKCPDTAWKC
>SlitOBP28
MIVRFLLCLYIVEFYGAHARTDQEIKAWFFREGMDCNIEHPISPKEMLELKENKIPDTNNAKCFVACVFKKTGML
DSKGMFDAENSIAMTQKDFANDPNRLESSKKLLEACKKVNDEAVSDGEKGCERSVLLHKCFVETAPQLGIKLP
>SlitOBP29
MWNLLVVFLAICSCVYARRRSSGAEINGLTEEELKMEFTKLIMKCNKDGEVDMTELVQLQNYVVPTKQSTKCVL
ACAYKAAEVMNAKGEYDIDHAYKVAEMMKNGDEKRLVNAKKMADLCVKVNELSVSDGEKGCDRAAMIFKCT
VENAPKFGFKL
>SlitOBP33
MTCSQALALLALVAISQQATTGCKNCIMLGKEEKAMFRAHSDACVAASRVEPRLVDAMLAGELLDEPALRKHV
YCVLLKCKLISKDGKLQKAAVLGKMAARPDAKNATKVLESCADQTGDTPEDLAWNLFRCGYDKKALLFDYMPT
NVASETDNNS
>SlittoPBP1
MANARWRFVFVVYALYLTSAVLGSQDLMAKMTKGFTRVVDDCKTELNVGDHIMQDMYNYWREDYQLINRD
MGCMLLCMAKKLDLMDDQTMHHGKTEDFAKSHGADDDVAKKLVSVIHECEQQHTGIADDCMRVLEVAKCF
RTKIHELKWAPSMEVIMEEVMTAV
>SlittoPBP2
MSLRVALVVAASLLVVVQASQDVMKNLAINFAKPLDDCKKEMDLPDSVTTDFYNFWKEGYELTNRQTGCAILC
LSSKLEILDQELNLHHGRAQEFAMKHGADEAMAKQIVDMIHTCAQSTPDVAADPCMKTLNVAKCFKLKVHEL
NWAPSVELIVGEVLAEV
>SlittoPBP3
MGSRNVFVALVVLTVAMRETEPSKDPMKYIASGFVKVLEECKHELNMNDHLIADLFHYWKLEYTLLNRDTGCA
IICMGKKLDLLDANGRMHHGNAQEFAKKHGAGDEVASQIVQIIHECEKKHERDDDECLRVLEVAKCFRTGIHEL
NWQPNVEVIVSEVLTEI
>SlittoGOBP1
MLLLLALPLLAAVLPLRADVNVMKDVTLGFGQALDKCRQESQLTEEKMEEFFHFWRDDFKFEHRELGCAIQC
MSRHFNLLTDSSRMHHENTEQFIQSFPNGEVLARQMVELIHACEKQHDHEDDHCWRILHVAECFKQACVQR
GIAPSMEMMITEFIMEAEAR
>SlittoGOBP2
MATVTSSVMGTAEVMSHVTAHFGKALEECREESGLSAEVLEEFQHFWRDDFEVVHRELGCAIICMSNKFSLLQ
DDSRMHHVNMHDYVKSFPNGHVLSEKLVGLIHNCEKQFDSMTDDCERVVKVAACFKVDAKAAGIAPEVAMI

EAVMEKY
>SlittoOBP1
MFKLCVFLALGFVACHGASNSNPGTPNANPGTYCGVTPDNIYRCLNNPRVVTPEVSTKCGSQFTECEKMTCIF
RELKWSKRGAIDKAKVRAYFDQYETEHPEWAQAVQHVKAFCLASELRAQGVFLNCPAYDIMQCVLASFIKHAS
PSVWSTATDCAYPKAYAADCPVCPSDCYSPQIPYGSCNACYTQPRTV
>SlittoOBP2
MVRKISGLLCCLCVFGISFSDSAISADSESRCRNPPTAPQKIERVITLCQDEIKLSILREALDVIKEEHTMPAQRRRD
KREVPFTHDEKRIAGCLLQCVYRKVKAVDGYGFPTLEGLVGLYSDGVNERGYFMAVLEASRECLMKNHDKFSRT
VPMDNGRNCDISFDIFECISDRIGEYCGTSGL
>SlittoOBP3
MKSFVVICIVFVVGVCATEKGNKIASECIKESGVKSDVLAEAKKGNLGDDPAFKEFTYCFFKKVGIVGEDGKLNRD
VAIAKLPSGVDKAEAEKLLDSCKSKTGKDAVETVYEIFKCYQHGTKSHIMFAS
>SlittoOBP4
MKTLLVFAACILVAQALTDEQKEKLKKHRTECLTETKVDEQLVNKLKGGDYKMDNEALKKYALCMMMKSELMT
KDGKFKKDVALAKVPNPADKPTVEKLIDACLANKGNTPHQTAWNYVKCYHEKDPKHAIFL
>SlittoOBP5
MTKVLFAIVLTMVTFAVVLSASTKEAMTTTMSDQVNSIDVDVLAVMDMCNDSYRIDPTYLQALNESGSFIDET
DKTPKCFIRCVFENVGIVSEDGKQFNPARAAVIFAGERNGKPMEDIADMTALCATDRQETCPCDRSYKFLRCLM
SMEIERYEKS
>SlittoOBP7
MFTEALPLFVILVAVTHGGKNKPVFSDEIKEIIQTVHDECVAKTGVAEEDITNCENGIFKEDAKLKCYMFCLLEEAS
LVDDDDTVDYDMLVSLIPDEYYERTTKMIFACKHLDTPDKDRCQRAFEVHKCSYEKDPDLYFLF
>SlittoOBP8
MFGSKIVFSLFMIVSVCYGAVDIKKYFKVCDRNAIDVNDCMAEAVRQGIATMINGIDELGVPPIDPYLQKDFRLE
YKNNQLAAKLTLKNIYVEGLKEAIVHDARLRADDDKFHLEVDLSGPRVSVRSDYYGEGQFNALKIVAYGQVNTT
MTDLVYTWKLAGVPEKNGTETYIRIKDFYMRPDVGSLVTHFRNDNPESRELTDLGTRFANENWRMLYKEFLPY
AQANWNRIGVRVANKLFLKVPYDQLFPTSS
>SlittoOBP9
MCLVKYHVLVLCVILVGSYALNCRSSGGPKEAELKNIYKKCLKMQEGKNSSKGNSAQDWKEPRVQIQSNDNDG
SGNRGRGNKNNRNDMNGGRDDRFGRDDYFNGREDFPQSDEYGGDMGQYNNNYYSTTQSSRRYKRERRPS
NSGQRSQYNPNNHKISGYEDNFRSDERNTTDNNSSKETDNKSCALHCFLENLEMTGEDGMPDRYLVTHAITK
DVKNEDLRDFLQESIEECFQILDNENTEDKCEFSKKLIDLSVRE
>SlittoOBP10
MKEGNRYSHERRITNDSGDQLMVINATDDDYSGYGSGNMGEKLLTSVPRPATPSNNINKNNINRTKRNEPLLN
RPDSDQCLSQCVFANLQVVDSKGIPREAELWNKVQSSVTSQQSRSALHDQIQACFQELQSEAEDNGCSYSNKL
ERCLMLRFSDRKVDGKGNAKKSSTEQTG
>SlittoOBP11
MSKFTCLVLCVVAVSLNGVHATAEEKAAFIEAVKPYVQECSKEHGVTPEDIKSAKAAGNADGINSCFLSCVYKKA
EVINEKGEYDVDKALEKLKKFVSNEDDYAKFANIGKKCASVNEKSVSDGEAGCERAALLTSCFLEHKSEISA
>SlittoOBP12
MSVVRCSSFLVALFCFVSVNAMSGDEEAGIKEALRPFVQECADEFGITEEQFEEAKKKASAADIDPCFMSCFLKK
AEFFDSQGKFDVDSTMAFAKEHLTSEPAMKFVEAVGDECVKINDEDVSDGDKGCDRAKLLFECIAETKKKME
>SlittoOBP13
MITSSSLLVLTAVVQVLFAQQPVFESGPPEPWGPPQRPAHRRQFLPRIPKRCWVPPQRINVYNCCPIPTLYPDED

MQSCGFEKTSGNTDQPQKPVFRPEGTCKEGYCVMGKFDLLFANNSVDFVKFREYLDNWAESYPEFANAIRIAK
QECAQDGGPEVPPICEPDKLFLCLTSTIFWNCKLRDGDGCAALQEHMNECKQYYTRQMEPTMKDIEVR
>SlittoOBP14
MDQKRICLFVIAMFLASGSDAMSRQQLKNSGKMLKKNCMNKIGVTDDQVGSIDKGKFIEDRKVMCYIACIYEL
TNVIKNNKLNYEASIKQIDLMYPPDVKESAKAAVEKCKDVQKKYKDICEASFYAAKCMYEYKPEDFIFA
>SlittoOBP15
MFNNCFVYSMTREQIKNSGKLIKKTCSAKNDLTEDEVKDVDKGKFIEKKDFMCYIACVYKMGQTVKGSTINHD
MMLRQVDMMFPNDMKAPVKAAIEHCRPVAKNYKDLCEASYWTAKCIYDFDPANFMFP
>SlittoOBP16
MLAEELKDCFDGSGPKDPMKCEIDLCIAKKKGFATDDGKLDIKKFEEVITKDVGSDKDLLDEIKTNCINGDLNNY
GPPEFCDFIKIKHCVTLHMMNHCSEWSDDGNCKVVKELVGKCAKVI
>SlittoOBP17
MRTFRLLCCILSIFFIFDQSYGMTRQQLKNSGKLMKKSCMPKNDVTEDEVGDIEKGKFIETRNVMCYIACVYTM
SQVVKNNKLSYEAVIKQVDVMFPAEMRDAVKAAATHCKETTKKYKDLCESSYWTAKCMYDYDAQNFVFP
>SlittoOBP18
MILXYTQKLTNMLLTKIVKFFILVATCEAMTMKQIKNTGKMMRKTCQPKNNVEDEKIDPLSDGVFIDEKEVKCY
MACIMKMANTIKNGKLNYDAAMKQADLLFPDDIKEPAKEAITACRKVADAHKDICDASFHVTKCIYNHNPSIFY
FP
>SlittoOBP19
MYSKICILLFISYTCLVTADSVSFIKKCKWDDGKCAKESGQNVIQKFAAGISEYNVGVSDPLHIEYVDASSPNMKLI
VTDVVVTGLRNCEVKKIQRFEDSSKLIVKLLCAAELNGKYDMKGQLFVIPIEGNGGLYSKVPKIQINAEVDLNTKQ
GKDGKDHWIVKSWRHTFELKDKSTVKFENLFPDNEFLRTSTNELIAQNGNDVIIEIGANLIKAIVGKIVENIKKFFI
AVPIEDLSL
>SlittoOBP20
MWVQALVLTLATLVTLVAAAVEMDEDMAELARMVRDNCAGETGVDVALVEQVNAGAELMPDDKLKCYIKCT
METAGMMADGEVDIEAVLALLPPSLAEHNAPALRACGTQRGADHCDTAFRTQQCWQNANKADYFLI
>SlittoOBP22
MNRLLLVYLVVLYAGCSYGMTRAQVKKTMGIIKNQCMPKNSVTEEQVGRIEQGVFIEDRNVMCYVACIYKSLQ
VVKNDKLDMALITKQIDILYPPELKEPVKKSVAACFHSQDNYSDFCEGVFYASKCLYEKDPASFIFP
>SlittoOBP26
MFNSSVFMYCLYFCALTPYLVSAMTAEQKALIHEHFETIGKSCNKDSTMITAEDIANLRAKKIPTGPNAPCFLAC
MMKQIGVMDDNGMVQKETALEMAKAVFDDPEEIKAIEDYLHSCSHINTESVSDGAAGCERAMLAYKCMTEN
ASKFGFDI
>SlittoOBP25
MSKLSCIVFCAVAMRLCVFVASEDANSIFHAPIKPIVIDCAKEYGLSEDDIKKNRGLDGLKNLPPCFIRCVLNKLDII
NDKGQYDADSGIATIKGLMSNNEYLEKISGVLKECESVNEKSVSDGDAGCERALLGAMCYLDHKTIVLA
>SlittoOBP24
MYKIISLVFCIAVCLNRVHGNAEDKIAIMTAVKPFVEECAKKHGVTFEALLTAKASGKIDGVEPCFYSCVYKKTEFL
NSKGEYDVDTALAKLKKYISNDDDYAKLSQVGKRCASVNSKPVGDGEAGCERGVLLTQCFLDHKGSVPM
>SlittoOBP28
MIVRFLLCLYIVEFYGVHVQARTDQEIKAWFFREGMDCNIEHPISXKEMLELKENKIPDTNNAKCFVACVFKKTG
MLDSXGMFDAENSIAMTQKDFANDPNRLESSKKLLETCKKVNDETVSDGEKGCERSVLLHKCFVETAPQLGIKL
P
>SlittoOBP23

MAKLSCLVLCVVAASLGSIHVAKGESLRESLRPVIVACSKEHGVTDAEIQAAKEAGSPASIKPCFIACVFKKAGFIN
EQGQLDLETGLKNLRQFVKDDEQYKQLEGVANKCSPVKDKAVSDGAAGCDRGVHLAGCFLDHKTSIII
>SexiPBP1
MAGAKWQFVCVVFALYLTSAALGSQELMMKMTKGFTKVVDDCKAELNAGEHIMQDMYNYWREDYQLINR
DLGCMILCMAKKLDLMEDQKMHHGKTEEFAKSHGADDEVAKKLVSIIHECEQQHAGIADDCMRVLEISKCFRT
KIHELKWAPNMEVIMEEVMTAV
>SexiPBP2
MAFCRSATMSVRVALVVAASMLVVVQASQDVMKNLAINFAKPLDDCKKEMDLPDSVTTDFYNFWKEGYELT
NRQTGCAILCLSSKLEILDQELNLHHGRAQEFAMKHGADETMAKQIVDMIHTCAQSTPDVAADPCMKTLNVA
KCFKLKIHELNWAPSMELIVGEVLAEV
>SexiPBP3
MGSHNVFVALVLLAVGMRVAEPSKDAMKYITSGFVKVLEECKQELNMNDHIIADLFHFWKLEYALLSRDTGCVI
ICMSKKLDLLDANGRMHHGNAQEFAKRHGAGDDVASKIVQIIHDCEKKHERDDDECLRVLEVAKCFRTGIHDL
DWQPKVEVIVSEVLTEI
>SexiGOBP1
MLFLLRALPLLAAVLPLRADVNVMKDVTLGFGQALDKCRQESQLTEEKMEEFFHFWRDDFKFEHRELGCAIQC
MSRHYNQLTDSSRMHHDNTEQFIKSFPNGEVLARQMVELIHSCEKQYDHEDDHCWRILHVADCFKQGCVQR
GIAPSMEMMMTEFIMEAEAR
>SexiGOBP2
MTAEVMSHVTAHFGKALEECREESGLSAEVLEEFQHFWREDFEVVHRELGCAIICMSNKFSLLQDDTRMHHV
NMHDYVKGFPNGHVLSEKLVELIHNCEKRFDSMTDDCERVVKVAACFKVDAKAAGIAPEVAMIEAVMEKY
>SexiOBP1
MSKFTCLVLCVVAGCLSGVHATAEEKAALIEAVKPYIQECSKEHGVTPEDIKSAKEAGNADGINACFLRCVYNKA
GVINDKGEYDADKALEKLKKFVSNEDDYAKFAEIGKKCASVTETSVSDGEAGCERAALLTSCFLEHKSEVHA
>SexiOBP2
MKSFVVFCIVLVVGVCANEKGNKLDRPFASECIKETGVKNELLEEAKKGIISEDPAFKAFTYCFFKKIGIVGEDGLL
NRDVAIAKLPSGVDKSEAEKLLDSCKSKTGKDAVDTVFEIFKCYQQGTKSHIMFAS
>SexiOBP3
MVKLTCVVFCAVAMALSVFVAGEDANSVFQGAIKPLIAECAKEYKLSDEELLKNRGLAGLSNLPPCFIGCVLKKF
DIINDKGLYDAEAGIAKIEKLLPNNEFLDKISGVLKSCESANEKSVGDGDAGCERAVLVATCYLEHKTAVIA
>SexiOBP4
MWNFLVVFLAICSCVYGLTEEELKMEFTKLIMKCNKDGKVDMTELVQLQNYVVPTKQTTKCVLACAYKAAEVM
NAKGEYDIDHAYKVAEMMKNGDEKRLVNAKKMADLCVKVNEQSVSDGEKGCDRAAMIFKCTVENAPKFGFK
L
>SexiOBP5
MTMKQIRNTGKMMRKTCQPKNNVEDEKIDPIAEGVFIDEKEVKCYMACIMKMANTIKNGKLNYDAAIKQAD
LLLPDDIKEPAKEAITACKKVADAHKDICDASFHITKCIYNHNPGIFYSP
>SexiOBP6
MLGSLLFVFAFSVFSLGAEALLIDDLKQKYADSILQCSQQYPLDRADAELLQNKVMPDKESTKCLFACVYKVTGV
MSDQGELSVEGVNALSQKYLADDPEKLKKSEEFTEACRTVNDAPVSDGARGCDRAALIFKCTIEKSPDFSFV
>SexiOBP7
MTKVLFAIVLMMITFAVTLSASTKEAMTTTMTDQVNSIEVDVLAVMDMCNDSYRIDPTYLQALNESGSFIDET
DKTPKCFIRCVFENVGIVSEDGMQLNPARAAVIFAGERNGKPMEDIADMTALCATDRQETCPCDRSYKFLRCL
MSMEIERYEKS

>SexiABP1

MSVVRYSSFVVALFCLVSVNAMSGDEEAGVRDALRPYVQECADEYGITEEQFEEAKKKASADDIDPCFMSCFLK
KAEFFDAQGKFDVDSTMAFAKEHLSSEPAMKFVEAVGDECVKINDEDVSDGDKGCDRAKLLFDCIAETKKKM
D

>SexiOBP10

MDRKRICLFVIAMFLASGSDAMSRQQLKNSGKMLKKNCMNKIGVTEDQVGSIDKGKFIEDRKVMCYIACIYEL
TNVIKNNKLNYEASIKQIDLMYPPDIKESAKAAVEKCKDVQKKYKDICEVSFYAAKCMYEFKPEDFIFA

>SexiOBP8

MARRQQGAMFTETLPLFVILVAVTHGGKDKPVFSDEIKEIIQTVHDECVAKTGVAEEDITNCENGIFKEDAKLKC
YMFCLLEEASLVDDDDTVDYDMLVSLIPDEYYERTTKMIFACKHLDTPDKDRCQRAFEVHKCSYEKDPDLYFLF

>SexiOBP9

MKTLFVFAACILLAQALTDEQKEKLKKHRTECLSETKVDEQLVNKLKGGDYKTESEPLKKYALCMMMKSELMTK
EGKFKKDVALAKVPNPADKPTVEKLIDACLANKGNTPHQTAWNYVKCYHEKDPKHAIFL

>SexiOBP11

MKEGNRYSHERRITNDSGDQLMVINATDDDYSGYGSGNMGEKLLTSVPRPASSSNNINKNNTRRTRRNEPFL
NRPDSDQCLSQCVFANLQVVDSRGIPREAELWNKVQTSVTSQQSRSALHDQIRACFQELQSEAEDNGCSYFNK
LERCLMLRFSDRKVDGKGNPKKSSTEQT

Supplementary Table 5.

>BmorGOBP1
MWKLVVVLTVNLLQGALTDVYVMKDVTLGFGQALEQCREESQLTEEKMEEFFHFWNDDFKFEHRELGCAIQC
MSRHFNLLTDSSRMHHENTDKFIKSFPNGEILSQKMIDMIHTCEKKFDSEPDHCWRILRVAECFKDACNKSGLA
PSMELILAEFIMESEADK
>BmorGOBP2
MFSFLILVFVASVADSVIGTAEVMSHVTAHFGKTLEECREESGLSVDILDEFKHFWSDDFDVVHRELGCAIICMS
NKFSLMDDDVRMHHVNMDEYIKGFPNGQVLAEKMVKLIHNCEKQFDTETDDCTRVVKVAACFKKDSRKEGI
APEVAMIEAVIEKY
>BmorPBP1
MSIQGQIALALMVYMAVGSVDASQEVMKNLSLNFGKALDECKKEMTLTDAINEDFYNFWKEGYEIKNRETGC
AIMCLSTKLNMLDPEGNLHHGNAMEFAKKHGADETMAQQLIDIVHGCEKSTPANDDKCIWTLGVATCFKAEI
HKLNWAPSMDVAVGEILAEV
>BmorPBP2
MKLQVVLVVLTVEMVCGSRDVMTNLSIQFAKPLEACKKEMGLTETVLKDFYNFWIEDYEFTDRNTGCAILCMS
KKLELMDGDYNLHHGKAHEFARKHGADETMAKQLVDLIHGCSQSVATMPDECERTLKVAKCFIAEIHKLKWAP
DVELLMAEVLNEVSWKS
>BmorPBP3
MARYNIVVAVLVLGVVGARGSSEAMRHIATGFIRVLDECKQELGLTDHILTDMYHFWKLDYSMMTRETGCAIIC
MSKKLDLIDGDGKLHHGNAQAYALKHGAATEVAAKLVEVIHGCEKLHESIDDQCSRVLEVAKCFRTGVHELHW
APKLDVIVGEVMTEI
>BmorOBP5
MKQRLRVLLLRFCILQTVLSESGVDVVKNLSLSFARFFLECDEERHFQPEVRLKVMTFWYSESSTWDRDVGCAF
LCIFKKMEIDNPQDPSYRTHLELLSFANSEDNKIANQMVEIFYACGENTETDPCLWALEQVKCYKNRINQLGLTP
TF
>BmorOBP8
MLRVVVICVCFLVIAPYGINASSLDDLKMVYKNVIKECVGDYPITAADLKLIKARQIPNDDIKCVFACAYKKTGM
MTEEGMLSVEGIKDMSQKYLSDNPEQLRKSKEFAEACSSVNDQQVSDGTKGCERAALIFKCSTEKITNFGFEL
>BmorOBP9
MLRVVVICVCFLVVAPYGINAVSYEQKIKIRDQLDRAGFECFKDHKITEDDIKNLRANKPATGENVPCFIACVMKK
TGVMNDQGVIRKGPVLELAKKVLADDKDIKKLQDYIHSCSHVNSETVHDKGKGCEFAMQAYTCMSANASKFG
FNI
>BmorOBP10
MLRVVVICVCFLVIAPYGINAVSDEQKIKIREQIDKSGFECFKDHKITEDDIKNLRARKPATGENVPCFIACVMKKT
GVMNDQGVIHTEPVLQLAKKVLTDDKDIKKLQDYIHSCSHVNSKTVHDKGQGCEFAIQTYTCMSANASKFGFD
V
>BmorOBP11
MSANSFVVLAFCALAVGVNALTEEQKAEITKSSLPLIAECSKEFSVNQGDIDAAKKLGDPSGLNSCFVGCFMKKA
GIINASGLFDVAATIEKSKKYLTSEEDLKAFEKLTETCAPENDKPVSDSDKGCERAKLLLDCFVANKGSFSVFSL
>BmorOBP12
MTSFMVFFVLSVLTLKYSDALTDEQKNKIQSKFIEIGAECIVEHPISIDDINSFKNKKFPSGVNAGCFVACIFNKIGL
FDDKGNLSHNSALEKAKGIFNADEEVKNLEEFLNRCAKVNGEAVGDGVKGCERAKLAYNCLIENSLEFGFNIDF
>BmorOBP13

MLKIHVLLCFGMAILYFGSAKAVTPEESKAFEAFAKPVIEQCQKDFGMDKESFAQKNLDEIDECLIACVVEKFGIT
NDEKIDGDALKALVTKFVGNEEERNKINKIVEECTEDANKSGDGTCNTSTILFLCLLKNGKDLWGF
>BmorOBP14
MSRQQLKNSGKMLKKQCMGKNDVTEEEIGDIEKGKFIEQKNVMCYIACIYQMTQIIKNNKISYEASIKQIDLMY
PPELKESAKASAGRCKDVSKKYKDICEASYWTAKCMYEDNPKDFIFA
>BmorOBP15
MFLKNIFIECVLLYFVMLNTSFVNTMTKQQIKNSGKILKKACISKNDVTEDQISDIDKGKFIEDKNVMCYIACVYS
MSQVVKNNKFVHDAMVKQVDMMFPTEMRDAVKASIANCRGVAKNYKDICEASFWTAKCMYEFDPANFVF
A
>BmorOBP16
MRISFLFLISVTIITFDSVFAMTRAQVKKTMTIMKNQCMPKNGVTEDQVGKIEEGIFLENHNVMCYIACVYKTI
QVVKNDRLDKDLISKQIDVLYPQEIRESTKKAVGDCINLQEKYDDWCEGIFRSTKCLYEKDPANFIFP
>BmorOBP17
MTRQQLKNSGKIMKKTCMPKNDVTEEEIGQIEQGKFLEQRNVMCYIACIYTVTQVVKNNKLSYDAVIKQVDV
MFPAEMRPAVKAAAENCKDISKTFKDICEASYWTAKCMYDFDPKNFVFP
>BmorOBP18
MILIVIAKFLILISLCETMTMKQIKNTGKMMRKSCQPKNNVDDEKINPINDGVFIEENEVKCYIACIMKMANTM
KNGKLNFEAAMKQADLLLPDEMKEPTKEAIVACRKVADSYKDVCDASFHVTKCIYNHNPSVFFFP
>BmorOBP19
MTSAKTDVEIKAWFLGQAVECSKDHPVTTEELRMHKHELPDSKNAKCLMKCVFRKCNWLDSKGMYDINAAY
ASSTKDFSDDKTKQENANKLFDTCKSVNEENVGDGEEGCDRSLLLAKCLTKAAPQVSIYYS
>BmorOBP20
MAVHIFLILASYMALAAHGQLDDEIAELAAMVRENCADESSVDLNLVEKVNAGTDLATITDGKLKCYIKCTMET
AGMMSDGVVDVEAVLSLLPDSLKTKNEASLKKCDTQKGSDDCDTAYLTQICWQAANKADYFLI
>BmorOBP21
MITASLHVIFALLAFVYGGKDKPVLSEEIKEIIQTVHDECVGKTGVSEEDITNCESGIFKEDVKLKCYMFCLLEEAGL
VNDDGTVDYEMFTSLIPEEYFDRATKMIFSCKELDTPDKDKCERAFEVHKCSYEKDPDFYFLF
>BmorOBP22
MLKVFVVVVCTLGASQLCAALYTQKVAVSFPKDKTTIVVEAMKSCIAKTGANPNVIEVISSGKVSEDEKFKEFFYC
ACNDIGVVNPDGHIKVKECIELFPKETQPLVEPVIKNCDKEGVNKYDTLFKYLKCFQETSPVRVTLA
>BmorOBP23
MTSKVLLSCVVLAVLATTVLAEDSRKLVSFAPEVAKKLKVLIQECLNENGLGEDAIEVIRAGEYREDEPFQNLVYCA
YKKFGALDENNRIISQVAAASFPKDIDVVTVIESCGKEDGNTPVEQVFKYFKCFQKNSPVRMQLY
>BmorOBP25
MKSVVLICLAFAVFNCGADNVHLNEDEREKANWYTAECGVETGVSTEVINAAKIGKYSKDKAFKKFVLCFFKKS
AILNSDGTLNMVVALAKLPSGVNKSEAQSVLEQCKNKTGQDAADKAFAILQCFHKGTKTHILF
>BmorOBP26
MKSVVLICLAFAVFNCGADNVHLAETQKEKAKQYTSECVRESGVSTEAINAAKIGKYSKDKAFKNFVLCFFNKSA
IFNSDGTLNMDVALAKLPPGVNKSEAQSVLKQCKNKTGQGAADKAFEIFRCYYKGTKTHILF
>BmorOBP27
MKSVVLICLAFAVFNCGADNVHLTETQKEKAKQYTSECVKESGVSTEVINAAKTGQYSEDKAFKKFVLCFFNKSA
ILNSDGTLNMDVALAKLPPGVNKSEAQSVLEQCKDKTGQDAADKAFEIFQCYYKGTKTHILF
>BmorOBP28
MLKVFIVTFFAFQLSAIARLQANGCVAVPFPKDKTIIIVEAMKSCIAKTGANPNFIDVIRSGKVSEDEKFKEFYYCT

CNDTGFVNPDGHIKVKECIELFPKETQPLVEPVIKNCDKEEGVNKYDTLFKFLKCFQETSPVRVALA

>BmorOBP29

MTGPAAAAVLLALLAAAGQATTGCKNCVILGKEERAMFRSHSDACLAQSRVEPRLLESMMNGELIDDAALRK
HVYCVLLSCKMIGKDGKLLKAAILGKLAARPAGRDVTKVLEACAEQPGASPEDVAWNIFRCGYNRKAVLFDYM
PAGGASSGNTENHP

>BmorOBP30

MRSFVILLNYGLLCCGQFMAEDYYYDIVTRDPDDLMREKENEVRALRAFQADCAEDVQVKPDLVVNLKSGDW
QTEDVSLKKWALCVLMKLGLMTAQGVFKMNEAMSKIPDMNDKIIAEKLIDDCLSLQATTPHDAAWNYIKCHH
QKDPEGNFSSLNIF

>BmorOBP31

MKTFIVFVVCVVLAQALTDEQKENLKKHRADCLSETKADEQLVNKLKTGDFKTENEPLKKYALCMLIKSQLMTK
DGKFKKDVALAKVPNAEDKLKVEKLIDACLANKGNSPHQTAWNYVKCYHEKDPKHALFL

>BmorOBP32

MYSHKYLNDFTNIPEILIILLSSVALMSYGYNTKLFSHSLGSEPSLSILYARDKKSDKVTNECLMEMYPKNLYKYPLR
IDRNDIPCIIHCVLKKFGIISNDGFINIKNYYRRVQAIHRYDPRILISDVGETCAQNINGMNLDHDVCKKAKVFND
CTQLYAISYREPEDW

>BmorOBP33

MYAHDKLSDMIADQCLNEMYPRSKRLEIEESDEPCIIFCVLKKFGIMSPTGVINLEAYRKRVQLPEQLAQRNSIN
DFGSACLESAEATQHKQDVCKKAKVFNECTHLYKILLK

>BmorOBP34

MEKMILLNVFAVVLPCVLASRTRGSSGTLVDFTDPKVQGHLDALVRMAQSCVIKVRATPKDVRAYFTNSSPVSR
SGQCFATCMLEQSDIINHGKVNRDLLVHLAGLVNGKNSRVVRKLNSVSRLCLDSISGMTDRCQLASTYNDCLNE
NMIEFAFPLDIAEEAVRKMPFHLIQPK

>BmorOBP36

MAVSEISRILTFLTIVSFIYIVYSFKPLTKDEHIERYNKMNEDIEPFRKNLTECARQVKASMADVEKFLKRIPQSNME
GKCFVACILKRNSLIKNNKLSQENLLEVNRAVYGDDSEVMSRLKTAILECSKIVEDIFEICEYASVFNDCMHMKM
EHILDKITMERRMEALGQMSSNPDEWSEEEDEMLKLVKDEL

>BmorOBP37

MFYPFRFTLLFYGLFVIYLVRAEPEKENHFTLALKKTLFSTARSCMSHVNANETDLEYLRKDPPFPDKAACIIKCLLE
KIGVVKNNKYSKMGFLTAVSPLVFTNKKKLDHYKSVSENCEKEINHDQTTECELGNEVVSCIFKYAPELHFKT

>BmorOBP38

MANLVLLLTFVLMTLSMARLKSTEAPKSKTALFNDQDNMGYEELDMEEIMSACNESFRIEYAYLESLNDSGSFP
DETDKTPKCYIRCVLEKTEILSENGVLNPATAALVFAGERNGKPMSDLEEMAVACADRHEKCKCEKAYNFVKCL
MYMEIDKYEKKN

>BmorOBP39

MVRKISALLCCFCVLGISMCDSAISTDNEQRCKNPPTAPQKIERVITLCQDEIKLSILREALDVIKEEHTMPAERKR
NKREVPFTHDEKRIAGCLLQCVYRKVKAVDGFGFPTLEGLVGLYSDGVNERGYFMAVLEASRECLMKNHDKFS
RTTPMDNGRNCDVSFDIFECISDRIGEYCGTSGL

>BmorOBP40

MSEFIQPSWRTQCNFRLNWDNRNRLSIDISHGAATTQTPVPTTKPKALRDFMVVPQSCDKTTCVFKKLNIVSD
KGVVDVKSFIKLLDKFTNSYPVWNSAKARVITTCLRKSLIAYDGGCELNNILACTFDVLSENCPLNGNNQTC

>BmorOBP41

MLTILFLLPIVVGVLSGNIPEQPRVYCGELPNTIYSCLGNPKIIQPEVSEKCNKPISECDKTRCIFKESGWAKNNVID
KKKVSDYFEQFAKDNPDWSAAVQNFKTTCLSDSLKPQGVDTNCPAYDIIHCALISFIKFASPSQWSTSEQCVYPR

QYAGACPVCPERCFAPSVPNGSCNACLALLRTP
>BmorOBP42
MMGYACVFVILAVLQAISAEDPPGLPPFLKDAPEKCKSPPRVKNPNECCISEPFFKEADFIECGIEKPGSERGPPD
CSKQNCLLKKYNLLKNDETPDIEAIKSLLDKYIEKNPSFKSSVEKAKECLREDLPGPPQICLANRMTLCIGTVLLME
CPDEKWNTTDDCKAFKDHMTECQKYFPK
>BmorOBP43
MKVCVLFAIFTVAQAAKATLKPISACCNIPELGNPEPLAECSNPKLPGPCKDIQCVFEKSGFLTENKTLIKEAYKTH
LRQWAKEHEGWSVAVEKAISDCVDKDLRQYLEFPCSAYDVFTCTGIAMLKKCPNEHWTC
>BmorOBP44
MSRLVLFFTILVVLQEFIINLYFNFITEIDSCCVKKYPKLFDSEFITECYNTQRKANDKCERDMCVARKLNLLTEEDSI
NKDALLRFVEEGFKTEIDLVNAIKKKCFEEDISNIGKPEMCEVAKYKICITSRMAEDCPKWDSKGICSSAQQKVEN
FMKMLS
>HarmPBP1
MEFHRSTMMSVRLALVVAVCLFIRVDASQDVIKNLSMNFAKPLEDCKKEMDLPDSVTTDFYNFWKEGYEFTN
RQTGCAILCLSSKLELLDQELKLHHGKAQEFAKKHGADDAMAKQLVDLIHGCAQSTPDVADDPCMKTLNVAKC
FKAKIHELNWAPSMELVVGEVLAEV
>HarmPBP2
MAASRWLFARAFCLVLMMGSAMSSKELLTKMTGGFTKVVDACKTELSVGDHIMQDMYNFWREEYQLVNRD
LGCMIMCMTAKLDLIGDDQKMHHGKAEEFAKSHGADDALAKQLVGLIHGCETQHQAIEDHCSRALEIAKCFRT
KIHELKWAPSMEVIMEEIMTAA
>HarmPBP3
MGSRHVFFALVVLAVSVRKAEPSKDAMQYITSGFVKVLEECKHELNLNEQILADLFHFWKLEYSLLGRDTGCAII
CMSKKLDLLDANGRMHHGNAAEFAKKHGAGDEVASKIVTIIHECEKKHEQDGDECLRVLEVAKCFRTGIHELN
WQPKVEVIVSEVLTEI
>HarmGOBP1
MPGVLRALLVLAAAAPLLADINVMKDVTLGFGQALDKCREESQLTEEKMEEFFHFWRDDFKFEHRELGCAIQC
MSRHFNLLTDSSRMHHDNTEKFIQSFPNGEVLARQMVELIHSCEKQFDHEDDHCWRILHVAECFKGSCVQRG
IAPSMELMMTEFIMEAEAR
>HarmGOBP2
MTSKSCLLLVAMATLTASVMGTAEVMSHVTAHFGKALEECREESGLSAEVLEEFQHFWREDFEVVHRELGCAII
CMSNKFSLLQDDSRMHHVNMHDYVKSFPNGHVLSEKLVELIHNCEKKYDTMTDDCDRVVKVAACFKVDAKA
AGIAPEVAMIEAVMEKY
>HarmOBP1
MSKFTFFVLCVVAVSLSKVYASDEDKAKLHEALKPLVEECMKDHEVSLDDLKAAKEAKSADGVKPCFLACVYKK
AEVLNDKGEFDADHALEKLKEFVSDEDVLAKVAEVGNTCKAVNDKAVSDGDAGCERAALLTACFLEHKAEILV
>HarmOBP2
MMDRKRLCLLIIAMFLAQGSDAMSRQQLKNSGKMLKKNCMNKNQVTEDQIGSIDKGKFVEDKKVMCYIACI
FEMTNVVKNNKLNYDASIKQIDLMYPPDLKESAKAAVEKCKDVQKKYKDICEASYWTAKCMYDFKPEDFIFA
>HarmOBP3
MSKFTCFVLCVLAVSLGEVRSNALEKAAIRAAVYPLIVDCAKEHAVTLEQLKAAKASHSAEGINPCFQSCVYKKT
GIFNDNGEYDVANAKTKLQKFVTDEDEYARIAEVGKTCASVNDKSVSDGAAGCERAALLTACFLEHRAQIII
>HarmOBP4
MSKLTCVVFAAVAVVFSNVNADDETRASFRQVLGPLVMECRNEFGITEDDLKKAQQERSPDALKPCFIACVFKK
FGIITSAGKYDSDASISRIKDVVKNDDLLAKLKSVGEKCNSVNDASVSDGDAGCERAALLAKCFIENKSELSI

>HarmOBP5
MSKFTCLVLCVVAASLSQAYASEEEKAAFREAIKPIVEECSKEHGVSHDELKSAKDNQNADNIKPCFLGCVYKKAE
VFNSKGEYDVDKALEKLKKFVSNDEAYAKFAEVGKKCASVNDKAVSDGDAGCERGALLTACFLEHKAEVPL
>HarmOBP6
MSKFTCLLLCVVAVSLSKVHATEEEKEAIRAAVRPIMQECGKEHGVTLDDLKAAKAAHSADGIKPCFQSCVYKKA
GIFNDNGEYDIANAKTKLQKFVTNDEEYARIAEVGKMCASVNDKPVTDGAAGCDRAALLTACFLEHRAQIII
>HarmOBP7
MFRFGVLSFVVLLFCMESSYALSSEEELSIKEALHPFVVECAEEYGMTEEMFEEAKKKGSAEDIDPCFMSCFLKKT
GFFDDSGKFDAEKSISFAKEHITSESAIKFLEAGAGECVKINDEDVSDGENGCDRAKLLFDCLTELKKKMSE
>HarmOBP7.2
MSRFGVLSFVVLVFCMENIYALSSEEELSIKEALHPFVVECAEEYGMTEEMFEEAKKKGSAEDIDPCFMSCFLKKT
GFFDDAGKFDAEKSISFAKEHITSETAIKFLEAGAGECVKINDEDVSDGDKGCDRAKLLFDCLTDLKKKMSE
>HarmOBP8
MLLIEIVKFLTLVAMCEAMTMKQIRNTGKMMRKSCQPKNNVADEQIDPIAEGVFNEDKEVKCYMACIMKMA
NTIKNGKLNYEAAIKQADLLLPDDIKEPAKEAITACRKVADAYKDICDASFHITKCIYTQNPGIFYFP
>HarmOBP9
MCKFSVLFLYSAVMAVNIWSASCISEEDKAAIITAIAPLAQNCGSECGLDNDDFEKYKEDGSDMDPCFKACLMT
QMGVLDKEGKYDGKGLHKAMEEADYPGDKDDAQKFLDELDRCFDAKGDNSGSDEEAKMKRADVLFRCMQ
DMKEK
>HarmOBP9.2
MCKCSVVFLYLAVMAINIWRASCLSEEDKAAIITAIAPLAQNCGSECGLDNDDFEKYKEDGSDMDPCFKACLMT
QMGVLDKEGKYDGKGLHKAMEEADYPGDKDDAQKFLDELDRCFDAKGDNSGSDEEAKMKRADVLFQCMQ
DMKEN
>HarmOBP13
MFTGTLPLVVFLATFAYGGKEKPVFSDEIKEIIQTVHDECVAKTGVAEEDITNCENGIFKEDPKLKCYMFCLMEEA
SLVDDDDAVDYDMLVSLIPEEYVDRTTKMIFSCKHLDTPDKDKCQRAFEVHKCSYEKDPDLYFLF
>HarmOBP18
MKSFVVFCVLVAGAFAANVSLPPKQNEKANQIATECMKESGLKPEVLAEAKKGHISDDEHLKKFTFCFFKKAGIV
SEDGKLNTEVALAKLPPGVDKAEAEKLLETCKGKTGKDVTDTVFEIFKCYHHGTKTHILLGF
>HarmOBP15
MGSRHVFFALVVLAVSVKKEKPSKHPMPYITSRFVKVLEECQHELKLNEHILEHLFHFWKLEYSLLGKDPGCAIIC
MSTKLDLLDLYGRMHRGNAAEFAKKHAAGDEVPSKIVTIIHFCQKKHEQDGDECLQVLEVATCCRTGLHDLNW
QHQVEVIVPDVLTEI
>HarmOBP16
MFKLCVVLAFIVATCHGGTLERTSSTCGQIPRELTACLDLQPAVSPEIQEKCRRANECERLTCVFREYNLLDGAEV
NKERTAAFLDNFVKQYPSWEVAIDVAKTSCLRSSGLKPQGVFLDCPAYDIIQCVFANLVKNALPSQWSSMSQCN
HAREFAAACPICPDACFAPLVPIGTCNACSAARRSS
>HarmOBP17
MRAWSVTLVALLGALGAARAVAMDEDMAELARMVRENCAAETGADVALVERVNAGADLMPDDKLKCYIKC
TMETAGMMADGEVDIEAVLALLPPELAEHNAPSLRACGTVRGADHCDTAFRTQQCWQNANKADYFLI
>HarmOBP18a
MTRQQLKNSGKLMKKSCMPKNDVTEEEVGDIEKGKFIESRNVMCYVACIYTMTQVVKNNKLSYEAVIKQVDM
MFPAEMRDAVKAAATSCKDITKKSKDLCESAYWTAKCMYDYDAENFVFP
>HarmOBP22

MTREQIKNSGKLIKKTCMAKNDLSEDQVKDVDKGKFIEEKPFMCYIACVYKMGQTIKGNTVNHDMMIKQVE
MMFPNEMKAPMKAAIEHCRPVVKKYKDVCEVSYWTAKCIYEFDPPNFMFP
>AipsPBP1
MAPHPSVTMYVRLALVIIAGLFITVECSQEIIKNLSLQFAKPLEDCKKEMDLSDTVITDFYNFWKEGYEFTNRQFG
CAILCLSSKLELLDQDLKLHHGKAQEFAKKHGADEAMAKQLVDMIHSCTQSTPDVADDPCMKTLNVAKCFVAK
IHDLKWAPSMDLIMGEVLAEV
>AipsPBP2
MAASRWCIACLVCVLFAARSVMTSQEVVASFSKGFTNVVEHCKAEVNAGEHIMQDIYNFWREEYQLVNRDLG
CMVLCMANKLGLIGEDQKMHHAKAEEFAKSHGADEAVAKQLVAILYECETKHAAVEDECGMALEIAKCFRTK
MHELKWAPSMEVAMEEIMTAV
>AipsPBP3
MGTYNVFFAFVLMAAGVREIEPSKDAMKYITSGFVKVLEECKQELNMNDRIIADLFHYWKLDYTLLNRDTGCAI
ICMSKKLDLLDDTGRMHHGNAQEFALKHGAGEEVASKIVTIIHDCEKKFERDDDECLRVLEVAKCFRTGIHDLD
WQPKVEVIVSEVFTDM
>AipsGOBP1
MTQPGQVLVLVLLAAAALADVNVMKDVTLGFGQALDKCRQESDLTEEKMEEFFHFWRDDFKFEHRELGCAIQ
CMSRHFNLLTDSSRMHHVNTEEFIQSFPNGEVLARQMVALIHGCEKQFDHEDDHCWRILHVAECFKHACVAH
GVAPSMEMMMTEFIMEAEAR
>AipsGOBP2
MTLRCCLLLVVVAAVTRSVVGTAEVMSHVTAHFGKALEECRDESGLSAEVLEEFQHFWREDFEVVHRELGCAII
CMSNKFSLLQDDSRMHHVNMHDYVKGFPNGEVLSGKLVELIHNCEKQYDTLTDDCDRVVKVAACFKVDAKA
AGIAPEVAMIEAVMEKY
>AipsOBP1
MDISKRRSKNAFRRLLVNTWLRLVQIFTCLSAPPVVSADVTSKCQGSKYENECDKLTCVFRKAKWLDGNAVDKA
KLITYFEQFEKDHPEWAPAMQNVKTSCLGAELKTQGVFLNCPAYDVMHCVLGSFIKHATPTQWSTSASCSYPR
AYAAACPICPEDCFSAQVPFGSCNACYLPPRTP
>AipsOBP2
MSKFTCLVLCVVAASISRVHADDDANKAAFREAFKPILDECSKEHGVSNDDIDAAKKAGSADAIKPCFFGCIYKK
AEVFNAKGEYDVDSALSKLKKFVPDEAKFAKYAEIGKKCASVNEKPVTDGDAGCERGAMLTACFLENRAEMLI
>AipsOBP3
MIRSCRCLVFAAVFQVVLGQGLTGTDSGPPGFQRPQSYVPKHCFAPPPGVDLHTCCPIPQLFPDEDMESCGIQK
LTKEQYENPSPARIPCQESICLLRNANLLKQNNSIDYEKMGDFVDNWAKMDPDFTIPITNAKKVCLIEGGPPAPP
VCEPDRIFTCLTSYVLWNCKLRLDSGEGCKILKEHMDGCRPFLAGP
>AipsOBP4
MFGYQFLSFAAALICFGSSYALTSEEEANIKEAFHPFIMKCAEEYGITEEQFEEAKEKHSAEGIDPCFMSCFMKES
GFFDSAGKFDADKTKEFVDAHLTSERAITFMEAVGSECAKVNDEEVTDGDKGCDRAKLMWGCIQDLKEKMEG
SE
>AipsOBP5
MKYFVLFVALVAGIHANVTLPPEQSEKALKTASECIKETGVSKEVLAEAKKGHIADDEGLKKFTLCFFKKAGIVDN
DGKLNLETALAKLPPGVDKAEAKKVLEGCQAKSGKTPQDTAFEIYKCYHAGAKTHIALAGI
>AipsOBP7
MSKFTCVLCVVALSLSSVYVTRAHKPNLRDAWRSELDECAKEYPVTNDEIDTAVRSGDSSNLNPCFNFCVFNKT
GFFTENGEYDLKNGLIKLRKAIRDDEEYTKFEEVATECTEDKNTSCDEKAKCDSANRLSLCFLRFKDKVRI
>AipsOBP8

MYLRSTNGGVRSFPLGESAYTTKIVEICSKETGLKKQVPPEEKEIKFSQRKGLREFNDCYLAKTGVTTSDGKLNIDE
ALEKLPPGFAKPFVEHCQANIILGYIEENVNDFSTCFHQEVQNHLLSFYGFENYWVMLVLGTSFDKTRFTTLFFD
KHFDFWLAERAGFVNL
>AipsOBP12
MYSGTIFLFSFILLIVSNVTFVSSQMTREQVKNSGKLVKKTCSAKNDLTEDEVKDVDKGKFIEEKKFMCYVACVYK
MGQAVKGNSLNHDMMIRQVDMLFPADMKAPVKAAIEHCRPVAKKYKDICEASYWTAKCVYEFDPPNFMFP
>AipsOBP13
MVLIYIVKFLILVAMCEAMTMKQIRNTGKMMRKSCQPKNNVEDEKIDPIAEGIFIDEPEVKCYMACIMKMANT
LKNGKLNFDAALKQADLLLPDDIKEPAKEAIIACKKAAEGHKDICDVSFHVTKCIYNQNPGIFYFP
>AipsOBP15
MDHNRLCLLVIAMFLATGSDAMTRQQLKNSGKILKKNCMNKHQVTEDQIGTIEKGKFVEDKKVMCYIACIYEL
TSVIKNNKLNYESSLRQIDIMYPADLKESAKAAVENCKDVQKKYKDICEASFHTAKCMYDFKPEDFIFA
>AipsOBP16
MFPGSIPFISGCVHLGVSNYFRSTQSNLVVHYEDDQIVDAIYNCQDENGFDEVLSNSTNLEENFPEKEGLKKSN
DCFLKKTGFVTSDGKLNIDKTLEKLPPSFVKPIVEHCQANIALNYTTESVENFSSCYHDGILNHIFAATEVGIFPFIQ
TWKFFVPGTSFADTILILN
>AipsOBP17
MNQLLVFVLIVACVRISNGMTREQVKKTMTVIKKQCMPKNSVTEDQIGKIEQGVFNEDRNVMCYVACVYKSL
QVVKNERLDLGLISKQIDALYPPELKEPTKKAVSQCINIQDSYNDLCEAVFHSVKCLYEKDPATFIFP
>AipsOBP18
MKTLFVFAACILLAQALTDEQKEKLKKHRTECLTETKVEEALVNKLKGGDYKTESEPLKKYALCMMTKSELMTKD
GKFKKDVALAKVPNAADKPSVEKLIDACLANKGNTPHQTAWNYVKCYHEKDPKHAIFL
>AipsOBP19
MFTGTVPFVLCLVAVAFGGKDKPVFSEEIKEIIQTVHDECVANTGVAEEDITNCENGIFKEDPKLKCYMFCLMEE
ASLVDDDGTVDYDMLVSLIPDEYYERTTKMIFACKHLDTPDKDKCQRAFEVHRCSYEKDPDLYFLF
>AipsOBP21
MLKFSVVCLYFSVAAVNFWNVHCISEDEKKAFIEAMKPMVEECGSDCGLTEEDYKKHSKGEDMDPCFKKCMM
QKLGFLDEDGKYNRKQLHESISEYTGDKDEAKRVQEQLDSCFDANGDNDGDDEESQMKRVDVLFKCLKEIKE
>AipsOBP22
MSMWFRAMVVVGALAAARCGVVMDEDMAELARMVRESCVDETGADVKLVEAVNGGADLMEDDKLKCYI
KCTMETAGMMSDGEVDIEAVMALLPPEMAEHNGPALKSCGTQRGADDCDTAWKTQVCWQNANKAEYFLI
>AipsOBP23
MSKFTYLVLCFVAVSRVYANEDERAAFHEAAKPILVECSKENGVSFDKLKAAKEAGSADGIDPCFFSCVFKKTGVF
NSKGDFDLDNSLTKLKEFVSNDEDYAKVAEVGKKCESVNEKDVSDGEAGCERASLLTACFLEHRAEIPV
>AipsOBP24
MAKLLLAMILTVMTFALTMSATTKDAGTKEAIMTTTVANQDSSIDSNDVDVLAVMNVCNESFRIEMSYIQALN
ESGSFVDETDKTPKCFIRCVFENVGIVSEDGRMFNPARAAVIFAGERNGKPMDDIADMTALCAADRKETCPCD
RSYQFLRCLMSMEIERYEKS
>AipsOBP26
MSKFTCIVLFVVAASLTKVTQAVSEEEKAVAREAMAPILAECSKAEGVSDEDIEEAKKNPSVDAVNSCFIRCVMR
KTDALNEKGLFDSDAALAKIRPFVKSDEDFAKFEEIGKACMSVNDKEVSDGEAGCDRAKLLLACFLEHKAEMLY
>SlitPBP1
MANARWRFVFVVYALYLTSAVLGSQDLMVKMTKGFTRVVDDCKTELNVGDHIMQDMYNYWREDYQLINRD
MGCMLLCMAKKLDLMDDQTMHHGKTEDFAKSHGADDDVAKKLVSVIHECEQQHAGIADDCMRVLEVAKCF

RTKIHELKWAPSIEVIMEEVMTAV

>SlitPBP2

MAFCPSVTMSLRVALVVAASLLVVVQASQDVMKNLAVNFAKPLDDCKKEMDLPDSVTTDFYNFWKEGYELTN

RQTGCAILCLSSKLEILDQELNLHHGRAQEFAMKHGADEAMAKQIVDMIHTCAQSTPDEAADPCMKALNVAK

CFKLKVHELNWAPSVELIVGEVLAEV

>SlitPBP3

MGSRNVFVALVVLTVGMREIEPSKDPMKYIASGFVKVLEECKHELNMNDHLIADLFHYWKLEYTLLNRDTGCAI

ICMGKKLDLLDASGRMHHGNAQEFAKKHGAGDEVASQIVQIIHDCEKKHERDDDECLRVLEVAKCFRTGIHEL

NWQPNVEVIVSEVLTEI

>SlitGOBP1

MLLLLRALPLLAAVLPLRADVNVMKDVTLGFGQALDKCRQESQLTEEKMEEFFHFWREDFKFEHRELGCAIQC

MSRHFNLLTDTSRMHHENTEQFIQSFPNGEVLARQMVELIHACEKQHDHEEDHCWRILHVAECFKQACVQR

GIAPSMEIMITEFIMEAEAR

>SlitGOBP2

MTSKCCLLLVLMAAATSSVMGTAEVMSHVTAHFGKALEECREESGLSAEVLEEFQHFWREDFEVVHRELGCAII

CMSNKFSLLQDDSRMHHVNMHDYVKSFPNGHVLSEKLVGLIHNCEKQFDSMTDDCERVVKVAACFKVDAKA

AGIAPEVAMIEAVMEKY

>SlitOBP3

MWMQALVLTLATLATLAAAAVEMDEDMAELARMVRDNCAGETGVDVALVEKVNAGAELMPDDKLKCYIKC

TMETAGMMADGEVDIEAVLALLPPSLAEHNAPALRACGTQRGADHCDTAFRTQQCWQNANKADYFLI

>SlitOBP4

MTKVLFAIVLTMITFAVVLSASTKEAMTTTMSDQVNSIDVDVLAVMDMCNDSYRIDPTYLQALNESGSFIDETD

KTPKCFIRCVFENVGIVSEDGKQFNPARAAVIFAGERNGKPMEDIADMTALCATDRQETCPCDRSYKFLRCLMS

MEIERYEKS

>SlitOBP5

MSVVRCSSLLVAIFCFVSVNAISGDEEAGIKDALRPFVQECADEFGITEEQFEEAKKKASAADIDPCFMSCFLKKA

EFFDSQGKFDVDSTMAFAKEHLTSEPAMKFVEAVGDECVKINDEDVSDGDKGCDRAKLLFECIAETKKKME

>SlitOBP6

MSKFTCLVLCVVAVSLSGVHATAEEKAAFIEAVKPYVQECSKEHGVTPEDIKSAKAAGNADGINSCFLSCVYKKA

EVITEKGEYDADKALEKLKKFVSNEDDYAKFANIGKKCASVNEKSVSDGEAGCERAALLTSCFLEHKSEISA

>SlitOBP7

MDQKRICLFVIAMFLASGSDAMSRQQLKNSGKMLKKNCMNKIGVTEDQIGSIDKGKFIEDRKVMCYIACIYELT

NVIKNNKLNYEASIKQIDLMYPPDVKESAKAAVEKCKDVQKKYKDICEASFYAAKCMYEFKPEDFIFA

>SlitOBP8

MLLTKIVKFFILVATCEAMTMKQIKNTGKMMRKTCQPKNNAEDEKIDPISDGVFIDEKEVKCYMACIMKMANT

IKNGKLNYDAAMKQADLLFPDDIKEPAKEAITACRKVADAHKDICDASFHVTKCIYNHNPGIFYFP

>SlitOBP10

MVRKISGLLCCLCVFGISFSDSAISADSESRCRNPPTAPQKIERVITLCQDEIKLSILREALDVIKEEHTMPAQRRRD

KREVPFTHDEKRIAGCLLQCVYRKVKAVDGYGFPTLEGLVGLYSDGVNERGYFMAVLEASRECLMKNHDKFSRT

VPMDNGRNCDISFDIFECISDRIGEYCGTSGL

>SlitOBP12

MKTLFVFAACILLAQALTDEQKEKLKKHRTECLTETKVDEELVNKLKGGDYKMDNEALKKYALCMMMKSELMT

KDGKFKKDVALAKVPNPADKPTVEKLIDACLANKGNTPHQTAWNYVKCYHEKDPKHAIFL

>SlitOBP13

MITSCLLVLSAVVQVLLAKQPVFESGPPEPWGPPERTSHPGQFQPRVPKRCWVPPQRINVYNCCPIPTLYPDED
MQSCGFEKLSENKPQKPVYRPEGTCKEGYCVMGKFDLLLANNSVDYVKFREYLDNWAESYPEFANAIHIAKEE
CAQDGGPEVPPICEPDKLFLCLTSTIFWNCKLRDGEGCAALQEHMNECKQYYTRVMAPTIKDFEVR
>SlitOBP15
MYSINCFIFSVILIVMFDNCFVYSMTREQIKNSGKLIKKTCSAKNDLTEDEVKDVDKGKFIEKKDFMCYIACVYKM
GQSVKGSTINHDMMLRQVDMMFPNDMKAPVKSAIEHCRPVAKNYKDLCEASYWTAKCIYDFDPANFMFP
>SlitOBP16
MYRFVILSIVLVSALADDIDIRECGRIFHPPPHGCCKANNAVKNKDMLAEELKDCFDGSGPKDPMKCEIDLCIAK
KKGFATDDGKLDIKKFEEVITKEVGSDKDLLDEIKTNCINGDLNNYGPPEFCDFMKIKHCVTLHMMNHCSEWS
DDGNCKVVKELVGKCAKVI
>SlitOBP17
MKTFRLLCCILSIFLFFDQSYGMTRQQLKNSGKLMKKSCMPKNDVTEDEVGDIEKGKFIETRNVMCYIACVYTM
SQVVKNNKLSYEAVIKQVDVMFPAEMRDAVKAAATHCKETTKKYKDLCESSYWTAKCMYDYDAQNFVFP
>SlitOBP18
MFKLCVFLALGFVACHGAPNSSPGTPNANPGTYCGVTPDNIYRCLNNPRVVTPEVSTKCGSQFTECEKMTCIFR
ELKWSKRGAIDKAKVRAYFDQYETEHPEWAQAVQHVKAFCLASELRAQGVFLNCPAYDIMQCVLASFIKHASP
SVWSTATDCAYPKAYAADCPVCPSDCYSPQIPFGSCNACYTQPRTV
>SlitOBP20
MEKILIFTFITLSGFAHARISVMYAHDKLSDLVAQQCLSEMYPKNKRIEIQESDEPCIIFCVLKKFGIISASGVINLDIY
RKRVQIAHQLDQKTSIMDYGGSCMENAEATQHKQDVCKKAKVFNDCTHLYRILLM
>SlitOBP21
MARRQRGAMFTEALPLFVILVAVTHGGKNKPVFSDEIKEIIQTVHDECVAKTGVAEEDITNCENGIFKEDAKLKC
YMFCLLEEASLVDDDDTVDYDMLVSLIPDEYYERTTKMIFACKHLDTPDKDRCQRAFEVHKCSYEKDPDLYFLF
>SlitOBP22
MSKFTCIILCVVAASLTKVSHAAVTEEEKEAFREAMAPIIAECSEEHGVSEADIKAAKESASADNIKPCFLGCVMK
KIEVLDAKGLYDAETGLGKLRKFVKDDDEFAKFEDIAKKCLKVNDESVSDGEAGCDRAKLVLGCFIEHKVEMPF
>SlitOBP23
MAKFSCLVLCVVAASLGSIHVASGESLRESLRPVIVACSQEHGVTDAEIQAAKDAGSPASIKPCFIACVFKKAGFIN
EQGQLDLETGLKNLRQFVKDDEQYKKLEEVAKKCSQVKDKAVSDGAAGCERGVLLAGCFLEHKTSIII
>SlitOBP25
MAKVTCIVLFVVGVSLSSIQADDGKNESEVEIDVNQIIDDCIEEYHIPRRLFLAAAETGSTHALTPCFWSCCFKGV
GVLNSEGQYDIDATLDLSKKIFTDHEYEKVEIIVKKCESVNGAPVSNGNIECEKSVLLADCLFDNAKKHFPNMFG
VDY
>SlitOBP27
MYKFVILCSIFVAASNADVAQTLTKRETKASLKPLSVCCDIPELADEFQLAKCSPRPPGPCEDVQCIFEVSGFLTDR
NTLNKAAYRSHLQKWEKNHPGWTDSIYKAITDCVDNDPRQHLEVPCKAYDVFTCTGIAMLKKCPDTAWKC
>SlitOBP28
MIVRFLLCLYIVEFYGAHARTDQEIKAWFFREGMDCNIEHPISPKEMLELKENKIPDTNNAKCFVACVFKKTGML
DSKGMFDAENSIAMTQKDFANDPNRLESSKKLLEACKKVNDEAVSDGEKGCERSVLLHKCFVETAPQLGIKLP
>SlitOBP29
MWNLLVVFLAICSCVYARRRSSGAEINGLTEEELKMEFTKLIMKCNKDGEVDMTELVQLQNYVVPTKQSTKCVL
ACAYKAAEVMNAKGEYDIDHAYKVAEMMKNGDEKRLVNAKKMADLCVKVNELSVSDGEKGCDRAAMIFKCT
VENAPKFGFKL
>SlitOBP33

MTCSQALALLALVAISQQATTGCKNCIMLGKEEKAMFRAHSDACVAASRVEPRLVDAMLAGELLDEPALRKHV
YCVLLKCKLISKDGKLQKAAVLGKMAARPDAKNATKVLESCADQTGDTPEDLAWNLFRCGYDKKALLFDYMPT
NVASETDNNS
>SlittoPBP1
MANARWRFVFVVYALYLTSAVLGSQDLMAKMTKGFTRVVDDCKTELNVGDHIMQDMYNYWREDYQLINRD
MGCMLLCMAKKLDLMDDQTMHHGKTEDFAKSHGADDDVAKKLVSVIHECEQQHTGIADDCMRVLEVAKCF
RTKIHELKWAPSMEVIMEEVMTAV
>SlittoPBP2
MSLRVALVVAASLLVVVQASQDVMKNLAINFAKPLDDCKKEMDLPDSVTTDFYNFWKEGYELTNRQTGCAILC
LSSKLEILDQELNLHHGRAQEFAMKHGADEAMAKQIVDMIHTCAQSTPDVAADPCMKTLNVAKCFKLKVHEL
NWAPSVELIVGEVLAEV
>SlittoPBP3
MGSRNVFVALVVLTVAMRETEPSKDPMKYIASGFVKVLEECKHELNMNDHLIADLFHYWKLEYTLLNRDTGCA
IICMGKKLDLLDANGRMHHGNAQEFAKKHGAGDEVASQIVQIIHECEKKHERDDDECLRVLEVAKCFRTGIHEL
NWQPNVEVIVSEVLTEI
>SlittoGOBP1
MLLLLALPLLAAVLPLRADVNVMKDVTLGFGQALDKCRQESQLTEEKMEEFFHFWRDDFKFEHRELGCAIQC
MSRHFNLLTDSSRMHHENTEQFIQSFPNGEVLARQMVELIHACEKQHDHEDDHCWRILHVAECFKQACVQR
GIAPSMEMMITEFIMEAEAR
>SlittoGOBP2
MATVTSSVMGTAEVMSHVTAHFGKALEECREESGLSAEVLEEFQHFWRDDFEVVHRELGCAIICMSNKFSLLQ
DDSRMHHVNMHDYVKSFPNGHVLSEKLVGLIHNCEKQFDSMTDDCERVVKVAACFKVDAKAAGIAPEVAMI
EAVMEKY
>SlittoOBP1
MFKLCVFLALGFVACHGASNSNPGTPNANPGTYCGVTPDNIYRCLNNPRVVTPEVSTKCGSQFTECEKMTCIF
RELKWSKRGAIDKAKVRAYFDQYETEHPEWAQAVQHVKAFCLASELRAQGVFLNCPAYDIMQCVLASFIKHAS
PSVWSTATDCAYPKAYAADCPVCPSDCYSPQIPYGSCNACYTQPRTV
>SlittoOBP2
MVRKISGLLCCLCVFGISFSDSAISADSESRCRNPPTAPQKIERVITLCQDEIKLSILREALDVIKEEHTMPAQRRRD
KREVPFTHDEKRIAGCLLQCVYRKVKAVDGYGFPTLEGLVGLYSDGVNERGYFMAVLEASRECLMKNHDKFSRT
VPMDNGRNCDISFDIFECISDRIGEYCGTSGL
>SlittoOBP3
MKSFVVICIVFVVGVCATEKGNKIASECIKESGVKSDVLAEAKKGNLGDDPAFKEFTYCFFKKVGIVGEDGKLNRD
VAIAKLPSGVDKAEAEKLLDSCKSKTGKDAVETVYEIFKCYQHGTKSHIMFAS
>SlittoOBP4
MKTLLVFAACILVAQALTDEQKEKLKKHRTECLTETKVDEQLVNKLKGGDYKMDNEALKKYALCMMMKSELMT
KDGKFKKDVALAKVPNPADKPTVEKLIDACLANKGNTPHQTAWNYVKCYHEKDPKHAIFL
>SlittoOBP5
MTKVLFAIVLTMVTFAVVLSASTKEAMTTTMSDQVNSIDVDVLAVMDMCNDSYRIDPTYLQALNESGSFIDET
DKTPKCFIRCVFENVGIVSEDGKQFNPARAAVIFAGERNGKPMEDIADMTALCATDRQETCPCDRSYKFLRCLM
SMEIERYEKS
>SlittoOBP7
MFTEALPLFVILVAVTHGGKNKPVFSDEIKEIIQTVHDECVAKTGVAEEDITNCENGIFKEDAKLKCYMFCLLEEAS
LVDDDDTVDYDMLVSLIPDEYYERTTKMIFACKHLDTPDKDRCQRAFEVHKCSYEKDPDLYFLF

>SlittoOBP10
MKEGNRYSHERRITNDSGDQLMVINATDDDYSGYGSGNMGEKLLTSVPRPATPSNNINKNNINRTKRNEPLLN
RPDSDQCLSQCVFANLQVVDSKGIPREAELWNKVQSSVTSQQSRSALHDQIQACFQELQSEAEDNGCSYSNKL
ERCLMLRFSDRKVDGKGNAKKSSTEQTG
>SlittoOBP11
MSKFTCLVLCVVAVSLNGVHATAEEKAAFIEAVKPYVQECSKEHGVTPEDIKSAKAAGNADGINSCFLSCVYKKA
EVINEKGEYDVDKALEKLKKFVSNEDDYAKFANIGKKCASVNEKSVSDGEAGCERAALLTSCFLEHKSEISA
>SlittoOBP12
MSVVRCSSFLVALFCFVSVNAMSGDEEAGIKEALRPFVQECADEFGITEEQFEEAKKKASAADIDPCFMSCFLKK
AEFFDSQGKFDVDSTMAFAKEHLTSEPAMKFVEAVGDECVKINDEDVSDGDKGCDRAKLLFECIAETKKKME
>SlittoOBP13
MITSSSLLVLTAVVQVLFAQQPVFESGPPEPWGPPQRPAHRRQFLPRIPKRCWVPPQRINVYNCCPIPTLYPDED
MQSCGFEKTSGNTDQPQKPVFRPEGTCKEGYCVMGKFDLLFANNSVDFVKFREYLDNWAESYPEFANAIRIAK
QECAQDGGPEVPPICEPDKLFLCLTSTIFWNCKLRDGDGCAALQEHMNECKQYYTRQMEPTMKDIEVR
>SlittoOBP14
MDQKRICLFVIAMFLASGSDAMSRQQLKNSGKMLKKNCMNKIGVTDDQVGSIDKGKFIEDRKVMCYIACIYEL
TNVIKNNKLNYEASIKQIDLMYPPDVKESAKAAVEKCKDVQKKYKDICEASFYAAKCMYEYKPEDFIFA
>SlittoOBP15
MFNNCFVYSMTREQIKNSGKLIKKTCSAKNDLTEDEVKDVDKGKFIEKKDFMCYIACVYKMGQTVKGSTINHD
MMLRQVDMMFPNDMKAPVKAAIEHCRPVAKNYKDLCEASYWTAKCIYDFDPANFMFP
>SlittoOBP16
MLAEELKDCFDGSGPKDPMKCEIDLCIAKKKGFATDDGKLDIKKFEEVITKDVGSDKDLLDEIKTNCINGDLNNY
GPPEFCDFIKIKHCVTLHMMNHCSEWSDDGNCKVVKELVGKCAKVI
>SlittoOBP17
MRTFRLLCCILSIFFIFDQSYGMTRQQLKNSGKLMKKSCMPKNDVTEDEVGDIEKGKFIETRNVMCYIACVYTM
SQVVKNNKLSYEAVIKQVDVMFPAEMRDAVKAAATHCKETTKKYKDLCESSYWTAKCMYDYDAQNFVFP
>SlittoOBP18
MILXYTQKLTNMLLTKIVKFFILVATCEAMTMKQIKNTGKMMRKTCQPKNNVEDEKIDPLSDGVFIDEKEVKCY
MACIMKMANTIKNGKLNYDAAMKQADLLFPDDIKEPAKEAITACRKVADAHKDICDASFHVTKCIYNHNPSIFY
FP
>SlittoOBP20
MWVQALVLTLATLVTLVAAAVEMDEDMAELARMVRDNCAGETGVDVALVEQVNAGAELMPDDKLKCYIKCT
METAGMMADGEVDIEAVLALLPPSLAEHNAPALRACGTQRGADHCDTAFRTQQCWQNANKADYFLI
>SlittoOBP22
MNRLLLVYLVVLYAGCSYGMTRAQVKKTMGIIKNQCMPKNSVTEEQVGRIEQGVFIEDRNVMCYVACIYKSLQ
VVKNDKLDMALITKQIDILYPPELKEPVKKSVAACFHSQDNYSDFCEGVFYASKCLYEKDPASFIFP
>SlittoOBP26
MFNSSVFMYCLYFCALTPYLVSAMTAEQKALIHEHFETIGKSCNKDSTMITAEDIANLRAKKIPTGPNAPCFLAC
MMKQIGVMDDNGMVQKETALEMAKAVFDDPEEIKAIEDYLHSCSHINTESVSDGAAGCERAMLAYKCMTEN
ASKFGFDI
>SlittoOBP25
MSKLSCIVFCAVAMRLCVFVASEDANSIFHAPIKPIVIDCAKEYGLSEDDIKKNRGLDGLKNLPPCFIRCVLNKLDII
NDKGQYDADSGIATIKGLMSNNEYLEKISGVLKECESVNEKSVSDGDAGCERALLGAMCYLDHKTIVLA
>SlittoOBP24

MYKIISLVFCIAVCLNRVHGNAEDKIAIMTAVKPFVEECAKKHGVTFEALLTAKASGKIDGVEPCFYSCVYKKTEFL
NSKGEYDVDTALAKLKKYISNDDDYAKLSQVGKRCASVNSKPVGDGEAGCERGVLLTQCFLDHKGSVPM
>SlittoOBP28
MIVRFLLCLYIVEFYGVHVQARTDQEIKAWFFREGMDCNIEHPISXKEMLELKENKIPDTNNAKCFVACVFKKTG
MLDSXGMFDAENSIAMTQKDFANDPNRLESSKKLLETCKKVNDETVSDGEKGCERSVLLHKCFVETAPQLGIKL
P
>SlittoOBP23
MAKLSCLVLCVVAASLGSIHVAKGESLRESLRPVIVACSKEHGVTDAEIQAAKEAGSPASIKPCFIACVFKKAGFIN
EQGQLDLETGLKNLRQFVKDDEQYKQLEGVANKCSPVKDKAVSDGAAGCDRGVHLAGCFLDHKTSIII
>SexiPBP1
MAGAKWQFVCVVFALYLTSAALGSQELMMKMTKGFTKVVDDCKAELNAGEHIMQDMYNYWREDYQLINR
DLGCMILCMAKKLDLMEDQKMHHGKTEEFAKSHGADDEVAKKLVSIIHECEQQHAGIADDCMRVLEISKCFRT
KIHELKWAPNMEVIMEEVMTAV
>SexiPBP2
MAFCRSATMSVRVALVVAASMLVVVQASQDVMKNLAINFAKPLDDCKKEMDLPDSVTTDFYNFWKEGYELT
NRQTGCAILCLSSKLEILDQELNLHHGRAQEFAMKHGADETMAKQIVDMIHTCAQSTPDVAADPCMKTLNVA
KCFKLKIHELNWAPSMELIVGEVLAEV
>SexiPBP3
MGSHNVFVALVLLAVGMRVAEPSKDAMKYITSGFVKVLEECKQELNMNDHIIADLFHFWKLEYALLSRDTGCVI
ICMSKKLDLLDANGRMHHGNAQEFAKRHGAGDDVASKIVQIIHDCEKKHERDDDECLRVLEVAKCFRTGIHDL
DWQPKVEVIVSEVLTEI
>SexiGOBP1
MLFLLRALPLLAAVLPLRADVNVMKDVTLGFGQALDKCRQESQLTEEKMEEFFHFWRDDFKFEHRELGCAIQC
MSRHYNQLTDSSRMHHDNTEQFIKSFPNGEVLARQMVELIHSCEKQYDHEDDHCWRILHVADCFKQGCVQR
GIAPSMEMMMTEFIMEAEAR
>SexiGOBP2
MTAEVMSHVTAHFGKALEECREESGLSAEVLEEFQHFWREDFEVVHRELGCAIICMSNKFSLLQDDTRMHHV
NMHDYVKGFPNGHVLSEKLVELIHNCEKRFDSMTDDCERVVKVAACFKVDAKAAGIAPEVAMIEAVMEKY
>SexiOBP1
MSKFTCLVLCVVAGCLSGVHATAEEKAALIEAVKPYIQECSKEHGVTPEDIKSAKEAGNADGINACFLRCVYNKA
GVINDKGEYDADKALEKLKKFVSNEDDYAKFAEIGKKCASVTETSVSDGEAGCERAALLTSCFLEHKSEVHA
>SexiOBP2
MKSFVVFCIVLVVGVCANEKGNKLDRPFASECIKETGVKNELLEEAKKGIISEDPAFKAFTYCFFKKIGIVGEDGLL
NRDVAIAKLPSGVDKSEAEKLLDSCKSKTGKDAVDTVFEIFKCYQQGTKSHIMFAS
>SexiOBP3
MVKLTCVVFCAVAMALSVFVAGEDANSVFQGAIKPLIAECAKEYKLSDEELLKNRGLAGLSNLPPCFIGCVLKKF
DIINDKGLYDAEAGIAKIEKLLPNNEFLDKISGVLKSCESANEKSVGDGDAGCERAVLVATCYLEHKTAVIA
>SexiOBP4
MWNFLVVFLAICSCVYGLTEEELKMEFTKLIMKCNKDGKVDMTELVQLQNYVVPTKQTTKCVLACAYKAAEVM
NAKGEYDIDHAYKVAEMMKNGDEKRLVNAKKMADLCVKVNEQSVSDGEKGCDRAAMIFKCTVENAPKFGFK
L
>SexiOBP5
MTMKQIRNTGKMMRKTCQPKNNVEDEKIDPIAEGVFIDEKEVKCYMACIMKMANTIKNGKLNYDAAIKQAD
LLLPDDIKEPAKEAITACKKVADAHKDICDASFHITKCIYNHNPGIFYSP

>SexiOBP6
MLGSLLFVFAFSVFSLGAEALLIDDLKQKYADSILQCSQQYPLDRADAELLQNKVMPDKESTKCLFACVYKVTGV
MSDQGELSVEGVNALSQKYLADDPEKLKKSEEFTEACRTVNDAPVSDGARGCDRAALIFKCTIEKSPDFSFV
>SexiABP1
MSVVRYSSFVVALFCLVSVNAMSGDEEAGVRDALRPYVQECADEYGITEEQFEEAKKKASADDIDPCFMSCFLK
KAEFFDAQGKFDVDSTMAFAKEHLSSEPAMKFVEAVGDECVKINDEDVSDGDKGCDRAKLLFDCIAETKKKM
D
>SexiOBP10
MDRKRICLFVIAMFLASGSDAMSRQQLKNSGKMLKKNCMNKIGVTEDQVGSIDKGKFIEDRKVMCYIACIYEL
TNVIKNNKLNYEASIKQIDLMYPPDIKESAKAAVEKCKDVQKKYKDICEVSFYAAKCMYEFKPEDFIFA
>SexiOBP8
MARRQQGAMFTETLPLFVILVAVTHGGKDKPVFSDEIKEIIQTVHDECVAKTGVAEEDITNCENGIFKEDAKLKC
YMFCLLEEASLVDDDDTVDYDMLVSLIPDEYYERTTKMIFACKHLDTPDKDRCQRAFEVHKCSYEKDPDLYFLF
>SexiOBP9
MKTLFVFAACILLAQALTDEQKEKLKKHRTECLSETKVDEQLVNKLKGGDYKTESEPLKKYALCMMMKSELMTK
EGKFKKDVALAKVPNPADKPTVEKLIDACLANKGNTPHQTAWNYVKCYHEKDPKHAIFL
>BmanPBP1
MSIKGQIALALMVYMAVGSVDASQEVMKNLSLNFGKALDECKKEMTLTDAINEDFYNFWKEGYEIKNRETGC
AIMCLSTKLNMLDPEGNLHHGNAMEFAKKHGADETMAQQLIDIVHGCEKSTPANDDKCIWTLGVATCFKAEI
HKLNWAPSMDVAVGEILAEV
>BmanPBP3
MARYNIVVAVLVLGVVGARGSSEAMRHIATGFIRVLDECKQELGLTDHILTDMYHFWKLDYSMMTRETGCAIIC
MSKKLDLIDGDGKLHHGNAQAYALKHGAATEVAAKLVEVIHGCEKLHESIDDQCSRVLEVAKCFRTGVHELHW
APKLDVIVGEVMTEI
>HassPBP1
MNFAKPLEDCKKEMDLPDSVTTDFYNFWKEGYEFTNRQTGCAILCLSSKLELLDQEMKLHHGKAQEFAKKHG
ADDAMAKQLVDLIHGCSRSTPDVTDDPCMKALNVAKCFKAKIHELNWAPSMDLVVGEVLAEV
>HassPBP2
MMGSAMSSKELLTKMSEGFTKVVDACKTQLNVGDHITQDMYNFWREEYQLVNRDLGCMIMCMVAKLDLIG
DDQKMHHGKAEEFAKSHGADDVLAKQLVSLIHSCETQHQAIEDHCSRVLEIAKCFRTKIHELKWAPSMEVVME
EIMTAA
>HassPBP3
MGSRHVFFAFAVLAVSVRKAEPSKDAMQYITSGFVKVLEECKHELNLNEQILADLFHFWKLEYSLLGRDTGCAII
CMSKKLDLLDANGRMHHGNAAEFAKKHGAGDEVASKIVTIIHECEKKHEQDGDECLRVLEVAKCFRTGIHELD
WQPKVEVIVSEVLTEI
>HassGOBP1
MDINVMKDVTLGFGQALDKCREESQLTEEKMEEFFHFWSDDFKFEYRELGCAIQCMSRHFNLLTDSSRMHHD
NTEKFIQSFPNGEVLARQMVELIHSCEKQFDHEDDHCWRILHVAECFKGSCVQRGIAPSMELMMAEFIMEAE
SL
>HassGOBP2
MTSKSCLLLVAMATLTASVMGTAEVMSHVTAHFGKALEECREESGLSAEVLEEFQHFWREDFEVVHRELGCAII
CMSNKFSLLQDDSRMHHVNMHDYIKSFPNGHVLSEKLVELIHNCEKKYDTMTDDCDRVVKVAACFKVDAKA
AGIAPEVAMIEAVMEKY
>HassOBP3a

MSKFTCFVLCVLAVSLAEVRSNALEKAAIRAALYPLIVDCAKEHSVTLEQLKAAKAAHSAQGINPCFQSCVYKKT
GIFNDNGEYDIANAKTKLQKFVTDEDEYARIAEVGKTCASVNDKSVSDGAAGCERAALLTACFLEHRAQIII
>HassOBP17
MKTFVILAACVMLVQASGLTDEQKEKLKKHRSECLTETKVDEQLVNKLKGGDYKTESEPLRKYALCMMMKSEL
MTKDGKFKKDVALAKVPNAADKPTVEKLIDACLANKGNTPHQTAWNYVKCYHEKDPKHAIFL
>HassOBP7
MSRFCVLSFVVLLFCMENIYALSSEEELSIKEALHPFVVECAEEYGMTEEMFEEAKKKGSAEDIDPCFMSCFLKKT
GFFDDAGKFDAEKSISFAKEHITSESAIKFLVAGAGECVQINDEDVSDGDKGCDRAKLLFDCLTDLKKKLSE
>HassOBP13
MFTGTLPLVVFLATFAYGGKEKPVFSDEIKEIIQTVHDECVAKTGVAEEDITNCENGIFKEDPKLKCYMFCLMEEA
SLVDDDDAVDYDMLVSLIPEEYVDRTTKMIFSCKHLDTPEKDRCQRAFEVHKCSYEKDPDLYFLF
>HassOBP1
MCKLTCLVLAAVAVVFSNVNADDEPRASFRQMLGPFVMECKKEFDITEDDLKKAQQEHSPDALKPCFIACVFK
KFGIITSAGKYDSDASISRIKDVVKNDDLFAKLKSVTEKCNSVNDASVSDDDAGCERAALLAKCFIENKSEISFN
>HassOBP2
MLNLFIVVLALCSSVAVYALTEEELKLKFTKLIMKCNKDSEVDMQELVQLQSYVVPTKTATKCVLACAYKAASVM
NAQGLYDIDHAYKVAEMMKNGDEKRLTNAKKMTDICVKVNDIKVSDGEKGCDRAALIFKCTVDNAPKFGFKL
>HassOBP3
MSRQQLKNSGKMLKKNCMSKNQVTEDQIGSIEKGKFVEDKKVMCYIACIFEMTNVIKNNKLNYDASIKQIDL
MYPPDLKESAKAAAEKCKDVQKKYKDICEASYWTAKCLYDFKPEDFIFA
>HassOBP4
MSKFTCLLLCVVAVSLSKVHATEEEKEAIRAAVRPIMEACGKEHGVTLDDLKAAKAAHSADGIKPCFQSCVYKKA
GIFNDNGEYDIANAKTKLQKFVTNDEEYARIAAVGKTCASVNDKPVTDGAAGCDRAALLTACFLEHRAEIII
>HassOBP5
MSKFSCLVLCVVAASLSQAYASEEEKAAFREAIKPIVEECSKEHGVSHDELKSAKDNQNADNIKPCFLGCVYKKAE
VFNSKGEYDVDKALEKLKKFVSNDEAYAKFAEVGKKCASVNDKAVSDGDAGCERGALLTACFLEHKAEVPL
>HassOBP9
MCKFSVLFLYSAVMAVNIWSASCISEEDKAAIITAIAPLAQNCGSECGLDNDDFEKYKEDGSDMDPCFKACLMT
QMGVLDKEGKYDGKGLHKAMEEADYPGDKDDAQKFLDELDRCFDAKGDNSGSDEEAKMKRADVLFQCMQ
DMKEK
>HassOBP16
MFKSIVFCALVIVASHADVLKKRDSKGASLKPLSVCCDIPELGDPKNLEKCSNPKMPGPCDDIQCISEASGFLIDR
NTLNVDAYKAHLTKWEEEHKSWKVAVDRAIEECANNQTRQYLDFPCKAYDVFTCTGIAMLKKCPEAAWKC
>MsexPBP1
MKVAVVAIVVYLAVGNVDSSPEVMKNLCLNFGKALDECKAEMNLSDSIKDDFANFWVEGYEVSNRDTGCAIL
CLSKKLDMIDPDGKLHHGNAMEFAKKHGADEAMAKQLLDIIHNCENSTPPNDDACLKTLDIAKCFKKEIHKLN
WAPNMDLVVGEVLAEV
>MsexPBP2
MVSTKWRLLVVTIAVLTMEMVSASQEVLKQMSVGFSKVLQTCKTELSVGDHIIQDFYNYWREDYDLLNRDFGC
MVICMAVKHDLINDQLTMHHGNAHAFAKTHGADDDTAQQLVTILRECEAKHQSVEDVCNRALEMAKCFRTK
IHELKWAPAMEVVLEEIMTSV
>MsexPBP3
MAIIPIFTVLLMMTAVKEIAPSSDAMRHIANGFLKVLDQCKHELGLTDQIVVDLYQFWKLQYALLNRDTGCAIIC
MSKKLDLLDSTGRMHHGNTQEFAVSHGATDEVASKVVVIIRDCEKQQEGEEDDCVRVLEVAKCFRTAIHELNW

APNMEVVVDELLTER
>MsexGOBP1
MGQDTRSLVLVVLVGLVGAVSADVQVMKDVTLGFGQALEQCREESQLTEEKMEEFFHFWREDFKFEHRELGC
ALQCMSRHFNLLTDSSRMHHENTDKFIKSFPNGAVLSKTMVELIHNCELQHDAEEDHCWRILRVAECFKISCTK
AGIAPSMEVMMAEFIMELKQ
>MsexGOBP2
MVNRLILMVVVVFITDSVMGTAEVMSHVTAHFGKALEECREESGLPVEVMDEFKHFWREDFEVVHRELGCAII
CMSNKFELLQDDTRIHHVKMHDYIKSFPNGQVLSEKMVQLIHNCEKQYDDIADDCDRVVKVAACFKKDAKKE
GIAPEVAMIEAVIEKY
>MsexABP1
MSVISFFVLCFGVLAVSVGAVSENERNQISQSILPHIVKCSQEYGVSEGQIKDAKESVNPLGLNPCFLGCVLKSAGI
IDKNGLFDVEATKEKSKKYISSEKDVTNFDKIIKDCTEVNQKNVSDGNKGCDRAKELVTCFLAKRGDFSVFTF
>MsexABP2
MDRKDLCLLIIAFILADGVDSMSKQQLKNSGKMFKKQCMGKNKVTEDEIGEIDKGRFVEQQNVMCYIACIYQ
MSQVVKNNKLNYEASLKQIDIMYPPELKDTAKGALEACKDIAKKNKDLCEASFKTAKCMYEYSPKDFLFP
>MsexABP3
MITATLHVVFALLGFVYGAKNKPVFSEEIKEIIQTVHDECVGKTGVSEEDIANCENGIFKEDVKLKCYMFCLLEVA
GLADEDGTVDYDMLVSLIPEEYSERASKMIFACNHLDTPEKDKCQRSFDVHKCTYEKDPEFYFLF
>MsexABP4
MFRYIFCAICLILILFDASYAMSRQQLKNSGKMMKKSCIPKNDVTEDEVGQIEQGKFIEDRRVMCYIACIYTMTQ
VVKNNKLSYDAIVKQVDMMFPPEMRTAVKTAAENCKDIAKKYKDICEASYWTAKCMYDFDSKNFVFP
>MsexABP6
MKVTNGLFLVFLVSVLACGIAASPKKIYRIPPQASEKIVEEVLKCVQKMGLDSTVVNLLKEGKYTEDDRVIETLMC
SNQNVGNVNGDGKVNIDKVMNDIFSNKPEIRSALVACEKDGGKSPLETFKNFILCFKEKVPVKVML
>MsexABP7
MQACLFLTLVLAVVGLNAHNVHLTDGQKEKANEPIAACIKETGIKPEVIAEAKKGHYSEDEAMKKFILCFFHKAGI
VNADGKLNLDVAIAKLPPGVDKTEATKTLEGCKDNGGKDAADPAFAIFKCYKDATKTHVLF
>MsexABP8
MKALLVLAACLVLAQALTDEQKEKLKKHKSECLSETKVEEQLVNKLKAGDYKAENDNLKKYALCMMMKSELMT
KEGKFKKDVALSKVPNPADKPMVEKLIDTCLANKGNTPHQTAWNYVKCYHEKDPKHAIFL
>MsexABPX
MISSLVHVLTLLAAGALALDEEQAELARMVRENCVDEIGVDEGLLAKVDDGADLMPDPKLKCYLKCTMEMAG
MISDGVVDVEAVLGLLPDDVKLRTTDIVRACDTQKGADDCDTAFLTQTCWQQANRADYIFI
>MsexOBP10
MIRAVVFCCCMVALMPFSANAMTDEQKEKIHEHFEKLGLGCLKEYTITEDDIKDLRAKKVPSGENAGCFLACM
MKEIGVLNDEGMLEKGRAMELAKEVFDDAEELKKIEESMHSCSSVNSESVGDGEKGCERAMLAYKCMVENAS
KFGFDI
>HvirPBP1
MMSVRLMLVVAVWLCLRVDASQDVMKNLSMNFAKPLEDCKKEMDLPDSVTTDFYNFWKEGYEFTNRHTGC
AILCLSSKLELLDQEMKLHHGKAQEFAKKHGADDAMAKQLVDMIHGCSQSTPDATDDPCMKALNVAKCFKAK
IHELNWAPSMELVVGEVLAEV
>HvirPBP2
MMGSAMSSKELLTKMTGGFTKVVDHCKTELNVGDHIMQDMYNFWREEYQLVNRDLGCMIMCMTAKLDLV
GDDQKMHHGKAEEFAKSHGADDALAKQLVGLIHGCETQHQAIEDHCSRTLEVAKCFRTKIHELKWAPSMEVI

MEEIMTAA
>HvirGOBP1
MPGVLRALLLLAAAAPLLADVNVMKDVTLGFGQALDKCREESQLTEEKMEEFFHFWRDDFKFEHRELGCAIQC
MSRHFNLLTDSSRMHHDNTEKFIQSFPNGEVLARQMVELIHSCEKQFDHEEDHCWRISHLADCFKSSCVQRGI
APSMELMMTEFIMEAEAR
>HvirGOBP2
MTSKSCLLLVAMVTLTTSVMGTAEVMSHVTAHFGKALEECREESGLSAEVLEEFQHFWREDFEVVHRELGCAII
CMSNKFSLLQDDSRMHHVNMHDYVKSFPNGHVLSEKLVELIHNCEKKYDTMTDDCDRVVKVAACFKVDAKA
AGIAPEVTMIEAVMEKY
>HvirABP1
MSKFSCLVLCVVAASFSQAFASEEEKTAFREAIRPIVEECSKEHGVSHDELKSAQENQNADNIKPCFLGCVYKKSE
VFNSKGEYDVDKALEKLKGFVSNEAAYAKFAEVGKKCVTVNDKPVSDGAAGCERGAMLTACFLEHKAEVPL
>HvirABP2
MSRFCLLSFVVMIIYLGSIHALSSDEESSIKEALHPFVVECAEEYGITEEMFEEAKKKGSAEDIDPCFMSCFLKKAEF
FDGAGKFDVEKTMSFAKSHITSEPAIKFLEAAGGACVKINDEDVSDGDQGCDRAKLLFDCLMELKKKISE
>HvirABPX
MCARSLTLVTLLAALGAARAVAMDEDMAELARMVRENCAAETGADVALVERVNAGADLMPDDKLKCYIKCT
METAGMMADGEVDIEAVLALLPPELAEHNAPSLRACGTVRGADHCDTAFRTQQCWQNANKADYFLI
>HvirOBP2
MMGSAMSSKELLTKMTGGFTKVVDHCKTELNVGDHIMQDMYNFWREEYQLVNRDLGCMIMCMTAKLDLV
GDDQKMHHGKAEEFAKSHGADDALAKQLVGLIHGCETQHQAIEDHCSRTLEVAKCFRTKIHELKWAPSMEVI
MEEIMTAA
>HvirOBP0021
MDRKKMCLLIIAMFLAIGCDAMSRQQLKNSGKMLKKNCMNKNQVTEDQIGTIDKGKFVEDKKVMCYIACIYE
MTNVIKNNKLNYDASMKQIDLMYPPDVKDSAKAAVEKCKDVQKKYKDICEASFWTAKCMYDFKPEDFIFA
>MbraPBP1
MTYSKWRLSCLVCVIFVASSVMASKELITKMSSGFTKVVDQCKNELNVGEHIMQDMYNFWREEYELLNRDLG
CMVMCMANKLDLIGEDQKMHHGKAADFAKSHGADDDQAKQLVGIVHDCENTHQGVEDACSRALEVAKCF
RSKMHELKWAPSMEVIMEEIMTAV
>MbraPBP2
MALHRSTTMSVRLALVAIASLFITVECSQEIMKNLSMNFAKPLEDCKKEMDLPDSVTTDFYNFWKEGYEFTNR
QTGCAILCLSSKLELLDQELKLHHGKAQEFAQKHGADEAMAKQLVDLIHGCTQSTPDVAADPCMKALNVAMC
SKTKVHELNWAPSVELIVGEVLAEV
>MbraGOBP2
MTSKSSLFLVVLATVTSSVMGTAEVMSHVTAHFGKALEECREESGLSAEVLEEFQHFWREDFEVVHRELGCAIIC
MSNKFSLLQDDSRMHHVNMHDYVKSFPNGEVLSGTLVELIHNCEKKYDGMTDDCDRVVKVAACFKVDAKAA
GIAPEVAMIEAVMEKY
>AperPBP1
MLGKISLLLLPVFVAINLVHSSPEIIKNLSQNFCKAMDQCKQELNIPDSVIADLYNFWKDDYVMTDRLAGCAINC
MATKLDVVDPDGNLHHGNAKEFAMKHGADASMAQQLVDIIHGCEKSAPPNDDKCMKTIDVAMCFKKEIHKL
NWVPDMDVVLGEVLAEV
>AperPBP2
MIRKVLLSVLLAVLMTINLGQASPEVMKNLCMNYGKAMDQCKQELNLPDSVIADLYNFWKDDYVMTDRLAG
CAINCLSTKLDIVDPDGNLHHGNAKEFAMKHGADDGMAHELVDIIHGCEKSSPPNDDKCIKTMDIAMCFKKEI

HKLNWVPNMDLVVGEVLAEV
>AperPBP3
MIAKTFNLLVIVYLSTNTAVDSSQDVMKSMTLTFTKGLDACKKEMDLPDTIDVDFNNFWKEDYVVTNRNAGCA
IMCLASKVDLVDSMGILIHGSSHEFAKQHGADDNMAKQLSDTLHSCEKTIGTLNDECLRALNVANCFKVEIHKL
DWAPSMDLIIGEILAEI
>AperGOBP1
MAHTLQTVVLLLGTSILHPILADVNVMKDVTLGFGQALEKCREESQLTEEKMEEFFHFWSEDFKFEHRELGCAIL
CMSRHFNLLTDSSRMHHENTDKFIKSFPNNEVLSKHMVNLIHSCEQQHDADLDHCWRILRVAECFKRSCQEA
GVAPSMELLMAEFIMESEIN
>AperGOBP2
MGYKLLLMYIAIVIDSVIGTAEVMSHVTAHFGKALEECRDESGLSPEILNEFKHFWSEDFDVVHRELGCAIICMS
NKFSLLKDDTRIHHVNMHDYVKSFPNGEVLSAKMVNLIHNCEKQYDDITDECDRVVKVAACFKVDAKKEGIAP
EVAMIEAVIEKY
>ApolPBP1
MLRKISLLLLPVFVAINLVHSSPEIMKNLSNNFGKAMDQCKDELSLPDSVVADLYNFWKDDYVMTDRLAGCAI
NCLATKLDVVDPDGNLHHGNAKDFAMKHGADETMAQQLVDIIHGCEKSAPPNDDKCMKTIDVAMCFKKEIH
KLNWVPNMDLVIGEVLAEV
>ApolPBP2
MSLSLFLAMLVTINLVQASPEIMKNLCMNYGKTMDQCKQELGLPDSVINDLYNFWKDDYVMTDRLAGCAINC
LSTKLDIVDPDGNLHHGNAKEFAMKHGADDGMAQQLVDIIHRCEKSTPPNDDKCTKTMDIAMCFKKEIHKLN
WVPNMDLVVCEVLAVV
>ApolPBP3
MIAKTFNLLVIVYLSTNTGVDASQEIMKTMTLTFTKGLDACKKEMDLPDTVDVDFNNFWKEDYVVTNRDAGC
AIVCLASKINLVDSMGILIHGSAHEFAKQHGADDNMAKQLSDTLHTCETIIGTGNDECTRALHVANCFKVEMHK
LDWAPSMDLIIGELLAEI
>AsegGOBP1
MTHPGQALVLVLLAAAALADVSVMKDVTLGFGQALDKCRQESDLTEEKMEEFFHFWRDDFKFEHRELGCAIQ
CMSRHFNLLTDSSRMHHDNTEQFIQSFPNGEVLARQMVSLIHGCEKQFDHEEDHCWRILHVAECFKHACVAT
GVAPSMEMMMTEFIMEAEAR
>AsegGOBP2
MTLKCCLLLVVVAAVTRSAVGTAEVMSHVTAHFGKALEECRDESGLSAEVLEEFQHFWREDFEVVHRELGCAII
CMSNKFSLLQDDSRMHHVNMHDYVKGFPNGEVLSGKLVELIHNCEKQYDTLTDDCDRVVKVAACFKVDAKA
AGIAPEVAMIEAVMEKY
>AsegPBP1
MSVRLALFMIAGVFITVECSQEIMKNLSLQFAKPLEDCKKEMELSETVITDFYNFWKEGYEFTNRQFGCAILCLS
SKLELLDQDLKLHHGKAQEFAKKHGADEAMAKQLVDMIHGCSQSTPDVADDPCMKTLNVAKCFVAKIHDLK
WAPSMDLIMGEVLAEV
>AsegPBP2
SQKVVASFSKGFKDVVDHCKAELNMGEHIMQDVYNFWREEYQLVNRDFGCMVLCTANKLGLLKEDQKMHH
DKAEEYAKKHGADDATAKQVVAIIFECENNNSGMDDECNRALEIAKCFRTKMHELKWAPSVEDAIEEIMTAVM
>AsegPBP3
MGTYNVFFAFVLMAAGVREIEPSKDAMKYITSGFVKVLEECKQELNMNDRIIADLFHYWKLDYTLLNRDTGCAI
ICMSKKLDLLDDTGRMHHGNAQEFALKHGAGEEVASKIVTIIHDCEKKFERDDDECLRVLEVAKCFRTGIHDLD
WQPRVEVIVSEVLTDM

>AsegOBP2
MSKFTCLVLCLVAASISRVYADEDANKAAFREAIKPIIDECSKEHGVSNDDIDAAKKAGSADAIKPCFFGCIYKKAE
VFNAKGEYDVDNALSKLKKFVPDEAKFAKYAEIGKKCASVNEKPVTDGEAGCERGAMLTACFLENRAEILI
>AsegOBP3
MIRSCRCLVFAAVFQVVLGQGLTGTDSGPPGFQRPQSYVPKHCFAPPPGVDLHSCCPIPQLFPDEDMESCGIQK
LTKEQYDNPSPARVPCKESFCLLRNANLLKANNNSIDYAKMEDFVDSWAKMDPDFTMPITNAKKVCLIEGGPP
SPPVCEPDRVFTCLTSYVLWNCKLKLEKGEGCKILKEHMDGCRPFLAGP
>AsegOBP4
MCSCYSPCFMFGFQVCGILFFHHSPPSIFSLRSCIQPHINFALSHPLSPSLTSSSFTLAHSEPTASMNAIARSLVKW
ASTNSLVLSTSNLPALSKNPDSFIKQLMKQGSIPSALCFSFASSNCSSVIPYSSAHFTTNG
>AsegOBP5
MKFLVICVALVAGIHANVTLPPEQSEKALKTASECIKETEVSSEVLAEAKKGNIPEDDGLKKFALCFFKKAGIVDND
GKLILETAVAKLPPGVDKVEAKKVLEGCQSKSGKTPQETAFEIYKCYHAGAKTHIALAGI
>AsegOBP6
MFKYSVFVLFVVASTQADVLSQRENKGASLKPLSVCCDIPELGDPKHLAKCSNPKLPGPCNDVQCVFEESGFLTD
KNTLNKEAYRHHLKQWEENNKGWTIAVDKAIKECVDSDPRQHLDFPCKAYDVFTCTGIAMLKKCPDSAWKC
>AsegOBP8
MYLRSASKGVHSFPYGESTYTTKIVEFCSKETGITREVPPEEKGVKFSERKGLREFNDCYLEKTGFVTCDGTLNIDE
SLAKLPPGFAKPFVEHCQANIILKYIQDNVNDFSTCFHQEVQNHFLSFYGFTNYWVMLVLGTSFEKTRFTTLFYD
KNFDFWLAERAGFVEL
>AsegOBP9
MFHLYFYVFICGVLSFNVKAASLDELKMKYVEMIIECSDTYPITAADTLQLKTKTMPDNESIRCLFACVYKKAGM
MNEQGELSVEGVNEMTRRYLSDDPDKIKKSEQFTEACKSVNDVPVSDGTRGCDRAALIFKCTVEKSPDFDLL
>AsegOBP13
MVLIDIVKFLILVAMCEAMTMKQIRNTGKMMRKSCQPKNNVEDEKIDPIAEGIFIDETEVKCYMACIMKMANT
LKNGKLNFDAALKQADLLLPDEVKEPAKEAIIACKKAAEGHKDICDVSFHVTKCIYNQNPGIFYFP
>HzeaPBP
MMSVKLALVVAAWLFIRVDASQDVIKNLSMNFAKPLEDCKKEMDLPDSVTTDFYNFWKEGYEFTNRQTGCAI
LCLSSKLELLDQELKLHHGKAQEFAKKHGADDAMAKQLVDLIHGCAQSTPEVVDDPCMKTLNVAKCFKAKIHEL
NWAPSMDLVVGEVLAEV
>HzeaGOBP2
MTSKSCLLLVAMATLTASVMGTAEVMSHVTAHFGKALEECREESGLSAEVLEEFQHFWREDFEVVHRELGCAII
CMSNKFSLLQDDSRMHHVNMHDYVKSFPNGHVLSEKLVELIHNCEKKYDTMTDDCDRVVKVAACFKVDAKA
AGIAPEVAMIEAVMEKY
>OnubPBP1
MGLSLRLLVVVAAAIFGAESSQDVMKQMTINFGKALDTCRKELDLPDSINADFYNFWKEGYELSNRQTGCAIM
CLSSKLDLVDPEGKLHHGNTHEFAKKHGADDSMAKQLVELIHKCEGSVADDPDACMKVLDIAKCFKAEIHKLN
WAPSMDLIVAEVLAEV
>OnubPBP2
MWLSKTPVVIAVMCSMSVVVHSSQAVMKDMTKNFIKAYEVCAKEYNLPEAAGAELMNFWKEGYVVTSREA
GCAILCLSSKLNLLDPEGTLHRGNTVEFAKQHGSDDAMAHQLVDIVHACEKSVPPNEDNCLMALGISMCFKTEI
HKLNWAPNHELMLEEMMAEMKQ
>OnubPBP3
MWLPKTLVVMAVMSSMSVVVHSSQTVMREMTRNFIKAYEVCAKEYNLPEATGSELINFWKEGHELTTREAG

CAILCMSTKLNLLDVQGSVHRGNTVEFAKHHGSDDAMAHQVVDILHACEKATPNEDKCMLALSIAMCFKAEI
HKLDWAPNHELMFEELVSDMWNS
>OnubPBP4
MADATKWRVAAILVICFAVKLNTVMSSEEVMTKMGVTFFNVLEECKKELKVTTNINEGLVRFWSQGAAPEREL
GCVFLCMAHKKDLLEDQKRLHHENAHQFARGHGADDDKATEIVSLLRECEQQFITITDDCSRALEVARCFQAH
MQRLQWAPSMEVMVEEILAGMA
>OnubPBP5
MKGFAGIPVTLMLVLIGVSEIEMVPEAMKQLTGGFLKVLDQCKKELNLSDGVISDLYHLWKEEYDQISRDAGCVI
HCMSQKLELVGGDGKMHHVNIKDFALKHGAGDEIATQLVTLAHECEKQKASIEDDCERTLEMSKCFRSDVKQV
DWTPKMEVIITEVIEV
>OfurPBP1
MGLSLRLLVVVAAAIFGAESSQDVMKQMTINFGKALDTCRKELDLPDSINADFYNFWKEGYELSNRHTGCAIM
CLSSKLDLVDPEGKLHHGNTHEFAKKHGADDSMAKQLVELIHKCEGSVADDPDACMKVLNIAKCFKAEIHKLN
WAPSMDLIVAEVLAEV
>OfurPBP2
MWLSKTLVVIAVMCSMSVVVHSSQAVMKDMTKNFIKAYEVCAKEYNLPEAAGAEVMNFWKEGYVLTSREAG
CAILCLSSKLNLLDPEGTLHRGNTVEFAKQHGSDDAMAHQLVDIVHACEKSVPPNEDNCLMALGISMCFKTEIH
KLNWAPDHELLLEEMMAEMKQ
>OfurPBP3
MWLPKTLVVMSVMSSMSVVVHSSQTVMGEMTKNFIKAYEVCAKELNLSEATGLQLINFWKEGHELTTRETGC
AILCMSTELNLLDVQGSVHRGNTVEFAKHHGSDDAMAHQVVDILHACEKATPNEDKCMLALSIAMCFKAEIH
KLDWAPNNELMFEELVLDMWNS
>OfurPBP4
MADATKWRVAAILVICFTVNLNTVMSSEELMTKMGVTFFNVLEECKKELKVTTNINEGLVRFWSQGAAPEREL
GCVFLCMAHKKDLLEDQKRIHHENAHQFARGHGAEDDKATEIVSLLRECEQQFITITDDCLRALEVARCFQAH
MQRLQWAPSMEVMVEEILAGMA
>OfurPBP5
MKGFAGVPVTLMLVLIGVSEIEMVPEAMKQLTGGFLKVLDQCKKELNLSDGVISDLYHLWKEEYDQISRDAGCV
IHCMSQKLELLGGDGRMHHVNIKDFALKHGAGDEIATQLVTLAHECEKQKAAIEDDCERTLEMSKCFRSDVKQ
VDWTPKMEVIITEVIEV
>OfurGOBP2
MVCSGFYLGLVVMAAVTSVKGTAEVMSHVTAHFGKALEECRTESGLSPEILEEFQHFWSEDFEVVHRELGCALI
CMSNKLSLLQDDTRIHHVNMHDYVKGFPNGEVLSEKMVNLLHNCEKQFDDITDDCQRTVKVAACFKVDAKKE
GIAPEVAMIEAVMERY
>PxylABP
MKFLVVFAICLVAAQALTDEQKEKLKKHKTECLAETKPEVEHVDKLKNGDYTTENEALKKYAHCMMIKSELMTK
DGKFRKDVALAKVPNPADKPMVEKLIDTCLANKGDTPQQTAWNYVKCYHEKDPKHAIFL
>PxylPBP1
MWFPVRLSAVLLITLAVMDTQGSKETMKDITSGFFKVLNECKHELNLPDHLVGDFYHYWRQEYALLDRDLGCA
ILCMSRKLELIDASGKLHHGNTQEFAEKHGADNSMASKLVEVLHSCEKQHEAVSDECQRALEVAKCFRSSVHEL
GWAPTIDVIIEEVLTDM
>PxylGOBP1
MERRWCLLVLAAAAAGLPGVVRGTVEVMKDVTLGFGEALEQCREQSQLTEEMMEEFYHFWREDFKFEARAV
GCAIHCMSRYFNLLGEQQRMHHDNTHKFIQSFPNGEVLSHQMVGIIHTCEQQHDAETDHCWRILRVAECFKR

ESQAQGLAPSMEMLMAEFIMEADV
>PxylGOBP2
MASVWSLVVCGLMMAALPAARGTAEVMSHVTAHFGKTLEECREESGLSGEIMEEFHHFWREDFEVVHRELG
CAIICMSNKFQLMQDDARMHHENMHDYIKSFPKGDLLSETMVRLIHNCEKKYDDIDDECSRVVKTAACFKKD
AQAEGIAPELTMIEAVLEKY
>DindPBP1
MWVKMILITVAVVMMSVKVDLSQTLLKDMTKNFLKAYEQCQQELSLPESTAKELMFFWKDGYEVSSREAGCT
ILCLSKKMDLIDPEGKLHKGKSADFVKQHGTDDETANKVVDILHTCESNAAPNDDHCMVALAVALCFKKEIHNL
NWAPDPEVILEELMAEMS
>MsepPBP
MVLHRSATMSAHLALVVIASFLIAVECSQEIMKNLSINFAKPLEDCRKEMDLPDSVLTDFNNFWKEGYEFTNRQ
TGCAILCLSSKLELLDPEMKLHHGRAQEFAQKHGADEAMAKQLVDMLHSCMQTTPDDANDPCLKTLKVVTCF
KTKIHELKWAPSMDLIVGEVLAEV
>EposPBP1
MMNHKELVLFAVVCLSLYQAVEPSQDVVKDMSLNFRKGLDACKKELNLPDTINADFNRFWNDDHVVTNRDT
GCAIMCLSSKLELVTDTGLHHGNTLEYAKQHGADETVAQQIVDLLHTCAQAVPDLQDTCMKVLEWAKCFKAEI
HKLNWAPSAEVMAAEMLAEV
>EposPBP2
MATVSKWRMLVLTLCLTGVWQVESSADVMKKLTTGFATALEKCRDELNLPDAVMQDFFNFWREDYELVNRD
MGCAIMCMATKFDLVTEEQKLHHGNAHEFAKSHGADDSMAKQLVTMLHECETQTASISDDCGRTLEIAKCFR
TKIHGLKWAPSMETILEEVMTEV
>EposGOBP1
MATVSKWRMLVLTLCLTGVWQVESSADVMKKLTTGFATALEKCRDELNLPDAVMQDFFNFWREDYELVNRD
MGCAIMCMATKFDLVTEEQKLHHGNAHEFAKSHGADDSMAKQLVTMLHECETQTASISDDCGRTLEIAKCFR
TKIHGLKWAPSMETILEEVMTEV
>EposGOBP2
MASYWAVCVVLVAGSHLVAGTAEVMSHVTAHFGKALEQCREESGLSTAVLEEFQHFWRDDFEVVHRELGCAIL
CMSNKFSLMQDDARMHHENMHDYVKSFPQGEVLSAKMVELIHNCEKPYDDIKDDCERVVKVAACFKVDAK
KAGIAPEVAMIEAVMEKY
>CfumPBP
MLKQKELLLFAXVCLSLTQMVEPSQDVIKGMCLNFGKGLEECKKEMNLPDTVDADFYNFWKDXYVLTNRDTG
CAIMCLSNKLELVSDGKLHHGNTLDFAKQHGADETVAQQLVDLIHTCEKALPDLEDPCLKVLEWAKCFKIEIHKL
NWAPTMDVLAGEMLAEI
>LdisPBP1
MSAKLLLVLAVCLVMRVDCSKEVMKQMTINFAKPMEACKQELNVPDAVMQDFFNFWKEGYQITNREAGCVI
LCLAKKLELLDQDMNLHHGKAMEFAMKHGADEAMAKQLLDIKHSCEKVITIVADDPCQTMLNLAMCFKAEIH
KLDWAPTLDVAVGELLADT
>LdisPBP2
MMWLRLILAVAVCLVIPVEPSKDVMHQMALKFGKPIKLCQQELGADDSVVKEFLDFWKDGYVMKDRQTGC
MLICMAMKLELLDSAMEIHHGSTFAFAKAHGADEAMAQQIIDIVHGCTTTYPAAETNDPCQRAVNVAMCFK
AHVHKLNWAPDVELLVADFLAESQ
>CsupGOBP1
MEAAKVIMAGLLVVGVVPSMRADMVVMKDITLGFGAALEHCREESGLTQENMEEFFDFWREDFKFEHRELG
CALRCMSRYFNLITDTNRMHHENTENFIKSFPNGEKLSKVLVQVIHECEKKFDHEEDHCWRILHIGECFRDMCR

SQNIAPAMEMLLAEFIMQAESDTNPVAL
>CsupGOBP2
MVCSGFYLGLVVMAAVTSVKGTAEVMSHVTAHFGKALDECREESGLSTEVLEEFKHFWSEDFEVVHRELGCALI
CMSNKLSLLHDDTRVHQVNMHDYVKSFPNGEALSEMMVKLIKNCERQYDNIKDDCDRTVKVAACFKADAKK
EGVAPEITMIEAVMEKY
>CsupPBP
MSLYMRIVVLALVYLFNGVESSQEIMKQLSLNFGKAYDSCKKELELPNEVDTDFFNFWKDDYAVTNRLTGCAIM
CMSNKLDLLDPDGKMHHGNAREFAKKHGADDSMAQQLIDILHNCEKGASPGPDGDACVQVLEISKCFKVEIH
KLNWAPSMDLIMAEVLADV
>CsupPBP3
MKCCLFGILVCFNVMVSDVESSQELIKKMSISFLKVLQECKLELSVPEEVLQSLMTFWNQDTDLSHRELGCVILC
VVSKLDLIELETYKLHPDNANEYVKKHGADDETASQIMNILRGCEIKNEAITDHCDRVREIAKCFHGHMHELKW
APNMEVIINELVATKAI
>CsupPBP1
MVRDTMMLKLVVVMCLTMTVVVDSSQTVMKSMTKNFLKAYEVCAKEYSLKEGTAGILIGFWKDDFSTTSRDV
GCAILCLSTKLDLIDPEGKLHHGKATEFAMQHGSGEEMAKKLVEILHNCEQTVTPNEDKCMRALDIAMCFKKEL
HTLGWAPDPELLFEELIAEMR
>CsupOBP
MRCCAVLFVLAFIGCIYAEQEIVHLPPEKVAQILPVAMQCVGESSVPPEVIFQYASGKSLGNDKKYQKFIHCVFTK
TGYADETGHINIDKAMEVFPKGTDKEAVKKIMEECSKERGEDPPETSFKFAKCFRKKAPVRIAL
>CsupOBP1
MASFHFKVNCFLYFVLLSSYFVYSMTRQQLKNSGKLLKKACIPKTNVSEEQIRDIDKGKFIGEKKIMCYIACIYTMS
QAIKNNKIQHDVMIKQVETMFPNDIKESAKFAIQQCRGIAKQHKDICEAAFWTTKCLYDADPATFIFP
>CsupOBP2
MGTVSTEHYPAHHVINMKAFIVLAVCIVAAQALTDEQKEKLKKHKSECLAETKVDEQLVNKLKAGDYKSDNEAL
KKYALCMLIKSELMTKEGKFKKDVALAKVANPADKPQVEKLIDTCSANKGNTPHQTAWNYVKCYHEKDPKHAIF
L
>CsupOBP3a
MFLKLLMLTVLFCAIHAMTRQQLKNSGKMLKKNCMGKNQVTEDQIGSIEKGKFIEDKNVMCYIACIYQMTQVI
KNNKLNYEASLKQVDIMYPAELKESAKKSIENCKHISSKYKDICEASYWTAKCMYEDNPKDFVFA
>CsupOBP11
MGFSRAVLLAAFVAGAWAMDEEMAELAKMLHDNCGEETGADLSLVDKVNAGADLMPDPKLKCYLKCIMET
AGMMTEGVVDVEAVLALLPDDMRAKNEQNLRGCGTQKGADHCDTAFLTQLCWQKANKADYFLI
>CsupOBP5a
MLLIIIAKFLVLVAICEAMTMKQIKNTGKMLRKTCQPKNNAADEKIDPLNEGVFIDEKEVKCYIACIMKMANTM
KNGRPNIEVAMKQADLLLPEELKEPAKEALTACRKVPDAHKDVCDAAFHLTQCVYNQNPDIFYFP
>CsupOBP6
MAKFVVLCLGLLAAALSVKALTKEELDHIKEATLMHFNECNKDFNVSEDDIKAAETQKNMDKIDACLIGCMMK
RSHLLDGEGKFDTEKAIELSKSFMKSEDDQKKFAEVVAECAKVNDEPVSDGANGCERSKMVLVCLAKHKAEFV
PARR
>CsupOBP5
MKLTLFPAFLLFTSILGLKDEHEENLKRWHMECFQETKVNPDLVLKLMGNWQIKNKLLKEWILCVFNKYDLM
SKEGVFKLDTAMSLVPSADRDMIEDYIDACLPKHIAEPLDIAWKYAKCYHVGAKDPINKNKRLHYMTLFY
>CsupOBP7

MTSGSEQDIIKTPEETDTNPDLMAIMGECNETFRIETSYLESLNESGSFPDESDKTPKCYIRCVLMKTGVTTEDG
KFIPDVTSQVFASQNIKEQMDGIQNMATACAVDRNESCKCDRSYMFMKCLMESEIKSFMKIM
>CsupOBP8
MGFSRAVLLAAFVAGAWAMDEEMAELAKMLHDNCGEETGADLSLVDKVNAGADLMPDPKLKCYLKCIMET
AGMMTEGVVDVEAVLALLPDDMRADHCDTAFLTQLCWQKANKADYFLI
>CsupOBP9
MNCSVIAIIFAFLSLTSAELELSDEIKEIIQHVHNECVAKTGVAEEDIKNCENGIFKEDEKLKCYMFCLMEEANLAD
DDGVVDYEMMVSIIPEQYTDRVTKMIFACRHLDTPEKSKCQRAFDVHKCSYSKDPEFYFLF
>CsupOBP4
MFRLFLCLVVFVTTSYGDLLTQERSRGATLKPISACCGIPELGDSKPLTECSKPKLLGPCNDIQCVFEKSGFLVDRNT
LNKDVYKKHLRKWAEAHDSWTEAVERAIADCVDKELRQYLDYPCRAYDVFTCTGIAMLKKCPQEAWKC
>LstiPBP
MGFSVRLLVVLVAVTIYGVNSSQDIIKQMTINFGKALDSCRKELDLPDSINADFYNFWKEGYELSNRQTGCAIMC
LSSKLDLVDPEGKLHHGNTHEFAKKHGADDAMAKQLVDLIHKCESDVPDDPDPCLKVLNIAKCFKAEIHKLNW
APSMDLIMAEVLAEV
>LstiGOBP2
MSSAWILLGLVMAAVSSVRGTAEVMSHVTAHFGKALEECRTESGLSPEILEEFQHFWSEDFEVVHRELGCAIIC
MSNKFSLLQDDTRIHHVNMHDYVKSFPNGEVLSEKMVSLLHNCETQYNDMTDDCDRTVKVAACFKADAKKE
GIAPEVAMIEAVMEKY
>AtraPBP1
MKMHLFVQVIAASVMLMAGVDSSPEIMKDLSINFGKALDTCKKELDLPDSINEDFYKFWKEDYEITNRLTGCAI
KCLSEKLEMVDADGKLHHGNAREFAMKHGADDAMAKQLVDLIHGCEKSIPPNDDRCMEVLSIAMCFKKEIHN
LKWAPNMEVVVGEVLAEV
>DpleGOBP1
MKDVTLGFGEALKLCREESQLTEEKMEEFFHFWRDDFKFDDRAVGCAIKCMSSHFDLLTDSHRMHHRNMDN
FIKSFPNGEVLSQQMVTLIHECEQQHDSEEDHCWRILRVAECFKSSCKKHGIAPTMELLMAEFVMESEAN
>DpleOBP1
MTRQQIKNSSKMLKKNCMGKNDVTEDMVGEIDKGKFIEDKNVMCYIACIYQMSQLVKNNKLNYEASIKQVDL
MFPPDMKEAMKASIENCKDISKKYKDICEASYWTAKCIYDDNPKNFIFA
>DpleOBP
MYILAVVKFLAALTICEAMTMKQIKSTGKMMRKTCQPKNNVADDKIDDIGTGVFIEEREVMCYVACIMKMAN
AIKNNKLNYEAAIKQADLLLPDEIKEPTKEAITACRKVADSYKDICEASFHVTKCIYNHNPSIFFFP
>DpleGOBP1a
MKKLTTGFVKAMEECKAELNLGDHIIQDFMNYWREEYELLNRDTGCAIMCMASKHDLITEDMKIHHENAHEF
AKSHGADDDLAKQLVQMIHDCEKQFTDITDDCSKTLEISKCFRTKIHELKWAPSMETILEELMTET
>DpleOBP4
MKKWFLELTVECSKEHPVTKEEIQMLKDHKIPDNKNVKCLMGCVFRKIGWLDDNGMFSFNNAYKTSEEEYPD
DKTKLEKAKNLYSLCEKVNTAEVSDGKEGCERSSLLAKCLIENSSKMGFVVQ
>DpleOBP2
MAVRGLLLLSAVLAATSVTVDGTAEVMSHVTAHYGKSLEECRKETGLSKEILEEFKHFWSDDFEIVHRSPFTVRL
HPHLHDDARMHHINMNDYVKGFPEGDVLADKLVQMIHKCEKEYDDIKDDCDRVVKVAACFRADAKKEGIAP
EVAMIEAVMEQY
>DpleOBP1a
MTAEQKQMVHQHFEQVGMECIKTHEITAEDVTNLRTRKIPTGENASCFLSCIFKHVGIMDDNGLLQKESAIELA

KKVFDDEEELKLIEDYLHSCSSVNTATVSDGEKGCERSLLAYKCMIENASQFGIDL
>DpleOBP4a
MRIMCVVFVLSFGYVYGLTDEEVKKEFIKEVMTCTKDITVDMFDLMELEQLKVPTKTNVKCVLACAYKRVGTM
NKEGKYDIKEAYKISETMMKGDDKRIENGKKLADLCSKVNEADVRDGNKGCERAALLFKCVIENAPKLGFKV
>DpleOBP5
MECSKDYPLTDEDIAQLKDKQFPDKDDVRCLFACAYKKTGMMDDQGKLSVDGVNNLAKKYFSDDQDKLQKS
QKFTEACAGVNDEAVTDGEKGCERAALIYKCSIEQASQITIGYQTCKILAKRPRIGVLAQAQEQDRLNRDLFLHL
GNTMQQ
>DpleOBP6
MLNLLRIALFIVAVNADLLNHERTKGATLKPISVCCDIPELGDPKPLAECSNMKLQGPCSDVQCVFEKSGFLLDKQ
TLNKEGYRNHLMKWLEGHKEWKDGIEKAISDCVDVDLRQYLDYPCKAYDVFTCTGIAMLKKCPKDSWKC
>SinfPBP1
MADSRWWLASFICVMIMTSSVMSSKELVSKMSSGFSKGLDQCKAELNVGEHIMQDMYNFWREEYELVNRD
LGCMVMCMASKLDLVGEDQKMHHGKAEEFAKSHGADDELAKQLVGIVHACETQHQAIDDPCSRTLEVAKCF
RSKMHELKWAPTMEVAIEEIMTAV
>SinfPBP2
MALHRSPTMSARSVLVLIASLFVIVKCSQEVMKNLTHNFSKPLEDCKKEMDLPDSVFTDFLNFWKEGYEFTSRH
TGCAIVCLSSKLELLDPDLKLHHGKAQEFAQKHGADEAMAKQLVGLIHGCMEAIREPADDPCMRALNVAKCFK
AKIHELNWAPSLDLIVGEVLAEV
>SinfPBP3
MRSNNIIFSVLLMSVGVREIEPSKDAMKYITSGFVKVLEECKKELNMNDHIIADLFHYWKLDYTLLNRDTGCAIIC
MSKKLDLLDVNGRMHHGNAREFALKHGAGDEVAGKIVDIIHDCEKKFERDDDECLRVLEVAKCFRTGIHELDW
QPKVEVIMSEVFTEI
>DtabPBP1
MTKTYTFLAVAIVLLAIDSRVDSSQDVMKDLSVKFGESMNQCIKEMDLPDVSADFYNYWKEDFVITRRETGCLF
SCLAKKVSMQHSDGLLHKDNTHNFATKHGADDEMAAKLVETIHACENSISESDDCVRVLSIANCFKKEMHKLN
WAPSAELVTQELMTIL
>DtabGOBP2
MLVYLVPLVIGLVMEPVVGTAEVMSHVTAHFGKALQECRDESGLSPEILEEFQHFWSEDFEVVHRELGCAIICM
SNKFSLLQEDTRMHHVNMHDYVKSFPNGQVLSEKLVQLIHNCEKQYDSITDDCERVVKVAACFKVDAKKEGIA
PEVAMIEAVMEKY
>DtabGOBP1
MRWTQLALLALCCLMQARGDQTVMKDVTLGFGQALEKCREESGLTDEKMEEFFHFWHDDFKFVHRELGCAI
LCMSRHFNLLTETSRMHHENTDNFIKSFPNGEILAAKMVEIIHTCELRFENEADHCARILRIAECFRDTCKSVDLA
PTMEILIAEFILQAESGKR
>GmolPBP3
MARFSILFSLVIIAVTVKEIELSSDAMKAITSGFLKVLEQCKQELNLQGHVISDLYHYWKEDYSLLNRDTGCAIICM
SKKLDLIDASGKLHHGNTAEFAAKHGAASEVASKLVEILHACEKTHDAIEDDCMRALEIAKCFRTDINQLNWTPK
MDVIITEVLTEM
>GmolPBP2
MAASGRWRMLLAVFVLSVCVNRVTPSADIMKKLTTGFATALDKCKNELNVQENVMQDCYNFWREDYELLNR
DTGCVILCMALKFDLIDEDAKLHHKNAHEFAKTHGADDDLAKQLVAMIHDCEKQNSDADDCIRTLGIAKCFRTK
IHGLKWAPSMETVLEEVMTEVKPS
>GmolGOBP1

MSKNLVRLLLALTAVAVAQATQEVLKDVTLGFGEALEHCRESTGLTTEKMEEFFHFWSDDFKFELREVGCAIQC
MSKYFNLLTDGERMHHENTDKFIKSFPNGEVLAKQMVTLIHTCEQQFDDMEDHCWRILRIAGCFKTGCQERG
IAPSMELIMAEFIMEADA
>GmolGOBP2
MALYWMVTVLLVVGGRMVDGTAEVMSLVTAHFGKALEQCREESQLSPEVLDEFHNFWREDFEVVHRELGCA
IICMSNKFSLLQDDARMHHDNMHDYILSFPKGDVLSAKMVELIHNCEKQYDDISDDCSRVVKVAACFKVGATQ
AGIAPEVAMIEAVLEKY
>CpomPBP2
MAAAAKWRTLVAAFILTLCVNRVTPSAEIMKKLTTGFATALDKCKTELNVQENVMQDFYNFWREDYELLNRDT
GCVIICMALKFDLIDEDAKLHHKNAHEFAKTHGADDDLAKQLVGMIHECEKATTEADDCIRTLDIAKCFRTKIHG
LKWAPSMETVLEEVMTEVKPS
>CpomGOBP2
MALYWLVGLVLVGGKMVDGTAEVMSHVTAHFGKALEQCREESQLSPEVLDEFHNFWREDFEVVHRELGCAI
MCMSNKFSLLQDDARMHHENMHDYVKSFPQGDVLSAKMVELIHNCEKQYDDIPDDCSRVVKVAACFKVDA
KKAGIAPEVAMIEAVLEKY
>CpomGOBP1
MSHIARLVLSLAAVALAHATVEVMKDVTLGFGEALEHCRESSGLTEEKMEEFFHFWHDDFKFEHRELGCAIQC
MSRHFNLLTDGQRMHHENTDKFIKSFPNGEVLSKTMVTLIHSCEQKFDDMEDHCWRILRIAECFKSGCQERGL
APSMEMMMAEFIMESEV
>HevirPBP
MVFHRSTMMSVRLVLVVAVGLLIRVDASQDVIKNLSMNFAKPLEDCKKEMDLPDSVTTDFYNFWKEGYEFTN
RQTGCAILCLSSKLELLDQELKLHHGRAQEFAKKHGADDAMAKQLVDLIHGCAQSTPDVADDPCMKTLNVAKC
FKAKIHELNWAPSMELVVGEVLAEV
>HevirGOBP1
MRGELRALLLLAAAAPLLADVNVMKDVTLGFGQALDKCREESQLTEEKMEEFFHFWRDDFKFEHRELGCAIQC
MSRHFNLLTDSSRMHHDNTEKFIQSFPNGEVLARQMVELIHSCEKQFDHEDDHCWRILHVAACFKGACVQRG
IAPSMELMMTEFIMEAEAR
>HevirGOBP2
MTSKSCLLLVAMATLTTSVMGTAEVMSHVTAHFGKALEECREESGLSAEVLEEFQHFWREDFEVVHRELGCAII
CMSNKFSLLQDDSRMHHVNMHDYVKSFPNGHVLSEKLVELIHNCEKKYDTMTDDCDRVVKVAACFKVDAKA
AGIAPEVAMIEAVMEKY
>CmedOBP1
MDGKNLPSGVFLIILVILLSSDFSFGMTRQQLKNSGKIMKKSCMPKNDVTEDQIGEIEQGKFIEERNVMCYVAC
VYTMTQVVKNNKLSYEAVVKQVDMMFPPEMRDAVKAAAAHCKDVAKKHKDLCEASYWTAKCMYDFDPKN
FVFP
>CmedOBP2
MLLVILAKFLMVLAMCNAMTMKQIKNTGKMMRKSCQPKNNVADEKIDPLGDGVFIDEKEVKCYMACIMKM
ANTMKNGKPNYEAAIKQVDLLLPEEIKQPAKEALAACKKVPDAHKDPCDAAFHVTKCIYNHNPSIFFFP
>CmedPBP1
MGFLVKLVLLAMVVGVQSSQDVMKKVTVHFSKALETCKKELDLPDAINTDFFNFWKEDYELQNRLTGCALMC
MSSKLDLVDPEGKLHHGNAHEYAKSHGADDSVAKQLVDLLHGCESSTAQSDDDCSRVLGIAKCFKAEIHKLKW
APDME
>CmedPBP2
MWAKTLMVVVTVVMMSVNVESSQTLLKDMTKNFLKAYGQCQKELGLPDSTATELMNFWKEGYEIKSREAGC

AIMCLSKKLEVIDPEGKLHKGKTTEFIVAHGTDEATAHKLIDILHACMQSVTPSEDHCLMSLQVAMCFKAEIHKL
GWAPDTELLFEEMVAEMQ
>CmedPBP4
MEVEMLPEGMKQLTGGFIKVFEACKTELGLKDGMLTDMYHLWREEYDQVSPDAGCMFGCMSKKLDLLDAS
GKIHHGNTKEYVMQNGGGEDLAAQLLSISQECEKQHEGVEAECARMLEMAKCFRSGIKRVQWSPKMEVVIT
EIIADV
>CmedGOBP2
MLSSWVLLGLMMAAVASVKGTAEVMSHVTAHFGKALEECREESGLSPEILEEFKHFWNEDFEVVHRELGCALI
CMSNKFSLLQEDTRIHHVNMHDYVKSFPNGEVLSDKMVELLHNCEKQYDAITDDCDRTVKVAACFKKDCQKE
GIAPEITMIEAVIERY
>CmedGOBP1
MRLEAALLCALAAAALVEGSHKIMSDVTLGFGQALEHCREESGLTEDKMEEFFHIWSKDFKFQDRELGCALRC
MSKHFNLITDANELHHENTENFIKSFPNGEQLAKELVVLIHNCEKQFEGVEEHCMRTLRVGECIRDACLSRDLAP
TMELLLAEFIMQTEA
>DpunPBP1
MTKTYTFLAVAIVLLAIDSRVDSSQDVMKDLSVKFGESMNQCIKEMDLPDVSADFYNYWKEDFVITRRETGCLF
SCLAKKVSMQHSDGLLHKDNTHNFATKHGADDEMAAKLVETIHACENSISESDDCVRVLSIANCFKKEMHKLN
WAPSAELVTQELMAIL
>DpunGOBP2
MLVYLVPLVIGLVMEPVVGTAEVMSHVTAHFGKALQECRDESGLSPEILEEFQHFWSEDFEVVHRELGCAIICM
SNKFSLLQEDTRMHHVNMHDYVKSFPNGQVLSEKLVQLIHNCEKQYDSITDDCERVVKVAACFKVDAKKEGIA
PEVAMIEAVMEKY
>DpunGOBP1
MRWTQLTLLALCCLMQARGDQTVMKDVTLGFGQALEKCREESGLTDEKMEEFFHFWHEDFKFVHRELGCAIL
CMSRHFNLLTETSRMHHENTDNFIKSFPNGEILAAKMVEIIHTCELRFENEADHCARILRIAECFRDTCKSVDLAP
TMEILMAEFILQAESGKR
>DsupPBP1
MTKTYTFLAVAIVLLAIDSRVDSSQDVMKDLSVKFGQSMNECIKEMDLPDVSTDFYNYWKEDFVITRRETGCLF
SCLAKKQSMHHSDGELHKDNTHKFATIHGADDEMAAKLVETIHTCENSISESDDCVRVLGIANCFKKEMHKLN
WAPSAELVTQELMAIL
>DsupGOBP2
MLVFLVPLVIGLVMEPVVGTAEVMSHVTAHFGKALQECRDESGLSPEILEEFQHFWSEDFEVVHRELGCAIICM
SNKFSLLQEDTRMHHVNMHDYVKSFPNGQVLSEKLVQLIHNCEKQYDSITDDCERVVKVAACFKVDAKKEGIA
PEVTMIEAVMEKY
>DsupGOBP1
MRWTHLTLLALCCLMQARGDQTVMKDVTLGFGQALEKCREESGLTDEKMEEFFHFWHDDFKFVHRELGCAIL
CMSRHFNLLTDTSRMHHENTDNFIRSFPNGEILAKKMVEIIHTCELRFEDEPDHCARILRIAECFRDTCKSADLAP
TMEILMAEFILQAESGKR
>DhouPBP2
MAVLTKWRMFVVFIYLNLNNVQSSQDIIKSMTSSFGKLVTECQTELELGDEIIQEFVNYWSEDYQLVNRDIGCM
ILCMASRLDLVNFTDMTLHHGNAHEFAKSHGADDATAGQIVIIIHDCEKANIDEGDLCTRIMKVAKCFKVNMH
DLKWAPSIEVILEEVMISV
>DkikPBP2
MAVLTKWRMFVVLVFLNLNNVQSSQDIIKSMASSFGKLVTECQTELELGNEIIQEFVSYWREDYQLVNRDMGC

MILCMASRMDLIEFGDMTLHHGNAHEFAKSHGADDATAGQIVTIIHDCEKANMDEGDLCTRVMKVAKCFKV
KMHDLKWAPSIEVILEEVMISV
>DhouOBP17
MKTFILIAVCLVAVQALTDEQKEKLKKHKTECLAETKPDEQLVAKLKNGDYKTESEPLKKYALCMMVKSELMTKD
GKFKKDVALAKVPDAADKPMVEKHIDACLANKGSTPQETAWRYVKCYYEKDPKHAIFL
>DhouOBP10
MFRPLFIYFTFIYFLFCDVQAQSNLDELQKNYLNFILECAVENPVTSDDLEELKNQKMPDKENVKCLFACAYKKA
GMMNDKGDLFIEGVQEINKKYFGDNPEKMNKSEQFIQACKSVNDSPVSDGNKGCDRAALIFQCSVEKAPDFE
LVV
>DhouOBP13
MGAYLFMCLMLTAVGLEAHTIHLTHTQKDKAHQLTMECMKESNVKPEVITEAKKGHYADDEKLKKFTLCFFQK
AGILTPDAKLNVDTALEKLPPGVDKAEATKVLEECKNKTGKDKADTAFEIFKCYHHGTKTHILF
>DhouOBP6
MSRTSVVIFWTLYLMGLFCPNNAAVTAEQKSKIHEKLEGIGLLCVKDNAITEDDITSLRARKVPGGPNAPCFLAC
MMKEIGVMDSDGKIQKESALEIAKTIFEDSEDLKNIEDYLHSCAHINTEEVSDGDKGCDRAMLAYKCMTENSSK
FGFDNV
>DhouOBP11
MVRSASLIVVALAVLSKYSLALSDEKKTQVYGLLFAKAVECGKEFPISDDEVLALKNKKIETDNAKCFAACTLKKAG
ALDEAGKLSSAGAVKAIQDTLTDPADIKNLEDFFAHCSSVNDEAVNDDKGCDRAKLIFDCFRDNADAYGFNLDF
>DhouOBP4
MLGIRVGIIALFVLQAYCFDDEVLELAKMVRESCAEETNVDISLVEKINTGAGLTPDPVLKCYIKCTMETAGMMS
DGVMDLEVVMTLMPDDLKNKHGAGLEACGTQKGADDCDTAYLTQVCWQKTCKSDYFLI
>DhouOBP9
MFSITLSTAFLYLIAIIQAGKEKPIFSDEIKEIIQIVHDECVEKTGVTEEDITNCENGIFKEDVKLKCYMFCLLEEASLV
DDEGVVDYDMLISLIPDAYHDRASHMLLSCKHLDTPDKDKCERAFDVHKCSYEKDPDLYFLF
>DhouOBP3
MYFQVILCFCMVGACYARTDTEIRAWFFRQGVECSKDHPVTVEEMKLMHQHQFPESRNSKCIGACVFRRAN
WLDSKGMFDLEQGIAFSEKEYADNPTKLEAGKKLFQFCTKVNEEPVTDGEDGCDRALLLSKCLIGNAKEYGFEVI
PTDGPEE
>DhouOBP1
MLFKVIIHLFVYWIFCEAMTMKQMKNSGKMMRKTCQPKNNVEDEKLDGIMKGEFLDDTNLKCYMACIMKM
ANAVKNGKINYEQSLKQADLLLPEEIKEEAKAAITTCKNAANGHKDICDASFHMTKCVYEANPSIFYFP
>DhouOBP16
MKIQIVVLFSILVVVSRAERTPYHLPEKETENLLQVGSQCLTETNVPTETWDAIKKDKYEHDDIYKKFLFCMMTKL
EFVDDQGHPIKNKLYGTFKEDDLKKVIDKCGADPKGDTPVDVTYSFYKCCIDNTNIRIAI
>DhouOBP7
MNLDSLLVTVLMFSNMLCVWSVTRQQLKNSGKLLKKACMPKHDVTEEQVGNIDKGTFIEDRNVMCYIACIYS
VGQAVKNDKIIHDAMIKQVNQMFPAKMKDAVKAAIENCRGVGKSTIRNKLTVFVWRLCHI
>DhouOBP2
MSIKYVCVALLAFTAYAIALTQEDKQAFLTSIAPLIGECSKEYGVNPEEIEAAKAAHSGESLKPCFVACFFKKLGVIN
DNGDFDPEASKAKGKQFFKDVEDQNKVSEIADICTSINAETVSDKTQGCDRSKLLMECYSKNKGGFSPFV
>DhouOBP14
MQIHSSVSVFVVCAILFNGQTNCARTVAKAPPELAGPILETISACGEETKNNPERIMAAMKDNKVFEDDAFLKFI
DCTFQKSGYAKPDGTLDIEKSVKIFPKNVDMKTVFENCNKDKGSTRVETTFKIYKCYQQTSPVQLVF

>DkikOBP14
MCLGTLVANTFGITQEDIRKFREVMRPIVSSCANQYGITLEQVIEARKAGTMEQLDPCLSECVFKKTGVINDKGL
YDPDTAIEKSKKYVTDPADVEKFNNIHKACIPVNDAPVGDSKDCERAKLLLSCLVKHKGEFSTLAL
>DkikOBP7
MFRPVFIYFTFIYFLFCDVQAQMNLDELQKAYLNYVLECAVENSVTPDDLEELKNLKMPDKENVRCLFACAYKK
AGMMNDKGELFIEGVQEITKKYFSDNPEKMKYSEQFIQACESVNDAPVSDSKKGCDRAALIFKCGVENVPDFE
FIV
>DkikOBP20
MKAFILIAVCLVAVQALTDEQKEKLKKHKTECLAETKPDEQLVAKLKNGDYKTESEPLKKYALCMMVKSELMTKD
GKFKKDVALAKVPDAADKPMVEKHIDACLANKGSTPQETAWRYVKCYYEKDPKHAIFL
>DkikOBP6
MCSAELCVLRDLFADKLEYDNDFNNVLVPAIRECTSEMGYTEDDLDTARKNQQLDDCMFICVFHKMGLMKDD
KMDLDETYALTEKFFKPDTDKEDIRKVSVDCYKVSDAKSENDKKCCDRARLIFDCLKDNIM
>DkikOBP13
MFSITLSTAFLYLIAIIQAGNEKPIFSDEIKEIIQIVHDECVEKTGVTEEDITNCENGIFKEDVKLKCYMFCLLEEASLV
DDEGVVDYDMLISLVPDAYHDRASHMLLSCKHLDTPDKDKCQRAFDVHKCSYEKDPDLYFLF
>DkikOBP13a
MKNLIFIITFALITFSLTNPIRAEDDKNKQTDQASKAIKEIDLNEVMAECNKSFTIQMSYVDALNKTGSFPDESDKT
PKCYVRCMLEKTGIMSSDGVFDLKRIPVVFANQHDGDFLVKDEVIASLCAERKEKCQCEKAYNFMKCFRTTEIN
YYEKETQ
>DkikOBP11
MNLDSLLVTVLMFSNMLCVWSVTRQQLKNSGKLLKKACMPKHDVTEEQVGNIDKGTFIEDRNVMCYIACIYS
VGQAVIKNDKIIHDAMIKQVNQMFPANMKDPVKAAIENCRGVAKNYKDICEASYWTAKCMYDYDPSNFVFA
>DkikOBP15
MTRAQVKNTMTMMKKQCLPKTGATEDKVARIEEGVFIEEHSVMCYIACVYKAIQVVNNDRLDMGLVSKQIDL
LYPQNMKEPAKKAAGQCVSIQDKYSDMCEAIFYATKCYYEADPSSFIFP
>DkikOBP19
MKLQIVVLFSVLVVISSAERTPYHLPPKETENLLQVGAQCLTETNVPTETWDAIKKDKYEHNEIYKKFLFCMMSK
LEFVDNQGHPIKNKLYETFKEDDLKKVIDECGADPKGDTPVDVTYGFYKCCIDHTNIRIAI
>DkikOBP3
MLSYKYPIFIVFSHFDVVAPNMYDKRIQICIQALSLILLLVGDCYAMTKQQLKKSGKMMKKSCMPKNDVTEDEV
GEIENGKFIEEKNVMCYIACIYTMGGVVKNNKIALDLMLKQIDTMFPAEVKDELKAAANSCKDTLKKYKDICESS
YFMAKCLYDTAPDAFLFP
>DkikOBP18
MLHCKTTVLVFVFVSTVYADMKRPFQFPKERIDNFMNTASTCNKELNTNIDVSELFKGKYDQSESFKDSFYCIAV
NSGFYDANGWPKLEKLYEIYKDEDVRTVLKDCTADLDGERPKDLASNYLICFLVKAPITVIF
>DkikOBP16
MQIHSSVSVFVVCAILFTGQTNCARTVVKAPPELAGPILETISACSRETKNNPERITSAMKENKVFEDDAFLKFID
CTFQKSGYAKPDGTLDIEKSVKVFPKNVDMKTVFENCNKDKGSTRVETTFKIYKCYQQTSPVQLVF
>DkikOBP5
MCRTSAVIFCTLYLIGLFGSNHAAVTAEQKSKIHEKLEGIGLLCVKDNAITEDDITSLRARKVPSGPNAPCFLACM
MKEIGVMDSDGKIQKESALEIAKTIFEDSEDLKNVEDYLHSCAHINTEEVSDGDKGCDRAMLAYKCMTENSSKF
GFDSV
>DkikOBP22

MYGVVAIFTFLAVAWGDTPSDPSRVYCGFTPDSIYACLHNPKVIKQDVSVKCDKSTSECDRMNCIFRESGWLDG
SNVDKQKVSAYFDQFAKDQPEWSTAVQHLKTDCLNKDLPAQGVILNCPAYDIVHCALTAFIKNASPSQWSTAEQ
CAYSRSYASACPVCPSSCFAPQVPIGSCNACY
LPPRTPQS
>DkikOBP23
MVGQSICLLVAVILQVISAETPLNVLNPLHRECYRPPHNVERPHECCKLTSFYKEEDFKECGLDKIEEGEKSGYRH
GPPDCTKSNCLLAKNDMLKDDKPDLEKIKAYITNWADKNPAFKDAVDDAITKCIKEDLPGPPHACLASKLAGCLT
FRLFLKCPAENWESSANCDSVKEHIEKCK
SLFENPPQ
>DhouGOBP2
MLVFLVPLVIGLVMEPVVGTAEVMSHVTAHFGKALQECREESGLSPEILEEFQHFWSEDFEVVHRELGCAIICMS
NKFSLLQEDTRMHHVNMHDYVKSFPNGQVLSEKLVQLIHNCEKQYDSITDDCERVVKVAACFKVDAKKEGIAP
EVAMIEAVMEKY
>DhouGOBP1
MRWTHLTLLALCCLMHARGDQTVMKDVTLGFGQALEKCREESGLTEEKMEEFFHFWHDDFKFVHRELGCAIL
CMSNHFNLLTETSRMHHENTDKFIKSFPNGEILAKQMVEMIHTCELRFESEPDHCARILLVAECFRDTCKSTNLA
PTMEILMAEFILQAEAGKR
>DhouPBP1
MAKAYTFLAVAIVFLAIDSRVDSSQDVMKELSVRFGESMNLCIKEMDLPDVSADFFNYWKEDYVITRRETGCLF
SCLAKKVHMQHPDGLVHKDNTHNFATKHGADDEMAAQLVEVIHSCENSISESDDCIRVLGIANCFKKEMHKL
NWAPSAELVTQELMAIL
>SfruOBP
MSKFACLVLCVVAVSLSGVHATAEEKAAFIEAVKPHIQECSKEHGVTPEEIKSAKAAGNADGINSCFLSCVYKKAE
VINDKGEYDADKALEKLKKFVSNEDDYAKFAEIGKKCASVNEKSVSDGDAGCERAALLTTCFLEHKSEIPA
>PaegOBP
MVGFVALISLSLLAVCNADIGVDPPVKCGSMPPSVYSCLGTPRVVNPKLAAQCDKTLPECEKLTCIFRKSGWMD
GDKVDKVKLTAYFDQFATDNPDWQPAVDNLKTTCLQGDLPAQGVLLNCPAYDAMQCTFASFLKHAQPSQWS
TSESCNAARQYAASCPVCPTDCFASQIPVGSCNACLVLPRSP
>SnonGOBP1
MQEVVRALVLLSVAGALADVNVMKDVTLGFGQALDKCRQESDLTEEKMEEFFHFWREDFKFEHRELGCAIQC
MSRHFNLLTDSSRMHHTNTEQFIQSFPNGEVLAQQMVLMIHSCEKQFDHEEDHCWRILHVAECFKQECVSKG
VAPSMEMMMTEFIMEAEAR
>SnonGOBP2
MTTKCSLLLVVIAAVTSSVMGTAEVMSHVTAHFGKALEECREESGLSAEVLEEFQHFWREDFEVVHRELGCAIIC
MSNKFSLLQDDSRMHHVNMHDYVKGFPNGELLSQKLVDLIHNCEKQYDSIPDDCDRVVKVAACFKVDAKAA
GIAPEVAMIEAVMEKY
>SnonOBP1
MMKNSVVCLYFAVMAVNFWNVKCMSDEEKKAVLETLTPIVEGCASDYGLTNEDFKKHENIDDDNFLPFKKCLL
QKLEIMDETGKYNSDAISKSIVDYTGDKEQAKKFKEQFDSCFRKNGNNDGDDEASQMKRVFVMFKCFKEMEE
>SnonOBP2
MLLIKFVKFLILVAMCEAMTMKQIKNTGKMMRKTCQPKNNVEDDKIDPIAKGIFIDEKEVKCYMACIMKMAN
TIKNGNFNFEAALKQADLLLPDEVKEPAKEAIIACKKAADGHKDICDVSFHITKCIYNQNPGIFYFP
>SnonOBP3
MKSFTVFCVVLVAGIHAANVTLPPNQKDKARQIGAECIKETGVTTEILAEAKKGPIPEDESLKKFTYCFFKKAGIID

NDGKLNIDVALAKLPPGVDKDDAKKVLEDCKSKTGKEPQDTAFEIFKCYHAGTKTHILLTDA
>SnonOBP4
MKCLLVFAACILLAQALTDEQKEKLKKHRTECLTETKVEEQLVNKLKGGDYKTDNEALKKYALCMMVKSELMTK
DGKFKKDVALAKVPNAADKPTVEKLIDACLANKGNTPQQTAWNYVKCYHEKDPKHAIFL
>SnonOBP5
MLKFTCLALCVVAASISRVYAGEEEKAAFREAIKPIIEECSKEHGVSTDEIHSAKTAGNAEGIKPCFLGCVYNKAAVI
NDKGEYDVDTALLKLKKFVPDEEKYAKLAEVAKKCASVNDKPVTDGEAGCERAALLTACFLENKAEAFI
>SnonOBP6
MSKFTCIVLCVVAASLTKVSHAVTEEEKAAFHEVMEPIVDECAKEHGVSEAEIKAAHEAGSADSIKPCXLAMYHE
KTEMLDSKGLFDAEKSLSKLKKYVKNDEEFAKYEEIGKSCMSVNEKAVSDGEAGCERAKLIIDCFLVXHKADVPF
>SnonOBP7
MFTGTLPVVLCLCAVVYGGKDKPVFSDEIKEIIQTVHDECVGKTGVAEEDITNCENGIFKEDPKLKCYMFCLLEEA
SLVDDDGTVDYDMLVSLIPDEYYERTTKMIFSCKHLDTPDKDKCQRAFEVHKCSYGKDPELYFLF
>SnonPBP1
MADSRWWFASFICVIIMTSSVMSSKELVSKMSSGFSKVLDQCKAELNVGEHIMQDMYNFWREEYELVNRDL
GCMVMCMASKLDLVGDDQKMHHGKAEEFAKSHGADDELAKQLVGIIHACETQHQAIEDPCSRTLEVAKCFRS
KMHELKWAPTMEVAIEEIMTAV
>SnonPBP2
MALHRSPIMSARLALVLIASLFIVVKCSQEVMKNLTHHFSKPLEDCKKEMDLPDSVITDLHNFWKEDFEFTSRHT
GCAILCLSSKLELLDPDLKLHHGKAQEFAQKHGADEAMAKQLAGLIHGCMETIREPADDPCVRAQNVVMCFK
AKIHELNWAPSLDLIVGEVLAEV
>SnonPBP3
MRSNNIIFSVLLMAVGVREIEPSKDAMKYITSGFVKVLEECKKELNMNDHIIADLFHYWKLDYTLLNRDTGCAIIC
MSKKLDLLDANGRMHHGNAKEFALKHGAGDEVAGKIVDIIHDCEKKFERDDDECLRVLEVAKCFRTGIHELDW
QPNVEVIINEVFTEI
>HmelGOBP1
MISLLTLSLACGVLTGLDATMEVMKDVTLGFGEALQSCREESQLTEDKMEEFFHFWRDDFKFEDREVGCAMKC
MSSHFNLLTDSHRMHHENTDKFIKSFPNGEVLSKRMISIIHTCEQQFDALEDHCWRILRIAECFKVACKKEGIAP
TMELLMAEFIMEADPS
>HmelGOBP2
MAVRCYVVVFLALLALATLPRVRSTAEVMSHVTAHFGKSLEECREESGLSSDILDEFQHFWSEEFQVVHRELGC
AIICMSNKFLLMHDDARMHHVNMHDYIKSFPQGELLSEKMVNLIHNCEKQFDDIEDECSRVVKVAACFKESAK
EEGIAPEVAMIEAVLEKY
>HmelPBP_C
MFKRLTVVVLVFVGLNKVTSNETLRGITASFLKVLEECRQELNIGDNVLADMYYFWKLDRTLIHRDTGCAIVCMS
KKLNLLDTSGKLHHGNAEEFALQHGAADDMAKKLVTTVHECEQKHELEEDQCLRALEIAKCFRGAMHEINWA
PKVDVAISEILTEV
>HmelPBP_D
MAVQKWQLILVMCVLMQACKVSPSQEIMHKLTKGFATAFEQCKQELNLGDNIMQDFLNYWREEYELLNRDT
GCAIMCMAQKHDLLTEDGIHHEKVHGFTKSHGADDELAKQLVTMIHECEKSNAGVSDECMKTLEVAKCFRTKI
HELKWAPDMETILEEIMTDI
>HmelOBP3
MVGLKALHDIQINKDTIITRNMNLKTDNKISHNHDPDWSYSSFPKEVKSHVEQFKRNMSECLKEVQLNDKRQ
VRRLSPKKESPVHGECLIACVLKRNGVIENGKIYKDNLLSLVRKFYGKDEKLMKKLEKNVDRCIEASVKNKDDCTV

ASYLNECTNDLMANNKHKIIVNY
>HmelOBP6
MENISYKGKLFLLLFIAFAHVTLVDSYSHKFFSQNLDTEPSLSIQYARDKKSDMITNECLTEMYPKNIYRYPLHIDR
NDVPCIIHCVLKKFGIMSNDGVINTRNYYRRVRAIHRYDPRVLISDVGETCAQNINGMNLDHDVCKKAKVFND
CTQLYAISYRESDD
>HmelOBP8
MLIHAVLALIGYTLAAQHRSFSGTMVDLSDPKVQGHLDALVRMAQSCVIKVRASPKDVRAYFTNSPPITRSGQC
FAACMLEQSDVINHGKVNRELLVHLAGLVNGKNSRVVRKLYGISRLCLDSIEGMSDRCQLASTYNDCLNENMIE
FAFPLDIAEEAVRKMPFHLIQPK
>HmelOBP12
MKIIIIITLALLTFSVAQCGSSVGNRMSTDDITTTTMATTMEDDDARSNFDVMSVMVDCNDTFRVEMSYLESLN
KSGSFPDETDKTPKCFVRCVLEKSDIVSGDSQFNVTRTAEVFSQIRDTSQNDIIKMATACSDRPEKCKCERSYQYL
KCLLETTIEINEMKSSK
>HmelOBP13
MQGFIVHCSIVAILVVGASALEGEMAELAKMLRDSCIEETGADIALIDKVNDGADLMPDPKLKCYIKCVMETAG
MMSQGAVDVEAVVAVLPPELQRHADKLRACGTKAGANDCDTAFLTQACWQGGCKEDYILI
>HmelOBP14
MKTLKLLILLFVFFFGDSQGMTDDEMREEFMRITMICSKDYKVDMKDLLSLQQLNIPTKKDVKCLLACAYKKTG
SMNKEGLYDIEASYRIAEMTKNGDPKRLENAKKLVDICAKVNDETVSDGEAGCDRAGLIFKCVVENAPKVKNN
>Hmel-OBP16
MFLIVLIFQGYSDEERTKIYATMLPHILQCSTEYGITEDELKASKENEKFGSINPCFMGCIFKKIHVINKEGIFNVEK
AEKLSENFLVHDEDKKKASAVIKACATINDEDVSDGEKGCDRAKLLFECLLPFRQQVILKLIMCIEKATKT
>Hmel-OBP17
MTSLQLVFLVFGVVAVSLGSVSAFSDEERNKIYAGMLPLVLECSKDYGLTEDDLKAAKESGSIGSINPCLMACVFK
KINVINDKGLFDVDKAGELSQKFLTETDDQQKATEIIKTCASVNEKDVSDAEKGCDRSKLLFDCLLPFKGQVKYNI
VIILIIYSSR
>Hmel-OBP18
MTRQQLKNSSKLLKKNCMAKNDVTEDLVGDIEKGKFIEDQKVMCYIACIYQMSQLVKNNKLNVEASIKQVDM
MFPPEMKDATKASIENCKDVSKKYKDLCEASFWTAKCLYEDNPKNFFFA
>Hmel-OBP19
MTRPQLKKTLTIMKNQCMPKHRVTNEKVGQIEQGVFVEDHDVMCYIACVYKTAQVVKNKRLDKDLVSKQIDIL
YPAEIREAVKLSTVKCIPVQYNYEDECEGIFYSVKCLYEDNPANFIFP
>Hmel-OBP20
MTRQQLKNSGKLMKKSCMPKNDVTEDQIGQIEQGKFLEERNVMCYIACVYTMTQVVKNNKLNYEATIKQVD
LMFPPDMKDAVKAAVEHCKDISKKYKDICEVSYWTAKCMYDFDPNNFVFP
>Hmel-OBP21
MTMKQIKNTGKMMRKTCQPKNNVADEKIDPLNKGEFIEEKEVMCYVACIMKMANTIKNNKLNYEAAIKQAD
MLFPDEIKEPAKEAITACRKVVDDYKDLCESSFYTTKCIYNYNPSIFFFP
>HmelOBP22
MGNSIKKDEEQHAMFRIKFYYIVIFLIIFTAFILVMAFTPLTKDEQIDKFNKMNEGVEPFRRNLTECARQVKASMV
DVENFLKRIPQTSLQGKCFVACILKRNSIIKNNKILKENLLEANKAVYGEDSEVLSRLKLAINECSDVVANIFEICEFS
SVFNDCMHMKMEHILDKVIMERRMEALGQMTGDPDVWTDEEDELLKLVKDEL
>HmelOBP23
MQSAALLAAVFLALITFGFGQKEKPEFSEEIKEIIQHVHNECVAKTEVTEEDIANCENGIFKEDIKLKCYMFCLLEE

GSLVDENDNVDYDMMISLIPEQYTDRVSKMITACKHLDTPDKNKCQRAFDVHKCSYDSDPKVNF

>Hmel-OBP2

MSILLCFLFLSIALIDGKTVNIPVDPDITSQLVQISADCIASNGLTEEVLKQVMEWKLENNEPTKKLLFCFGTKLNT

TDKNGHVILNEALKLAVSKKRPVFGDAIKRCNDQEGSDKYDTLFKIIICMRDQENIFLRF

>HmelOBP9

MLVFILFSAHNMENKCDEDVSPTCVPSVDPIEECIEKLHVNRALVEKLKSGNSKLFDKNLKRWLLCFFEKTCVMT

PDGVLRQDVVLKDIPDQDKSKIEKITSICLYQKLHFAVDTAWNYLNCFREKDPKYSVIANKI

>HmelOBP10

MKTFIVLAICFVAAQALTDEQKDKLKKHKSECLAETKVDEKLVDKLKTGDFEIENEPLKNYTLCMLVKSGLMSMD

GEFKKDVALAKVPNAADKQLVGQLIDLCLANKGAAPEETAWNYSKCYHQKDAKHSIFQ

>Hmel-OBP11

MKTFIVLAICFVAAQALSDEQKDKLKKHRSECLAETKADEQLVNKLKTGDFKTENEPLKKYSLCMLIKSELMTKD

GKFKKDVALAKVPNAADKPAVEKLIDLCLANKGNTPHQTAWNYSKCYHEKDPKHSIFQ

>Hmel-OBP24

MRAVWCLLAVSLAVVSGKVLDTLVIVPPERVPGIVQASSKCIEEMNLDKDTMQKFFSWQLGDSESTRKYMYCL

GVKSGYIADDGSMVKKEVLGLAGSHGGNIDGVIDECNNLKYSDKYEAVFKIVMCFHEKSKLEFKV

>HmelOBP27

MDTGKMRAVWCLFLVTLATVYAGNVKVEYVDIPKDYIPAVEKASFECIKKLKLEEEQISLQGFLNWELSESDNTK

KYIFCLANGSGFFADDGSILKDKVLAIMGKYRDRVDKVIDECSKVKYDNKYEEVYRMEVCFRDLSGLYFRM

>Hmel-OBP28

MNTLWFFLFLSIALVKGKALFHVPPEYAGEILKAAADCIDSTGAGVDAVQKVISANLENTEPFKKFLYCFSSKSGYV

DSDGHFIVDQMTKLIGNHKDKAKFIDNLNLCNKSEGGNTIDTIYQIAVCFKDNSPIYFTV

>Hmel-OBP29

MKTFWCFLFLSIALVSGRAIVNVSQEFTGDILKTAVDCTDSSGAGVDALQKMASANFEDTEPFKKFLYCFASNSG

YVDSDGHFIMDKMTKLIGNHKDKAKYVDAINLCNKRKGGRTIDTIYELANCFKDHSPIYFTL

>Hmel-OBP30

MFDIPKEFVPDIIKASAECADKLGLDTLNILSKFFSGELKDSESVRQYMYCLGTTSGYVNDDGRLNKDRLGKVVG

EHKSKVESVVDECNKAKASDKYETVYKAVVCFRENSGLQFKI

>HmelOBP31

MLMRKNPICIYISIIYLGVQYVLVEYIKVPKEFIDDAINGSGQCAEELGLPGDTLNKLLSNNFEDSQAMRKYIYCLG

IALDVGDGTGSLKHSLSKYASNDRRKAEITKTIDECNKEKASDKYEKAYKVSTCYLNTSSVQFKV

>HmelOBP35

MKVLIICVGLFALIEAHNIHLSHGQKEKVKEYAAECMKESGAKPEVLADAKKGHLVDDEGLKKFILCFFQKTGVLS

SDAKLNTDVALSKLPAGIDKVTAAKVLNDCKNKKGATHADTAFEIFKCYYTHTKQHILFEK

>Hmel-OBP1

MSVRVLFIFIIVTACQANIQVSPPVTCGYLPRAIHECIGSPHIVKPEISAQCSKSISECERMTCVFQKSGWMSGNA

VDKDKVKSYFDQFSTDNPQWALAVNHVKAACLNMDLPSQGVYLNCPAYDILTCVFSGFIKNAQPDQWSSSES

CSYPRQFASACPYCPSDCFAAQVPIGSCNACLALPRSP

>BmorCSP1

MKCLTIAALLFVAGLSIAEKYTDKYDNIDVDEILENRKLLVPYIKCVLDEGRCTPDGKELKAHIKDGMQTACAKCT

DKQKVSARKIVKHIKQHEADYWEQMKAKYDPKDEFKEIYEGFLAGQN

>BmorCSP2

MKSVILICFLGVATVVIARPKTPFDNINIEEIFENRRLLLGYINCILERGNCTRAGKDLKSSLKNVLEENCDKCSEDQ

RKSIIKVINYLVSSEPESWNQLKSKYDPEGKYLIKYEAKMESN

>BmorCSP3
MNSLIAFCLFAVLAVALARPDDKYTDRYDNVNLDEVLSNSRLLQPYIKCILDKDRCAPDAKELKEHIREALETECA
KCTEAQKKGTRRVIGHLINNESKSWNELTAKYDPENKFTAKYEKELREIKA
>BmorCSP4
MKVLIVLSCVLVAVLADDKYTDKYDKINLQEILENKRLLESYMDCVLGKGKCTPEGKELKDHLQEALETGCEKCTE
AQEKGAETSIDYLIKNELEIWKELTAHFDPDGKWRKKYEDRAKAKGIVIPE
>BmorCSP5
MKTVIVCLLALTAVALARPEQYTDKYDTVDLDQLISNRRLLIPYVHCILEKGQCTAEGKELKSHIKEALETNCAKCT
KAQKGGTEKMIGHLINHEAEFWEELKAKYDPTNEFTKKYETELKRVTA
>BmorCSP5a
MNSLIAFCLFAVLAVALARPDDKYTDRYDNVNLDEVLSNSRLLKPYIKCILDKDRCAPDAKELKEHIREALETECAK
CTEAQKKGTRRVIGHLINNESKSWNELTAKYDPENKFTAKYEKELREIKA
>BmorCSP6
MKSLIVLSCLLAACLAADLSKYENFDVEPIVTSDRLLKAYINCFLDKGRCTPEASDFKKALPDTIATNCGKCTEKQK
ANVRKVIKVIQQKHSTEWEKLVKKHDPSGKHRADFDKFLLGS
>BmorCSP7
MKGFYVLCFALFAAVYCKETYSSENDDLDIEALVGNIDSLKAFIGCFLETSPCDAVSGDFKKDIPEAVAEACGKCTP
AQKHLFKRFLEVVKDKLPQEYEAFKTKYDPQGKHFDALLSAVANS
>BmorCSP8
MKTILILCALVSVVVCRPEEYYSSQYDNFDVEQLVGNLRLLKNYAKCFLDQGPCTAEGTEFKKRIPEALRTKCAKC
NPKQRHLIRTVVKAFQTKLPDLWEELAIKEDPKGQYKHEFTAFINAMD
>BmorCSP9
MKFVLALIALAVVVAARPNDDLFYDKKYDNFNVDEIIDNPRLLKAYTFCFNDKGKCTAEGNDFKKWIPESLQTSC
GKCSEKQKYLVAKFVHAIKDKMPDEFDILRKLHDPKGEYTENLDKFLETYGH
>BmorCSP10
MKSSLFCVLVLTVVVSSSRQQSYPRNDNININAILQNDRILLGYFKCVMDRGPCTKDGKTFKRALSEALPTACAR
CSNKQKAAFRTLLLAIRARSEPSFLELLDKYDPSRSNRELLYTFLATGL
>BmorCSP14
MKSSLFCVLVLTVVVSSSRQQSYPRNDNININAILQNDRILLGYFKCVMDRGPCTKDGKTFKRALPEALPTACAR
CSNKQKAAFRTLLLAIRARSEPSFLELLDKYDPSRSNRELLYTFLATGL
>BmorCSP14a
MACVAVTWARPESTYTDKWDNINVDEILESNRLLKGYVDCLLGKGRCTPDGKALKETLPDALEHECVKCTGKQ
KSGADKVIRHLVNKRPDLWKELAVKYDPDNIYQARYKDKIDAVKGSA
>BmorCSP11
MKLTSFLLVGMAMVSAEFYSSRYDDFDVKPLVENDRILQSYTNCFLDKGPCTPDAKEFKKVIPEALETTCGKCSP
KQKQLIKTVIKAVIERHPEAWEELVNKYDKDRKFRPSFDKFINEDD
>BmorCSP9a
MRAVIFLYTCVFVVVGQDINAMMSMPKYDERYDYLDVDDIFRNKRLVRNYVDCLINAQRCTPEGKALKRILPE
ALRTKCIRCTERQKRTSVKVIRRLKNEYPEEWAKLASRWDPTGDFTRYFEDYLAKEHFNTIPGSGPTVNVLSLQT
TPPPPPPPPSRPASVFTNPPPPVMSTSPRPVVLNRFRR
>BmorCSP13
MKLLLVFLGLFLAVLAQDKYEPIDDSFDASEVLSNERLLKSYTKCLLNQGPCTAELKKIKDKIPEALETHCAKCTDK
QKQMAKQLAQGIKKTHPELWDEFITFYDPQGKYQTSFKDFLES
>BmorCSP12

MFMLFIISFIIVPVLKCCGTETSTYTTQYDEVDIKEIMGNERLLVAYIGCLLDKNPCTPEGKELKRNIPDALQSDCSK
CSDKQRENADAWIEFMIDNRPEDWTKLEER
>BmorCSP15
MIENFYSKCTISKSVLFLCLIFLPYALNQKYYDSRYDYYDIDHLVQNPRLLKKYLDCFLGKGPCTPIGRLFKQVMPE
VITTACAKCTPTQKRFARKTFNAFRRYFPETLMELRRKFDPESKYYDAFEKVITNA
>BmorCSP16
MIEWKRFKILHFLSYLGLLVLVVVCAAQQNRPQVTDTALDEALNDKRFIQRQLKCALGEAPCDPIGKRLKTLAPL
VLRGACPQCSPQETKQIQKTLSYVQRNFPQHWAKLVRQYAG
>PxutCSP1
MNSLLLFSLLTLFVVAFANEQYTDRYDNINIDEILSNKRLLTSYIKCILDKGRCTPEGKELKLHIKDGMQNSCSKCTD
FQKNGARKVVKYIRANEKESWEEMKKKYDPKDEYKEKYEAFLAADN
>PxutCSP5
MKLLPILVIAACVVMVKAGTYTDRYDSMNVDDVIANKRLFIAYVKCILNKGRCTPEGKELKSHITEALQSGCDKCT
ARQRRSIRKAIKHLIHKENNYWNQLVNMYDPNKMYSKMYERELGTI
>PxutCSP7
MKLIVLVLCVTALAYAEDKYEDIEDNFNLQELLENDRLLTGYIKCLLNKGPCTPEVKKIKEKLPEALATNCAKCTDKQ
KQMGKVLVKQVKKAHPELWDELKNLYDPQGKYQKEFQQFLSD
>PxutCSP3
MKTLVVLACVLLSVYAADKYNSKYDNFDVETLITNERLLKSYINCFLDKGRCTAEGTDFKKALPEAIETTCGKCTEK
QKLNIRKAIRAIQQKYPGQWEDLVKKNDPSGKHRANFDKFIQGS
>PxutCSP2
MKLFMVCALLCVAAVAWGKPASTYTDKWDYINVDEILESQRLLKGYVDCLMDRGRCTADGKTLKETMPDALE
HECSKCTEKQKESSDKVIRFLINKRPELWKELATKYDPDNVYQQRYKDKIEAVKEH
>PxutCSP10
MKLILMILASTIALVQGDATKQRYDAFDIQTALQNDDIILSLINCFGDTTPCSPEMKAFKNDIPTALQTACGKCSD
RQREVIRHVIRTVMKKYPDAWTYLIDKYDPENKYRDGFYQFIGQDD
>PxutCSP6
MKTIILLCTLAAAALAAPADTYNSQYDNFDATELVGNTRLLKSYGRCFLGQGPCTAEGSDFKKTIPEALRTTCAKC
TPKQRELVRVVVRGFQTKLPEIWEELVKQQDPKGEFKEAFDRFLNSSD
>PxutCSP4
MNTFLMVCLLALVAAVSADQYTDRYDNVDLDEILSNRRLLVPYLNCILEEGKCSPDGKELKSHIKEALENNCGKC
TETQKSGSRKVIGHLINNEKEYWGKLTAKYDPERKYVTKYESELRKIAA
>PxutCSP8
MKSKAALVLMCVLAAALAESYSDKYDNIDLQEIADNDRLLDAYANCLLEKGKCSPEGKELKGHMKDAIETGCEK
CTDAQKKGTNFMIDHLIRKKPEIWNQLANKYDPTGKWRKVYEDRAKEHGIVIPH
>PxutCSP8a
MVSRNLMILCCLVVAVVAKPTYTDKYDNIDLEEFKENKRLLLAYVDCILDKGKCTAEGKALKDNLLDATETGCEKC
TEKQKEGSYEMIEHLIKNEPEIWNELCAKYDPTGKWRKEYEEKAKAKGIKIPQKISTK
>PxutCSP9
MQINHIVIALCALAATCLAQAQTDRPPVSDTALEEALNDKRFIQRQLKCALGEAPCDPIGKRLKTLAPLVLRGACP
QCTPQETKQIQRTLSYVQRNFPQQWAKIVRQYSG
>PxutCSP8b
MASKSLIILLCVVASVWCTYTNKYDNINYEEVVSNEKLLNKYADCILDRGRCTAEAKELKEHLKEAIENGCKECTEK
QKEGTNYVIRYLIENKRNLWEELCAKFDPDGKWRKHYEEEAKAKGIKIP

>PxutCSP13
MRQWLLCVFALTVVVSCSSQQQYVNRYDNFNADSIIQNERILLAYYKCVMEKGPCTKDGKNFKRVLPETISTAC
GRCSPKQKVVVRKLLLGIRAKSEPRFFELLDKYDPSRLNREALYTFLVTGN
>PxutCSP11b
MKCLLLLPMLMALVAAETYSTENDDLDIEAVVGDLNTLKSFMDCFNDKKPCDAVQADFKKDLPEAFQNACAK
CTAAQKHIFKRFLEESEKKVVDDLNALKKKYDPESKHYAALLSAISKS
>PxutCSP12
MVFLSLVLAMLIPTALSYDAAYDKVDANKVLADDALFRSYVDCFLDRKPCTADFSEEFKKILPEVIKEACAKCSEP
QKKNVRKIVKALYEKYPDDAIKFADKYDPKRDYESAFAAFVAEE
>PxutCSP11a
MKTIVALTVLVGLATTALAYSTEHDDLDIAAIVADKNELQKFIDCFTEKAPCTKLTQDFKTKLPEVVREACEKCNPT
QKQKLKIFLDGLNEKFPEEYNALKKQFDPTGKLFEALNAALAKA
>PxutCSP4c
MKVLVCFLLALVIIAQARSNRKYTSRYDNIDLDDVLANRRLLVSYIKCLLDLGKCSSEGKELKSHINDALEDNCAQC
TDVQKSGFRRVIGHLINHEKDYWGKLTAKYDPERKYVLKYERDLREISA
>PxutCSP4b
MAIATAMPSPSETYTDRFDNIDLDEIIGNRRLLVPYIHCVLDKGKCTPDGKELKSHIAEAIENDCAKCTEVQRKGT
RKVLGHLINNEQEFWDELTARYDPEHKYSVKYENELRTSK
>PxutCSP4a
MKSVVLLCLFVVLATAVAGPAGQYTDRYDSINIDEILGNRRLLIPYVHCLLDQGKCTPEGKELKSHIKEALENNCAK
CTPPQRTRSRQVIGHLINHEPTFWNELCQKYDPTSKYTRKYEEELRTIAA
>BmanCSP3
MNSLIAFCLFAVLAVALARPDDKYTDRYDNVDLDEVLSNSRLLQPYIKCILDTDRCAPDAKELKEHIREALETECAK
CTEAQKKGTRRVIGHLINNESESWNKLTAKYDPENKFTAKYEKELREIKA
>HarmCSP26
MDSRIAVVCVVLAAFAVDQTVGAPQKDAVAASGPAYTTKYDHIDVDQVLASKRLVNSYVQCLLDKKPCTPEGA
ELRKILPDALKTQCAKCNATQKNAALKVV
>HarmCSP17
MKYAIIFAVVALATIVYAVPRPDGEKYPTKYDNVDVDSILANDRLLKNYIDCIEGTGKCTPEGDELKRHLKDAIQN
CCEKCSDKQKDGVKKVFKHLEDKKP
>HarmCSP
MKVLLVLCLFAAAALADDKYTDKYDNINLDEILENKRLLLAYVNCVMERGKCSPEGKELKEHLQDAIETGCSKCTE
AQEKGAYKVIEHLIKNELDIWRELAAKYDPKGDWRKKYEDRARANGIQIPE
>HarmCSP16
MKILVLLLAAVVTAQYEEDTYGTDHDDLDIVALVEDKDQFNSFIDCFIDEAPCDDVAETFKSVIPEAVLEVCAKCTP
AQKHIVRVFNESFKKKMPEKFQKFKNKYDPEGKYFENFEAAVGAF
>HarmCSP8
MKCIYVLSFLLALAAVQAEDKYSTENDNLDIDAVVANVDTLTSFVACFVDQEPCDAVAADFKKDIQEAVTTRCAK
CTDAQKHIFYKFILGLKEELPRGYEEFGRKYDPENKHFSALENAVSPA
>HarmCSP21
MKSLLLLCLVIAAVWARPETYDTRYDDFDAETLVENVRLLKAYGHCFLGTGPCTPEGSDFKKTIPDALRTGCGKCT
AKQRHLIRVVVQGFRSKTPDLWQQLVKKEDPNGQYKEVFTRFLNGSD
>HarmCSP19
MNSLIVFCVLSLAALTIARPDGATYTDKYDNVDLDEILGNRRLMVPYIKCMLDQGKCAPDAKELKEHIREALENG

CAKCTDKQKEGTRRVIAHLIKHKNADWQKLKAKYDPEGKYTHKYEKELEEVQH
>HarmCSP10
MKVLVVLSCLIVAAFAADKYNAKYDNFDVDTLITNDRLLKAYINCFLDKGRCTPEGSDFKKTLPEAIETTCGKCTD
KQKNNIRKVIKAIQQKHPKEWDALVKKNDPSGKHRANFDKFIQGSR
>HarmCSP20
MKLIVAVALLCLVAESWAASTYTDKWDNINVDEILESQRLLKAYVDCLLDRGRCTPDGKALKETLPDALENECSK
CTDKQKSGSDKVIRHLVNKRPEMWKELSAKYDPNNIYQDRYKDKIEAVKGQ
>HarmCSP22
MQTRYAVVLCCVVAACVAQTQRPPVSDSALEDALQDKRFIQRQLKCALGEAPCDPIGKRLKTLAPLVLRGACPQ
CTPQETKQIQRTLSYVQRNFPQQWAKIVRQYAG
>HarmCSP14
MNSAIVLCVVALAGMVLARPDGDGDKYTSRWDDVDLDEILENDHLLIPYIKCSLDEGKCAPDAKELKEHIQEAL
ETGCAKCTDKQKEGTRRVIAHLIKKKLQEWEKLKAKYDPEGKYAKKYEKELEEVKNA
>HarmCSP23
MKAVFLLCLVVVAVSARPEAQYTNKYDNVNLDEILVNKRLLVPYIKCALDQGKCSPDGRELKSHIREALENYCAKC
TPVQQDGTRRVIAHLIKHKLEEWEKLKAKYDPEGKYTHKYEKELEEVQH
>HarmCSP18
MKLLVVAIVVTLAAFAVAETYTDRYDNINIDEIIENRKLLVPYIKCVLEQGRCTPEGRELKAHIKDALQTSCTKCTQK
QRKASRKVVKHIRANELDYWKQLLAKYDPDSIYVKNYESFLAADD
>HarmCSP9
MNSLIVFCVLSLAALTIARPDGATYTDKYDNVDLDEILGNRRLMVPYIKCMLDQGKCAPDAKELKEHIKEALENE
CGKCTEAQKKGTRRVIGHLINHEADFWNELAAKYDPERKYTTKYEKELKEVEA
>HarmCSP12
MNSAIVLCVVALAGMVLARPDGDGDKYTSKWDNIDLDEILGNDRLLVPYIKCALDEGKCAPDAKELKEHILEAL
ETGCDKCTDKQKEGTHRVIAHLIKYKLEEWEKLRAKYDPEGKYAKKYEKELEELKRA
>HarmCSP11
MNSAIVLCVVALAGMVLARPDGGTYTTKYDNVDLDEILANDRLLIPYIKCLLDEGKCAPDAKELKEHIREALENG
CAKCTDKQKEGTRRVIAHLIKHKNADWQKLKAKYDPEGKYTHKYEKELEEVQH
>HarmCSP24
MKTAIVLLLALFGVVLTARFDNIDIDKVLGNQRVLESYLKCMYDEGPCTPEGRDLREKAPEALETNCKDCTDNQK
ALVRKASLFLIKNRPDDWKKLSDKFDPEGKYKKAFDEFLKEKN
>HarmCSP13
MKVLLVLCLFAAAALADDKYTDKYDNINLDEILENKRLLLAYVNCVMERGKCSPEGKELKEHLQDAIETGRSKCTE
AQEKGAYKVIEHLIKNELDIWRELAAKYDPKGDWRKKYEDRARANGIQIPE
>HassCSP
MKVLLVLCLFAAAVLADDKYTDKYDNINLDEILENKRLLLAYVNCVMERGKCSPEGKELKEHLQDAIETGCTKCTE
AQEKGAYKVIEHLIKNELDIWRELAAKYDPKGDWRKKYEDRARANGIQIPE
>AipsCSP1
MKAVIVLCALVVAVCARPEEEKYPDKYDNTNYKEILENDRLYRAYCDCLLDAGKCTPEGKELKSRIKDALETKCEK
CTDKQKEAVRYVIKYLINKKPEDWKKVCDKYDPDGKFKSQYEKELKDL
>AipsCSP2
MKIILALCVLVAAVSAYDTRYDDFDVETLVGNVRLLKSYGHCFLGTGPCTPEGTDFKKTIPDALQSGCGKCSPKQK
HLIRTVVKGFQTKTPDIWQQLVKKEDPHGEYKEIFTRFINGSD
>AipsCSP3

MNSFIVLCIASLAVMAYARPEEAKYTDRYDNVDLDEVLSNRRLLVPYVKCILDQGKCAPDGKELKEHIREALENEC
GKCTETQRKGTRRVIAHLINNEADYWNELTVKYDPQRKFTAKYEKELKEIKQ
>AipsCSP4
MKVVLLTLCFALGVLAQDKYESVNDDFDVSKVLNNDRLLQSYAKCLLNKGPCTSEVKEVKAKLPEALETRCAKCT
DKQKQMGKVLAQEVKKNHPDIWKELVAMYDPQGKYQEAWKEFLQE
>AipsCSP5
MQIKYALLLCCVAAMSVAQTQRPAVSDTALEDALQDKRFIQRQLKCALGEAPCDPIGKRLKTLAPLVLRGACPQC
TPQETKQIQRTLSYVQRNFPQQWAKIVRQYAG
>AipsCSP6
MKLIIAVALLCMVAASWGKPASTYTDKWDNINVDEILESQRLLKAYVDCLMDRGRCTPDGKALKETLPDALENE
CSKCTEKQKSGSDKVIRHLVNKRPDLWKELSTKYDPDNIYQDKYKTQIESVKQ
>AipsCSP7
MKFVLLLCVMVAVVYAEDKYTDKFDNIDLDEILTNRRLLLSYFNCVMGKGKCTAEGKELKDNLEDAIKTGCAKCT
ENQEKGSYRVIEHLIKNELDLWRELCAKFDPTGEWRQKYEDRARANGIEIPKD
>AipsCSP8
MNFLVLSMVIALAGFVAAETYTDRYDHINIDEIIENRKLLVPYIKCTLDQGRCTPEGRELKAHIKDAMQTSCSKCT
PKQRKGARKVVKHIRAKEQEYWNQILAKYDPENQYSENYEAFLAADD
>AipsCSP9
MMMHSTLSMLLVLYLTVQSKAIETPTYTTKYDGIDLDEILNNERLLTGYVNCLMDLGPCTADGKELKKNIPDAIE
NDCKKCTDRQREGSDRVMHYLIDHRPDDWVKLEEKYNSDGSYKMKYLSSKPIEETKESNVTKSDEDTKDAAKE
>AipsCSP10
MKAECVLLATLTVAVAADFYSSKYDSFDVQPLLENDRILLSYTKCFLDQGPCTPDAKDFKKVIPEALQTTCGKCTP
KQKQLIKTVIKEVIARHPEAWEQITDKYDKDRTYKESFDKFLAEE
>AipsCSP11
MRAVLLLCVFVYTVVGQDVNQMENLPKYDSRYDYLDIDAIFTNKRLVRNYVDCLINAVRCTPEGKQLRKILPEAL
RTKCIRCTERQKRTAVKVIKRLKYEYPDEWAKVSSRWDPTGDFTRYFEEFLAKESFNSIPGSGIAIPTSQPLAPPAP
TMPPTVASPAPAPTEPTPPRPVVLNRFGDGEIMQGSPSSAGVTPSPMTQATTRPTTTMRPTMRPSMRPTMT
QPATNPTRPTIMTMTWAGAASNTQPTRFPLRPVSDLPTPYSTAITIIDQIGIKIIRTTELFTDILKNTVRAVVGR
>SlitCSP1
MKSFIVLCLFGLAAVALAKPNGSTYTDRYDNVNLDEILGNRRLLTPYIKCILEEGKCTADGKELKSHIREALEQNCA
KCTDAQRSGTRRVLGHIINNEEESWNRLKAKYDPESKYTVKYELELRKLKQ
>SlitCSP2
MKSMIVLCVLSVAALVVARPDDSHYTDRYDNVNLDEILSNRRLLVPYVKCILDQGKCAPDAKELKEHIREALENE
CGKCTETQKNGTRRVIEYLINNEEEYWNELTVKYDPERKYTAKYEKELKKIKA
>SlitCSP3
MKVVFLVCVLAAVVYAHPHESHYTDKWDNIDLDEILNNKKILASYVKCCLDQGKCTPDAKELKSHIKEALENRCG
KCTPAQKDGTRKVLTHLINYEPEMWNQLCEKYDAEGKYRKMYEDEYKSVKH
>SlitCSP4
MKFVLVLCLMAAAVLAEDEKYPSKYDNIDLDEILANKRLLTAYVNCIMERGKCSPEGKELKEHLVDAIETGCSKCT
EAQEKGAYKVIEHLIQNELDTWHELTDKYDSSGKWRKTYEDRARANGIVIPE
>SlitCSP5
MKVALLTLCFALGVLAQDMYENANDNFDISEVLGNERLLNSYAKCLLNKGPCTPEVKQVKDKLPEALETRCAKC
TDKQKQMGKTLAQEVKKNHPDIWKQLVAMYDPEGKYQQAWKDFLQE
>SlitCSP6

MQIKYALVLCCVAAVSLAQTQRPPVSDTALDDALQDKRFIQRQLKCALGEGPCDPIGKRLKTLAPLVLRGACPQC
SPQETKQIQRTLSYVQRNYPQQWAKIVRQYAG
>SlitCSP7
MKADCLLIMTLMAVVAADFYNSKYDSFDVQPLLENDRILLSYTKCFLDQGPCTPDAKDFKKIIPEALETTCGKCS
PKQKQLIKKVIKAVIAKHPEAWEQLSDKYDKDKKYKDSFDKFLAEKE
>SlitCSP8
MMKKSFITMMLLIYLTIQSNATETSTYTSKYDGIDLDEILSNDRLLTGYVNCLMDLGPCTADGKELKKNLPDAIEN
DCKKCTERQREGADRVCHYLIDNRPEEWTKLEEKYKSDGSYRAKYLASKEATDEKASNVTNSNEDTNNVSKE
>SlitCSP9
MNFLVLSIVVTMAAFVAAETYTDRYDHINIDEIIENRKLLVPYIKCTLDQGRCTPEGRELKAHIKDAMQTSCSKCT
EKQKKGARKVVRHIRAKEQEYWKQILAKYDPEDQYKENYETFLAAED
>SlitCSP10
MRVVLLLCALVHLVVGQDVNDMVNMPKYDQRYDYLDVDAIFTNKRLVRNYVDCLINAVRCTPEGKALKRILPE
ALRTKCVRCTERQKRTAVKVIKRLKNEYPDEWSKLASRWDPTGDFTRYFEEFLAKEHYNTIPGSGSALPTSSPLA
PPRVPQSPPATNPTPGPTEPTPPRPIVLNRFGDDGELMMGSPSSAGITPRPMTQATTRPSTTTRPPSTRPVPPR
PTMMTWAGAASNTQATRFPLRPVSEISPPYSTAITLIDQIGYKIIKTTELVTDLLRNTVRAVVGR
>MbraCSP1
MKFVLLLCVMVAAVVAEDKYTDKYDNINLDEILANKRLLVAYVNCVMERGKCSPEGKELKEHLQDAIENGCKKC
TENQEKGAYRVIEHLIKNEIEIWRELTAKYDPTGNWRKKYEDRAKAAGIVIPEE
>MbraCSP2
MKSCIVLCVLSVAVMALARPEEAHYTDRYDSVDLDEILGNRRLMVPYIKCILDQGKCAPDAKELKEHIREALENE
CGKCTETQKNGTRRVIGHLINHEDAYWKELTAKYDPQSKFTAKYEKELKEIKH
>HvirCSP1
MKFIVAVALLCLVAESWAASTYTDKWDNINVDEILESQRLLKAYVDCLLDRGRCTPDGKALKETLPDALENECSK
CTEKQKAGSDKVIRYLVNKRQDLWKELSAKYDPNNIYQDRYKDKIEAVKGQ
>HvirCSP2
MALARPDGAAYTDKYDNVDLDEILSNRRLLVPYVKCILDQGKCAPDAKELKEHIIEALENECGKCTEAQKKGTRR
VIGHLINNEADYWNELTAKFDPEKKYVQKYEKELKEVKA
>HvirCSP3
MKVLVVLSCLVVAAFAADKYNAKYDNFDVDTLITNDRLLRAYINCFLDKGRCTPEGSDFKKTLPEAIETTCGKCTD
KQKNNIRKVIKAIQQKHPKEWDDLVKKNDPSGKHRANFDKFIQGSR
>HvirCSP4
MKFLLVLCVMAAVALADDKYTDKYDNINLDEILENKRLLLAYVNCVMERGKCSPEGKELKEHLQDAIETGCSKCT
EAQEKGAYKVIEHLIKNELDIWRELTAKYDPKGDWRKKYEDRARANGIQIPE
>HvirCSP5
MNSLIVFCVLSAAAMALARPDTYTDKYDSVDLDEXLSNRRLLVPYVNCLLEQGKCAPDAKELKEHIREALENAC
GKCTEAQKNGTRRVIGHLINKEPEFWNQLKAKYDPNNKYTQKYEKELKEVQEDKQ
>HvirCSP6
MKFLVVAILVTMAAFVVAETYTDRYDNIDIDEIIANRKLLVPYIKCILDQGRCTPEGKELKAHIKDALQTSCTKCTD
KQKKASRKVVKHIRAKEQDYWKQLLAKYDPDSQYVQNYESFLAGDD
>HvirCSP7
MKAIFLLCALVVAVHARPEQYTAKYDYVDYKEPVANRRLLVAYIKCILDQGKCTPEGTELKSHISDAMKTNCSKCT
PKQREGTRYVIAHLIKHEPEYWKQLCDKYDPEGKYASKYEKELKTDS
>HvirCSP8

MKILVLLLAAVVTAQYEETYGTDHDDLDVVAVVSDKEQFNSFIDCFIDEGPCDEVAATFKSVIPEAVLEACAKCTP
AQKHLVRVFNESLKKKMPEKYQKYKNKYDPEGKYFVNFEAAVGAF
>HvirCSP9
MKADCLLIVTLVAVVAADFYNSKYDSFDVQPLLENDRILLSYTKCFLDQGPCTPDAKDFKKVIPEALQTTCGKCSP
KQKQLIKTVIKAVINRHPDAWNQLIEKYDKDKNYKESFDKFLAEQD
>HvirCSP10
MKVVLITLCFALGLLAQDQYESANDNFDISEVLTNDRLLQAYANCLLNKGPCTPEVKQVKEKLPEALETRCAKCT
DKQKQMGKTLAQEVNKNHPDIWKQLVSMYDPQGKYQQAWKDFLQE
>HvirCSP11
MKCVYVLSFLLAVAAVHAEDKYSTENDDLDIDAVVANLASLREFVGCFMDAVTCNAIAADFKKDIPEAVSTQCA
KCTDAQKHIFYKFLLGLKEKLPNDYQAFKIKYDPDNKHFSALEDAVSGA
>HvirCSP12
MNSAIVLCVVALAGMVLARPDGDGDKYTSKWDNVNLDEILANDRLLVPYIKCVLDEGKCAPDAKELKEHIKEAL
ETGCAKCTDAQKDGTRRVIAHLIKKKVEEWEKLKAKYDPEGKYSQKYEKELEEVKNA
>HvirCSP13
MKAVFLLCLVVVAVSARPESQYTNKYDNVNLDEILVNKRLLVPYIKCALDQGKCSPDGKELKSHIREALENYCAKC
TPVQQDGTRRVIAHLINNEPDYWTQLSAKYDRDGKFALKYEKELRTIA
>HvirCSP14
MTGFFLGVLTQNHDLYAINSFHSAEILLLDVLLRAYAIWQIIIGPCTPNIKHVNQHMPKILKARSVKRIYWKNIMV
KILTHNVSTNHPDI
>SexiCSP1
MKSFIVLCLFGLAAVAMARPDGSTYTDRYDNINLDEILGNRRLLTPYIKCILEEGKCTPDGKELKSHIREALEQNCA
KCTDAQRNGTRRVLGHLINNEEESWNRLKAKYDPQSKYTVKYELELRKLKQ
>SexiCSP3
MKVVFLVFVLTAVVYSHPHDSHYTDKYDNIDLDEILNNKKILTSYINCCLDLGKCTPDGKELKSHIREALENKCGKC
TEAQKNGTRKVMTHLINFEPDYWNQLCAKYDPEGKYKAMYEKEYKTLVH
>SexiCSP2
MKSMIVLCVLSVAALVVARPDDSHYTDRYDNVDLDEILSNRRLLVPYIKCILDQGKCAPDAKELKEHIREALENEC
GKCTETQKKGTRRVIEYLINNEEEYWNELTAKYDPERKYTTKYEKELKKIKA
>HzeaCSP
MKVLLVLCLFAAAALADDDKYTDKYNNINLDEILENKRLLLAYVNCVMERGKCSPEGKELKEHLQDAIETGCSKC
TEAQEKGAYKVIEHLIKNELDIWRELAAKYDPKGDWRKKYEDRARANGIQIPE
>PxylCSP4
MQTVTLLCLLAAVAAAAAAPADTYDAKYDSFNAHELVQNQRLLKSYGKCFLSKGPCTAEGSDFKRVIPEALKTTC
GKCTRKQRELVRVVVKGFQEQLPQVWTEIVSKEDPKGEYKDSFAKFLEGSD
>PxylCSP3
MNSLVLVCLALVAVAAARPQATYTSKYDGVNVDEILANDRLMMPYIKCALDHGRCSPEAKELKSHIKEALENNC
AKCTDKQKPAVRKVIAHLINHKPAEWRQLSDKYDPAGKYTAQYEDQLRAVKA
>PxylCSP5
MKVVFLVFVLTAVVYSHPHDSHYTDKYDNIDLDEILNNKKILTSYINCCLDLGKCTPDGKELKSHIREALENKCGKC
TEAQKNGTRKVMTHLINFEPDYWNQLCAKYDPEGKYKAMYEKEYKTLVH
>PxylCSP1
MKSAAFIALFLIGKAVCEDKPTYTTKYDNIDLDEILSSERLLTGYVNCLLDQGPCTPDGKELKHTLPDAIDNDCRKC
TQKQKEGSDRVMGYIIEYRPNDWAKLEKKYLSDGSYKKKYLEKKNASENNGDSKSTEAKNKDDEEKKSKGDGE

EK
>PxylCSP2
MQKLTLACLLVAVAAAAARPNDSHYTDRYDNVNLDELISNRRLLVPYVKCVLDQGKCSPDGKELKEHIQEALEN
NCGKCTDKQREGTRKMIGHLINHEQEFWDQLIAKYDPERKYVSKYEKELKEVKASCCWLV
>PxylCSP
MQKLTLACLLVAVAAAAARPNDSHYTDRYDNVNLDELISNRRLLVPYVKCVLDQGKCSPDGKELKEHIQEALEN
NCGKCTDKQREGTRKMIGHPINHEQEFWDQLIAKYDPERKYVSKYEKELKEVKASCCWLV
>CfumCSP4
MRSCIVFACLLVSVFAAEKYNSKYDNFDVETLISNDRLLKSYVNCFLDKGRCTPEGTDFKKTLPDAVETTCAKCTD
KQKTNIKKVIKAIQTRHPRQWDELVKKNDPTGKHIVNFNKFIES
>CfumCSP2
MKSILYLVLTVVVTCSAQQQYYNNRYDNLNADSIVQNERVLLAYYKCVMDKGPCTKDGKNFKRVLPETLSTACA
RCSPKQKGLVRTLLLGIRVKSEPRFNELLDKYDPDRSNRDDLYKFLVTGN
>CfumCSP3
MKLLLVSLLACLVAVAFGRPQNKYTDKWDNINIDEILESQRLLKAYIDCLLDKGRCTPDAKTLKDTLPDALENECN
KCTEKQKSGSDKVIRHLVNKRPEMWKELSVKYDPDHIYEGRYKDQIEKIKA
>CsupCSP1
MRTVIFLSLLLVAIVIAEDKYDSIGDNININELLENDRLLKSYVKCLLNKGPCTPEVKKVKDTLPEALATRCAKCTER
QKQIGKQLAKEVKKRHPELWNEMIAFYDPEGKYQDAFQDYLKP
>CsupCSP2
MPSVFTYMIMALTLVAGEFYDAKYDDFDIEPLLENDRILQGYTKCFLDEGPCTPEAKDFKKVIPEALETSYGKCSP
KQRILIKKVIRAMMEKHPDSWNKLVDKYDKDKKFRATFNKFIEEED
>CsupCSP3
MKSMTVFCMLAFVAVAYALPQQYTDKYDSVDLTEILSNRRLLVPYLNCILTTGKCSPDGKELRSHIKEALENYCAK
CTETQKNGTRRVIGHLINNEADYWKKLVDKYDPERKYVVKYEEELKTVAA
>CsupCSP4
MPKYDRRYDYLDLDTLFLNKRLVGNYVDCLINAQRCTPEGKQLKRILPEALRTKCIRCTETQKKTAVKVIKRLKYEF
PEEWAKLSSRWDPTGDFTRYFEEYLDNENFNTISGSIDNNESVPSSRPAPVPVAPTLAPLPPVPPKLPNVPVTRP
SLPPPTQASTQRIPDATTSVKPVILNRFETTRADDGQPLIC
>DpleCSP
MKTFVVFAMCLAAALALPADTYNPEYDNFNAKELVENPRLLKNYGKCFLDQGPCTAEGSDFKKTIPEALRTTCA
KCTPKQRELIRTVVRGFQKDLPEMWSELVKKEDPKGEYKESFEKFLNGSD
>DpleCSP1
MDQGRCTPEGNTLKVHVTDAIQNSCSKCTEIQKTKARKVVNYIRENNKDVWDELIKKYDPKDEYKEKYEAFLE
GKF
>DpleCSP1a
MDSPYGCRVHRVLQGEASTVPGTSDPGVVSMKTLTPFSLFFMAKLCLGDNSYTTKYDGINLDEILASHRLLTGYI
NCLLDKGPCSPDGKELKNNLPEAIDNDCHKCTQRQKEGADKVMHFIIDNRPEDWDKLEEKYNSDGSYKLKYLT
TKLSENSEDVSKNYDEDDEEVNESRDHTSDNDHDTEMENH
>DpleCSP2
MDLFPISVLLVACFTAINTQTYEKRYDSVNIDDVLSNKRLLAAYVKCVLDQGRCTPEGKELKSHIADALQSGCDKC
TETQKDGVRKVIKHLIKNERDYWKQLVEKFDPEGVYAEKYEDEIRDV
>DpleCSP3
MFDNIKALSLSAAASSLGCIESKRIIKMRTFVVLACLVVSILAAEKYNSKYDNFDVETLVSNDRLLKSYINCFLEKGK

CTAEGTDFKKALPEAVETVCGKCTDKQKVNIRKVIKAIQQKHPTQWEELVKKNDPTGQHRANFEKFIQES
>DpleCSP4
MGQPEGCNLSDQKMILKSCVVLCCVLVCVLGDEKYTDKYDNVNLQEILENKRLLEAYVNCILDKGKCSPEGKELK
EHIQDALETGCEKCTDKQMEGTTTMIDHLVKHERAMWKELTDKFDPKGIWRKKYEDRAREKGIVIPED
>DpleCSP5
MKIFILLLCVFAAVIAEENSDLSTENIDLTEVVGNDKLIESYANCLINKGPCTPEMEKIKGLLPEAIATKCSKCTEKQK
EVGSQLIKGVREKHPEIWKKLTTYFDPEEKYRESVEEFLKA
>DpleCSP6
MKSCVLLALLSLVAVAWGRPQHYTDKWDNINVDEILESQRLLRGYVDCLVDKGRCTSDAKTLKETLPDALENDC
KKCTEKQKTSADKVIRHLVNKRPDLWKELAGKYDPKDIYQQKYKNKIEAVKEKH
>DpleCSP7
MYHRYIWFEVCDTTLTSRKNARATNPVQSGPQSSWKKLDREMLLEVVLVQSWLSASEDVEVEVEALWFRISM
QRLNLEVLLNLMFKMKSVMILALFAFVAADKDYYVLDKIDLSKIENNIEELKIFMDCVLDKGTCSDLYNSYKVHID
ESFKTACGKCTPEQKQFVSQFFKLYRKVLPQDIEELINKYDPERKYIDDLLAEVDKYNV
>DpleCSP8
MAVCYCRPDTYTDRYDSIDLDEILRNRRLLVPYIKCILEEGRCSADGKELKSHIKEALENDCAKCTKAQQDGTKKVI
GHLINKENDYWQQLVEKYDPEHKYVEKYEKELRA
>DpleCSP9
MATLYIFIFATISTLVLAEFYSSRYDDFDIQPLLENDRILFSYTKCFLDQGPCTPEAKDFKKAIPEALETTCGKCSPKQK
QLIRKVIKAIIEKQPQAWEQLVEKYDTEKKYRESFNKFIEEEN
>DpleCSP10
MKVVIILATLITLVLTQDTYSSDLENFDIDELLENDRLLESYGKCLEYKGPCTSQGREFRKLMPEAIRTNCLKCTLKQ
REMVRKAAKALNVKLPKIWNELVKQEDPKGEYKQKFEDFLQRSD
>DpleCSP12
MILTVTCVLSYDEKYDRLDVDAILADDQHFTSYLDCFLDKAPCTTEYSKEFRELLPEVIKTACEKCSDTQKVKVRKF
VKAIFEKKAEMAQEFRTKFDPTGEYEPAFLAFLKQNK
>DpleCSP13
MRCLLLALCALTVVASGVAQQNQFLHRYDNFNPDSIIQNERILLAYYKCVMDKGPCTKDGKNFKRVLPETLSTAC
GRCSPNQKIVVRKMLLGIRAKSEPRFFELLDKYDPTRVNREALYSFLITGDERNF
>DpleCSP11b
MKVIIVLSALLVLSSAETYNSENDDLDIEKLVSDPASLGAFLDCFNDKGACDELSGDFKKDLREAVEQACEKCTAA
QKHIFKRFLEVIKVQKADDYKIFQQKYDPENKYLPALEAAIAKY
>DpleCSP11
MSDMQLERTLADRVMMQRHIKCALSEGPCDPTGMRLRTLAPLVLRGSCPQCSSQETRQIRRTLAFVQRNYP
WEWTKILSHVIVIFAMAALCFAETQRPAVSDTALEDALNDKRFIQRQLKCALGEAPCDPIGKRLKTLAPLVLRGAC
PQCSPQETKQIQRTLSYVQRNFPQQWAKIVRQYSG
>DpleCSP12a
MKTIIFLAVIAFAVADPELYDVVTLDMDKMEKDPQEFNSLIDCLLDRGPCTPVFQTYRGIVQEATENICKKCNPY
QKRAYWHFLRILRTTNPVDYENFRQKFDADNVYVDILESVLRGFANLK
>DpleCSP13a
MGMRHFPKNCYKMMIIITLAALLINAVVAEEEKYDLPLLDMKEIAGNPNQLKSFLDCLLDRAPCKDVYQGYRDL
APESIREACRKCTTELKIFAYIFFYTLKTFLPEEYQNFRNKYDPDNIYIDRLENEVSKYRLAAIAS
>DpleCSP14
MKSIFLVFLLAFVLNCAIADEQYYVLQKVNLSESSDIIGVMKNLMNCFLERSPCSEAFESYRVRIPEAFQQACKKC

SPEQKRFAAEFIQSLKAEMPEDYNDFIKKYDPENKYFDALEAELNKFI
>DpleCSP15
MKIVYFIFFIVFGVFADNKYYDSRYDYYDIDHFVQNPRLLKKYIDCFLDKGPCTPIGRVFKLVLPEIVITACNKCSPS
QKRFAHRTFEAFRNISPQNYAELRRKLDPQNKHFNTFLKSIATNS
>DpleCSP7b
MLKGATWQSAYLMVVPSPMDSLIGEEEERRKRWENGGKSKRGKGKGQPALTLIGRSEAIKNYFTPIFCERVYKK
ERGLKHRHTRLYQAHYIKGIKSKKKMKLLFLLVLVSFAAAEEQFYSLQKIDLSKAEENIGEFKKFTDCLLEKGPCSDV
YESYRVRVNESLQSACGKCTPELKQFAAKFFEILKNYLPQEYDGFLKKYDPENKYYDVFMAEVLKYKV
>DpleCSP15a
MKIVILLSLLSVVAANDHFYYNLIPLEVTALAKNPKQIFEFLDCLLDKGPCNDVFEGYRAVSLEAVQQACKRCTAD
QKRFVQRRLQMNRYKMKIFITLAALVINAVVAEEELYDLPLLDMREIARNPDQLKSFLDCLLDRAPCNPLYQGYR
DLAPESIRESCRKCTPALKIFAYVFFTTLKTFLPEEYQNFRNKYDPDNIYIDRLEEEVRKYAFGTFSG
>DpleCSP16
MLDKFKGGGPKAIFDIVTAHSAKRTVEYLAMAGLVIESNPPYCTELAPCDFYLFQELKIKIEVFILGALKMRSTINM
KIVILLSLLSVVAANDHFYYNLIPLEVTALAKNPKQIFEFLDCLLDKGPCNDVFEGYRAVSLEAVQQACKRCTADQK
RFGNIFLMLLRKLLPQEYHNFRYKYDPKNKYFDALEAELSKYKYLPCL
>DpleCSP7c
MADSPCLPPWILLIEKGGGHERAADALLSFGLVGSMSGLLLSALCRIRRRLVSLKSIRVIFNGSPFLFLRQIFNMRT
LILALVAFAAANSQYYVLEKLDMDLLVKNLDELKIFTNCLLEKIPCNKVHESYRVKTLEAVQEACKKCNPHTKHMF
WEYLQALKADLPKEYIEFRHKYDPDNKYFDIHEAEISKYAIE
>DpleCSP17
MVIIIILAALVINAVVAEQEKYNSSLFDLEEVVNLAPESIRESFRKWTPALKIFAYVFFSTLKTFLPEEYQNFRNKYDP
DNIYIDRLEEEVRKYAFGTFSG
>SinfCSP1
MKLIIAVALLCMVAVSWGQRASTYTDKWDNINVDEILESERLLKAYVDCLLDRGRCTPDGKALKETLPDALENE
CSKCTAKQKTGSDKVIRHLVNKRPELWRQLSIKYDPDHIYQDKYKTQIESVKQ
>SinfCSP2
MKADYVFLATLMAVVAADFYDSKYDSFDVQPLLENDRILLGYSKCFLDQGPCTPDAKDFKKVIPEALETTCGKCS
PKQKQLIKSVIKAVIDRHPDTWEQLSEKYDKDKKFKESFDKFLAEED
>SinfCSP3
MMKFAILLCVMVAAVMAEEKYTDKYDNIDLDEILSNKRLLMAYVNCVLDKGKCSPEGKELKDHLSDAIETGCKK
CTEAQEKGTYKVIEHLIKNELDIWRELCDKFDPTGTWRKKYEDRAKANGIVIPE
>SinfCSP4
MKAVICLCALVVVAIARPEDQYTSQYDNTNIPEVLGNQRLLHSYIKCCLDLGKCTSDAKELKSHIQEALETHCAKC
TKKQEQSINIVLKHLINHEPEYWKQLCDKYDPEGKYAKIYEKELKNAS
>SinfCSP5
MKSYIVLCVLSVAVMALARPEEAQYTDRYDNVDLDEVLGNRRLLVPYIKCVLDEGKCAPDAKELKEHIREALENK
CAKCNEAQKKGTRRVIGHLINHEADYWNQLTAKYDPERKFTAKYEKELKEITV
>SinfCSP6
MMYSALATMLLIYLTSQSNAMETSTYTTKYDGIDLDEILNNERLLNGYVNCLMDLGPCTADGKELKKNIPDAIE
NDCKKCTDRQRDGSDRVMHYLIDHRPDDWVKLEKKYNSDGSYKMKYLSSKPTEGNSVTNVTKSEEDTKNDSK
E
>SinfCSP7
MKAVICLCALVVVAVARPEEQYTSKYDGTNIPEVLANQRLLHSYIKCGLDQGKCTTEGKELKSHLKEALETHCAKC

TDKQKEATRLVIKHLINNEADYWKQLSDKYDPEGKYAKKYEDELKTVS
>SinfCSP8
MNSFLILCLFGLVMAAVARPNTTYTDKYDSVNLDEILGNRRLLTPYIKCMLDQGKCTPDGKELKSHIREALEEDC
AKCTKTQRDGTRRVLGHLINHEKDYWNQLKAKYDPQSKYSSKHEEELRTLKQ
>SinfCSP9
MKAVICLCALVVVAVARPEEQYTSKYDGTNIPEVLANQRLLHSYIKCGLDQGKCTTEGRELKSHLKEALETHCAKC
TDKQKEATRLVIKHLINNEADYWKQLSDKYDPEGKYAKKYEDELKTVF
>SinfCSP10
MQFKYAILLCCVAAVSVAQTQRPAVSDTALEDALQDKRFIQRQLKCALGEAPCDPIGKRLKTLAPLVLRGACPQC
TPQETKQIQRTLSYVQRNFPQQWAKIVRQYAG
>SinfCSP11
MKLVCIVLLLTVVAVQAQYSTENDDLNIDALVANEESFKSFVGCFLDRVTCDAVPADFKKDIPDAVKTSCTKCTDA
QKHIFHKFLLGLKEKLPSDYEAFKNKYDPGSEYFTALEAAVANS
>SinfCSP12
MKVLVVLSALIAFTAAAALSPSELDMLEAFDYDTLFANDEHRNIVFNCMLEKGDCGDFKQVAELSREIVKNKCAE
CSPKQKAKYDTVMKKLHDNFEPFYNDLMKKIEAKQE
>GmolCSP
MRLLIAIALACIIGLAYGRPQTKYTSKWDNINVDEILESQRLLKAYTDCLMDRGRCTPDAKALKETLPDALENECA
KCTEKQKQGSDKIIRNLVNKHPDIWKELSGKFDPDNKYTEKYRDQLEKVKQ
>CmedCSP16
MKILAVTLVLTTCVTINTADESTYTTKYDGVDINEVLGNERLLTSYVNCLLDLGPCTPDGKELKNNLPDAIQNDCK
KCSDRQREGADQVMDYIIDHRAEDWAKLEKKYNSDGSYKKKYLERKQAKEAKEAKESKEHDESQEKTE
>CmedCSP7
MMKFATALVLAVCVGLAVGETYSTENDDFDIEALVKNPEEFQKFSGCFLDKNECDAVSGDFKKDIPEAFEQACA
KCTDAQKHLFNRFLSALKDKRPQDFEDFKKKYDPEAKYYAALEKAVAKA
>CmedCSP
MRPIVAMFLLAIIGLAAAAPAEKTDDSDEVNVDEILANRRLLLPYIKCALEQGKCSSSGKKVKDHIKKSLQNDCET
CTEKQKKDTNAVFKHLINKENDYWNQLIAKYDPQRQYAPKHEKLYLGKA
>CmedCSP6
MKAIILFGLTTLVAVAWGRPGTLYTDKWDHINVDEILESQRLLRGYVDCLLDKGRCTPDGKALKETLPDALEHNC
SKCTEKQKSSSDKVIRHLINKQPDYWKELSTKYDPQNIYQERYKDKIDDVKAKA
>CmedCSP20
MKFLVVLSMVFAVAVADTYKTTHDSIDVEAVVTNPDSLKAFTGCFLDTGACNEVAASFKKVLPEATEQACAKCT
PAQKHMLRRYLEEVKKTSPEDFAALNKKYDPEGKYVEALRAAIANA
>CmedCSP18
MRIIILLSCLVVVFAADKYSTKYDNFDVDTLISNDRLLKAYINCFLEKGRCTPEGADFRKALPEAVETTCAKCTEKQ
KNNIRKVIKAIQQKHPKQWEELKKKTDPSGKFRADFDKFIQGS
>CmedCSP21
MKTFTAICLLALAASAAAFPGSDKYTDRYDNINLDEIISNKRLLVPYIKCMLEQGKCSPEGKELRSHIKEALENYCG
KCTETQRDGTRRVIGHLINNEPDYWSQLTVKYDPDHKYVTKYEKELKTVVA
>CmedCSP12
MQKILCIVFLSSVVSTIAYPAPQMTDGQLEQTLADRSTMQRHLRCALQEGPCDPVGRRLRILAPLVLRGACPQC
SVQETRQIQRTLAYVQRNYPWEWAKIVRQYG
>CmedCSP15

MKLVIFACLVALAAVAHAGPQRGYTDVYDSIDINEVLGNRRLLLPYLHCVLGEGKCTPPGKELKSHIKEALETQCA
KCTPAQRTGTRKVIAHLINHEAEYWNKLTAKYDPTGQFTKKYENELRVIA
>CmedCSP22
MKVVLVLFAVIAVALSAKTTYTSKFDNIDVDNILNNKRLLEGYVKCLMGTGKCTSEGNELKEKLPEALATECEKCT
PAQKEKSEKIIRFLVNNRRELWDQLAVKYDPKDEYRQKYLDQAKAKGIDV
>CmedCSP13
MKVLILTLCLAAAALAQEKYDSVDDNFDISEVLDNERLLNSYAKCLLDKGPCTPEVKKVKDKLPEALQTRCAKCTD
KQKQIGKKLAQEVKKKRPDLWKDLVAHYDPQGKYQESFQDYLKP
>CmedCSP17
MRSTILLTILAIVALSLAEDTDQAKDTKSKESGKESDKESGNESGKESGKEETYTDRFDNINVDEIVANRRLLVPYL
KCALDKGRCTPEGKELKIHIQDAMQTACKKCTEKQKTGARQVVNHIKEKEPLYWEELLAKYDPKNELKPIYEPFL
AGKDK
>CmedCSP10
MRSWLVLVLAVVVSCSAQHYNRYDNFNADSIIQNDRVLLAYYKCVMDKGPCTKDGKNFKRVLPETLSTACGRC
SAMQKLVVRKLLLGIRSKSEPRFLELLDKYDPERSNREALYNFLVTGN
>CmedCSP4
MISTKYLLVLCCVAAALARPNDKYTDKYDNLNLQEILENKRLLKAYVDCVMGRGKCSPEGKELKEHLQEAIETGC
EKCTEAQEKGAYTAIEYLIKNELDIWRELAAHFDASGKWRKKYEDRARANGIIIPE
>CmedCSP14
MQIQVLLLVVAAAACAYAAETAHPTVSDTALDDALNDKRFIQRQLKCALGEGPCDPIGKRLKTLAPLVLRGACPQ
CSSQETKQIQRTLSYVQRNYPQQWAKIVRQYAG
>CmedCSP19
MLYLTVLLVLVGQCYAKLHNYENFDLESFLSNEAKAKAFLGCVSDDSKCGSQGDLEMKNDIVEMMMTSCAGCT
EKEKEKYKEGLAVLQKSVGDPQTAMFNQFANLFLWGTDPEGKNQ
>CmedCSP8
MKTQIIFLCVLGVIALVSCAPHQSYANQPETTPEEIMRVESQLPCVLNQGPCSELGLRIKMVLPEILTTRKCSTCTP
EENAKVGRILYIMHEKFRHHLITLNNIYGKKQQSSNFHHSN
>CmedCSP2
MKTFVALFALVAVAVARPEDTYNTAYDNFNAKELVENARLLKNYGKCFLDQGPCTPEGADFKKTIPEALKTDCAK
CTPKQRELIREVVSGFQSKLPEVWAELVKKHDPEGAYKESFEAFLHAKN
>CmedCSP1
MKSFVFFFLSALMVAAMAADYNDLKDVNLEELLSDEARRKQIFDCVMDLGPCGEYQIYKDVVPGVIATQCGDC
TPELKKKYEENSKFLLEKYPKEFTAVVEKYGPKKEE
>CmedCSP3
MPPAYFCTLVMVTMVTADFYNPKYDDFDIQPLLENDRILVGYTKCFLDQGPCTPEAKDFKKVIPEALETSCGKCS
PKQRQLIKMVIKAMMERHPDSWEVLVDKYDKDKKYRDNFNKFIESDDK
>DhouCSP12
MKIFLLLCLLAVGYSHATETYTTENDDLDIDRLVNNNDEFLSFIRCFLDMQECNSVAADFKKDLPEAVQTACLKCT
QAQKHIFNKFLAGLKEKLPKEYEEFSNKFDPQGIYFGPLEK
>DhouCSP10
MKTFDIIMLSIVSLVIADFYSPRYDDFDIQPLLENDRILLGYVKCFLDEGPCTPEAKDFKKVIPEALQTTCGKCTLKQ
KELIRTVIRAIIQDHPESWEQLIDKFDKEKMYRDAFNKFLADKNE
>DhouCSP6
MKGIIFLSCLLAVVVSGKYTDKYDNFDYKEVVENEKLFQAYLKCFLDIGPCTPEGKEVRSHLEEALQNGCEECTEK

QKEGIEYCIRYMIEKKPEQWKSLCDKFDPTGKYKKQYEKEAEQRGIKIP
>DhouCSP2
MRVLVVLAYLVAATYAAEKYNSKYDNFDVETLIQNERLLKAYINCFLDKGRCTAEGADFKKALPEAVETTCAKCTD
KQKTNIRKVIRAIQEKHPKYWDELVSKNDPTGKYRVNFDKFIQGS
>DhouCSP14
MNSIIILSCLLAVVACEQYTDKYDNTNLEEIMENDNLRQAYLKCLLEEGPCTAEGKKLKELLTEALETECSKCTDKQ
KQGAKYCIKFLIKNSPKEWEKLSKKYDPNGKYQAKYEKLAKEEDVELPK
>DhouCSP7
MKTVIVCLCVLVAVTVARPGDHYTDKYDKINIPEILNNRRLLIPYILCILDKGKCTAEGKELKSHIREAIVEDCAKCT
NTQRSATETVIAHLVNYEPEYWNELKGKFDPSGIYTKNHEADLRKLKAH
>DhouCSP11
MKVFLITLCLAFAAMAEDKYESVNDDFDINKVLKNDRLLNSYVKCLLDKGPCTPEVKQVKDKLPEALATHCAKCT
DKQKQMGKQLAQEVKNNRPELWQELVHKYDPKGKHQEAFKDFFES
>DhouCSP3
MQTSRIVFACVVLAVCAAEQTPRPEVSDTALDDALSDKRFIQRQLKCALGEGPCDPIGKRLKTLAPLVLRGACPQ
CSPQETKQIQKTLSYVQRNYPQQWAKIVRQYAG
>DhouCSP9
MKWYILSAILALTTLVAAEQYTDRYDSMNVDEILANRKLLIPYIKCVLGQGRCTPEGKELKAHIKDAMQSACEKC
TDKQKVYAKKVVKHIKAQEAQYWQDLKKYYDPKNEYQHTYEGFLASED
>DhouCSP5
MRGVFLLCLCAYVVGQNVDDMVDMPKYDQRYDYLDVDAVFTNKRLVRNYVNCLINAQRCSPEGKQLKRILPE
ALRTKCVRCTDRQKKTAVKMIRRLKYEYPEEWMKLSSRWDPTGDFTRYFEEFLAKENSNIIPGSGSELPSSVAPA
PSEEVPSTPEQDKSAEMPVVVPLSIPSMPISPNRFSDGELMLGSSSSASGSSRPTTVKPVTIKQVMPTRPTMWT
STDINTMPTRFTLGSSTELQPLSTAITFIDQIGYKIIRTTELVTDILKNTVRAVVG
>DhouCSP4
MKTLIILFALAAIAYARPEEYYSTQYDDFDVDELVNNIRLLKNYGKCFLSQGPCTPEGNDFKTVIPDAVKTKCVKCS
PKQKVLIRKVVRAFQDELPEIWSELVQQEDPKGLYKTEFENFLNGTD
>DhouCSP1
MKLLLVVTVISFCSFAINGEEKYDKKYDNINIDEILDNKRLLVAYIKCILEKGKCTAEGYELKSHITDALQTGCKKCTK
QQRDGIRKVISHMINHEPEYWGQMVDKYDAERIYTHRYEKELKTIH
>DhouCSP16
MQRPIFLCLSAAILIALIPSMLGAPQMSDAQLEQALADSATIERHIKCALGEGPCDTVGRRLRTLAPLVLRGACPQ
CTVQETRQIRRTLAFVQRNYPWEWAKIVRQYG
>DhouCSP13
MNFIIITSFVIFQIFIINGEETSYTNEFDGLDLHEILTNNRLLTAYVNCLLENGPCTPDGKELKKNLPDAIDNGCKKCT
QKQREGADEVMHYIIDNRPEDWTKLEDKYHSDGTYKRKYLASKQVMGEPQDNNSTQNAAT
>DhouCSP17
MKSFIILSCVVVLAFAQKYSGKLDSVDIKELFENDSMKTNIFNCFTEKGTCAPEFQEIKDLLTNPSTIDDNLSEAQL
EKFKDAAKYMHEKYRSVYDELAAKNDPTGQWRKKYGLP
>DhouCSP8
MKGIIAVCVLSLAALVLSDCPDGLYTDRYDNTNVEEIMTNDRLLMPYVSCMLDKGKCTPEGKELKNHIAHALQN
GCECCTDDQKTKSRVVIGHFINEKPDVWDELTAKYDPKKLFSKKYEEELRKLKA
>DkikCSP7
MKTLIILFALAAIAFARPEEYYSTQYDDFDVDELVNNVRLLKNYGKCFLNQGPCTPEGNDFKKVIPDAVKTKCVKC

SPKQKVLIRKVVRGFQEELPEIWSELVQQEDPKGLYKTEFENFLNGTD
>DkikCSP12
MKSIIILSCLLAVVACEQYTDKYDNTNLEEIMENDNLRQAYLKCLLEEGPCTAEGKKLKELLTEALETECSKCTDKQ
KQGAKYCIKYLIKNSPKEWEKLSKKYDPNGKYQAKYEKLAKEEDVELPK
>DkikCSP1
MRVLVVLACLVAATFAAEKYNSKYDNFDVETLIQNERLLKAYINCFLDKGRCTAEGADFKKALPEAVETTCAKCTD
KQKTNIRKVIRAIQQKHPKYWDELVAKNDPSGKYRVNFDKFIQGS
>DkikCSP11
MRGLYLLCLCAYAVGQNMDDIVNMPKYDQRYDYLDVDAVFTNKRLVRNYINCLINAQRCSPEGKQLKRILPEAL
RTKCVRCTDRQKKTAVKMIRRLKYEYPEEWMKLSSRWDPTGDFTRYFEEFLAKENSNIIPGSGSEFPSSVAPAAS
EEQPSTAEQDQAAEMPIVVPLSIPSKPISPNRFNDGELVLGSSSSASGSSRPATVKPATVNQVVPTRPTMWTNT
DINTMPTRFTLGSSTELQPLSTAITFIDQIGYKIIRTTELVTDILKNTVRAVVG
>DkikCSP2
MKVFLITLCLAFAAMAEDKYESVNDDFDINEVLKNDRLLNSYIKCLLDKGPCTPEVKQVKDKLPEALATHCAKCT
DKQKQMGKQLAQEVKNNRPELWQELVHKYDPNGQHQKAFKDFFES
>DkikCSP17
MQRPIFLCLSAAILIALIPSLMGAPQMSDAQLEQALADSATMERHIKCALGEGPCDAVGRRLRTLAPLVLRGACP
QCTVQETRQIRRTLAFVQRNYPWEWAKIVRQYG
>DkikCSP9
MKTFCIIILSIVSLVIADFYSPRYDDFDIQPLLENDRILLGYVKCFLDEGPCTPEAKDFKKVIPEALQTTCGKCTSKQK
ELIRTVIRAIIQDHPESWEQLIDKFDKEKKYKDAFDKFLADKNE
>DkikCSP15
MQTSRIVFACVVLAVCAAEQTQRPEVSDTALDDALSDKRFIQRQLKCALGEGPCDPIGKRLKTLAPLVLRGACPQ
CSPQETKQIQKTLSYVQRNYPQQWAKIVRQYAG
>DkikCSP16
MKFVVVMVVIAVAAARPGNPYEKFDNLDVDEVLGNSKLTTSLLKCMVDDGRCTPEGTDLKNLAPGAVQNKCA
DCTAEQKTKAAKFMRAAKKDYTELFNKLVKIHDPNNEHTAELDEFLTQN
>DkikCSP13
MNFIIITSFVIFQIFIINGEETPYTNEFDGLDLHEILTNDRLLTAYVNCLLENGPCTPDGKELKKNLPDAIDNGCKKCT
QKQREGADEVMHYIIDNRPEDWTKLEDKYHSDGSYKRKYLASTQVMGEPQDNNSTQNTAI
>DkikCSP6
MKGIIAVCVLSLAAVVLSDCPDGLYTDRYDTTNVDEIVSNDRLMTPYVSCLLEKGNCTPEGKELKNHIAHALQTG
CECCTNQQKSKSRVVIGHFINEKPDVWNELAAKYDPKKVYTKKYEEELRKLKS
>DkikCSP4
MKGIIFLGCLLAVVVSGKYTEKYDNVDYKEIMQNPKLFHPYFKCVLDQGSCTAEGKELKAHISEALENGCEQCTE
KQKEGIDYCIRYLIENYPEQWKSLCDKFDPTGKYKKQYEEEAQKRGIKIP
>DkikCSP3
MKLVLCIAFTYLVVAVWCKPSSTYTDKWDHINVEEILESQRLLKAYVHCLLGKGLCTPDGKALKETLPDALEHECS
KCTAKQKSSSDTVIRFLVNKRPDLWKELSHKYDPDNIYQERYKDKIDSVKGSA
>DkikCSP10
MKILLLCLLTVGYAHATETYTTENDDLDIDRLVNNDDQFLSFIRCFLDKQECNSVAADFKKDLPEAVQTACLKCTQ
AQKHIFNKFLAGLKEKLPKEYEEFTNKFDPQGIYFGPLEKAVASS
>DkikCSP5
MKSVIVCLCVLVAVTIARPGDHYTDKYDNINIPEILNNRRLLIPYILCILDKGKCTAEGKELKSHIREAIVEDCAKCTD

TQRSGTETVIAHLVNYEPEYWNELKGKFDPSGIYTKNHEADLKKLKAH
>DkikCSP8
MKWYILSAILALTTLVAAEQYTDRYDSINVDEILANRKLLIPYMKCVLDQGRCTPEGKELKAHIKDAMQTACEKCT
AKQKVYAKKVVKHIKAQEAQYWQDLKKYYDPKNEYQQTYEGFLASED
>PrapCSP
MAGKLIVIFCVLVAVFAEKYTDKYDNINLQEILDNKRLLHAYVDCLLDKGKCSPEGKELRDHIQDALETGCSKCTE
AQEKGTYTIIEHLINKEKEIWEKLCAKYDAEGKYRKKYEERAKSAGLAV
>PaegCSP
MQSIVILMTLLSMALAIPSQTYDPEYDNFNAEDLVENVRLLRSYGKCFLEKGPCTAEGSDIKKIIPEALRTSCAKCT
PKQQQLVRTVARAFQSKLPDLWKELVLKEDPKGEFREAFTQFLDNSDITALKMKH
>SnonCSP1
MMKFVLLLCVMVAAVMAEEKYTDKYDNIDLDEILSNKRLLMAYVNCVLDKGKCSPEGKELKDHLSDAIETGCTK
CTDAQQKGTYRVIEHLIKNELDIWRELCAKYDPTGTWRKKYEDRAKANGIVIPE
>SnonCSP2
MKVVLLALCLALGVLAQDKYESANDDFNIKEVLENDRLLTAYSKCLLGTGPCTPEVKQVKDKLPEALETRCAKCT
DKQKEMGKTLAQEVKKNHPDIWKQLVAKYDPQGKYQQAWKDFLQE
>SnonCSP3
MRVLIVLACLIVMAFAADKYNSKYDNFDVETLISNDRLLKAYINCFLDKGRCTPEGSDFKKTLPEAIETTCAKCTEK
QKTNIRKVIKAIQQKHPKEWDDLVKKNDPSGKNXANFDKFIQGSS
>SnonCSP4
MYSALATMLLIYLTIQSNATETSTYTTKYDGIDLDEILNNERLLNGYVNCLMDLGPCTADGKELKKNIPDAIENDC
KKCTDRQRDGSDRVMHYLIDHRPDDWVKLEKKYNSDGSYKLKYLSSKTTEDNSVTNVSKSEEDTKNDSKE
>SnonCSP5
MNSFIILCLFGLVVAAVARPDTTYTDKYDSVNLDEILGNRRLLTPYLKCMLDQGKCTPDGKELKSHIREALEEDCA
KCTKTQRDGTRRVLGHLINHEKDYWNQLKAKYDPQSXYSSKXEEELRTLKQ
>SnonCSP6
MNFLVLSIVVTLAAFAATETYTDRYDHINIDEIIENRKLLVPYIKCTLEQGRCTPEGKELKAHIKDAMQTACSKCTEK
QKKGARKVVKHIKAKEQDYWKQIIAKYDPENQYSETYEAFLAEKE
>SnonCSP7
MQFKYAILLCCVAVVSVAQTQRPAVSDTALEDALQDKRFIQRQLKCALGEAPCDPIGKRLKTLAPLVLRGACPQC
TPQETKQIQRTLSYVQRNFPQTVGQNSPPIRGIIYKKVSCIGNMIG
>SnonCSP8
MKSYIVLCVLSVAVMALARPEDAQYTDRYDNVNLDEVLSNRRLLVPYVKCVLDEGKCAPDAKELKEHIREALEN
KCAKCNEAQKKGTRRVIGHLINHEADYWNQLTAKYDPERKFTAKYEKELKEITA
>SnonCSP9
MKAVICLCALVVVAVARPEEQYTSKYDGTNIPEVLANERLLHSYIKCGLDLGKCTTEGKELKSHLKEALETHCAKCT
DKQKEATRLVIKHLINNEADYWKQLSDKYDPEGKYAXKYEDELKTVS
>SnonCSP10
MPKYDQRYDYLDVDAIFTNKRLVRNYVDCLINSVRCTPEGKALKRILPEALRTKCIRCTERQKRAAVKVIRRLKYE
YPDEWSKLASRWDPTGDFTRYFEEFLAKEHFNTIPGSGSAIPTSAPLPPRNPTMPPTIASPAPGPTVPTPPKPAIL
NRFGDDGELMQGSSSSVGMTPRPMTQATTRPTTTMKPANTKPIPPRPTMTTWAGAASNTQPTRFPLRPSSD
LPPPYFHSHNTH
>SnonCSP11
MKLLIVLALVVLAVASPDGDHYDSKYDSFNVDELIQSPRLLKSYAHCFIGDGKCTPEGKEFKGWIPEATTSSCGKC

TEKQKVLVAKFIKAIKEKFPEEYGALVKKSDPEGTHGGDLQKFIDKYGS
>SnonCSP12
MKFVCIVLLLAVIAVQAQYSTENDNLDIDALVANEESFKSYLGCFLDRVTCDAVPAEFKKDIPDAVKTTCSKCTDA
QKQIFHKFLLGLKEKLPSDYEAFKNKYDPGNEYFTALEAAVANS
>SnonCSP13
MKADCVFLATLSVMVAADFYDSKYDSFDVQPLLENDRILLGYSKCFLDQGPCTPDAKDFKKVIPEALETTCGKCS
PKQKQLIKSVIKAVIDRHPDTWEQLSEKYDKDRKFKESFDKFLAEED
>SnonCSP14
MKLIIAVALLCMVAMSWGQRTSTYTDKWDNINVDEILESERLMKAYVDCLLDRGRCTPDGKALKETLPDALEN
ECSKCTAKQKTGSDKVIRHLVNKRPELWRELSTKYDPDHIYQDKYKTQIESVKQ
>SnonCSP15
MINLNKCSFRGSLILTYLFLVTVLAQDKFYDRRYDYYEIDTLIQNRRLLRKYLECFLWKGPCTPIGRVFRQILPEAVS
TACKKCTXSQRRLARKAFNAFRKYFPEXYVELMKRLDPKNKYYEAFEKAIXNA
>SnonCSP17
MKNWLLCLCVLTVVVSCYSQGANRYENFNADAIIQNDRILLAYYKCVMDKGPCTRDGKNFKRVLPETLSTACGR
CNPTQKAIVRTLLLGIKSKSEPRFMELLDKYNPDRSNRDALYNFLVTGN
>SnonCSP18
MNALLIAVFALVAPSALAYDEIYDKIDVDKILGDDALFTTYINCMLDKGECTVEHSADFKKLLPEVIATSCGKCSPI
QRQNVRKTVKALSEKKPEEFTQFRAKYDPKGEHEKSFSAFVMGTD
>SnonCSP19
MKTILALCLLVVAVCAKPEATYDSRYDNFDVESLVGNTRLLKSYGHCFLGTGHCTAEGSAFKKQIPDALRTGCSKC
SPKQRHLIRVVVKGFQTKTPDIWNQLVKKEDPTGEYKESFTKFLNSSD
>MsexCSP1
MKCICLLFLVVVAVYAEEKYTEENDDLDIEGVIKDADTMKAFTGCFMDTADCDHVSGDFKKDLPEAIQTACAKC
TDKQKHITKRYFEGLEEKYPELYQAFKNKYDPENKYFAALKAAIAKF
>MsexCSP4
MQVTYVLLVCVVVASCVAQQAQRPQVTDTALEDALNDKRFIQRQLKCALGEAPCDPIGKRLKTLAPLVLRGACP
QCSPQETKQIQRTLSYVQRNYPQQWAKIVRQYAG
>MsexCSP13
MRTVIVLTFLVAACFAAEKYNPKYDNFDVDTLISNERLLKAYINCFLDKGRCTPEGTDFKKALPEAVETTCAKCTEK
QKVNIRKVIKAIQQKYPKQWEELVKKNDPSGKHRANFDKFIQGS

Supplementary Table 6.

Gene	Forward primers (5'-3')	Reverse primers (5'-3')
PBP1	AAACTGAGCTTAACGTGGGC	TCGTGGATCTTAGTGCGGAA
PBP2	GCGTTCTGTCCATCGGTTAC	TTCATGGCAAATTCCTGGGC
PBP3	CGCCTCAGGATTCGTGAAAG	TTAGCTCGTGGATACCGGTC
GOBP1	ACTGACGGAGGAGAAGATGG	AGCCTCCATGATGAACTCGG
GOBP2	GTGCAGAGAAGAGTCAGGGT	ATCATTGCGACTTCTGGTGC
OBP1	GCTTACATCAGTACCACGCC	ACGCAGCATCAAACATCGTT
OBP2	ACAGAAGAACAAGTTGGGAGAA	TCATACAAACATTTCGATGCGT
OBP3	AGAGATGGACGAGGACATGG	TAGTCCGCCTTGTTAGCGTT
OBP4	CTGTTTGCCATCGTCCTCAC	TTTCCATTGCGTTCTCCAGC
OBP5	GTTCCTCGTTATTGGTGGCG	AACTTCATAGCCGGCTCACT
OBP6	TGGTTTTGTGTGTTGTGGCT	TTTTCGTTGACTGAAGCGCA
OBP7	TCTGGAAGTGATGCCATGTCT	CTGGCTTAAACTCGTACATGCA
OBP8	AGTTGCAACGTGTGAAGCTA	ACATGAAATGACGCATCGCA
OBP9	ATGTGCCTCGTCAAGTACCATG	CGACCTCGGTCCCAGTCATT
OBP10	GATTCCGAGTCAAGATGCCG	GGTACACACACTGCAGCAAA
OBP11	GTGTTCGTGGTTGGGGTTTG	TGTCTCCACGGCATCTTTTC
OBP12	ATGAAGACTCTGTTTGTTTTCGCT	TTACAAAAAAATCGCATGCTTG
OBP13	GATAACATCATGTTTATTAGTACTAAGTG	TAACGAACTTCAAAATCTTTTATTG
OBP14	GCCTTGTTTGTAGTAGCTGTGA	GGGGCAAGTAAACTCGCATC
OBP15	TGGGACAGAGCGTTAAAGGT	GGGAACATGAAATTAGCTGGGT
OBP16	ACCGCTTTGTGATCCTCAGT	TTCCGTCGTCACTCCATTCA
OBP17	CCTTTCGACTACTCTGTTGCA	AGCATCCCTCATCTCAGCTG
OBP18	GTGGAGCAATAGACAAGGCG	GTAGCAGGCGTTACAAGAGC
OBP19	ATGACCGAGTGCTTGAAGGA	CATGAGGTCGTTCGTGCAAT
OBP20	ATAACACTCTCCGGCTTCGCT	ATGTGTGCAGTCGTTGAAGACCT
OBP21	TGCTGTTACTCATGGCGGTA	GGCATCGGTCCTTATCTGGA
OBP22	ATGTCGAAGTTTACCTGCATTATCC	TTAGAACGGCATCTCAACTTTGTG
OBP23	ATGGCCAAGTTCTCTTGTCTTGTT	TTAGATGATGATACTAGTCTTATGCTCCA
OBP24	TGTGTTTATAAAAAGACAGAATTTTTG	CACCTCTCTCGCATCCAGCC
OBP25	TCAAGCAGATGATGGCAAGA	TTAATAATCAACACCAAACATATTTGG
OBP26	GGCCTGCATGATGAAGCAAA	GCACCATCACTGACCGATTC
OBP27	ATGTATAAATTTGTTATTTTGTGCTCTA	CTAGCACTTCCACGCTGTGTC
OBP28	ATGATCGTTCGGTTTTTATTATGTTT	TTATGGTAATTTTATTCCCAGCTGAG
OBP29	GCTCAAGATGGAGTTCACAAAGTTA	GGGTGCATTTTCAACTGTGC
OBP30	TCTAAAGCAGAAGTATGTGGACA	CTGGTCGTTCATCATTCCCG
OBP31	CGAACACCGCGAGAAAGAAA	AGCTCTATTAGCTTCTAATAAATGTTC
OBP32	CGAATTGATAGGAATGATATACCTTGTA	CTATTTCCATTCATCGGGATCTCT
OBP33	CTGGGTAAGGAGGAGAAGGC	CGCCTTCTTGTCATAGCCAC
β-actin	CGAGAAATCGTGCGTGACAT	ATCTGCTGGAAGGTCGAGAG

Supplementary Table 7.

Gene	Forward primers (5'-3')	Reverse primers (5'-3')
PBP1	AAGATGACCAAGGGATTC	CGCCAGTAGTTATACATG
PBP2	CGAAACCTTTGGATGATT	TCCAGAAGTTGTAGAAGT
PBP3	TACATCGCCTCAGGATTC	TGGAACAGGTCTGCTATG
GOBP1	GCCAGGAGAGTCAACTGA	CGGTGCTCGAACTTGAAG
GOBP2	ATGTCACCGCCCATTTCG	GGAACTCCTCTAACACCTCTG
OBP1	CAACTGATGACGACTATA	TGCTGGTATTATTCTTGT
OBP2	CTCAGTAACAGAAGAACAAGT	GCAAGCGACATAACACAT
OBP3	GCTCAAGTGCTACATCAAGT	TACAGCCTCGATGTCCAC
OBP4	AGTTCAATCCAGCGAGAG	ATGTCAGCGATGTCTTCC
OBP5	CGGTGTTCCTCGTTATTG	ATACCTGCCTCTTCATCTC
OBP6	ATGGCATCAACTCCTGTT	TATCGGCGTCGTATTCTC
OBP7	CTATGAATTGACGAATGTGATAA	CGCAGATGTCCTTGTATT
OBP8	TTGCGGATGCCCATAAAG	TACCAGGATTGTGATTGTAGATG
OBP9	CAATCCGAACAACCACAA	GTAACGCACACGACTTAT
OBP10	GATTACTCTGTGTCAGGATG	ATCGTGTGCTCTTCTTTG
OBP11	AATAGAGATGTAGCCATT	GTTGATAGCATTTGAAGA
OBP12	CGCACTGAATGTCTAACT	GCCTCATTGTCCATCTTAT
OBP13	ATTCCTACACTGTATCCA	ATTACGCAATATCCTTCTT
OBP14	GAGCCTTAGTAGTGTTCA	AATTGTTCTTCAGAGATGTC
OBP15	GATATGAAGGCACCAGTTA	TCGCTGTCCAATAAGATG
OBP16	CTTAACAACTACGGACCA	TCACTCCATTCAGAACAG
OBP17	AAGTGGTGAAGAATAACAA	ATCGTAGTCGTACATACA
OBP18	AATAGTAGTCCTGGAACC	GGCACCTGTATATGTTATC
OBP19	ACTGGTCCTATGCGAATT	GAACTTCCTTCAAGCACTC
OBP20	AATCCAGGAATCAGATGAAC	TCTAGTTGGTGAGCGATT
OBP21	GAGGAGGATATAACGAAT	AATCCACTGTATCATCAT
OBP22	AAACTGGACTTGGTAAACTAA	CGTCGCTTACAGATTCAT
OBP23	AACTGGATTGAAGAACCT	ATCACTCACTGCCTTATC
OBP24	TAGCAATGATGATGATTAC	GTTAGTAGTACACCTCTC
OBP25	TGTAGTAGGTGTGAGTCT	AGTCGTCTATGATCTGATTC
OBP26	AATTGGTGTTATGGATGAC	GATTGCCTTCAGTTCTTC
OBP27	GACTGATTCCATTTACAAA	TTCTTCAACATAGCGATA
OBP28	ACTCAATAGCAATGACACAA	CTTCGTCGTTCACTTTCT
OBP29	TGTGTATGCTCGAAGAAG	ACCTCTCCATCCTTGTTA
OBP30	GTGGACAACATATTAGAATG	TATAAACACAGGCAAACA
OBP31	TTGTACTGTTCTCATTCG	GTTCTCTGTCATCTTGTT
OBP32	CCTGTGCTCAGAACATTA	GTAGGACACTGCGTATAG
OBP33	CTGGGTAAGGAGGAGAAGGC	CGCCTTCTTGTCATAGCCAC
β-actin	CATCCGTAAGGACTTGTA	TGATCTTGATCTTCATTGTG
RL-31	AAGTTGTAACTCGTGAATAC	GTTCCCATTTGTTTCTCAG

Supplementary Table 8.

>BmorGOBP1
MWKLVVVLTVNLLQGALTDVYVMKDVTLGFGQALEQCREESQLTEEKMEEFFHFWNDDFKFEHRELGCAIQC
MSRHFNLLTDSSRMHHENTDKFIKSFPNGEILSQKMIDMIHTCEKKFDSEPDHCWRILRVAECFKDACNKSGLA
PSMELILAEFIMESEADK
>BmorGOBP2
MFSFLILVFVASVADSVIGTAEVMSHVTAHFGKTLEECREESGLSVDILDEFKHFWSDDFDVVHRELGCAIICMS
NKFSLMDDDVRMHHVNMDEYIKGFPNGQVLAEKMVKLIHNCEKQFDTETDDCTRVVKVAACFKKDSRKEGI
APEVAMIEAVIEKY
>BmorPBP1
MSIQGQIALALMVYMAVGSVDASQEVMKNLSLNFGKALDECKKEMTLTDAINEDFYNFWKEGYEIKNRETGC
AIMCLSTKLNMLDPEGNLHHGNAMEFAKKHGADETMAQQLIDIVHGCEKSTPANDDKCIWTLGVATCFKAEI
HKLNWAPSMDVAVGEILAEV
>BmorPBP2
MKLQVVLVVLTVEMVCGSRDVMTNLSIQFAKPLEACKKEMGLTETVLKDFYNFWIEDYEFTDRNTGCAILCMS
KKLELMDGDYNLHHGKAHEFARKHGADETMAKQLVDLIHGCSQSVATMPDECERTLKVAKCFIAEIHKLKWAP
DVELLMAEVLNEVSWKS
>BmorPBP3
MARYNIVVAVLVLGVVGARGSSEAMRHIATGFIRVLDECKQELGLTDHILTDMYHFWKLDYSMMTRETGCAIIC
MSKKLDLIDGDGKLHHGNAQAYALKHGAATEVAAKLVEVIHGCEKLHESIDDQCSRVLEVAKCFRTGVHELHW
APKLDVIVGEVMTEI
>BmorOBP5
MKQRLRVLLLRFCILQTVLSESGVDVVKNLSLSFARFFLECDEERHFQPEVRLKVMTFWYSESSTWDRDVGCAF
LCIFKKMEIDNPQDPSYRTHLELLSFANSEDNKIANQMVEIFYACGENTETDPCLWALEQVKCYKNRINQLGLTP
TF
>BmorOBP7
AVTEEELKIEFTKLVMKCTKDHPVDMSELMQLQQLIAPKKTESKCLLACAYKLNGVMTSQGLYNLEHAYKIAEM
SKNGDEKRLENGKKVADICVKVNDVEVSDGEKGCERAALIFKCTLENAPKVFKFGSSEYNCQ
>BmorOBP8
MLRVVVICVCFLVIAPYGINASSLDDLKMVYKNVIKECVGDYPITAADLKLIKARQIPNDDIKCVFACAYKKTGM
MTEEGMLSVEGIKDMSQKYLSDNPEQLRKSKEFAEACSSVNDQQVSDGTKGCERAALIFKCSTEKITNFGFEL
>BmorOBP9
MLRVVVICVCFLVVAPYGINAVSYEQKIKIRDQLDRAGFECFKDHKITEDDIKNLRANKPATGENVPCFIACVMKK
TGVMNDQGVIRKGPVLELAKKVLADDKDIKKLQDYIHSCSHVNSETVHDKGKGCEFAMQAYTCMSANASKFG
FNI
>BmorOBP10
MLRVVVICVCFLVIAPYGINAVSDEQKIKIREQIDKSGFECFKDHKITEDDIKNLRARKPATGENVPCFIACVMKKT
GVMNDQGVIHTEPVLQLAKKVLTDDKDIKKLQDYIHSCSHVNSKTVHDKGQGCEFAIQTYTCMSANASKFGFD
V
>BmorOBP11
MSANSFVVLAFCALAVGVNALTEEQKAEITKSSLPLIAECSKEFSVNQGDIDAAKKLGDPSGLNSCFVGCFMKKA
GIINASGLFDVAATIEKSKKYLTSEEDLKAFEKLTETCAPENDKPVSDSDKGCERAKLLLDCFVANKGSFSVFSL
>BmorOBP12

MTSFMVFFVLSVLTLKYSDALTDEQKNKIQSKFIEIGAECIVEHPISIDDINSFKNKKFPSGVNAGCFVACIFNKIGL
FDDKGNLSHNSALEKAKGIFNADEEVKNLEEFLNRCAKVNGEAVGDGVKGCERAKLAYNCLIENSLEFGFNIDF
>BmorOBP13
MLKIHVLLCFGMAILYFGSAKAVTPEESKAFEAFAKPVIEQCQKDFGMDKESFAQKNLDEIDECLIACVVEKFGIT
NDEKIDGDALKALVTKFVGNEEERNKINKIVEECTEDANKSGDGTCNTSTILFLCLLKNGKDLWGF
>BmorOBP14
MSRQQLKNSGKMLKKQCMGKNDVTEEEIGDIEKGKFIEQKNVMCYIACIYQMTQIIKNNKISYEASIKQIDLMY
PPELKESAKASAGRCKDVSKKYKDICEASYWTAKCMYEDNPKDFIFA
>BmorOBP15
MFLKNIFIECVLLYFVMLNTSFVNTMTKQQIKNSGKILKKACISKNDVTEDQISDIDKGKFIEDKNVMCYIACVYS
MSQVVKNNKFVHDAMVKQVDMMFPTEMRDAVKASIANCRGVAKNYKDICEASFWTAKCMYEFDPANFVF
A
>BmorOBP16
MRISFLFLISVTIITFDSVFAMTRAQVKKTMTIMKNQCMPKNGVTEDQVGKIEEGIFLENHNVMCYIACVYKTI
QVVKNDRLDKDLISKQIDVLYPQEIRESTKKAVGDCINLQEKYDDWCEGIFRSTKCLYEKDPANFIFP
>BmorOBP17
MTRQQLKNSGKIMKKTCMPKNDVTEEEIGQIEQGKFLEQRNVMCYIACIYTVTQVVKNNKLSYDAVIKQVDV
MFPAEMRPAVKAAAENCKDISKTFKDICEASYWTAKCMYDFDPKNFVFP
>BmorOBP18
MILIVIAKFLILISLCETMTMKQIKNTGKMMRKSCQPKNNVDDEKINPINDGVFIEENEVKCYIACIMKMANTM
KNGKLNFEAAMKQADLLLPDEMKEPTKEAIVACRKVADSYKDVCDASFHVTKCIYNHNPSVFFFP
>BmorOBP19
MTSAKTDVEIKAWFLGQAVECSKDHPVTTEELRMHKHELPDSKNAKCLMKCVFRKCNWLDSKGMYDINAAY
ASSTKDFSDDKTKQENANKLFDTCKSVNEENVGDGEEGCDRSLLLAKCLTKAAPQVSIYYS
>BmorOBP20
MAVHIFLILASYMALAAHGQLDDEIAELAAMVRENCADESSVDLNLVEKVNAGTDLATITDGKLKCYIKCTMET
AGMMSDGVVDVEAVLSLLPDSLKTKNEASLKKCDTQKGSDDCDTAYLTQICWQAANKADYFLI
>BmorOBP21
MITASLHVIFALLAFVYGGKDKPVLSEEIKEIIQTVHDECVGKTGVSEEDITNCESGIFKEDVKLKCYMFCLLEEAGL
VNDDGTVDYEMFTSLIPEEYFDRATKMIFSCKELDTPDKDKCERAFEVHKCSYEKDPDFYFLF
>BmorOBP22
MLKVFVVVVCTLGASQLCAALYTQKVAVSFPKDKTTIVVEAMKSCIAKTGANPNVIEVISSGKVSEDEKFKEFFYC
ACNDIGVVNPDGHIKVKECIELFPKETQPLVEPVIKNCDKEGVNKYDTLFKYLKCFQETSPVRVTLA
>BmorOBP23
MTSKVLLSCVVLAVLATTVLAEDSRKLVSFAPEVAKKLKVLIQECLNENGLGEDAIEVIRAGEYREDEPFQNLVYCA
YKKFGALDENNRIISQVAAASFPKDIDVVTVIESCGKEDGNTPVEQVFKYFKCFQKNSPVRMQLY
>BmorOBP25
MKSVVLICLAFAVFNCGADNVHLNEDEREKANWYTAECGVETGVSTEVINAAKIGKYSKDKAFKKFVLCFFKKS
AILNSDGTLNMVVALAKLPSGVNKSEAQSVLEQCKNKTGQDAADKAFAILQCFHKGTKTHILF
>BmorOBP26
MKSVVLICLAFAVFNCGADNVHLAETQKEKAKQYTSECVRESGVSTEAINAAKIGKYSKDKAFKNFVLCFFNKSA
IFNSDGTLNMDVALAKLPPGVNKSEAQSVLKQCKNKTGQGAADKAFEIFRCYYKGTKTHILF
>BmorOBP27
MKSVVLICLAFAVFNCGADNVHLTETQKEKAKQYTSECVKESGVSTEVINAAKTGQYSEDKAFKKFVLCFFNKSA

ILNSDGTLNMDVALAKLPPGVNKSEAQSVLEQCKDKTGQDAADKAFEIFQCYYKGTKTHILF
>BmorOBP28
MLKVFIVTFFAFQLSAIARLQANGCVAVPFPKDKTIIIVEAMKSCIAKTGANPNFIDVIRSGKVSEDEKFKEFYYCT
CNDTGFVNPDGHIKVKECIELFPKETQPLVEPVIKNCDKEEGVNKYDTLFKFLKCFQETSPVRVALA
>BmorOBP29
MTGPAAAAVLLALLAAAGQATTGCKNCVILGKEERAMFRSHSDACLAQSRVEPRLLESMMNGELIDDAALRK
HVYCVLLSCKMIGKDGKLLKAAILGKLAARPAGRDVTKVLEACAEQPGASPEDVAWNIFRCGYNRKAVLFDYM
PAGGASSGNTENHP
>BmorOBP30
MRSFVILLNYGLLCCGQFMAEDYYYDIVTRDPDDLMREKENEVRALRAFQADCAEDVQVKPDLVVNLKSGDW
QTEDVSLKKWALCVLMKLGLMTAQGVFKMNEAMSKIPDMNDKIIAEKLIDDCLSLQATTPHDAAWNYIKCHH
QKDPEGNFSSLNIF
>BmorOBP31
MKTFIVFVVCVVLAQALTDEQKENLKKHRADCLSETKADEQLVNKLKTGDFKTENEPLKKYALCMLIKSQLMTK
DGKFKKDVALAKVPNAEDKLKVEKLIDACLANKGNSPHQTAWNYVKCYHEKDPKHALFL
>BmorOBP32
MYSHKYLNDFTNIPEILIILLSSVALMSYGYNTKLFSHSLGSEPSLSILYARDKKSDKVTNECLMEMYPKNLYKYPLR
IDRNDIPCIIHCVLKKFGIISNDGFINIKNYYRRVQAIHRYDPRILISDVGETCAQNINGMNLDHDVCKKAKVFND
CTQLYAISYREPEDW
>BmorOBP33
MYAHDKLSDMIADQCLNEMYPRSKRLEIEESDEPCIIFCVLKKFGIMSPTGVINLEAYRKRVQLPEQLAQRNSIN
DFGSACLESAEATQHKQDVCKKAKVFNECTHLYKILLK
>BmorOBP34
MEKMILLNVFAVVLPCVLASRTRGSSGTLVDFTDPKVQGHLDALVRMAQSCVIKVRATPKDVRAYFTNSSPVSR
SGQCFATCMLEQSDIINHGKVNRDLLVHLAGLVNGKNSRVVRKLNSVSRLCLDSISGMTDRCQLASTYNDCLNE
NMIEFAFPLDIAEEAVRKMPFHLIQPK
>BmorOBP35
GMSTHVLDFKRNMTECLKEVQNNDKRPIKRLSPKQESPIHGECLIACVLKKNGVIQNGKVNKDNLMALVSKFH
AKETKLMKKLEKNLDRCINISVKNHDECSLASQLNDCTNDIMASSKQKILFNY
>BmorOBP36
MAVSEISRILTFLTIVSFIYIVYSFKPLTKDEHIERYNKMNEDIEPFRKNLTECARQVKASMADVEKFLKRIPQSNME
GKCFVACILKRNSLIKNNKLSQENLLEVNRAVYGDDSEVMSRLKTAILECSKIVEDIFEICEYASVFNDCMHMKM
EHILDKITMERRMEALGQMSSNPDEWSEEEDEMLKLVKDEL
>BmorOBP37
MFYPFRFTLLFYGLFVIYLVRAEPEKENHFTLALKKTLFSTARSCMSHVNANETDLEYLRKDPPFPDKAACIIKCLLE
KIGVVKNNKYSKMGFLTAVSPLVFTNKKKLDHYKSVSENCEKEINHDQTTECELGNEVVSCIFKYAPELHFKT
>BmorOBP38
MANLVLLLTFVLMTLSMARLKSTEAPKSKTALFNDQDNMGYEELDMEEIMSACNESFRIEYAYLESLNDSGSFP
DETDKTPKCYIRCVLEKTEILSENGVLNPATAALVFAGERNGKPMSDLEEMAVACADRHEKCKCEKAYNFVKCL
MYMEIDKYEKKN
>BmorOBP39
MVRKISALLCCFCVLGISMCDSAISTDNEQRCKNPPTAPQKIERVITLCQDEIKLSILREALDVIKEEHTMPAERKR
NKREVPFTHDEKRIAGCLLQCVYRKVKAVDGFGFPTLEGLVGLYSDGVNERGYFMAVLEASRECLMKNHDKFS
RTTPMDNGRNCDVSFDIFECISDRIGEYCGTSGL

>BmorOBP40
MSEFIQPSWRTQCNFRLNWDNRNRLSIDISHGAATTQTPVPTTKPKALRDFMVVPQSCDKTTCVFKKLNIVSD
KGVVDVKSFIKLLDKFTNSYPVWNSAKARVITTCLRKSLIAYDGGCELNNILACTFDVLSENCPLNGNNQTC
>BmorOBP41
MLTILFLLPIVVGVLSGNIPEQPRVYCGELPNTIYSCLGNPKIIQPEVSEKCNKPISECDKTRCIFKESGWAKNNVID
KKKVSDYFEQFAKDNPDWSAAVQNFKTTCLSDSLKPQGVDTNCPAYDIIHCALISFIKFASPSQWSTSEQCVYPR
QYAGACPVCPERCFAPSVPNGSCNACLALLRTP
>BmorOBP42
MMGYACVFVILAVLQAISAEDPPGLPPFLKDAPEKCKSPPRVKNPNECCISEPFFKEADFIECGIEKPGSERGPPD
CSKQNCLLKKYNLLKNDETPDIEAIKSLLDKYIEKNPSFKSSVEKAKECLREDLPGPPQICLANRMTLCIGTVLLME
CPDEKWNTTDDCKAFKDHMTECQKYFPK
>BmorOBP43
MKVCVLFAIFTVAQAAKATLKPISACCNIPELGNPEPLAECSNPKLPGPCKDIQCVFEKSGFLTENKTLIKEAYKTH
LRQWAKEHEGWSVAVEKAISDCVDKDLRQYLEFPCSAYDVFTCTGIAMLKKCPNEHWTC
>BmorOBP44
MSRLVLFFTILVVLQEFIINLYFNFITEIDSCCVKKYPKLFDSEFITECYNTQRKANDKCERDMCVARKLNLLTEEDSI
NKDALLRFVEEGFKTEIDLVNAIKKKCFEEDISNIGKPEMCEVAKYKICITSRMAEDCPKWDSKGICSSAQQKVEN
FMKMLS
>SlitPBP1
MANARWRFVFVVYALYLTSAVLGSQDLMVKMTKGFTRVVDDCKTELNVGDHIMQDMYNYWREDYQLINRD
MGCMLLCMAKKLDLMDDQTMHHGKTEDFAKSHGADDDVAKKLVSVIHECEQQHAGIADDCMRVLEVAKCF
RTKIHELKWAPSIEVIMEEVMTAV
>SlitPBP2
MAFCPSVTMSLRVALVVAASLLVVVQASQDVMKNLAVNFAKPLDDCKKEMDLPDSVTTDFYNFWKEGYELTN
RQTGCAILCLSSKLEILDQELNLHHGRAQEFAMKHGADEAMAKQIVDMIHTCAQSTPDEAADPCMKALNVAK
CFKLKVHELNWAPSVELIVGEVLAEV
>SlitPBP3
MGSRNVFVALVVLTVGMREIEPSKDPMKYIASGFVKVLEECKHELNMNDHLIADLFHYWKLEYTLLNRDTGCAI
ICMGKKLDLLDASGRMHHGNAQEFAKKHGAGDEVASQIVQIIHDCEKKHERDDDECLRVLEVAKCFRTGIHEL
NWQPNVEVIVSEVLTEI
>SlitGOBP1
MLLLLRALPLLAAVLPLRADVNVMKDVTLGFGQALDKCRQESQLTEEKMEEFFHFWREDFKFEHRELGCAIQC
MSRHFNLLTDTSRMHHENTEQFIQSFPNGEVLARQMVELIHACEKQHDHEEDHCWRILHVAECFKQACVQR
GIAPSMEIMITEFIMEAEAR
>SlitGOBP2
MTSKCCLLLVLMAAATSSVMGTAEVMSHVTAHFGKALEECREESGLSAEVLEEFQHFWREDFEVVHRELGCAII
CMSNKFSLLQDDSRMHHVNMHDYVKSFPNGHVLSEKLVGLIHNCEKQFDSMTDDCERVVKVAACFKVDAKA
AGIAPEVAMIEAVMEKY
>SlitOBP1
MKEGNRYSHERRITNDSGDQLMVINATDDDYSGYGSGNMGEKLLTSVPRPATPSNNINKNNTSRTKRNEPLLN
RPDSDQCLSQCVFANLQVVDSRGIPREAELWNKVQSSVTSQQSRSALHDQIQACFQELQSEAEDNGCSYSNKL
ERCLMLRFSDRKVDGKGNAKKSSTEQTG
>SlitOBP2
MTRAQVKKTMGIIKNQCMPKNSVTEEQVGRIEQGVFIEDRNVMCYVACIYKSLQVVKNDKLDMALITKQIDILY

PPELKEPVKKSVAACFHSQDNYSDFCEGVFYASKCLYEK
>SlitOBP3
MWMQALVLTLATLATLAAAAVEMDEDMAELARMVRDNCAGETGVDVALVEKVNAGAELMPDDKLKCYIKC
TMETAGMMADGEVDIEAVLALLPPSLAEHNAPALRACGTQRGADHCDTAFRTQQCWQNANKADYFLI
>SlitOBP4
MTKVLFAIVLTMITFAVVLSASTKEAMTTTMSDQVNSIDVDVLAVMDMCNDSYRIDPTYLQALNESGSFIDETD
KTPKCFIRCVFENVGIVSEDGKQFNPARAAVIFAGERNGKPMEDIADMTALCATDRQETCPCDRSYKFLRCLMS
MEIERYEKS
>SlitOBP5
MSVVRCSSLLVAIFCFVSVNAISGDEEAGIKDALRPFVQECADEFGITEEQFEEAKKKASAADIDPCFMSCFLKKA
EFFDSQGKFDVDSTMAFAKEHLTSEPAMKFVEAVGDECVKINDEDVSDGDKGCDRAKLLFECIAETKKKME
>SlitOBP6
MSKFTCLVLCVVAVSLSGVHATAEEKAAFIEAVKPYVQECSKEHGVTPEDIKSAKAAGNADGINSCFLSCVYKKA
EVITEKGEYDADKALEKLKKFVSNEDDYAKFANIGKKCASVNEKSVSDGEAGCERAALLTSCFLEHKSEISA
>SlitOBP7
MDQKRICLFVIAMFLASGSDAMSRQQLKNSGKMLKKNCMNKIGVTEDQIGSIDKGKFIEDRKVMCYIACIYELT
NVIKNNKLNYEASIKQIDLMYPPDVKESAKAAVEKCKDVQKKYKDICEASFYAAKCMYEFKPEDFIFA
>SlitOBP8
MLLTKIVKFFILVATCEAMTMKQIKNTGKMMRKTCQPKNNAEDEKIDPISDGVFIDEKEVKCYMACIMKMANT
IKNGKLNYDAAMKQADLLFPDDIKEPAKEAITACRKVADAHKDICDASFHVTKCIYNHNPGIFYFP
>SlittoPBP1
MANARWRFVFVVYALYLTSAVLGSQDLMAKMTKGFTRVVDDCKTELNVGDHIMQDMYNYWREDYQLINRD
MGCMLLCMAKKLDLMDDQTMHHGKTEDFAKSHGADDDVAKKLVSVIHECEQQHTGIADDCMRVLEVAKCF
RTKIHELKWAPSMEVIMEEVMTAV
>SlittoPBP2
MSLRVALVVAASLLVVVQASQDVMKNLAINFAKPLDDCKKEMDLPDSVTTDFYNFWKEGYELTNRQTGCAILC
LSSKLEILDQELNLHHGRAQEFAMKHGADEAMAKQIVDMIHTCAQSTPDVAADPCMKTLNVAKCFKLKVHEL
NWAPSVELIVGEVLAEV
>SlittoPBP3
MGSRNVFVALVVLTVAMRETEPSKDPMKYIASGFVKVLEECKHELNMNDHLIADLFHYWKLEYTLLNRDTGCA
IICMGKKLDLLDANGRMHHGNAQEFAKKHGAGDEVASQIVQIIHECEKKHERDDDECLRVLEVAKCFRTGIHEL
NWQPNVEVIVSEVLTEI
>SlittoGOBP1
MLLLLALPLLAAVLPLRADVNVMKDVTLGFGQALDKCRQESQLTEEKMEEFFHFWRDDFKFEHRELGCAIQC
MSRHFNLLTDSSRMHHENTEQFIQSFPNGEVLARQMVELIHACEKQHDHEDDHCWRILHVAECFKQACVQR
GIAPSMEMMITEFIMEAEAR
>SlittoGOBP2
MATVTSSVMGTAEVMSHVTAHFGKALEECREESGLSAEVLEEFQHFWRDDFEVVHRELGCAIICMSNKFSLLQ
DDSRMHHVNMHDYVKSFPNGHVLSEKLVGLIHNCEKQFDSMTDDCERVVKVAACFKVDAKAAGIAPEVAMI
EAVMEKY
>SlittoOBP1
MFKLCVFLALGFVACHGASNSNPGTPNANPGTYCGVTPDNIYRCLNNPRVVTPEVSTKCGSQFTECEKMTCIF
RELKWSKRGAIDKAKVRAYFDQYETEHPEWAQAVQHVKAFCLASELRAQGVFLNCPAYDIMQCVLASFIKHAS
PSVWSTATDCAYPKAYAADCPVCPSDCYSPQIPYGSCNACYTQPRTV*

>SlittoOBP2
MVRKISGLLCCLCVFGISFSDSAISADSESRCRNPPTAPQKIERVITLCQDEIKLSILREALDVIKEEHTMPAQRRRD
KREVPFTHDEKRIAGCLLQCVYRKVKAVDGYGFPTLEGLVGLYSDGVNERGYFMAVLEASRECLMKNHDKFSRT
VPMDNGRNCDISFDIFECISDRIGEYCGTSGL
>SlittoOBP3
MKSFVVICIVFVVGVCATEKGNKIASECIKESGVKSDVLAEAKKGNLGDDPAFKEFTYCFFKKVGIVGEDGKLNRD
VAIAKLPSGVDKAEAEKLLDSCKSKTGKDAVETVYEIFKCYQHGTKSHIMFAS
>SlittoOBP4
MKTLLVFAACILVAQALTDEQKEKLKKHRTECLTETKVDEQLVNKLKGGDYKMDNEALKKYALCMMMKSELMT
KDGKFKKDVALAKVPNPADKPTVEKLIDACLANKGNTPHQTAWNYVKCYHEKDPKHAIFL*
>SlittoOBP5
MTKVLFAIVLTMVTFAVVLSASTKEAMTTTMSDQVNSIDVDVLAVMDMCNDSYRIDPTYLQALNESGSFIDET
DKTPKCFIRCVFENVGIVSEDGKQFNPARAAVIFAGERNGKPMEDIADMTALCATDRQETCPCDRSYKFLRCLM
SMEIERYEKS*
>SlittoOBP6
ESKFGEIVKRTVIATAHTCMDHVNATAKDLEHLRDEPPYPETSACIVKCLLEKVKYMRKQTN
>SlittoOBP7
MFTEALPLFVILVAVTHGGKNKPVFSDEIKEIIQTVHDECVAKTGVAEEDITNCENGIFKEDAKLKCYMFCLLEEAS
LVDDDDTVDYDMLVSLIPDEYYERTTKMIFACKHLDTPDKDRCQRAFEVHKCSYEKDPDLYFLF
>SlittoOBP8
MFGSKIVFSLFMIVSVCYGAVDIKKYFKVCDRNAIDVNDCMAEAVRQGIATMINGIDELGVPPIDPYLQKDFRLE
YKNNQLAAKLTLKNIYVEGLKEAIVHDARLRADDDKFHLEVDLSGPRVSVRSDYYGEGQFNALKIVAYGQVNTT
MTDLVYTWKLAGVPEKNGTETYIRIKDFYMRPDVGSLVTHFRNDNPESRELTDLGTRFANENWRMLYKEFLPY
AQANWNRIGVRVANKLFLKVPYDQLFPTSS
>SlittoOBP9
MCLVKYHVLVLCVILVGSYALNCRSSGGPKEAELKNIYKKCLKMQEGKNSSKGNSAQDWKEPRVQIQSNDNDG
SGNRGRGNKNNRNDMNGGRDDRFGRDDYFNGREDFPQSDEYGGDMGQYNNNYYSTTQSSRRYKRERRPS
NSGQRSQYNPNNHKISGYEDNFRSDERNTTDNNSSKETDNKSCALHCFLENLEMTGEDGMPDRYLVTHAITK
DVKNEDLRDFLQESIEECFQILDNENTEDKCEFSKKLIDLSVRE
>SlittoOBP10
MKEGNRYSHERRITNDSGDQLMVINATDDDYSGYGSGNMGEKLLTSVPRPATPSNNINKNNINRTKRNEPLLN
RPDSDQCLSQCVFANLQVVDSKGIPREAELWNKVQSSVTSQQSRSALHDQIQACFQELQSEAEDNGCSYSNKL
ERCLMLRFSDRKVDGKGNAKKSSTEQTG
>SlittoOBP11
MSKFTCLVLCVVAVSLNGVHATAEEKAAFIEAVKPYVQECSKEHGVTPEDIKSAKAAGNADGINSCFLSCVYKKA
EVINEKGEYDVDKALEKLKKFVSNEDDYAKFANIGKKCASVNEKSVSDGEAGCERAALLTSCFLEHKSEISA
>SlittoOBP12
MSVVRCSSFLVALFCFVSVNAMSGDEEAGIKEALRPFVQECADEFGITEEQFEEAKKKASAADIDPCFMSCFLKK
AEFFDSQGKFDVDSTMAFAKEHLTSEPAMKFVEAVGDECVKINDEDVSDGDKGCDRAKLLFECIAETKKKME
>SlittoOBP13
MITSSSLLVLTAVVQVLFAQQPVFESGPPEPWGPPQRPAHRRQFLPRIPKRCWVPPQRINVYNCCPIPTLYPDED
MQSCGFEKTSGNTDQPQKPVFRPEGTCKEGYCVMGKFDLLFANNSVDFVKFREYLDNWAESYPEFANAIRIAK
QECAQDGGPEVPPICEPDKLFLCLTSTIFWNCKLRDGDGCAALQEHMNECKQYYTRQMEPTMKDIEVR*
>SlittoOBP14

MDQKRICLFVIAMFLASGSDAMSRQQLKNSGKMLKKNCMNKIGVTDDQVGSIDKGKFIEDRKVMCYIACIYEL
TNVIKNNKLNYEASIKQIDLMYPPDVKESAKAAVEKCKDVQKKYKDICEASFYAAKCMYEYKPEDFIFA
>SlittoOBP15
MFNNCFVYSMTREQIKNSGKLIKKTCSAKNDLTEDEVKDVDKGKFIEKKDFMCYIACVYKMGQTVKGSTINHD
MMLRQVDMMFPNDMKAPVKAAIEHCRPVAKNYKDLCEASYWTAKCIYDFDPANFMFP*
>SlittoOBP16
MLAEELKDCFDGSGPKDPMKCEIDLCIAKKKGFATDDGKLDIKKFEEVITKDVGSDKDLLDEIKTNCINGDLNNY
GPPEFCDFIKIKHCVTLHMMNHCSEWSDDGNCKVVKELVGKCAKVI*
>SlittoOBP17
MRTFRLLCCILSIFFIFDQSYGMTRQQLKNSGKLMKKSCMPKNDVTEDEVGDIEKGKFIETRNVMCYIACVYTM
SQVVKNNKLSYEAVIKQVDVMFPAEMRDAVKAAATHCKETTKKYKDLCESSYWTAKCMYDYDAQNFVFP*
>SlittoOBP18
MILXYTQKLTNMLLTKIVKFFILVATCEAMTMKQIKNTGKMMRKTCQPKNNVEDEKIDPLSDGVFIDEKEVKCY
MACIMKMANTIKNGKLNYDAAMKQADLLFPDDIKEPAKEAITACRKVADAHKDICDASFHVTKCIYNHNPSIFY
FP*
>SlittoOBP19
MYSKICILLFISYTCLVTADSVSFIKKCKWDDGKCAKESGQNVIQKFAAGISEYNVGVSDPLHIEYVDASSPNMKLI
VTDVVVTGLRNCEVKKIQRFEDSSKLIVKLLCAAELNGKYDMKGQLFVIPIEGNGGLYSKVPKIQINAEVDLNTKQ
GKDGKDHWIVKSWRHTFELKDKSTVKFENLFPDNEFLRTSTNELIAQNGNDVIIEIGANLIKAIVGKIVENIKKFFI
AVPIEDLSL
>SlittoOBP20
MWVQALVLTLATLVTLVAAAVEMDEDMAELARMVRDNCAGETGVDVALVEQVNAGAELMPDDKLKCYIKCT
METAGMMADGEVDIEAVLALLPPSLAEHNAPALRACGTQRGADHCDTAFRTQQCWQNANKADYFLI
>SlittoOBP21
IHYLCAARAVHKIKNITNCENGIFKEDAKLKCYMFCLLEEASLVDDDDTVDYDMLVSLIPDEYYERTTKMIFACKH
LDTPDKDRCQRAFEVHKCSYEKDPDLYFLILRREQLASRDDCVAISGIN*
>SlittoOBP22
MNRLLLVYLVVLYAGCSYGMTRAQVKKTMGIIKNQCMPKNSVTEEQVGRIEQGVFIEDRNVMCYVACIYKSLQ
VVKNDKLDMALITKQIDILYPPELKEPVKKSVAACFHSQDNYSDFCEGVFYASKCLYEKDPASFIFP
>SlittoOBP26
MFNSSVFMYCLYFCALTPYLVSAMTAEQKALIHEHFETIGKSCNKDSTMITAEDIANLRAKKIPTGPNAPCFLAC
MMKQIGVMDDNGMVQKETALEMAKAVFDDPEEIKAIEDYLHSCSHINTESVSDGAAGCERAMLAYKCMTEN
ASKFGFDI
>SlittoOBP31
RTEIDKSAYIDMLANFTGSHQAWIRATEKVVSRCLNANNLERRSDCLINDVLACTLDVLTEYCPYKRKSGICKHG
KHDVPCQISSSKSRPKNRREICLLPELVHHDHLYECDLDALYKVERVAVPVRHKKHVFPSLWHNCKQLETQTSCI
MDKMGILNRYKFMDYFKMKDKIRQFTEDKPEWSAMMDIYTSAYINLPMYSDHCTSERKLLNVIDTMLMTCP
VSKRKNTPQCNSLFMEMVKATPADNQTVTKEKAXXNYEALPPRXXVSDR
>SlittoOBP25
MSKLSCIVFCAVAMRLCVFVASEDANSIFHAPIKPIVIDCAKEYGLSEDDIKKNRGLDGLKNLPPCFIRCVLNKLDII
NDKGQYDADSGIATIKGLMSNNEYLEKISGVLKECESVNEKSVSDGDAGCERALLGAMCYLDHKTIVLA
>SlittoOBP24
MYKIISLVFCIAVCLNRVHGNAEDKIAIMTAVKPFVEECAKKHGVTFEALLTAKASGKIDGVEPCFYSCVYKKTEFL
NSKGEYDVDTALAKLKKYISNDDDYAKLSQVGKRCASVNSKPVGDGEAGCERGVLLTQCFLDHKGSVPM

>SlittoOBP28
MIVRFLLCLYIVEFYGVHVQARTDQEIKAWFFREGMDCNIEHPISXKEMLELKENKIPDTNNAKCFVACVFKKTG
MLDSXGMFDAENSIAMTQKDFANDPNRLESSKKLLETCKKVNDETVSDGEKGCERSVLLHKCFVETAPQLGIKL
P
>SlittoOBP29
GLTEEELKMEFTKLIMKCNKDGEVDMTELVQLQNYVVPTKQSTKCVLACAYKAAEVMNAKGEYDIDHAYKVAE
MMKNGDEKRLVNAKKMADLCVKVNELSVSDGEKGCDRAAMIFKCTVENAPKFGFKL
>SlittoOBP30
HKFSAIQIPYNVLSRDDSNYLFVNNFNVVITKNYYETNNSKQALSMDDLKQKYVDNILECSKQYPIDRADAEQL
QNRIMPDKEPIKCLFACVYKLAGMMNDQGELSVEGVNAISRKYLAEDPEKLQKSEEFTEACRSVNDAPVSDGT
RGCDRAALIFKCTIEKSPEFNFV
>SlittoOBP27
QLFNSGVVFCIIVAVFLKNASAITDELKAHIEAKFLTVGAECIKEHPLTIEDLSAFKNRVFPDGENAGCFSACIFNKL
GLFDDKGTLSHLTALENAKKVFEDQGELESIEKFLTTCAKVNDEEVSDGEKGCERAKLAYNCFIPKTIEQLGFDL
>SlittoOBP23
MAKLSCLVLCVVAASLGSIHVAKGESLRESLRPVIVACSKEHGVTDAEIQAAKEAGSPASIKPCFIACVFKKAGFIN
EQGQLDLETGLKNLRQFVKDDEQYKQLEGVANKCSPVKDKAVSDGAAGCDRGVHLAGCFLDHKTSIII
>HarmPBP1
MEFHRSTMMSVRLALVVAVCLFIRVDASQDVIKNLSMNFAKPLEDCKKEMDLPDSVTTDFYNFWKEGYEFTN
RQTGCAILCLSSKLELLDQELKLHHGKAQEFAKKHGADDAMAKQLVDLIHGCAQSTPDVADDPCMKTLNVAKC
FKAKIHELNWAPSMELVVGEVLAEV*
>HarmPBP2
MAASRWLFARAFCLVLMMGSAMSSKELLTKMTGGFTKVVDACKTELSVGDHIMQDMYNFWREEYQLVNRD
LGCMIMCMTAKLDLIGDDQKMHHGKAEEFAKSHGADDALAKQLVGLIHGCETQHQAIEDHCSRALEIAKCFRT
KIHELKWAPSMEVIMEEIMTAA*
>HarmPBP3
MGSRHVFFALVVLAVSVRKAEPSKDAMQYITSGFVKVLEECKHELNLNEQILADLFHFWKLEYSLLGRDTGCAII
CMSKKLDLLDANGRMHHGNAAEFAKKHGAGDEVASKIVTIIHECEKKHEQDGDECLRVLEVAKCFRTGIHELN
WQPKVEVIVSEVLTEI*
>HarmGOBP1
MPGVLRALLVLAAAAPLLADINVMKDVTLGFGQALDKCREESQLTEEKMEEFFHFWRDDFKFEHRELGCAIQC
MSRHFNLLTDSSRMHHDNTEKFIQSFPNGEVLARQMVELIHSCEKQFDHEDDHCWRILHVAECFKGSCVQRG
IAPSMELMMTEFIMEAEAR*
>HarmGOBP2
MTSKSCLLLVAMATLTASVMGTAEVMSHVTAHFGKALEECREESGLSAEVLEEFQHFWREDFEVVHRELGCAII
CMSNKFSLLQDDSRMHHVNMHDYVKSFPNGHVLSEKLVELIHNCEKKYDTMTDDCDRVVKVAACFKVDAKA
AGIAPEVAMIEAVMEKY*
>HarmOBP1
MSKFTFFVLCVVAVSLSKVYASDEDKAKLHEALKPLVEECMKDHEVSLDDLKAAKEAKSADGVKPCFLACVYKK
AEVLNDKGEFDADHALEKLKEFVSDEDVLAKVAEVGNTCKAVNDKAVSDGDAGCERAALLTACFLEHKAEILV*
>HarmOBP2
MMDRKRLCLLIIAMFLAQGSDAMSRQQLKNSGKMLKKNCMNKNQVTEDQIGSIDKGKFVEDKKVMCYIACI
FEMTNVVKNNKLNYDASIKQIDLMYPPDLKESAKAAVEKCKDVQKKYKDICEASYWTAKCMYDFKPEDFIFA*
>HarmOBP3

MSKFTCFVLCVLAVSLGEVRSNALEKAAIRAAVYPLIVDCAKEHAVTLEQLKAAKASHSAEGINPCFQSCVYKKT
GIFNDNGEYDVANAKTKLQKFVTDEDEYARIAEVGKTCASVNDKSVSDGAAGCERAALLTACFLEHRAQIII*
>HarmOBP4
MSKLTCVVFAAVAVVFSNVNADDETRASFRQVLGPLVMECRNEFGITEDDLKKAQQERSPDALKPCFIACVFKK
FGIITSAGKYDSDASISRIKDVVKNDDLLAKLKSVGEKCNSVNDASVSDGDAGCERAALLAKCFIENKSELSI*
>HarmOBP5
MSKFTCLVLCVVAASLSQAYASEEEKAAFREAIKPIVEECSKEHGVSHDELKSAKDNQNADNIKPCFLGCVYKKAE
VFNSKGEYDVDKALEKLKKFVSNDEAYAKFAEVGKKCASVNDKAVSDGDAGCERGALLTACFLEHKAEVPL*
>HarmOBP6
MSKFTCLLLCVVAVSLSKVHATEEEKEAIRAAVRPIMQECGKEHGVTLDDLKAAKAAHSADGIKPCFQSCVYKKA
GIFNDNGEYDIANAKTKLQKFVTNDEEYARIAEVGKMCASVNDKPVTDGAAGCDRAALLTACFLEHRAQIII*
>HarmOBP7
MFRFGVLSFVVLLFCMESSYALSSEEELSIKEALHPFVVECAEEYGMTEEMFEEAKKKGSAEDIDPCFMSCFLKKT
GFFDDSGKFDAEKSISFAKEHITSESAIKFLEAGAGECVKINDEDVSDGENGCDRAKLLFDCLTELKKKMSE*
>HarmOBP7.2
MSRFGVLSFVVLVFCMENIYALSSEEELSIKEALHPFVVECAEEYGMTEEMFEEAKKKGSAEDIDPCFMSCFLKKT
GFFDDAGKFDAEKSISFAKEHITSETAIKFLEAGAGECVKINDEDVSDGDKGCDRAKLLFDCLTDLKKKMSE*
>HarmOBP8
MLLIEIVKFLTLVAMCEAMTMKQIRNTGKMMRKSCQPKNNVADEQIDPIAEGVFNEDKEVKCYMACIMKMA
NTIKNGKLNYEAAIKQADLLLPDDIKEPAKEAITACRKVADAYKDICDASFHITKCIYTQNPGIFYFP*
>HarmOBP9
MCKFSVLFLYSAVMAVNIWSASCISEEDKAAIITAIAPLAQNCGSECGLDNDDFEKYKEDGSDMDPCFKACLMT
QMGVLDKEGKYDGKGLHKAMEEADYPGDKDDAQKFLDELDRCFDAKGDNSGSDEEAKMKRADVLFRCMQ
DMKEK*
>HarmOBP9.2
MCKCSVVFLYLAVMAINIWRASCLSEEDKAAIITAIAPLAQNCGSECGLDNDDFEKYKEDGSDMDPCFKACLMT
QMGVLDKEGKYDGKGLHKAMEEADYPGDKDDAQKFLDELDRCFDAKGDNSGSDEEAKMKRADVLFQCMQ
DMKEN*
>HarmOBP13
MFTGTLPLVVFLATFAYGGKEKPVFSDEIKEIIQTVHDECVAKTGVAEEDITNCENGIFKEDPKLKCYMFCLMEEA
SLVDDDDAVDYDMLVSLIPEEYVDRTTKMIFSCKHLDTPDKDKCQRAFEVHKCSYEKDPDLYFLF*
>HarmOBP18
MKSFVVFCVLVAGAFAANVSLPPKQNEKANQIATECMKESGLKPEVLAEAKKGHISDDEHLKKFTFCFFKKAGIV
SEDGKLNTEVALAKLPPGVDKAEAEKLLETCKGKTGKDVTDTVFEIFKCYHHGTKTHILLGF*
>HarmOBP15
MGSRHVFFALVVLAVSVKKEKPSKHPMPYITSRFVKVLEECQHELKLNEHILEHLFHFWKLEYSLLGKDPGCAIIC
MSTKLDLLDLYGRMHRGNAAEFAKKHAAGDEVPSKIVTIIHFCQKKHEQDGDECLQVLEVATCCRTGLHDLNW
QHQVEVIVPDVLTEI*
>HarmOBP16
MFKLCVVLAFIVATCHGGTLERTSSTCGQIPRELTACLDLQPAVSPEIQEKCRRANECERLTCVFREYNLLDGAEV
NKERTAAFLDNFVKQYPSWEVAIDVAKTSCLRSSGLKPQGVFLDCPAYDIIQCVFANLVKNALPSQWSSMSQCN
HAREFAAACPICPDACFAPLVPIGTCNACSAARRSS*
>HarmOBP17
MRAWSVTLVALLGALGAARAVAMDEDMAELARMVRENCAAETGADVALVERVNAGADLMPDDKLKCYIKC

TMETAGMMADGEVDIEAVLALLPPELAEHNAPSLRACGTVRGADHCDTAFRTQQCWQNANKADYFLI*
>HarmOBP18a
MTRQQLKNSGKLMKKSCMPKNDVTEEEVGDIEKGKFIESRNVMCYVACIYTMTQVVKNNKLSYEAVIKQVDM
MFPAEMRDAVKAAATSCKDITKKSKDLCESAYWTAKCMYDYDAENFVFP*
>HarmOBP19
ARTEHEIKEWLFREGVACNKDFPITPDEMMMLKDNKLPDSTNAKCLIACIFKKTGMIDSKGMFDPDKSIAMTE
KDFADNPEKLATSKKLMEACRGVNEQAVADGEKG
>HarmOBP20
KVFYLLTVLSACYGAVDITKYFKTCNRNAIDVNDCMADAVQKGIAVMINGIDELGIPPIDPYLQKDFRLEYKNNQI
AAKLNMKNIQVEGLRAAKVHDARLRADDDKFHLEVDLTSPKVTVHAEYHGEGKFNSLRILAFGEVNTTMTDLV
YTWKLDGVPEKNGTETYIRIKEFYMRPDVGSIVTNFKNDNPESRELTDLGTRFANENWRTLYREFLPYAQANW
NKIGTKVANKLFLKVPYDQLFPTSS*
>HarmOBP21
FQMSRAQVKKTMSLVKNQCMPKNSVTEDQVGKIEEGVFLEDRNVMCYVACIYKNLQVVKNDKLDMSLITKQI
DALYPPELKEPVKKAVSLCIHSQDNYNDLCEKVFHASKCLYEKDPASFIFP*
>HarmOBP22
MTREQIKNSGKLIKKTCMAKNDLSEDQVKDVDKGKFIEEKPFMCYIACVYKMGQTIKGNTVNHDMMIKQVE
MMFPNEMKAPMKAAIEHCRPVVKKYKDVCEVSYWTAKCIYEFDPPNFMFP*
>SexiPBP1
MAGAKWQFVCVVFALYLTSAALGSQELMMKMTKGFTKVVDDCKAELNAGEHIMQDMYNYWREDYQLINR
DLGCMILCMAKKLDLMEDQKMHHGKTEEFAKSHGADDEVAKKLVSIIHECEQQHAGIADDCMRVLEISKCFRT
KIHELKWAPNMEVIMEEVMTAV*
>SexiPBP2
MAFCRSATMSVRVALVVAASMLVVVQASQDVMKNLAINFAKPLDDCKKEMDLPDSVTTDFYNFWKEGYELT
NRQTGCAILCLSSKLEILDQELNLHHGRAQEFAMKHGADETMAKQIVDMIHTCAQSTPDVAADPCMKTLNVA
KCFKLKIHELNWAPSMELIVGEVLAEV*
>SexiPBP3
MGSHNVFVALVLLAVGMRVAEPSKDAMKYITSGFVKVLEECKQELNMNDHIIADLFHFWKLEYALLSRDTGCVI
ICMSKKLDLLDANGRMHHGNAQEFAKRHGAGDDVASKIVQIIHDCEKKHERDDDECLRVLEVAKCFRTGIHDL
DWQPKVEVIVSEVLTEI*
>SexiGOBP1
MLFLLRALPLLAAVLPLRADVNVMKDVTLGFGQALDKCRQESQLTEEKMEEFFHFWRDDFKFEHRELGCAIQC
MSRHYNQLTDSSRMHHDNTEQFIKSFPNGEVLARQMVELIHSCEKQYDHEDDHCWRILHVADCFKQGCVQR
GIAPSMEMMMTEFIMEAEAR*
>SexiGOBP2
MTAEVMSHVTAHFGKALEECREESGLSAEVLEEFQHFWREDFEVVHRELGCAIICMSNKFSLLQDDTRMHHV
NMHDYVKGFPNGHVLSEKLVELIHNCEKRFDSMTDDCERVVKVAACFKVDAKAAGIAPEVAMIEAVMEKY*
>SexiOBP1
MSKFTCLVLCVVAGCLSGVHATAEEKAALIEAVKPYIQECSKEHGVTPEDIKSAKEAGNADGINACFLRCVYNKA
GVINDKGEYDADKALEKLKKFVSNEDDYAKFAEIGKKCASVTETSVSDGEAGCERAALLTSCFLEHKSEVHA*
>SexiOBP2
MKSFVVFCIVLVVGVCANEKGNKLDRPFASECIKETGVKNELLEEAKKGIISEDPAFKAFTYCFFKKIGIVGEDGLL
NRDVAIAKLPSGVDKSEAEKLLDSCKSKTGKDAVDTVFEIFKCYQQGTKSHIMFAS*
>SexiOBP3

MVKLTCVVFCAVAMALSVFVAGEDANSVFQGAIKPLIAECAKEYKLSDEELLKNRGLAGLSNLPPCFIGCVLKKF
DIINDKGLYDAEAGIAKIEKLLPNNEFLDKISGVLKSCESANEKSVGDGDAGCERAVLVATCYLEHKTAVIA*
>SexiOBP4
MWNFLVVFLAICSCVYGLTEEELKMEFTKLIMKCNKDGKVDMTELVQLQNYVVPTKQTTKCVLACAYKAAEVM
NAKGEYDIDHAYKVAEMMKNGDEKRLVNAKKMADLCVKVNEQSVSDGEKGCDRAAMIFKCTVENAPKFGFK
L*
>SexiOBP5
MTMKQIRNTGKMMRKTCQPKNNVEDEKIDPIAEGVFIDEKEVKCYMACIMKMANTIKNGKLNYDAAIKQAD
LLLPDDIKEPAKEAITACKKVADAHKDICDASFHITKCIYNHNPGIFYSP*
>SexiOBP6
MLGSLLFVFAFSVFSLGAEALLIDDLKQKYADSILQCSQQYPLDRADAELLQNKVMPDKESTKCLFACVYKVTGV
MSDQGELSVEGVNALSQKYLADDPEKLKKSEEFTEACRTVNDAPVSDGARGCDRAALIFKCTIEKSPDFSFV*
>SexiOBP7
MTKVLFAIVLMMITFAVTLSASTKEAMTTTMTDQVNSIEVDVLAVMDMCNDSYRIDPTYLQALNESGSFIDET
DKTPKCFIRCVFENVGIVSEDGMQLNPARAAVIFAGERNGKPMEDIADMTALCATDRQETCPCDRSYKFLRCL
MSMEIERYEKS*
>SexiABP1
MSVVRYSSFVVALFCLVSVNAMSGDEEAGVRDALRPYVQECADEYGITEEQFEEAKKKASADDIDPCFMSCFLK
KAEFFDAQGKFDVDSTMAFAKEHLSSEPAMKFVEAVGDECVKINDEDVSDGDKGCDRAKLLFDCIAETKKKM
D*
>SexiOBP10
MDRKRICLFVIAMFLASGSDAMSRQQLKNSGKMLKKNCMNKIGVTEDQVGSIDKGKFIEDRKVMCYIACIYEL
TNVIKNNKLNYEASIKQIDLMYPPDIKESAKAAVEKCKDVQKKYKDICEVSFYAAKCMYEFKPEDFIFA*
>SexiOBP8
MARRQQGAMFTETLPLFVILVAVTHGGKDKPVFSDEIKEIIQTVHDECVAKTGVAEEDITNCENGIFKEDAKLKC
YMFCLLEEASLVDDDDTVDYDMLVSLIPDEYYERTTKMIFACKHLDTPDKDRCQRAFEVHKCSYEKDPDLYFLF*
>SexiOBP9
MKTLFVFAACILLAQALTDEQKEKLKKHRTECLSETKVDEQLVNKLKGGDYKTESEPLKKYALCMMMKSELMTK
EGKFKKDVALAKVPNPADKPTVEKLIDACLANKGNTPHQTAWNYVKCYHEKDPKHAIFL*
>SexiOBP11
MKEGNRYSHERRITNDSGDQLMVINATDDDYSGYGSGNMGEKLLTSVPRPASSSNNINKNNTRRTRRNEPFL
NRPDSDQCLSQCVFANLQVVDSRGIPREAELWNKVQTSVTSQQSRSALHDQIRACFQELQSEAEDNGCSYFNK
LERCLMLRFSDRKVDGKGNPKKSSTEQT*
>AipsPBP1
MAPHPSVTMYVRLALVIIAGLFITVECSQEIIKNLSLQFAKPLEDCKKEMDLSDTVITDFYNFWKEGYEFTNRQFG
CAILCLSSKLELLDQDLKLHHGKAQEFAKKHGADEAMAKQLVDMIHSCTQSTPDVADDPCMKTLNVAKCFVAK
IHDLKWAPSMDLIMGEVLAEV*
>AipsPBP2
MAASRWCIACLVCVLFAARSVMTSQEVVASFSKGFTNVVEHCKAEVNAGEHIMQDIYNFWREEYQLVNRDLG
CMVLCMANKLGLIGEDQKMHHAKAEEFAKSHGADEAVAKQLVAILYECETKHAAVEDECGMALEIAKCFRTK
MHELKWAPSMEVAMEEIMTAV*
>AipsPBP3
MGTYNVFFAFVLMAAGVREIEPSKDAMKYITSGFVKVLEECKQELNMNDRIIADLFHYWKLDYTLLNRDTGCAI
ICMSKKLDLLDDTGRMHHGNAQEFALKHGAGEEVASKIVTIIHDCEKKFERDDDECLRVLEVAKCFRTGIHDLD

WQPKVEVIVSEVFTDM*
>AipsGOBP1
MTQPGQVLVLVLLAAAALADVNVMKDVTLGFGQALDKCRQESDLTEEKMEEFFHFWRDDFKFEHRELGCAIQ
CMSRHFNLLTDSSRMHHVNTEEFIQSFPNGEVLARQMVALIHGCEKQFDHEDDHCWRILHVAECFKHACVAH
GVAPSMEMMMTEFIMEAEAR*
>AipsGOBP2
MTLRCCLLLVVVAAVTRSVVGTAEVMSHVTAHFGKALEECRDESGLSAEVLEEFQHFWREDFEVVHRELGCAII
CMSNKFSLLQDDSRMHHVNMHDYVKGFPNGEVLSGKLVELIHNCEKQYDTLTDDCDRVVKVAACFKVDAKA
AGIAPEVAMIEAVMEKY*
>AipsOBP1
MDISKRRSKNAFRRLLVNTWLRLVQIFTCLSAPPVVSADVTSKCQGSKYENECDKLTCVFRKAKWLDGNAVDKA
KLITYFEQFEKDHPEWAPAMQNVKTSCLGAELKTQGVFLNCPAYDVMHCVLGSFIKHATPTQWSTSASCSYPR
AYAAACPICPEDCFSAQVPFGSCNACYLPPRTP*
>AipsOBP2
MSKFTCLVLCVVAASISRVHADDDANKAAFREAFKPILDECSKEHGVSNDDIDAAKKAGSADAIKPCFFGCIYKK
AEVFNAKGEYDVDSALSKLKKFVPDEAKFAKYAEIGKKCASVNEKPVTDGDAGCERGAMLTACFLENRAEMLI*
>AipsOBP3
MIRSCRCLVFAAVFQVVLGQGLTGTDSGPPGFQRPQSYVPKHCFAPPPGVDLHTCCPIPQLFPDEDMESCGIQK
LTKEQYENPSPARIPCQESICLLRNANLLKQNNSIDYEKMGDFVDNWAKMDPDFTIPITNAKKVCLIEGGPPAPP
VCEPDRIFTCLTSYVLWNCKLRLDSGEGCKILKEHMDGCRPFLAGP*
>AipsOBP4
MFGYQFLSFAAALICFGSSYALTSEEEANIKEAFHPFIMKCAEEYGITEEQFEEAKEKHSAEGIDPCFMSCFMKES
GFFDSAGKFDADKTKEFVDAHLTSERAITFMEAVGSECAKVNDEEVTDGDKGCDRAKLMWGCIQDLKEKMEG
SE*
>AipsOBP5
MKYFVLFVALVAGIHANVTLPPEQSEKALKTASECIKETGVSKEVLAEAKKGHIADDEGLKKFTLCFFKKAGIVDN
DGKLNLETALAKLPPGVDKAEAKKVLEGCQAKSGKTPQDTAFEIYKCYHAGAKTHIALAGI*
>AipsOBP6-PG
QRENKGASLKPLSVCCDIPELGDPKHLAKCSNPKLPGPCNDVQCVFEESGFLTDKNTLNKEAYRNHLKQWEEN
NKGWTVAVDKAIKECVDNDPRQHLDIPCKAYDVFTCTGIAMLKKCPDSAWKC*
>AipsOBP7
MSKFTCVLCVVALSLSSVYVTRAHKPNLRDAWRSELDECAKEYPVTNDEIDTAVRSGDSSNLNPCFNFCVFNKT
GFFTENGEYDLKNGLIKLRKAIRDDEEYTKFEEVATECTEDKNTSCDEKAKCDSANRLSLCFLRFKDKVRI*
>AipsOBP8
MYLRSTNGGVRSFPLGESAYTTKIVEICSKETGLKKQVPPEEKEIKFSQRKGLREFNDCYLAKTGVTTSDGKLNIDE
ALEKLPPGFAKPFVEHCQANIILGYIEENVNDFSTCFHQEVQNHLLSFYGFENYWVMLVLGTSFDKTRFTTLFFD
KHFDFWLAERAGFVNL*
>AipsOBP9
VFICGVLSLNVKASSLDELKMKYVEMIIECSDTYPITAADTLQLKTKTMPDNESIRCLFACVYKKAGMMNEQGEL
SVEGVNEMTRRYLSDDPDKIKKSEQFTEACKSVNDVPVSDGTRGCDRAALIFKCTVEKSPDFDLL*
>AipsOBP10
CYRLKTQYSVFHFRKIKRYVLRFSNYVFFISDPFPGFKKCKSADEECVLENAKAGVVPFVNGIPEFGVNKLDPDFL
R*
>AipsOBP11

MTYKVFILVFLTYVSLATSALAPFITKCKWDDSKCIKESAQKVIPLFADGIPDLHVEKHDPLLIKRVDASSPNLKLIVT
DIEVKGLKNCEAKKITRDLKAMKLSVKFLCAVDFKGVYDMKGQLFVLPIEGNGDLTAHVPKIQLNAEVDMVDKT
GKDGKKHWGVKSWRHSFELKEKSNVKFENLFPDNEFLRKTTEELIASNGNDVIVEVGPEIIKAVTAKVIESIKKLF
DEVPVEELAIDE*
>AipsOBP12
MYSGTIFLFSFILLIVSNVTFVSSQMTREQVKNSGKLVKKTCSAKNDLTEDEVKDVDKGKFIEEKKFMCYVACVYK
MGQAVKGNSLNHDMMIRQVDMLFPADMKAPVKAAIEHCRPVAKKYKDICEASYWTAKCVYEFDPPNFMFP
*
>AipsOBP13
MVLIYIVKFLILVAMCEAMTMKQIRNTGKMMRKSCQPKNNVEDEKIDPIAEGIFIDEPEVKCYMACIMKMANT
LKNGKLNFDAALKQADLLLPDDIKEPAKEAIIACKKAAEGHKDICDVSFHVTKCIYNQNPGIFYFP*
>AipsOBP14
MFDPKTVFYLLTVFSVCFGAVDIRKYLKVCDRNAIDVSDCLTDAVQKGIAVMVNGIEELGVPPIDPYLQKEFRVEY
NNNQIAVKMVIKNIYVEGLKDAKVHDARLRADDDKFHLEVDMTSPHVFVKAHYHGEGQFNSLKVVAYGDFNT
TMSDLVYTWKLDGVPEKNGSETYVRIKEFYMRPDLSSIVTSFRNENPETRELTELGARFANENWRTLYKEFLPYA
QANWNRIGVRIANKLFLKVPYDQLFPSSS*
>AipsOBP15
MDHNRLCLLVIAMFLATGSDAMTRQQLKNSGKILKKNCMNKHQVTEDQIGTIEKGKFVEDKKVMCYIACIYEL
TSVIKNNKLNYESSLRQIDIMYPADLKESAKAAVENCKDVQKKYKDICEASFHTAKCMYDFKPEDFIFA*
>AipsOBP16
MFPGSIPFISGCVHLGVSNYFRSTQSNLVVHYEDDQIVDAIYNCQDENGFDEVLSNSTNLEENFPEKEGLKKSN
DCFLKKTGFVTSDGKLNIDKTLEKLPPSFVKPIVEHCQANIALNYTTESVENFSSCYHDGILNHIFAATEVGIFPFIQ
TWKFFVPGTSFADTILILN*
>AipsOBP17
MNQLLVFVLIVACVRISNGMTREQVKKTMTVIKKQCMPKNSVTEDQIGKIEQGVFNEDRNVMCYVACVYKSL
QVVKNERLDLGLISKQIDALYPPELKEPTKKAVSQCINIQDSYNDLCEAVFHSVKCLYEKDPATFIFP*
>AipsOBP18
MKTLFVFAACILLAQALTDEQKEKLKKHRTECLTETKVEEALVNKLKGGDYKTESEPLKKYALCMMTKSELMTKD
GKFKKDVALAKVPNAADKPSVEKLIDACLANKGNTPHQTAWNYVKCYHEKDPKHAIFL*
>AipsOBP19
MFTGTVPFVLCLVAVAFGGKDKPVFSEEIKEIIQTVHDECVANTGVAEEDITNCENGIFKEDPKLKCYMFCLMEE
ASLVDDDGTVDYDMLVSLIPDEYYERTTKMIFACKHLDTPDKDKCQRAFEVHRCSYEKDPDLYFLF*
>AipsOBP20
MLVINATDYDYEGYGTGNMGEKLLTSVPRPASSSNNINNNDTSRTRRSEPLLNKPDLDQCLSQCVFANLQVVDS
RGIPREAELWNKVQSSVTSQQSRSALHDQIRACFQELQSEAEDNGCSYSNKLERCLMLRFSDRKVEGKASTPKP
ASTEQS*
>AipsOBP21
MLKFSVVCLYFSVAAVNFWNVHCISEDEKKAFIEAMKPMVEECGSDCGLTEEDYKKHSKGEDMDPCFKKCMM
QKLGFLDEDGKYNRKQLHESISEYTGDKDEAKRVQEQLDSCFDANGDNDGDDEESQMKRVDVLFKCLKEIKE*
>AipsOBP22
MSMWFRAMVVVGALAAARCGVVMDEDMAELARMVRESCVDETGADVKLVEAVNGGADLMEDDKLKCYI
KCTMETAGMMSDGEVDIEAVMALLPPEMAEHNGPALKSCGTQRGADDCDTAWKTQVCWQNANKAEYFLI*
>AipsOBP23
MSKFTYLVLCFVAVSRVYANEDERAAFHEAAKPILVECSKENGVSFDKLKAAKEAGSADGIDPCFFSCVFKKTGVF

NSKGDFDLDNSLTKLKEFVSNDEDYAKVAEVGKKCESVNEKDVSDGEAGCERASLLTACFLEHRAEIPV*
>AipsOBP24
MAKLLLAMILTVMTFALTMSATTKDAGTKEAIMTTTVANQDSSIDSNDVDVLAVMNVCNESFRIEMSYIQALN
ESGSFVDETDKTPKCFIRCVFENVGIVSEDGRMFNPARAAVIFAGERNGKPMDDIADMTALCAADRKETCPCD
RSYQFLRCLMSMEIERYEKS*
>AipsOBP25
SRKLREAMRPIIEQCSKEHGVTDADIQASKDSNNAASLPDCFNHCLFEKSGFIDKNGRYDRDSGLKNLSKYLKD
VNQYNKVVEVTKECASVEEKPATGCELGTRLTACLLDHQTSILI*
>AipsOBP26
MSKFTCIVLFVVAASLTKVTQAVSEEEKAVAREAMAPILAECSKAEGVSDEDIEEAKKNPSVDAVNSCFIRCVMR
KTDALNEKGLFDSDAALAKIRPFVKSDEDFAKFEEIGKACMSVNDKEVSDGEAGCDRAKLLLACFLEHKAEMLY
*
>AipsOBP27
DSAISADAESRCRNPPTAPQKIERVITLCQDEIKLSILREALDVIKEEHTMPAQRRRDKREVPFTHDEKRIAGCLLQ
CVYRKVKAVDGYGFPTLEGLVGLYSDGVNERGYFMAVLEASRECLMKNHDKFSRTMPMDNGRNCDVSFDIFE
CISDRIGEYCGTSGL*
>AipsOBP28
MTHIFSSFIPYMITVSMFSFPVSVKIISPTVPVTVVSSSVIITFMFDMYRLVILSIVAVTTVVADTDLQECRRLVHPH
SMRCCKKSADAKEKMMKNDDLKECFDLPKDPVKCEHELCMAKKKGITTSDDKLDKAKFEEVVTKDIDDKDLVA
DIKANCINGDLTKYGPPDFCDFVKMRHCMSMQILNHCTEWNDFGDCPQLKSIIGDCVKLVAA*
>SlitOBP9
MCLVKYHVLVLCVILVGSYALNCRSSGGPKEAELKNIYKKCLKMQEGKNSSKGNSAQDWKEPRVQIQRNDWD
RGRVGSKENKNSRDDSRSGSKDKKGDSGMRDNRNDMMSRRDDMMSRGDERNDNRKHRTDDRMGNDN
DRSGNRGRGNKNNRNDMNGGRDDRFGRDDYFNGREDFPQSDEYGGDMGQYNNNYYSTTQSSRRYKRER
RPSNSGQRSQYNPNNHKISGYEDNFRSDERNTTDNNSSKETDNKSCALHCFLENLEMTGEDGMPDRYLVTH
AITKDVKNEDLRDFLQESIEECFQILDNENTEDKCEFSKNLLICLSEKGRANCDDWKDDLTF*
>SlitOBP10
MVRKISGLLCCLCVFGISFSDSAISADSESRCRNPPTAPQKIERVITLCQDEIKLSILREALDVIKEEHTMPAQRRR
DKREVPFTHDEKRIAGCLLQCVYRKVKAVDGYGFPTLEGLVGLYSDGVNERGYFMAVLEASRECLMKNHDKFS
RTVPMDNGRNCDISFDIFECISDRIGEYCGTSGL*
>SlitOBP11
MKSFVVFCIVFVVGVCATEKGNKIASECIKESGVKSDVLAEAKKGNLGDDPAFKEFTYCFFKKVGIVGEDGKLNR
DVAIAKLPSGVDKAEAEKLLDSCKSKTGKDAVETVYEIFKCYQHGTKSHIMFAS*
>SlitOBP12
MKTLFVFAACILLAQALTDEQKEKLKKHRTECLTETKVDEELVNKLKGGDYKMDNEALKKYALCMMMKSELM
TKDGKFKKDVALAKVPNPADKPTVEKLIDACLANKGNTPHQTAWNYVKCYHEKDPKHAIFL*
>SlitOBP13
MITSCLLVLSAVVQVLLAKQPVFESGPPEPWGPPERTSHPGQFQPRVPKRCWVPPQRINVYNCCPIPTLYPDE
DMQSCGFEKLSENKPQKPVYRPEGTCKEGYCVMGKFDLLLANNSVDYVKFREYLDNWAESYPEFANAIHIAKE
ECAQDGGPEVPPICEPDKLFLCLTSTIFWNCKLRDGEGCAALQEHMNECKQYYTRVMAPTIKDFEVR*
>SlitOBP14
MVKVTYIALFVVAVSLSSVQADDKNSKPEFNLDTITFQCAQKFDISEEQFSKAIMTFDASLLAPCFWS
>SlitOBP15
MYSINCFIFSVILIVMFDNCFVYSMTREQIKNSGKLIKKTCSAKNDLTEDEVKDVDKGKFIEKKDFMCYIACVYK

MGQSVKGSTINHDMMLRQVDMMFPNDMKAPVKSAIEHCRPVAKNYKDLCEASYWTAKCIYDFDPANFMF
P*
>SlitOBP16
MYRFVILSIVLVSALADDIDIRECGRIFHPPPHGCCKANNAVKNKDMLAEELKDCFDGSGPKDPMKCEIDLCIAK
KKGFATDDGKLDIKKFEEVITKEVGSDKDLLDEIKTNCINGDLNNYGPPEFCDFMKIKHCVTLHMMNHCSEWS
DDGNCKVVKELVGKCAKVI*
>SlitOBP17
MKTFRLLCCILSIFLFFDQSYGMTRQQLKNSGKLMKKSCMPKNDVTEDEVGDIEKGKFIETRNVMCYIACVYT
MSQVVKNNKLSYEAVIKQVDVMFPAEMRDAVKAAATHCKETTKKYKDLCESSYWTAKCMYDYDAQNFVFP
*
>SlitOBP18
MFKLCVFLALGFVACHGAPNSSPGTPNANPGTYCGVTPDNIYRCLNNPRVVTPEVSTKCGSQFTECEKMTCIF
RELKWSKRGAIDKAKVRAYFDQYETEHPEWAQAVQHVKAFCLASELRAQGVFLNCPAYDIMQCVLASFIKHA
SPSVWSTATDCAYPKAYAADCPVCPSDCYSPQIPFGSCNACYTQPRTV*
>SlitOBP19
MFRRTLLLFSIIYISACNGQTEAPEKNRMMGIDAVHDNNVKIDKDTIITRNLKLEKRSRGPKSVSNKNEDQIEPD
WSYANFPKEVSEHVEKFKKNMTECLKEVQTSDKRPVKRLSPKMESPVHGECLIACVLKRNGVIINGKVNKDNLI
ALVSKFYSKDTRLMKKLEKNLDRCIEMSVRAQDDCALALVLNDCTNDLMASNKHKIMVNY*
>SlitOBP20
MEKILIFTFITLSGFAHARISVMYAHDKLSDLVAQQCLSEMYPKNKRIEIQESDEPCIIFCVLKKFGIISASGVINLDI
YRKRVQIAHQLDQKTSIMDYGGSCMENAEATQHKQDVCKKAKVFNDCTHLYRILLM*
>SlitOBP21
MARRQRGAMFTEALPLFVILVAVTHGGKNKPVFSDEIKEIIQTVHDECVAKTGVAEEDITNCENGIFKEDAKLK
CYMFCLLEEASLVDDDDTVDYDMLVSLIPDEYYERTTKMIFACKHLDTPDKDRCQRAFEVHKCSYEKDPDLYFL
F*
>SlitOBP22
MSKFTCIILCVVAASLTKVSHAAVTEEEKEAFREAMAPIIAECSEEHGVSEADIKAAKESASADNIKPCFLGCVMK
KIEVLDAKGLYDAETGLGKLRKFVKDDDEFAKFEDIAKKCLKVNDESVSDGEAGCDRAKLVLGCFIEHKVEMPF*
>SlitOBP23
MAKFSCLVLCVVAASLGSIHVASGESLRESLRPVIVACSQEHGVTDAEIQAAKDAGSPASIKPCFIACVFKKAGFI
NEQGQLDLETGLKNLRQFVKDDEQYKKLEEVAKKCSQVKDKAVSDGAAGCERGVLLAGCFLEHKTSIII*
>SlitOBP24
CVYKKTEFLNSKGEYDVDTALAKLKKYISNDDDYAKLSQVGKDCASVNSKPVGDGEAGCERGVLLTQCFLDHK
>SlitOBP25
MAKVTCIVLFVVGVSLSSIQADDGKNESEVEIDVNQIIDDCIEEYHIPRRLFLAAAETGSTHALTPCFWSCCFKGV
GVLNSEGQYDIDATLDLSKKIFTDHEYEKVEIIVKKCESVNGAPVSNGNIECEKSVLLADCLFDNAKKHFPNMFG
VDY*
>SlitOBP26
CFLACMMKQIGVMDDNGMVQKETALEMAKAVFDDPEELKAIEDYLHSCSHINTESVSDGAAGCERAMLAYK
CMTENASKFGFDI*
>SlitOBP27
MYKFVILCSIFVAASNADVAQTLTKRETKASLKPLSVCCDIPELADEFQLAKCSPRPPGPCEDVQCIFEVSGFLTD
RNTLNKAAYRSHLQKWEKNHPGWTDSIYKAITDCVDNDPRQHLEVPCKAYDVFTCTGIAMLKKCPDTAWKC
*

>SlitOBP28
MIVRFLLCLYIVEFYGAHARTDQEIKAWFFREGMDCNIEHPISPKEMLELKENKIPDTNNAKCFVACVFKKTGM
LDSKGMFDAENSIAMTQKDFANDPNRLESSKKLLEACKKVNDEAVSDGEKGCERSVLLHKCFVETAPQLGIKLP
*
>SlitOBP29
MWNLLVVFLAICSCVYARRRSSGAEINGLTEEELKMEFTKLIMKCNKDGEVDMTELVQLQNYVVPTKQSTKCV
LACAYKAAEVMNAKGEYDIDHAYKVAEMMKNGDEKRLVNAKKMADLCVKVNELSVSDGEKGCDRAAMIFK
CTVENAPKFGFKL*
>SlitOBP30
ALSMDDLKQKYVDNILECSKQYPIDRADAEQLQNRIMPDKEPIKCLFACVYKLAGMMNDQG
>SlitOBP31
RNEHREKEKMNKLTNIFASILFVLFSFAFYLTISFTPLTKDEQMERYNKMTENVEPFRKNLTECARQVKASMAD
VENFMKRIPQASLQGKCFVACILKRNSIIKNNKISKEHLLEANRA
>SlitOBP32
RIDRNDIPCIIHCVLKKFGIMTNDGYINIKNYYRRVQAIHRYDPRILISDVGETCAQNINGMNLDHDVCKKAKVF
NDCTQLYAVSYRDPDEWK*
>SlitOBP33
MTCSQALALLALVAISQQATTGCKNCIMLGKEEKAMFRAHSDACVAASRVEPRLVDAMLAGELLDEPALRKH
VYCVLLKCKLISKDGKLQKAAVLGKMAARPDAKNATKVLESCADQTGDTPEDLAWNLFRCGYDKKALLFDYM
PTNVASETDNNS*
